# Cathodic Radical Cyclisation of Aryl Halides Using a Strongly‐Reducing Catalytic Mediator in Flow

**DOI:** 10.1002/anie.202203694

**Published:** 2022-07-18

**Authors:** Ana A. Folgueiras‐Amador, Alexander E. Teuten, Mateo Salam‐Perez, James E. Pearce, Guy Denuault, Derek Pletcher, Philip J. Parsons, David C. Harrowven, Richard C. D. Brown

**Affiliations:** ^1^ School of Chemistry University of Southampton Highfield Southampton SO17 1BJ UK; ^2^ Department of Chemistry Imperial College London White City Campus London W12 0BZ UK

**Keywords:** Electrosynthesis, Flow Chemistry, Mediator, Radical Cyclization, Reductive Cyclization

## Abstract

Electro‐reductive radical cyclisation of aryl halides affords the corresponding hetero‐ and carbo‐cycles in an undivided flow reactor equipped with steel and carbon electrodes using an organic mediator. A dissolving metal anode is not needed, and the mediator can be employed in a sub‐stoichiometric amount (0.05 equiv), increasing the practical utility of cathodic radical cyclisation. The methodology is applied to O‐, N‐, and C‐tethers, yielding tricyclic fused and spiro systems. In the absence of mediator, the major pathway is hydrogenolysis of the C−X bond, a 2 e^−^ process occurring at the cathode. Predominance of the radical pathway in presence of a strongly reducing mediator (**M**) is consistent with homogeneous electron‐transfer in a reaction layer detached from the cathode surface, where the flux of **M** ^.−^ leaving the electrode is such that little aryl halide reaches the cathode.

## Graphical abstract

Cathodic radical cyclisations of aryl halides have been achieved in an undivided flow electrolysis cell, using phenanthrene as mediator in loadings down to 0.05 equiv, and without the requirement for a sacrificial anode. It is proposed that mediated homogeneous electron transfer proceeds in a reaction layer detached from the cathode, accounting for the observed selectivity.

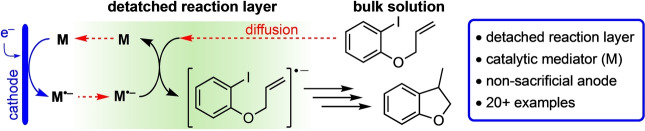

## Introduction

Radical cyclisation reactions have long since joined the realm of fundamental methodologies in organic synthesis. In particular, the usefulness of reductive radical cyclisations of aryl bromides and iodides has been widely demonstrated in spite of their ubiquitous association with the use of stoichiometric levels of toxic or expensive metal hydrides as mediators. While these concerns have been addressed, in part, by the development of catalytic procedures, alternative mediators, and efficient purification protocols, none have emerged as the go‐to replacement for tributyltin hydride in its myriad applications.[^1^, ^2^]

**Scheme 1:**
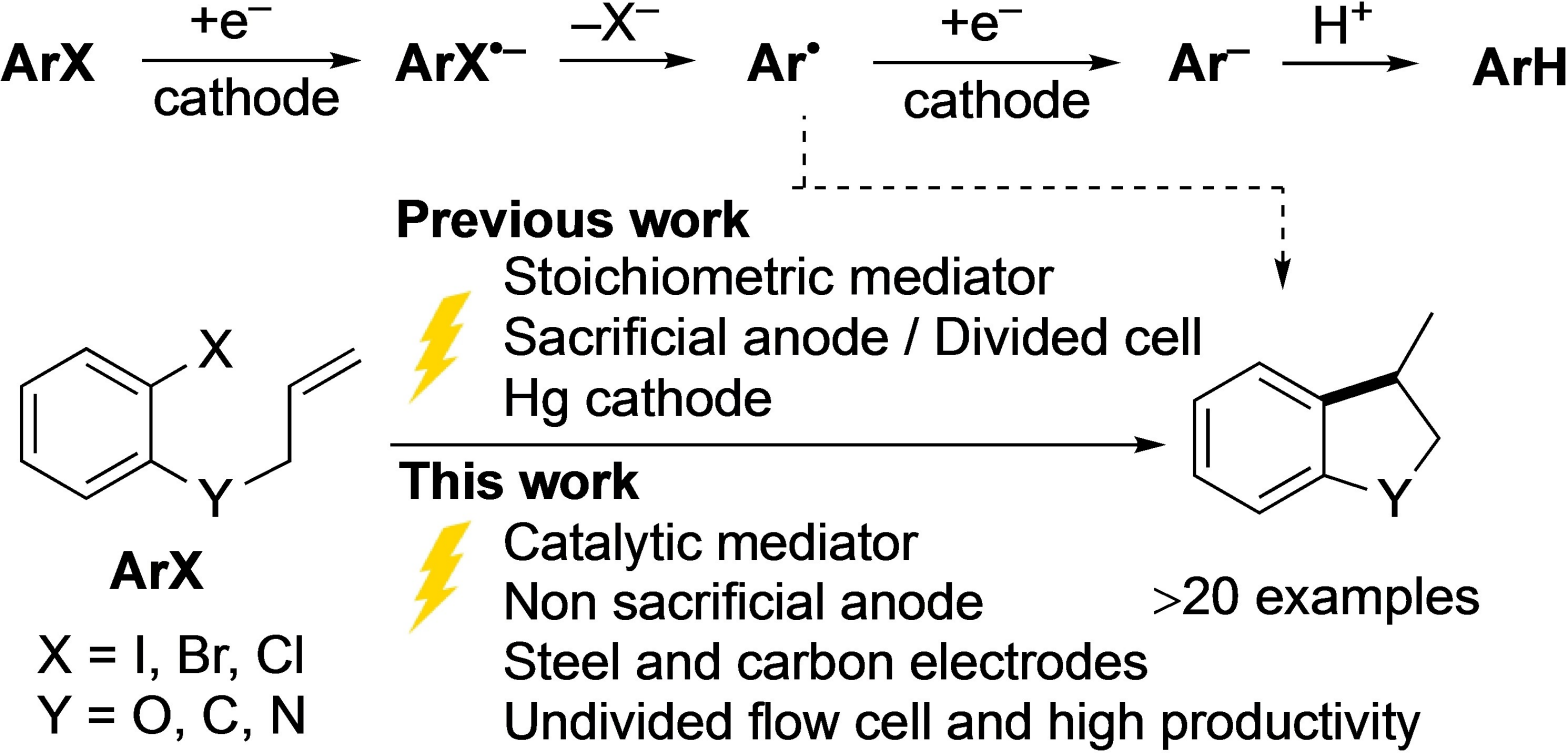
Electrochemical reduction of aryl halides. (top) Direct reduction following an ECE mechanism. (bottom) Reductive cyclisation in presence of mediator.

Reductive electrochemistry appears to offer an ideal means to generate aryl radicals from aryl halides, without the waste associated with traditional metal hydride reagents (Scheme 1).[^3^, ^4^] As a direct cathodic process, however, synthetic utility is highly limited due to competing reduction of the aryl radical and protonation (Scheme 1, top), and this ECE reductive‐dehalogenation pathway typically outpaces cyclisation.[^3^, ^5^] Frangible radical anions (**ArX** ^.−^), arising from single electron‐transfer, rapidly lose halide ion with the consequence that nascent aryl radicals are formed very close to the cathode.^[3]^ As the electrode provides an infinite source of electrons, and the reduction potential of aryl radicals are well positive (≈0 V, vs SCE) to aryl halides,^[6]^ transfer of a second electron is highly favourable leading to the aryl anion, and subsequent protonation.^[3]^ Thus, direct cathodic cyclisation is restricted to aryl halides that give rise to radical anions that are sufficiently long‐lived to diffuse away from the cathode prior to loss of halide ion, and systems strongly predisposed towards cyclisation of the aryl radical intermediate.^[7]^

Increased generality of aryl radical cyclisation has been achieved using electrochemically generated Ni^I^ species,^[8]^ or by using arenes in stoichiometric equivalence or excess as outer sphere electron‐transfer mediators.[^7d^, ^9^, ^10^] Mitsudo *et al*.^*[*9c]^ and Kurono *et al*.^[9b]^ reported cathodic cyclisations of aryl halides using fluorene (1 equiv) and phenanthrene (2 equiv) as mediators, respectively. In these studies, sacrificial Mg anodes were employed,^[11]^ while others also required sacrificial anodes, divided cells and, in some cases, undesirable cathode materials such Hg. Recently, radical cyclisation of an aryl chloride has been demonstrated using a very strongly reducing excited radical anion, generated photo‐electrochemically in a divided cell.^[12]^

Flow electrolysis cells provide a convenient platform for laboratory electrosynthesis, with enhanced rates of conversion due to efficient mass transport and high electrode area to reactor volume.^[13]^ Narrow gap configurations also facilitate operation with reduced amounts of supporting electrolyte, and scale up from mg to g scale is readily achieved using the same flow cell. For reductive electrosyntheses, identifying a reaction that does not interfere with the chemistry on the cathode is a common challenge, frequently leading to the adoption of a sacrificial anode or divided cells. In undivided narrow gap flow cells, however, implementation of sacrificial counter electrodes that erode with concomitant release of inorganic salts into the reaction medium is undesirable, leading to blockages and a requirement for routine replacement of the anode. On the other hand, the use of divided cells limits cell current and productivity, and demands a flow cell design that is more complex than the undivided reactors that are generally preferred for electrosynthesis.^[14]^

In this paper, we describe electro‐reductive cyclisation of aryl halides in an undivided electrochemical flow cell using an inexpensive organic mediator (phenanthrene), which can be applied in sub‐stoichiometric amounts (5 mol %). Carbon (anode) and stainless steel (cathode) provide convenient and robust electrode materials, with oxidation of halide ion as a suitable counter‐electrode reaction, negating the use of a sacrificial anode. Despite both substrate and aryl radical being more readily reduced than the mediator, the mediated pathway prevails. This is explained by the formation of a reaction layer detached from the electrode, where the flux of strongly reducing phenanthrene radical anions from the cathode balances the flux of aryl halide from the bulk, effectively blocking access of the substrate to the electrode.

## Results and Discussion

Our investigation of electrochemical radical cyclisations in flow began by adaptation of batch electrolysis procedures for cyclisation of 1‐(allyloxy)‐2‐iodobenzene (**1**) using stoichiometric mediator and a sacrificial anode.^[9b]^ A commercial narrow gap, extended path flow electrolysis cell was employed,[^15^, ^16^] in the first instance, equipped with a magnesium plate anode and a stainless steel (ss) cathode (Table 1). Steel is an inexpensive and robust cathode material that had demonstrated good performance in reductive deiodination reactions.[^6f^, ^7c^] Initial reductive cyclisation was conducted in MeCN containing Et_4_NBF_4_ as supporting electrolyte on a 0.5 mmol scale (flow rate 0.25 mL min^−1^, run time 20 min) passing 2.0 F (Table 1, Entry 1). Pleasingly, the desired dihydrobenzofuran **2** was realised in 72 % yield, although the reaction proved capricious due to Mg^2+^ salts clogging the reactor channel. Nevertheless, successful formation of dihydrobenzofuran **2** offered a platform to develop more broadly applicable cathodic radical cyclisations in flow.

**Table 1:** Optimisation of cathodic cyclisation (stoichiometric mediator).

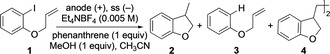							
Entry	Conc. **1** [M]	Anode & flow rate [mL min^−1^]	I [mA] (Q [F])	Yield [%]^[a]^			
				**1**	**2**	**3**	**4**
1^[b]^	0.1	Mg, 0.25	80 (2.0)		72		
2	0.1	graphite, 0.25	80 (2.0)	23	19	21	–
3	0.1	graphite, 1.0	320 (2.0)	19	37	6	1
4	0.1	graphite, 2.0	643 (2.0)	19	40	5	2
5^[c]^	0.1	graphite, 2.0	643 (2.0)	11	44	4	7
6^[d]^	0.05	graphite, 4.0	643 (2.0)	22	40	3	2
7^[e]^	0.05	glassy C, 4.0	643 (2.0)	36	49	5	6
8^[e]^	0.025	glassy C, 8.0	643 (2.0)	21	68	2	10
9^[e]^	0.012	glassy C, 16.0	643 (2.0)	30	60	1	8
10^[e]^	0.025	glassy C, 16.0	1290 (2.0)	27	63	1	7
11^[f]^	0.025	glassy C, 16.0	1290 (2.0)	22	72	0	7

[a] Yield determined using calibrated GC (except isolated yield for Entry 1). [b] Et_4_NBF_4_ [0.05 M], no MeOH, isolated yield. [c] No MeOH. [d] Some erosion of electrode observed. [e] Et_4_NBF_4_ [0.01 M]. [f] Et_4_NBF_4_ replaced with Bu_4_NI (0.5 equiv), no MeOH.

Exchanging the Mg anode for graphite under the same conditions led to a considerably lower yield of the cyclised product **2** (19 %), and more reduced by‐product **3** (21 %, Table 1, Entry 2). Selectivity for the cyclised product **2** was found to improve by increasing flow rates from 0.25 to 4.0 mL min^−1^ (Table 1, Entries 3–7), with relatively little dehalogenated product **3** (3–6 %) being formed. A total charge of 2.0 F was maintained by applying an increase in current commensurate with the flow rate. MeOH was originally added as a proton source, although similar results were obtained with and without 1 equiv of MeOH (Table 1, Entries 4 and 5). *Bis*‐dihydrobenzofuran **4** was observed as a by‐product, albeit in small amount.^[17]^

A range of working electrode (cathode) materials were examined, including Pt, Ag, leaded bronze, C, Ni, and ss (Table S1), with Ag and ss being superior in terms of selectivity and yield for the cyclised product **2**. However, use of Ag was limited due to deposit build‐up on the electrode surface, and therefore, further investigations used a ss cathode. Different anode materials were also tried; carbon‐based ones such as graphite, carbon polyvinylidene fluoride composite (C/PVDF) and glassy carbon gave higher yields and selectivity towards the cyclised product **2** (see Table S2). Ultimately, a glassy carbon anode was selected for its high resistance towards erosion, which is important when applying higher cell‐averaged current density (>30 mA cm^−2^; >600 mA).^[18]^

Using a steel cathode and glassy carbon counter electrode, higher flow rates of 8 and 16 mL min^−1^ improved yields of the dihydrobenzofuran **2** further (60–68 %, Table 1, Entries 8–10). At these high flow rates the concentration of aryl iodide **1** was decreased so that the cell current required to drive conversion of starting material did not exceed 1.3 A, and excessive Joule heating of the solution was avoided. A final improvement in the yield derived from exchanging Et_4_NBF_4_ with *n*‐Bu_4_NI (TBAI), as the latter performed a dual role as sacrificial salt and supporting electrolyte, giving the dihydrobenzofuran in 72 % GC yield with minor amounts of dimer **4** (7 %) and some unreacted starting material **1** (22 %, Table 1, Entry 11). The level of conversion at such a high flow rate is remarkable in view of the very short residence time in the electrolysis cell (<4 s), highlighting the efficient mass transport and high productivity (≈16 mmol h^−1^) achieved in the small gap/extended channel design.

Having established selective cathodic cyclisation in the absence of a sacrificial anode, the need for stoichiometric phenanthrene mediator was investigated (Table 2). Using 0.5 equiv of phenanthrene, selectivity for dihydrobenzofuran **2** was retained, albeit in lower yield (49 %, Table 2, Entry 2). The short residence time in the electrolysis cell (*T*_r_≈3.75 s) at 16 mL min^−1^ flow rate may not be sufficient when using sub‐stoichiometric mediator. Indeed, repeating the reaction at 2 mL min^−1^ gave an improved yield of **2** (78 %) with higher selectivity (Table 2, Entry 3). Furthermore, a 75 % yield of **2** was achieved using 0.05 equiv of mediator, with minimal formation of dehalogenated material **3** (Table 2, Entry 4). Increasing the current to 240 mA (3.0 F) gave cyclised product **2** selectively with high conversion in 82 % yield (Table 2, Entry 6). A smaller interelectrode gap (0.25 mm) gave the cyclised product **2** in 80 % yield, but with more dehalogenation (Table S3, Entry 17). Finally, in the absence of phenanthrene, reductive dehalogenation to **3** was a major pathway, with **2** only formed in low yield (25 %), affirming the involvement of mediator (Table 2, Entry 5). Other carbon materials (e.g. C/PVDF) could also be employed as anode (Table 2, Entry 7), but glassy carbon was retained due to its high resistance to erosion.

**Table 2:** Optimisation of cathodic cyclisation (sub‐stoichiometric mediator).

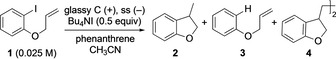							
Entry	Phenanthrene [equiv]	Flow rate [mL min^−1^]	I [mA] (Q [F])	Yield [%]^[a]^			
				**1**	**2**	**3**	**4**
1	1.0	16.0	1290 (2.0)	22	72	–	7
2	0.5	16.0	1290 (2.0)	19	49	–	6
3	0.5	2.0	160 (2.0)	18	78	1	5
4	0.05	2.0	160 (2.0)	18	75	4	5
5	0	2.0	160 (2.0)	23	25	48	–
6	0.05	2.0	240 (3.0)	7	**82** ^[b]^	2	8
7^[c]^	0.05	2.0	240 (3.0)	7	78	4	8

[a] Yield determined using calibrated GC. [b] Reaction was repeated five times with yields 81–83 %. [c] C/PVDF anode

Different aromatic mediators were investigated under the optimised conditions (see Table S5), giving comparatively lower conversions and selectivities than phenanthrene. Of the other mediators investigated, naphthalene gave the highest yield of cyclised product **2** (68 %) with 11 % of the dehalogenated product **3** (Table S5, Entry 3).

Two sets of conditions were established for electrochemical radical cyclisation of the model substrate **1**; using 1 equiv of phenanthrene at high flow rate (16 mL min^−1^), 1.29 A current, and passing the solution twice through the cell, the cyclised product **2** was given at a high rate of production (16 mmol h^−1^). Alternatively, using sub‐stoichiometric mediator (0.05 equiv) at 2 mL min^−1^, gave a higher product yield while maintaining a good rate of production (2.5 mmol h^−1^). The conditions optimised in flow were also found to transfer to batch electrolysis in an undivided cell (see Supporting Information), giving **2** in 74 % and 64 % unoptimised yields (GC) using stoichiometric and catalytic mediator, respectively. When 5 mol % of phenanthrene was applied in batch, rather more of the dehalogenated by‐product **3** (21 %) was obtained. This difference in selectivity is likely due to the different mass transport characteristics of batch cells.

To illustrate the scope and potential of the method, cyclisations of a range of aryl halides with appended alkenyl groups were investigated in flow (Table 3). First, cyclisation of bromo‐ and chloro‐ analogues of allyl ether **1** were found to proceed well but gave lower yields of **2** than was obtained from 2‐iodophenyl ether (Table 3). In general, cyclisations of aryl chlorides (see **11**, **14** and **19**) were found to be more challenging under the conditions optimised for **1**, which is due to their more negative reduction potential. Pleasingly, aryl bromides and iodides generally performed well in cathodic 5‐ and 6‐*exo*‐trig cyclisations without further optimisation of conditions, with only minor amounts of dehalogenated byproducts. Five (**2**–**19**) and six‐membered (**20** and **21**) cyclic ethers were obtained in moderate to high yields, including tricyclic and spirocyclic (**12**–**17**) examples. Compatibility with electron‐withdrawing and releasing substitution, carbamate, ester and nitrile functionality was also demonstrated. Pyridine and spiropiperidine systems are of particular interest in the context of small molecule drug discovery as exemplified by **15**, which is an intermediate towards a potent human mast cell tryptase inhibitor.^[19]^ The cyclisation to obtain nitrile **7** was also performed on a 25 mmol scale using 0.05 equiv of the mediator, giving 71 % yield, highlighting the ease of laboratory scale‐up of the flow method in the same reactor.

**Table 3:**
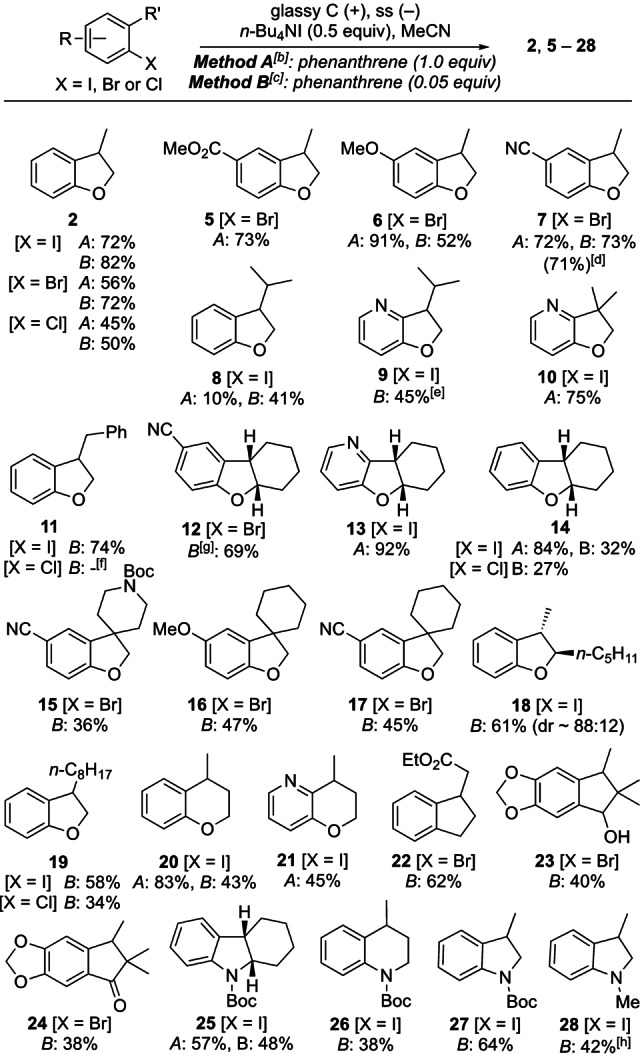
Illustration of substrate scope for cathodic cyclisation of aryl halides.^[a]^

[a] Isolated yields are reported, except for **2**, which is from calibrated GC analysis. Reactions carried out on 0.5–25.0 mmol scale in the Ammonite 8 flow electrolysis reactor. [b] Method A (two passes through the cell): ArX (0.025 M), 16 mL min^−1^, 1.28 A (2.0 F each pass). [c] Method B: ArX (0.025 M), 2 mL min^−1^, 240 mA (3.0 F). [d] Reaction carried out on 25.0 mmol scale, conditions B. [e] Reaction carried out on 5.0 mmol scale. [f] Starting aryl chloride recovered (76 %). [g] Phenanthrene (0.2 equiv). [h] Reaction carried out on 7.0 mmol scale in THF/CH_3_CN (7 : 1).

The substrate scope extended to *C*‐ and *N*‐ linked systems, giving 5‐ and 6‐membered products **22**–**28** (Table 3). In the case of the electron‐rich *N*‐methylaniline system **28**, partial iodination of the cyclised product was observed *para* to the nitrogen. Direct quenching of the electrolysis solution into thiosulfate as it flowed out of the reactor did not change the result, and further iodination was not seen when the product mixture was subjected to I_2_ or Bu_4_NI_3_, indicating the involvement of a transient electrochemically generated species such as [MeCNI]^+^.^[20]^ Interestingly, iodination was supressed by changing the reaction solvent to THF/MeCN (7 : 1), giving the indoline **28** in 42 % yield (7 mmol scale). This side reaction was not found to be problematic in related cyclisations of aryl ethers and Boc‐protected anilines.

**Figure 1:**
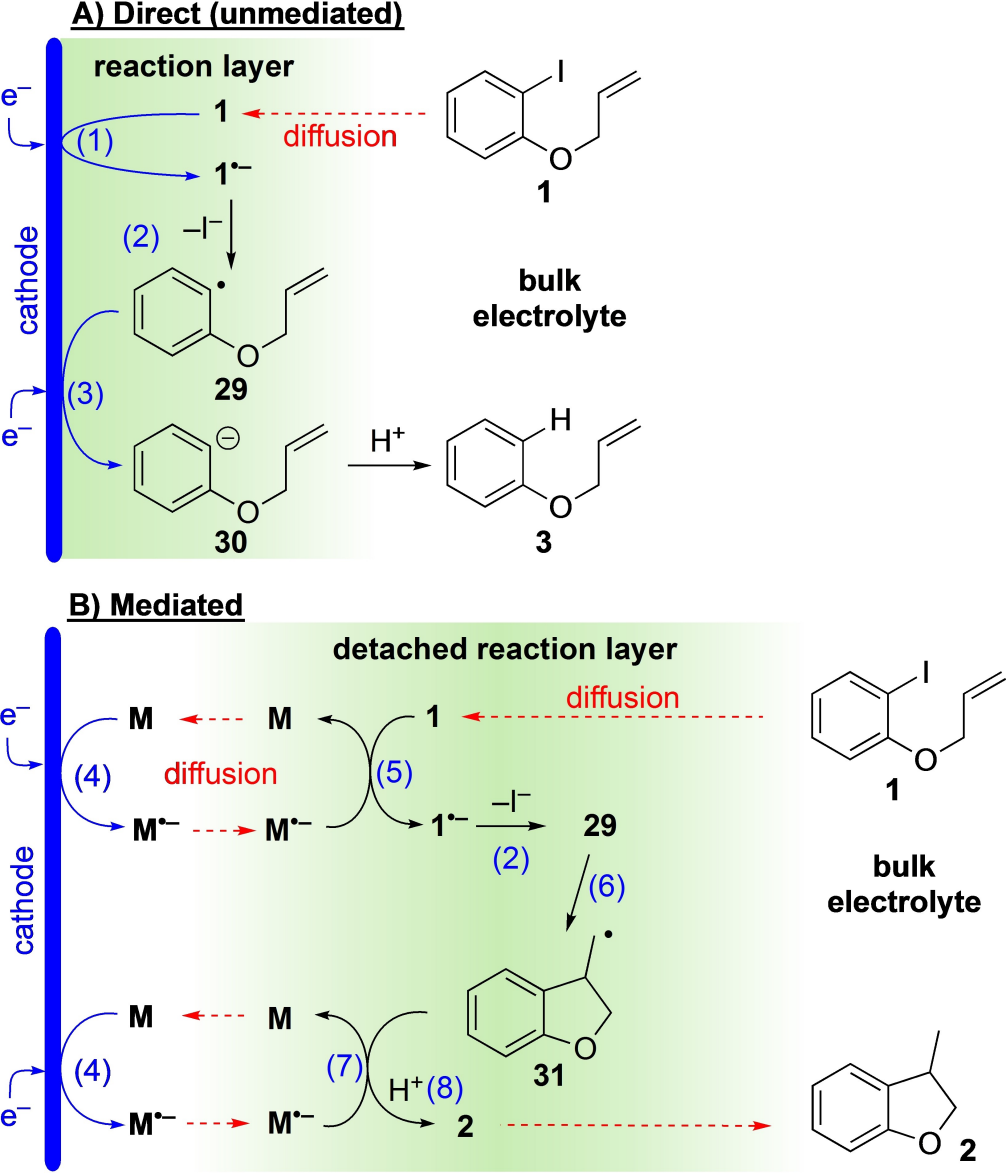
Simplified overview of electrochemical and chemical reaction steps for: A) unmediated cathodic reduction of aryl iodide **1**; B) mediated reductive cyclisation of aryl iodide **1** in presence of phenanthrene (**M**) showing a reaction layer detached from the cathode. Dashed red arrows indicate diffusion.

Simplified mechanisms for the unmediated and mediated electrochemical reactions are presented in Figure 1 (see Supporting Information for a more complete description, including experimental support for the involvement of aryl radicals in the mediated reaction via cyclisation to a vinylcyclopropane with concomitant cyclopropane‐opening). It is important to recognise that electron transfer (ET) steps (1), (3) and (4) are fast processes that can only occur when reacting species are immediately adjacent to the electrode surface. On the other hand, homogeneous electron transfer steps (5) and (7) occur via ET from the radical anion of phenanthrene (**M** ^.−^), which is a source of a single electron, and can proceed anywhere in solution where **M** ^.−^ and **1** or **31** are present. Thus, in the absence of a mediator (**M**), fast loss of iodide from radical anion **1** ^.−^ results in the formation of aryl radical **29** close to the cathode where it is reduced to its anion **30**. Protonation of **30** then affords the formal hydrogenolysis product **3**. This is evidenced by cyclic voltammetry (CV) of aryl iodide **1**, which shows a single irreversible peak (*E* ^0^=−2.2 V, vs SCE, see Figure S5 for CV) in the negative sweep corresponding to the well‐established 2e^−^ ECE reduction of **1** (Figure 1, steps (1–3)).[^3^, ^5^, ^21^]

By contrast, in the mediated pathway, radical anion **1** ^.−^ is generated wherever both **M** ^.−^ and ArI **1** are present, as homogeneous bimolecular SET (step 5) is a very favourable process due to the more negative reduction potential of **M** (*E* ^0^=−2.45 V, vs SCE).^[22]^ SET (step 5) occurs away from the electrode surface, and rapid loss of I^−^ from **1** ^.−^ is followed by cyclisation of **29** to alkyl radical **31** (steps 2 and 6). A further homogeneous SET from **M** ^.−^ (step (7)) then gives cyclised product **2** after protonation (step (8)).[^9b^, ^9c^, ^23^] Evidence to support formation of **2** by protonation as the major pathway was provided by labelling studies, where cyclisation of **1** in CD_3_CN and in THF/CD_3_CN (7 : 1) each led to substantial deuterium incorporation into the CH_2_‐H/D group of **2** (≈95 % and ≈84 %, respectively, from integration of ^1^H NMR). However, H‐atom abstraction by alkyl radical **31** to cyclised product **2** was evidenced as a minor pathway (see Supporting Information for details).

An important consideration remains, however, as heterogeneous ET's (steps (1) and (3)) may both proceed at potentials required to form **M** ^.−^. Predominance of the radical cyclisation in presence of **M** implies that the probability of ArI **1** or Ar⋅ **29** reaching the cathode is low. This situation arises under steady state conditions close to the cathode when its potential is negative to the mediator, where both substrate **1** and mediator **M** in the region near to the electrode surface have been reduced. Under such conditions, the concentration of substrate **1** near the electrode is negligible, whereas the concentration of **M** ^.−^ will be at its maximum. Reduction of the substrate becomes mass transport controlled, with the mediator acting as a charge shuttle between the electrode and the substrate that diffuses in from the bulk. The outward flux of the strongly reducing radical anion **M** ^.−^ from the electrode balances the flux of ArI **1** diffusing towards the electrode. Where the flux of **M** ^.−^ is sufficient, homogeneous ET (step 5) takes place in a layer away from the surface of the electrode, ensuring aryl radical anion **1** ^.−^ is formed in a “detached reaction layer”.^[24]^ Rapid loss of I^−^ ensues, and cyclisation of aryl radical **29** occurs before it can be reduced, either by diffusion to the cathode or in a bimolecular encounter with a radical anion (e.g. **M** ^.−^ or **1** ^.−^).^[25]^

**Figure 2:**
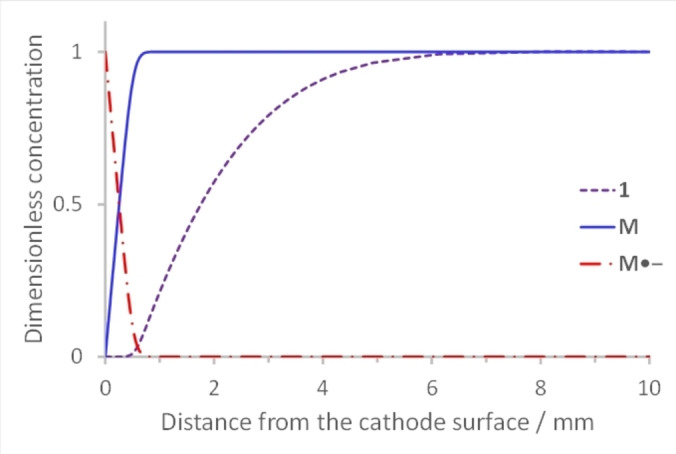
Simulated concentration profiles for **1**, **M**, and **M** ^.−^, illustrating the proposal of a reaction front away from the cathode surface. The simulation was performed with DigiElch v.8 (from Elchsoft) using a 1/1 ratio of **1** and **M**.

Simulations of amperometry data using the software, DigiElch, support the proposal that a reaction layer, where homogeneous electron transfer (step 5) occurs, becomes detached from the electrode surface with the flux of **M** ^.−^ from the cathode balancing diffusion of ArI **1** from the bulk (Figure 2, see Supporting Information for further details). Simulated steady state concentration profiles illustrate key features; the concentration of **M** ^.−^ is highest at the electrode, decreasing as it diffuses towards the bulk, encountering and reducing ArI **1** to **1** ^.−^. On the other hand, concentration of **1** is highest in the bulk, and decays to zero at a distance from the electrode indicating detachment of the reaction layer. Concentrations of radical intermediate **29** and radical anion **1** ^.−^ do not accumulate due to rapid onward reactions.

A suitable counter electrode reaction must also be available, and in the absence of a sacrificial anode, it is oxidation of halide ions (e.g. I^−^→I_3_^−^, 0.63 V vs SCE) produced during cathodic cyclisation.[^26^, ^27^] In the present work it was found to be advantageous to supplement cathodic halide ion with I^−^ from the supporting electrolyte Bu_4_NI (0.5 equiv).

## Conclusion

In conclusion, electrochemical radical cyclisation of aryl halides has been demonstrated in an undivided flow cell using phenanthrene as a mediator (0.05–1.0 equiv) without the requirement of a sacrificial anode. Although aryl iodides, such as **1**, are reduced more readily than the mediator, the mediated pathway prevails due to its spatial segregation in a reaction layer detached from the cathode surface. The flux of powerful single electron transfer agent **M** ^.−^ from the cathode matches the flux of ArI **1** from the bulk electrolysis solution preventing the direct 2e^−^ (ECE) reduction of ArI **1** to ArH **3** at the electrode.

## Conflict of interest

The authors declare no conflict of interest.

1

## Supporting information

As a service to our authors and readers, this journal provides supporting information supplied by the authors. Such materials are peer reviewed and may be re‐organized for online delivery, but are not copy‐edited or typeset. Technical support issues arising from supporting information (other than missing files) should be addressed to the authors.

Supporting Information

Supporting Information

## Data Availability

The data that support the findings of this study are available from the corresponding author upon reasonable request.
