# Supplementary material for: Cathodic Radical Cyclisation of Aryl Halides Using a Strongly‐Reducing Catalytic Mediator in Flow
Source: Angew Chem Int Ed Engl. 2022 Jul 18;61(35):e202203694. doi: 10.1002/anie.202203694 (PMC9543573; doi:10.1002/anie.202203694)

Angewandte Chemie
Eine Zeitschrift der Gesellschaft Deutscher Chemiker

Supporting Information

Cathodic Radical Cyclisation of Aryl Halides Using a Strongly-Reducing Catalytic Mediator in Flow

*A. A. Folgueiras-Amador, A. E. Teuten, M. Salam-Perez, J. E. Pearce, G. Denuault, D. Pletcher, P. J. Parsons, D. C. Harrowven, R. C. D. Brown**

Contents

1 General Experimental 5

2 Experimental setup (Figures S1–S2) 6

3 Optimisation of the reaction conditions 7

4 Radical clock experiment 11

5 Electrolysis using Bu_4NI_3 in place of Bu_4NI 12

6 Deuterium incorporation studies 13

7 Mechanistic studies: cyclic voltammetry and simulations 15

7.1 Experimental cyclic voltammograms 15

7.2 Simulations 18

7.3 Simulated concentration profiles 20

8 Flow Setup 21

8.1 Calculation for the current needed in the flow cell (I_{theo}) and charge applied (F) 21

8.2 General method A: stoichiometric phenanthrene (1 equiv.) 21

8.3 General method B: catalytic phenanthrene (0.05 equiv.) 21

9 Batch Setup 23

9.1 Calculation of the current needed in the batch cell (I_{theo}) and charge applied (F) 23

9.2 Reaction with catalytic phenanthrene (0.05 equiv.) in a batch cell 23

9.3 Reaction with stoichiometric phenanthrene (1 equiv.) in a batch cell 24

10 Reductive radical cyclisation in batch 25

10.1 Experimental procedure for the catalytic phenanthrene method: 25

10.2 Experimental procedure using stoichiometric phenanthrene in a batch cell: 25

11 Synthetic procedures 27

11.1 General Method A: Reductive cyclisation with stoichiometric phenanthrene 27

11.2 General Method B: Reductive cyclisation with catalytic phenanthrene 27

11.3 General Method C: Preparation of aryl ethers[8] 27

12 References 67

13 NMR spectra 69

13.1 1-(Allyloxy)-2-iodobenzene (1) 69

13.2 1-(Allyloxy)-2-bromobenzene 70

13.3 1-(Allyloxy)-2-chlorobenzene 71

13.4 3-Methyl-2,3-dihydrobenzofuran (2) 72

13.5 (Allyloxy)benzene (3) 73

13.6 1,2-bis(2,3-dihydrobenzofuran-3-yl)ethane (4) 74

13.7 Methyl 4-(allyloxy)-3-bromobenzoate 75

13.8 Methyl 3-methyl-2,3-dihydrobenzofuran-5-carboxylate (5) 76

13.9 1-(Allyloxy)-2-bromo-4-methoxybenzene 77
13.10 5-Methoxy-3-methyl-2,3-dihydrobenzofuran (6) 78
13.11 4-(Allyloxy)-3-bromobenzonitrile 79
13.12 3-Methyl-2,3-dihydrobenzofuran-5-carbonitrile (7) 80
13.13 1-Iodo-2-((3-methylbut-2-en-1-yl)oxy)benzene 81
13.14 3-Isopropyl-2,3-dihydrobenzofuran (8) 82
13.15 2-Iodo-3-((3-methylbut-2-en-1-yl)oxy)pyridine 83
13.16 3-Isopropyl-2,3-dihydrofuro[3,2-b]pyridine (9) 84
13.17 2-Iodo-3-((2-methylallyl)oxy)pyridine 85
13.18 3,3-Dimethyl-2,3-dihydrofuro[3,2-*b*]pyridine (10) 86
13.19 1-(Cinnamyloxy)-2-iodobenzene (*E:Z* ~ 14:1) 87
13.20 1-Chloro-2-(cinnamyloxy)benzene 88
13.21 3-Benzyl-2,3-dihydrobenzofuran (11) 89
13.22 3-Bromo-4-(cyclohex-2-en-1-yloxy)benzonitrile 90
13.23 5a,6,7,8,9,9a-Hexahydrodibenzo[*b*,*d*]furan-2-carbonitrile (12) 91
13.24 3-(Cyclohex-2-en-1-yloxy)-2-iodopyridine 92
13.25 (5a*S*,9a*S*)-5a,6,7,8,9,9a-Hexahydrobenzofuro[3,2-*b*]pyridine (13) 93
13.26 1-(Cyclohex-2-en-1-yloxy)-2-iodobenzene 94
13.27 1-Chloro-2-(cyclohex-2-en-1-yloxy)benzene 95
13.28 (4aS,9bS)-1,2,3,4,4a,9b-Hexahydrodibenzo[*b*,*d*]furan (14) 96
13.29 *tert*-Butyl 4-hydroxy-4-((phenylsulfinyl)methyl)piperidine-1-carboxylate 97
13.30 *tert*-Butyl 3-hydroxy-4-methylenepiperidine-1-carboxylate 98
13.31 *tert*-Butyl 4-(chloromethyl)-3,6-dihydropyridine-1(2*H*)-carboxylate 99
13.32 *tert*-Butyl 4-((2-bromo-4-cyanophenoxy)methyl)-3,6-dihydropyridine-1(2*H*)-carboxylate 100
13.33 *tert*-Butyl 5-cyano-2*H*-spiro[benzofuran-3,4'-piperidine]-1'-carboxylate (15) 101
13.34 Cyclohex-1-en-1-ylmethanol 102
13.35 1-(Bromomethyl)cyclohex-1-ene 103
13.36 2-Bromo-1-(cyclohex-1-en-1-ylmethoxy)-4-methoxybenzene 104
13.37 5-Methoxy-2*H*-spiro[benzofuran-3,1'-cyclohexane] (16) 105
13.38 3-Bromo-4-(cyclohex-1-en-1-ylmethoxy)benzonitrile 106
13.39 2*H*-Spiro[benzofuran-3,1'-cyclohexane]-5-carbonitrile (17) 107
13.40 1-(But-3-en-1-yloxy)-2-iodobenzene 108
13.41 1-Iodo-2-(oct-1-en-3-yloxy)benzene 109
13.42 3-Methyl-2-pentyl-2,3-dihydrobenzofuran (18, dr ~ 82:12) 110
13.43 (*E*)-1-(Dec-2-en-1-yloxy)-2-iodobenzene 111

13.44 (*E*)-1-Chloro-2-(dec-2-en-1-yloxy)benzene 112
13.45 3-Octyl-2,3-dihydrobenzofuran (19) 113
13.46 4-Methylchromane (20) 114
13.47 1,2-Di(chroman-4-yl)ethane 115
13.48 3-(But-3-en-1-yloxy)-2-iodopyridine 116
13.49 4-Methyl-3,4-dihydro-2*H*-pyrano[3,2-*b*]pyridine (21) 117
13.50 Ethyl (*E*)-5-(2-bromophenyl)pent-2-enoate 118
13.51 Ethyl 2-(2,3-dihydro-1*H*-inden-1-yl)acetate (22) 119
13.52 1-(6-Bromobenzo[*d*][1,3]dioxol-5-yl)-2,2-dimethylbut-3-en-1-ol 120
13.53 6,6,7-Trimethyl-6,7-dihydro-5*H*-indeno[5,6-*d*][1,3]dioxol-5-ol (23, dr ~ 3:2) 121
13.54 1-(6-Bromobenzo[*d*][1,3]dioxol-5-yl)-2,2-dimethylbut-3-en-1-one 122
13.55 6,6,7-Trimethyl-6,7-dihydro-5*H*-indeno[5,6-*d*][1,3]dioxol-5-one (24) 123
13.56 *tert*-Butyl (2-iodophenyl)carbamate 124
13.57 *tert*-Butyl cyclohex-2-en-1-yl(2-iodophenyl)carbamate 125
13.58 *tert*-Butyl 1,2,3,4,4a,9a-hexahydro-9*H*-carbazole-9-carboxylate (25) 126
13.59 *tert*-Butyl but-3-en-1-yl(2-iodophenyl)carbamate 128
13.60 *tert*-Butyl 4-methyl-3,4-dihydroquinoline-1(2*H*)-carboxylate (26) 129
13.61 *tert*-Butyl allyl(2-iodophenyl)carbamate 130
13.62 *tert*-Butyl 3-methylindoline-1-carboxylate (27) 131
13.63 2-Iodo-*N*-methylaniline 133
13.64 *N*-Allyl-2-iodo-*N*-methylaniline 134
13.65 1,3-Dimethylindoline (28) 135
13.66 Ethyl (*E*)-3-cyclopropylacrylate 136
13.67 (*E*)-3-Cyclopropylprop-2-en-1-ol 137
13.68 1-((3-Cyclopropylallyl)oxy)-2-iodobenzene (SI1, *E:Z* ~ 7:1) 138
13.69 3-(But-1-en-1-yl)-2,3-dihydrobenzofuran (SI2, *E:Z* ~ 3:1) 139
13.70 Tetrabutylammonium triiodide 140

1 General Experimental

All of the solvents and reagents were used as received from standard chemical suppliers unless otherwise stated. Tetraethylammonium tetrafluoroborate (Alfa Aesar, 99%) was recrystallized from hot methanol and dried at 60 °C in a vacuum oven (~10 mbar) for 24 h.

TLC was performed on aluminium-precoated plates coated with silica gel 60 with an F254 indicator; visualised under UV light (254 nm) and/or by staining with potassium permanganate or cerium ammonium molybdate (CAM). Flash column chromatography was performed with Sigma Aldrich 60 silica gel (40–63 micron).

Fourier-transform infrared (FT-IR) spectra are reported in wavenumbers (cm^{-1}) and were recorded using a diamond ATR accessory, as solids or neat liquids.

^{1}H NMR and ^{13}C NMR spectra were recorded in $CDCl_3$, DMSO-d_6 or CD_3CN solutions at 298 K (unless otherwise stated) at 400 MHz and 101 MHz, or 500 MHz and 126 MHz, respectively. Chemical shifts are reported in δ units using $CHCl_3$ (δ 7.27 ppm ^{1}H, δ 77.00 ppm ^{13}C), DMSO (δ 2.50 ppm ^{1}H, 39.52 ppm ^{13}C) and CH_3CN (δ 1.94 ppm ^{1}H) as an internal standard. Coupling constants (J) were recorded in Hz and are corrected. The following abbreviations for the multiplicity of the peaks are s (singlet), d (doublet), t (triplet), q (quartet), quint (quintet), sxt (sextet), sept (septet), oct (octet) br (broad), and m (multiplet). Melting points were obtained in an open capillary and are uncorrected. Electrospray resolution mass spectra were recorded on a H2Os ZMD quadrupole spectrometer. High resolution mass spectra were recorded on a Bruker Daltonics MaXis mass spectrometer equipped with a time of flight analyzer.

Yields of compounds **2, 3** and **4** and remaining starting material **1** were determined by gas chromatography using a Shimadzu GC 2014 equipped with an autosampler, FID detector and Agilent technologies HP5 column (length 30 m, I.D. 0.32 mm, film thickness 0.25 µm). The results were processed using GC Solution Lite software. Separations were carried out using He as carrier gas with a flow rate of 2.43 mL min^{-1} through the column. A split injection was conducted using a split ratio of 50:1. The injection and detector temperatures were maintained at 280 and 295 °C, respectively. The oven temperature was initially held at 70 ºC and then programmed to increase at 10 °C min^{-1} to 250 °C, where it was held for 2 min. The GC was calibrated using a range of solutions of known concentration of the starting material, the product and the side products.

Syntheses were carried out in an Ammonite 8 flow cell (Cambridge Reactor Design) with different anode and cathode materials as stated in the experimental part. Full details of the Ammonite 8 have been published elsewhere,[1] and the reactor is available commercially from Cambridge Reactor Design Ltd. (www.cambridgereactordesign.com). The cell current was controlled with a Rapid Electronics switching mode power supply (85-1903) or an Aim-TTi bench power supply (QL564). A peristaltic pump (Ismatec® REGLO Digital Ms-2/6) was used to flow the solutions through the electrochemical cell.

2 Experimental setup (Figures S1–S2)

Syntheses were carried out in an Ammonite 8 flow cell (Cambridge Reactor Design) with a glassy carbon anode and 316L stainless steel cathode unless otherwise stated.[1] This cell has a spiral electrolyte channel, 1 m in length and 2 mm in width and the interelectrode gap is 0.5 mm (Figure S1).

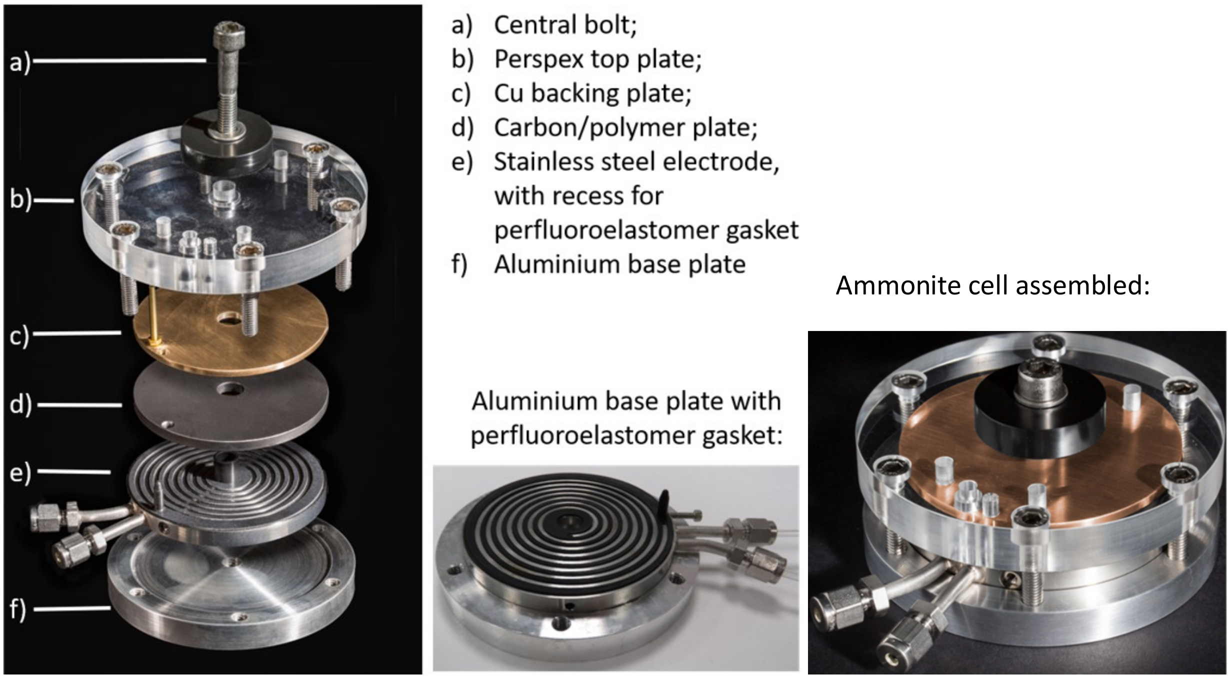

Figure S1: Pictures of the Ammonite 8 electrolysis cell.

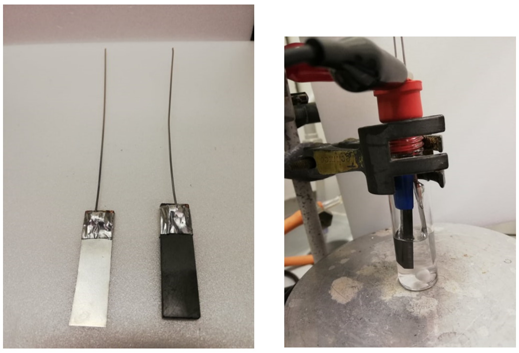

Figure S2: Pictures of the batch cell used used for comparison experiments. Left: Steel and carbon/PVDF electrodes; Right: Batch cell set up with the reaction solution.

3 Optimisation of the reaction conditions

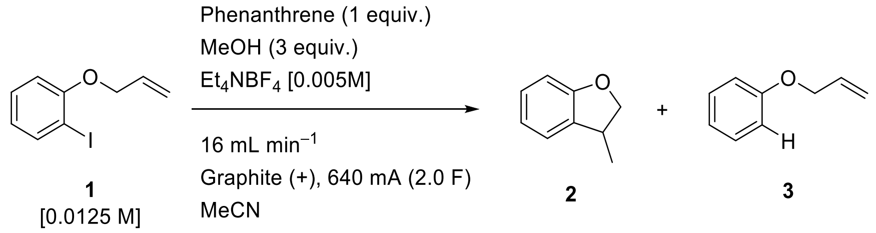

Table S1: Investigation of cathode material using graphite as anode.

Cathode	Yield (%)[a]		
	2	1	3
Pt	10	59	27
Ag	40	57	1
Leaded bronze	10	59	25
Ni	23	60	10
Glassy carbon	14	80	2
Stainless steel	35	53	4

[a] Yield estimated using ^{1}H NMR (DMT as internal standard)

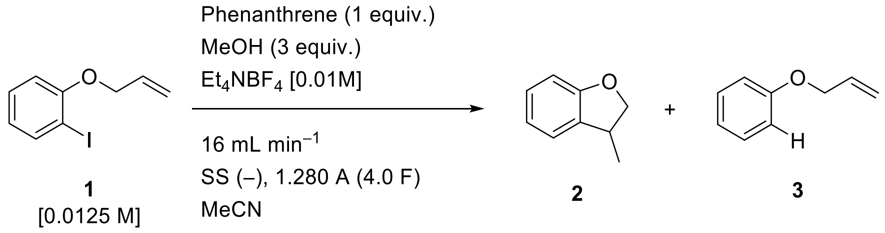

Table S2: Optimisation of anode material using Stainless Steel as cathode.

Anode	Yield (%)[a]		
	2	1	3
Graphite[b]	61	31	2
Pt	36	68	2
C:PVDF[b]	56	36	1
Glassy Carbon	57	36	2

[a] Yield estimated using ^{1}H NMR (DMT as internal standard); [b] carbon erosion observed

Decreasing the loading of mediator: optimisation

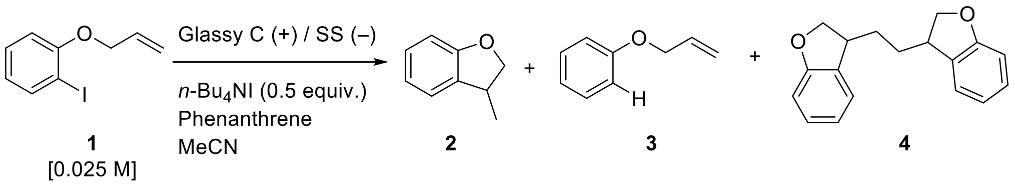

Table S3: Phenanthrene loading optimisation and study of the influence of the supporting electrolyte.

Entry	Med [equiv]	Flow rate [mL min^{-1}]	**I** [mA] (**Q** [F])	Yield (%)[a]			
				**1**	**2**	**3**	**4**
1	0			40	16	15	3
2	0.5	16.0	1280 (2.0)	19	49	-	7
3	1.0			22	72	-	7
4	1.0	0.50	40 (2.0)	39	51	5	3
5	1.0			17	79	1	5
6	0.50			18	78	1	5
7	0.25			19	71	-	10
8	0.10	2.0	160 (2.0)	19	70	3	8
9	0.05			18	75	4	5
10	0.01			20	55	15	6
11	0.0			23	25	48	–
12			200 (2.5)	8	75	2	8
**13**			**240 (3.0)**	**7**	**82**	**2**	**8**
14[b]			400 (2.5)	12	72	3	8
15[c]	0.05	2.0	200 (2.5)	16	53	16	5
16[d]			200 (2.5)	16	49	11	4
17[e]			240 (3.0)	3	80	10	6

[a] yield estimated using calibrated GC; [b] 0.05 M of **1**, brown deposit observed on the cathode after the reaction; [c] Et_4NBF_4 (0.0125 M) instead of *n*-Bu_4NI; [d] Et_4NBF_4 (0.0125 M) instead of *n*-Bu_4NI, MeOH (1 equiv.); [e] 0.25 mm interelectrode gap, instead of 0.5 mm.

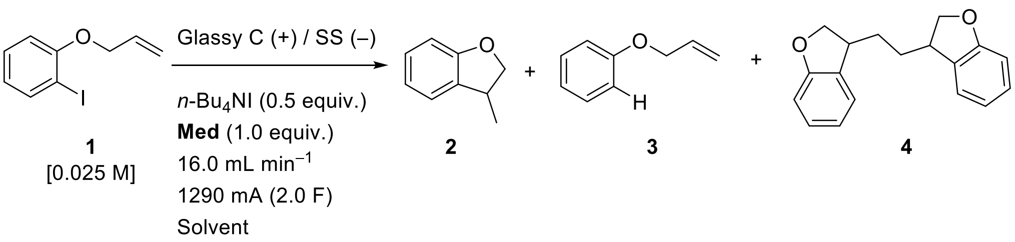

Table S4: Study of anhydrous solvents and degassing of the solution.

Entry	Solvent	Solution conditions	Yield (%)[a]			
			**1**	**2**	**3**	**4**
1	Anhydrous MeCN	Degassed	23	72	-	6
2	Bench-top MeCN	Non-degassed	24	68	-	10
3	Bench-top MeCN	Degassed	18	75	4	5

[a] yield estimated using calibrated GC

Mediator screening

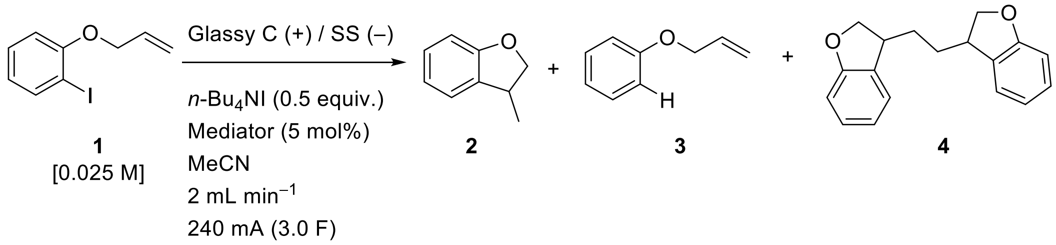

Table S5: Mediators screening

Entry	Med	Yield (%)[a]			
		**1**	**2**	**3**	**4**
1	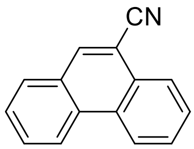	12	54	26	-
2	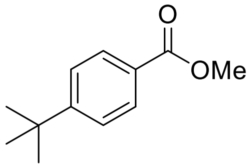	16	34	39	3
3	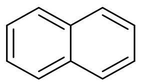	10	68	11	9
4	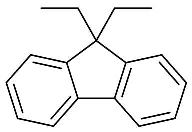	11	46	28	5
5	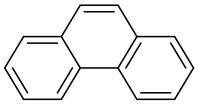	7	82[b]	4	7

[a] yield estimated using calibrated GC; [b] The reaction was run 5 times and the yield obtained was always between 81–83%

4 Radical clock experiment

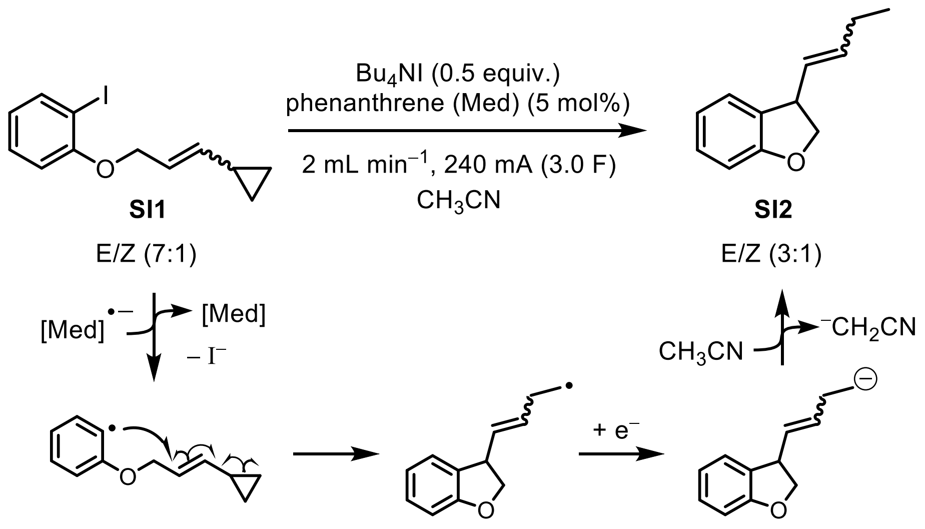

A solution of aryl halide (**SI1**, 0.150 g, 0.50 mmol), phenanthrene (4.5 mg, 0.025 mmol) and *n*Bu_4NI (93 mg, 0.25 mmol) in MeCN (20 mL) was degassed under nitrogen for 10 min. The solution was pumped through the Ammonite 8 reactor (glassy carbon anode, stainless steel cathode; internal volume = 1 mL) at a fixed flow rate of 2.0 mL min^{-1} and an applied current of 0.24 A (3.0 F). The reservoir with the starting material solution was kept under a flow of nitrogen while it was being pumped to the reactor. The reaction solution was collected, solvent was removed under reduced pressure and the crude mixture was purified by flash chromatography (hexane:toluene = 19:1) to afford 3-(but-1-en-1-yl)-2,3-dihydrobenzofuran (**SI2**, 0.045 g, 52%) as a colourless oil. Spectroscopic data are reported in Section 10 of the SI.

5 Electrolysis using Bu_4NI_3 in place of Bu_4NI

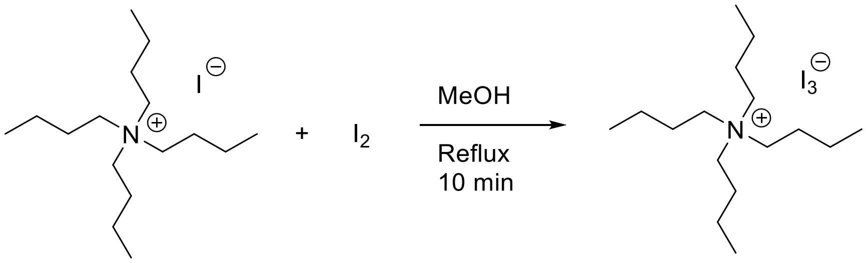

Procedure:[2] n-Bu_4NI (1.82 g, 5 mmol) and iodine (1.40 g, 5.5 mmol) were dissolved in MeOH (15 mL) and heated to 65 °C. After 10 min, the reaction mixture was cooled to rt and a black precipitate appeared. The mixture was cooled to 0 °C and the solid was filtered. The black solid was recrystallised from hot EtOH to give nBu_4NI_3 (2.65 g, 85% yield) as a black/dark purple solid. The spectroscopic and physical data were identical to the data measured for nBu_4NI_3 isolated from electrochemical experiments (see the Synthetic Procedures section in this document, compound **2**).

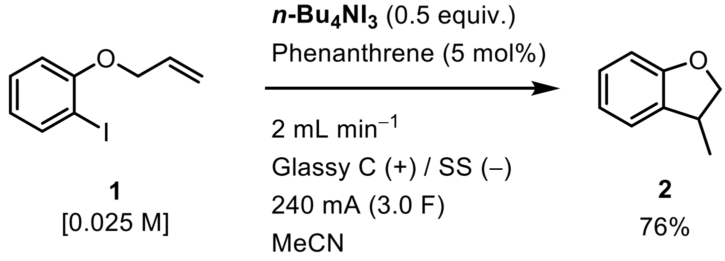

A solution of aryl halide (**1**, 0.130 g, 0.50 mmol), phenanthrene (4.5 mg, 0.025 mmol) and n-Bu_4NI_3 (156 mg, 0.25 mmol, prepared using the procedure described above) in MeCN (20 mL) was degassed under nitrogen for 10 min. The solution was pumped through the Ammonite 8 reactor (glassy carbon anode, stainless steel cathode; internal volume = 1 mL) at a fixed flow rate of 2.0 mL min^{-1} and an applied current of 0.24 A (3.0 F). The reservoir with the starting material solution was kept under a flow of nitrogen while it was being pumped to the reactor. The crude effluent solution was analysed by calibrated GC giving an estimated 76% yield of product **2**.

The optimum conditions presented in the main paper using n-Bu_4NI gave product **2** in 82% yield, infering that the presence of n-Bu_4NI_3 in the reaction mixture does not interfere significantly with the course of the reaction.

6 Deuterium incorporation studies

Experiments in CD_3CN and solvent mixtures of CD_3CN and THF were performed to confirm the source of the H/D in the product **2/2-d**. The experiments would also provide support for the proposed reaction pathway, and clarify whether cyclised radical **31** is reduced to a carbanion under the electrolysis conditions, subsequently undergoing protonation to afford **2**, or if alkyl radical **31** abstracts a hydrogen atom from solvent.

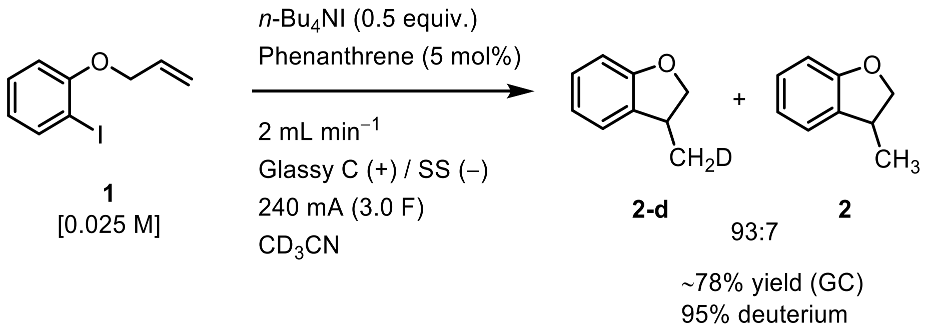

Procedure using CD_3CN as solvent: A solution of aryl iodide (**1**, 0.052 g, 0.20 mmol), phenanthrene (1.8 mg, 0.010 mmol) and n-Bu_4NI (37 mg, 0.10 mmol) in CD_3CN (8 mL) was degassed under nitrogen for 10 min. The solution was pumped through the Ammonite 8 reactor (glassy carbon anode, stainless steel cathode; internal volume = 1 mL) at a fixed flow rate of 2.0 mL min^{-1} and an applied current of 0.24 A (3.0 F). The reservoir with the starting material solution was kept under nitrogen while the solution was pumped to the reactor. The crude CD_3CN solution was directly analysed by ^{1}H NMR, giving a deuterium incorporation of 95% (**2-d**:**2** = 95:5, Figure S3).

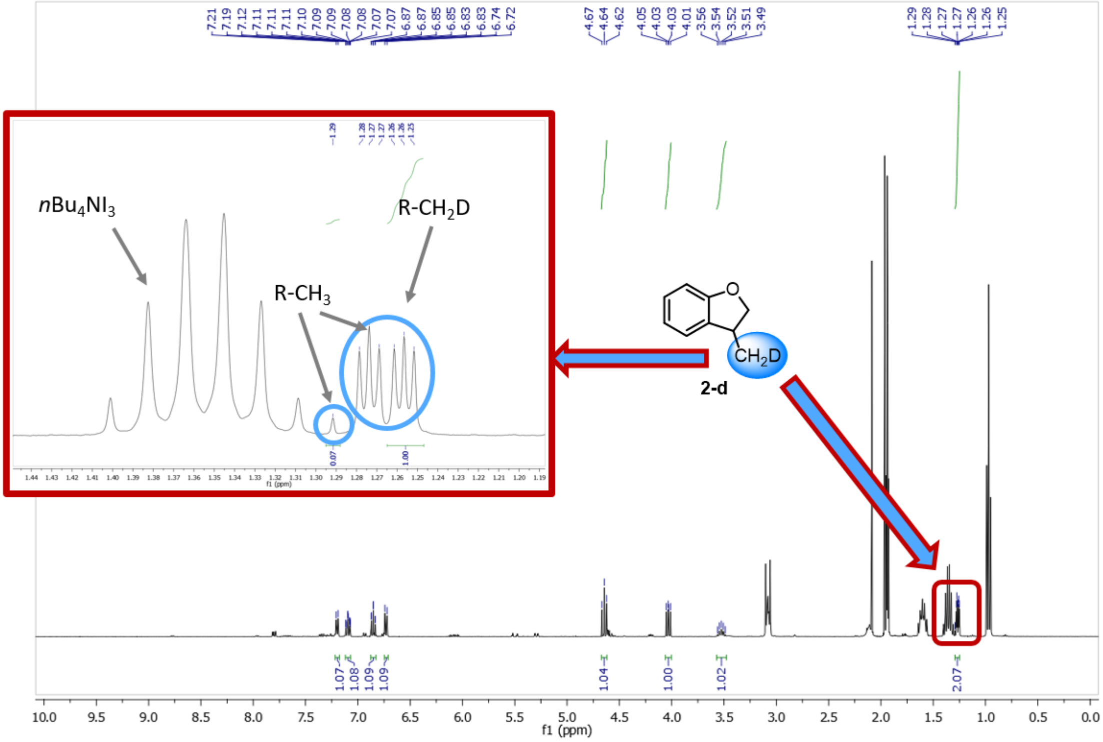

Figure S3: ^{1}H NMR of crude reaction in deuterated acetonitrile.

The experiment showed a high level of deuterium incorporation from acetonitrile. The reaction was carried out in CH_3CN in the presence of one equivalent of D_2O (1 equiv) or CH_3OD (2 equiv) without significant deuterium incorporation observed by ^1H NMR (not shown). The reaction did not proceed efficiently when attempted using larger amounts of CH_3OD or D_2O. Others have interpreted the results to indicate that H/D is introduced into the product by atom abstraction from CH_3CN/CD_3CN.[3] However, a highly basic alkyl anion arising from reduction of **31** (see section 7.2 in SI) would readily deprotonate CH_3CN, which is present in vast excess compared to MeOD or D_2O.

To provide further insight, the reaction was carried out in the presence of a large quantity of an H-atom donor solvent (THF), with a reduced amount of CD_3CN; reaction performed under the same conditions but in a mixture of THF/CD_3CN (7/1). The crude solution was analysed by ^1H NMR, giving a deuterium incorporation of 84% (**2-d**:**2** = 84:16) (Figure S4) from CD_3CN.

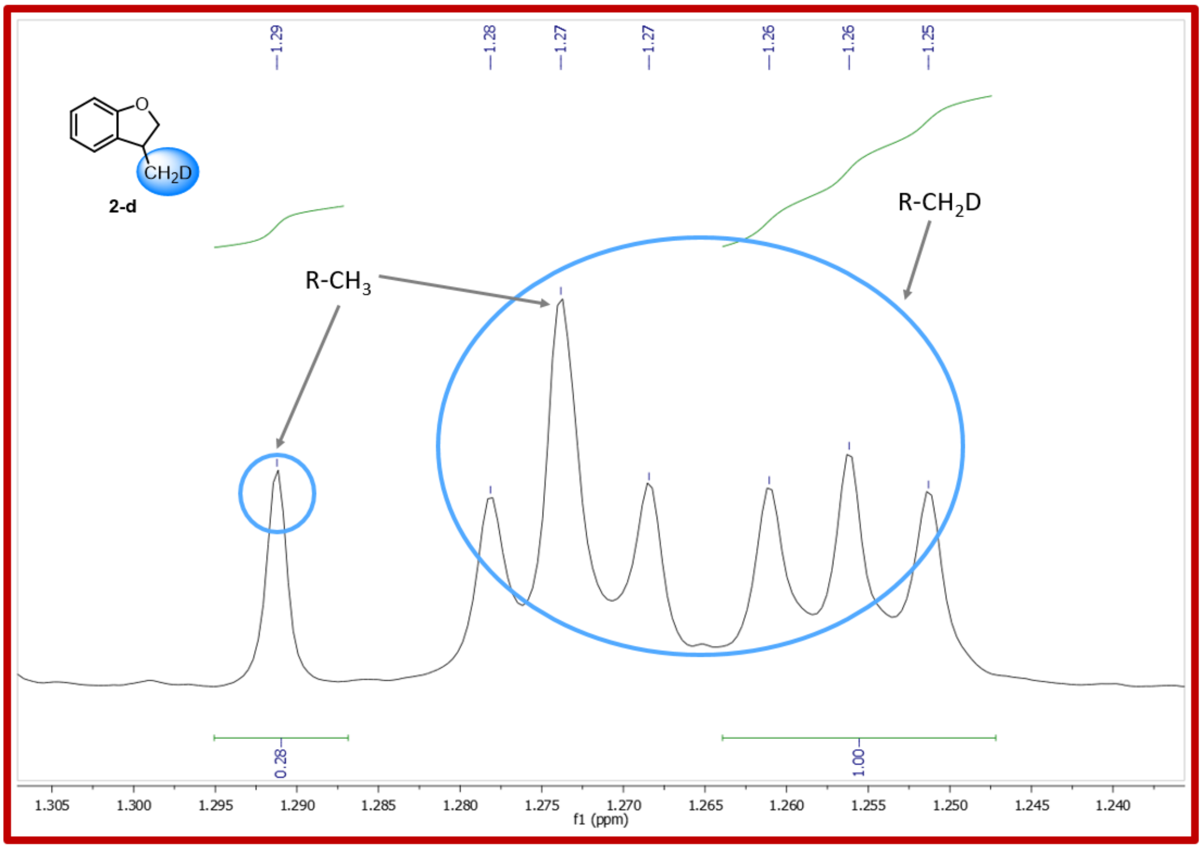

Figure S4: Expansion of crude ^1H NMR of reaction in THF/CD_3CN (7/1), showing ratio of deuterated and non-deuterated cyclised products (**2-d**/**2** respectively).

The observation of high levels of deuterium incorporation in THF/CD_3CN (7:1) supports the intermediacy of an alkyl carbanion and protonation (D^+ in the experiment) as the major pathway. Small amounts of the "unlabelled product" **2** likely arise by atom abstraction, presumably from THF, as a minor pathway. Deprotonation of the supporting electrolyte could also lead to the unlabelled product, but this seems less likely as CD_3CN is in large excess compared to Bu_4N^+, and this was not observed in the experiment using CD_3CN as the sole solvent.

7 Mechanistic studies: cyclic voltammetry and simulations

7.1 Experimental cyclic voltammograms

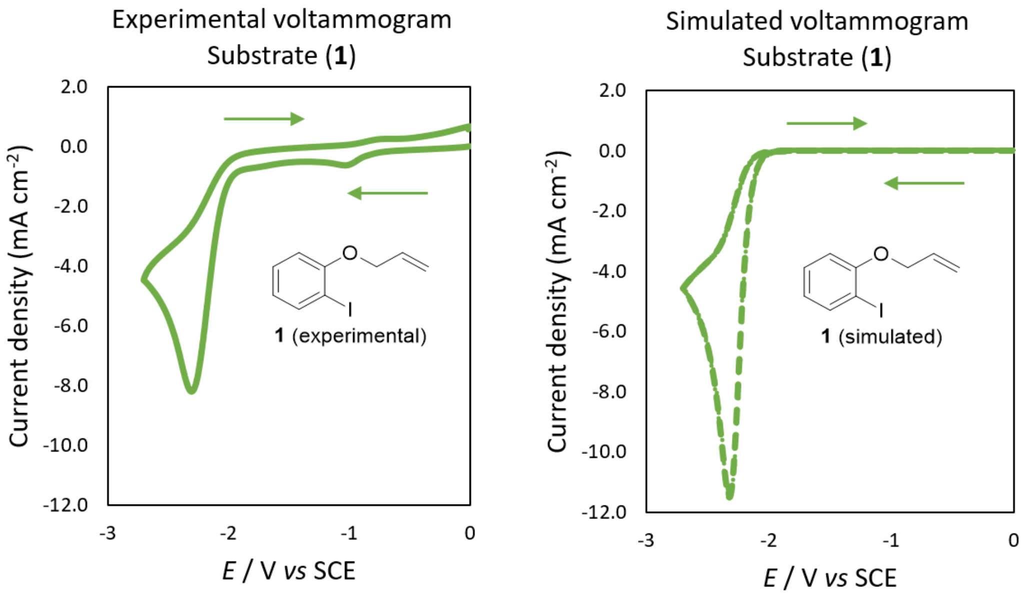

Figure S5: Left: Cyclic voltammogram (1st cycle) recorded using a 3 electrode configuration with SCE as reference electrode at 900 mV s^{-1}, with a glassy carbon (3 mm Ø) working electrode and a Pt counter electrode in 5 mM 1-(allyloxy)-2-iodobenzene (**1**), 100 mM Et_4NBF_4, degassed dry MeCN. The scan started at 0 V and the arrows indicate the scan directions. **Right**: Simulated cyclic voltammogram with the same chemical concentrations. Conditions shown in Table S6.

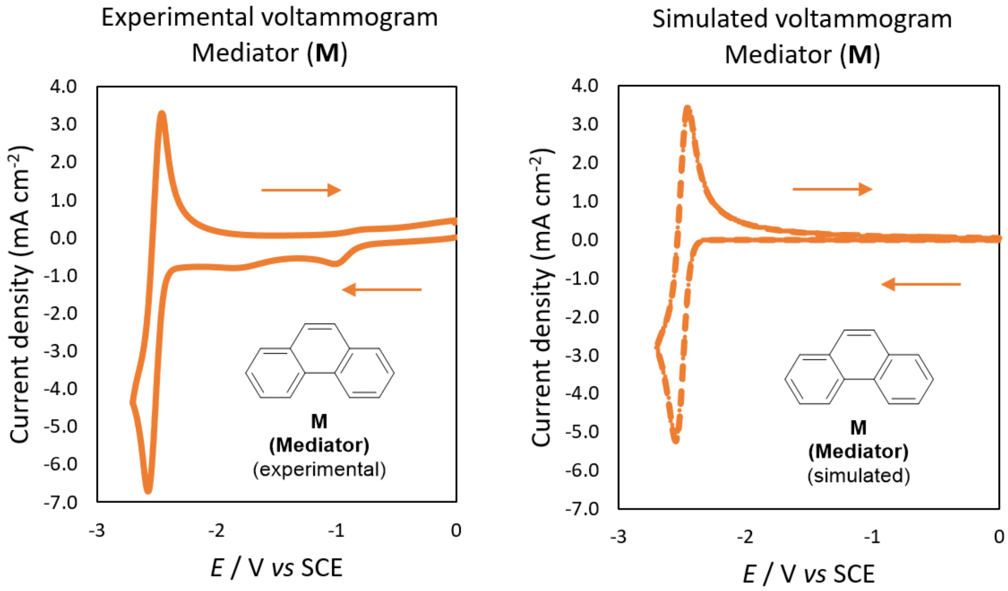

Figure S6: **Left**: Cyclic voltammogram (1st cycle) recorded using a 3 electrode configuration with SCE as reference electrode at 900 mV s^{-1}, with a glassy carbon (3 mm Ø) working electrode and a Pt counter electrode in 5 mM phenanthrene (**M**), 100 mM Et_4NBF_4, degassed dry MeCN. The scan started at 0 V and the arrows indicate the scan directions. **Right**: Simulated cyclic voltammogram with the same chemical concentrations. Conditions shown in Table S6.

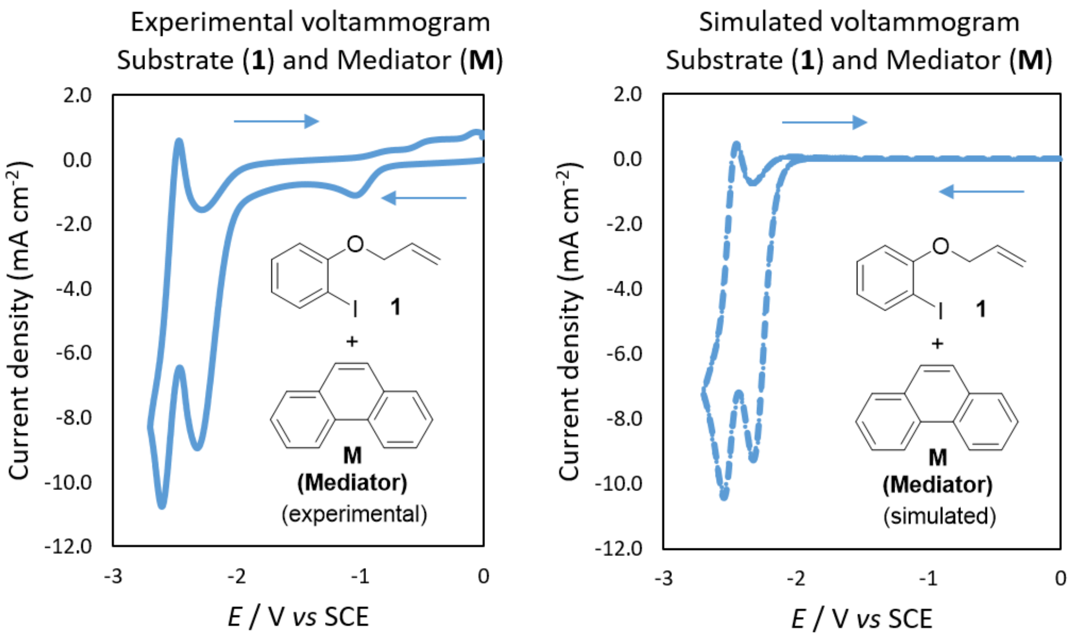

Figure S7: **Left**: Cyclic voltammogram (1st cycle) recorded using a 3 electrode configuration with SCE as reference electrode at 1000 mV s^{-1}, with a glassy carbon (3 mm Ø) working electrode and a Pt counter electrode in 5 mM 1-(allyloxy)-2-iodobenzene (**1**), 5 mM phenanthrene (**M**), 100 mM Et_4NBF_4, degassed dry MeCN. The scan started at 0 V and the arrows indicate the scan directions. **Right**: Simulated cyclic voltammogram (900 mV s^{-1}) with the same chemical concentrations. Conditions shown in Table S6.

It should be stressed that the voltammetry (in an unstirred cell), using a potential sweep towards negative values, is not representative of the situation in the (flow) reactor where the electrode sits at a potential negative to the mediator (and the substrate), since we work under galvanostatic conditions (i.e. constant current). The CV of the mixture (substrate **1** plus phenanthrene (**M**), Figure S7) shows the reduction wave for the substrate first, and then as the potential becomes more negative, the reduction wave for the mediator. This is as expected, and supported by the simulated voltammogram. There is no observable catalytic enhancement of the mediator current in the voltammetry because under the conditions used here, the homogeneous reaction between the radical anion of the mediator and the substrate has a negligable impact on the mediator flux. If a mediator that reduces at a potential positive to the substrate were used, the voltammogram would show the classical catalytic enhancement of the mediator current at the mediator reducing potential. We wish to stress that this is not the case in our work because the substrate concentration near the electrode is very low by the time the potential scan reaches the mediator reducing potential. At that point, the electrode reduces the mediator and the mediator radical anion diffusing away from the electrode encounters the substrate which is diffusing from the bulk. This is the cause of the development of the detached reaction layer.

All the experimental voltammograms present a redox wave at ~ –1.0 V, which is also observed in the background electrolyte (see Figure S8). This wave is attributed to the reduction of trace oxygen.

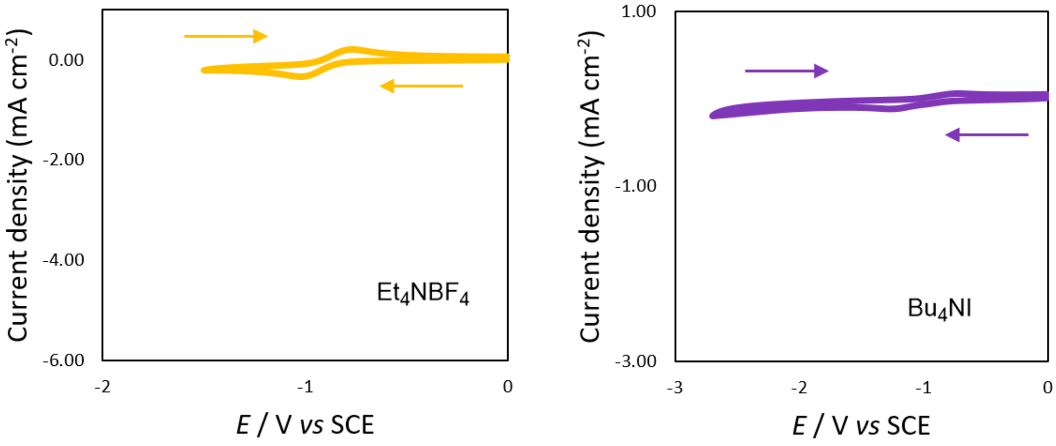

Figure S8: Cyclic voltammograms recorded using a 3 electrode configuration with SCE as reference electrode at 100 mV s^{-1}, with a glassy carbon (3 mm Ø) working electrode and a Pt counter electrode. **Left**: in 100 mM Et_4NBF_4, degassed dry MeCN. **Right**: in 100 mM Bu_4NI, degassed dry MeCN. The scan started at 0 V and the arrows indicate the scan directions.

Cyclic voltammograms were also recorded with a stainless steel working electrode instead of glassy carbon, using a 3 electrode configuration with SCE as reference electrode at 900 mV s^{-1}, with a stainless steel wire (AISI 304L) (Ø = 0.5 mm, L = 4 mm) working electrode and a Pt counter electrode in 5 mM 1-(allyloxy)-2-iodobenzene (**1**) or 5 mM phenanthrene (**M**), 100 mM Et_4NBF_4, degassed dry MeCN. On steel the substrate **1** was found to reduce at a potential *circa* 280 mV more positive than the mediator (Substrate **1**: $E^{p/2}$ = –2.27 V vs SCE; Mediator **M**: $E^{p/2}$ = –2.55 V vs SCE).

7.2 Simulations

The concentration profiles shown in Figure 3 of the main paper were simulated with DigiElch v.8 (ElchSoft) using the reaction mechanism shown in Table S6 and the conditions listed below:

- All the heterogeneous e^- transfer steps were initially assumed to be very fast (standard rate constant for e^- transfer, k_s, equal to 10^4 cm s^{-1}), to follow the Butler-Volmer (BV) kinetic formalism, and have a transfer coefficient, α, equal to 0.5. The standard potentials were estimated from the experimental voltammograms. When simulating the experimental voltammograms the electron-transfer rate constants and E^0 values were adjusted to improve the fit of the simulated and experimental data.
- The homogeneous steps were assumed to be very fast. Where possible, rate constants were obtained from the literature. The rate constant for step (2) was estimated from table 1 in ref 4 that quotes the original article ref 5.[4],[5] The rate constant for step (6) (k=8×10^9 s^{-1}) was taken from ref 6 which quoted the original article ref 7.[6],[7]
- The protonation of aryl anion **30** to **3** was assumed to be sufficiently fast for **29** to convert directly to **3** at the electrode surface.
- Step (8), the protonation of alkyl anion to afford **2** (step (8)) was assumed to sufficiently fast for **31** to convert directly to **2** and **M** upon reaction with $\mathbf{M}^{\bullet-}$ in step (7).
- The diffusion coefficients of **1** and **M** were determined by recording voltammograms over a range of sweep rates and analysing the sweep rate dependence of the background corrected reduction peaks at ~–2.3 V vs SCE for **1** and ~–2.56 V vs SCE for **M**. The following values were obtained and used in the simulations: D_1=3.3×10^{-5} cm^2 s^{-1} and D_M=2.0×10^{-5} cm^2 s^{-1}.
- The diffusion coefficients of $\mathbf{1}^{\bullet-}$, **2**, **3**, **29**, and **31** were assumed to be equal to that of **1**.
- The diffusion coefficient of $\mathbf{M}^{\bullet-}$ was assumed to be equal to that of **M**.
- The electrode geometry was set to planar and the diffusion to semi-infinite 1D.
- The simulation parameters were kept to their default values.
- The simulations were carried out with the adaptive grid simulator.
- The concentration profiles were taken at –2.7 V vs SCE after simulating a voltammetric sweep from 0 to –2.7 V vs SCE.

Table S6: Reaction mechanism used to simulate the concentration profiles and voltammograms. BV stands for Butler-Volmer, α is the transfer coefficient, k_s the standard rate constant for e^- transfer, and k is a homogeneous rate constant. The E^0 values were estimated from the experimental CVs and refined using simulations.

Step	Reaction	Parameters
1	**1** + e^- → **1**$^{\bullet-}$	E^0=–2.2 V vs SCE, BV kinetics, α=0.5, k_s=5x10^{-3} cm s^{-1}
2	**1**$^{\bullet-}$ → **29**	k=10^{10} s^{-1}
3[a]	**29** + e^- → **3**	E^0=–1 V vs SCE, BV kinetics, α=0.5, k_s=10^4 cm s^{-1}
4	**M** + e^- → **M**$^{\bullet-}$	E^0=–2.5 V vs SCE, BV kinetics, α=0.5, k_s=3x10^{-2} cm s^{-1}
5	**1** + **M**$^{\bullet-}$ → **1**$^{\bullet-}$ + **M**	k=10^3 mol^{-1} dm^3 s^{-1}
6	**29** → **31**	k=8×10^9 s^{-1}
7[a]	**31** + **M**$^{\bullet-}$ → **2** + **M**	k=10^3 mol^{-1} dm^3 s^{-1}

[a] Aryl radical **29** and alkyl radical **31** were considered to convert directly to products **3** and **2**, respectively. Protonation steps were assumed to be very fast and were not included as separate steps in the simulation.

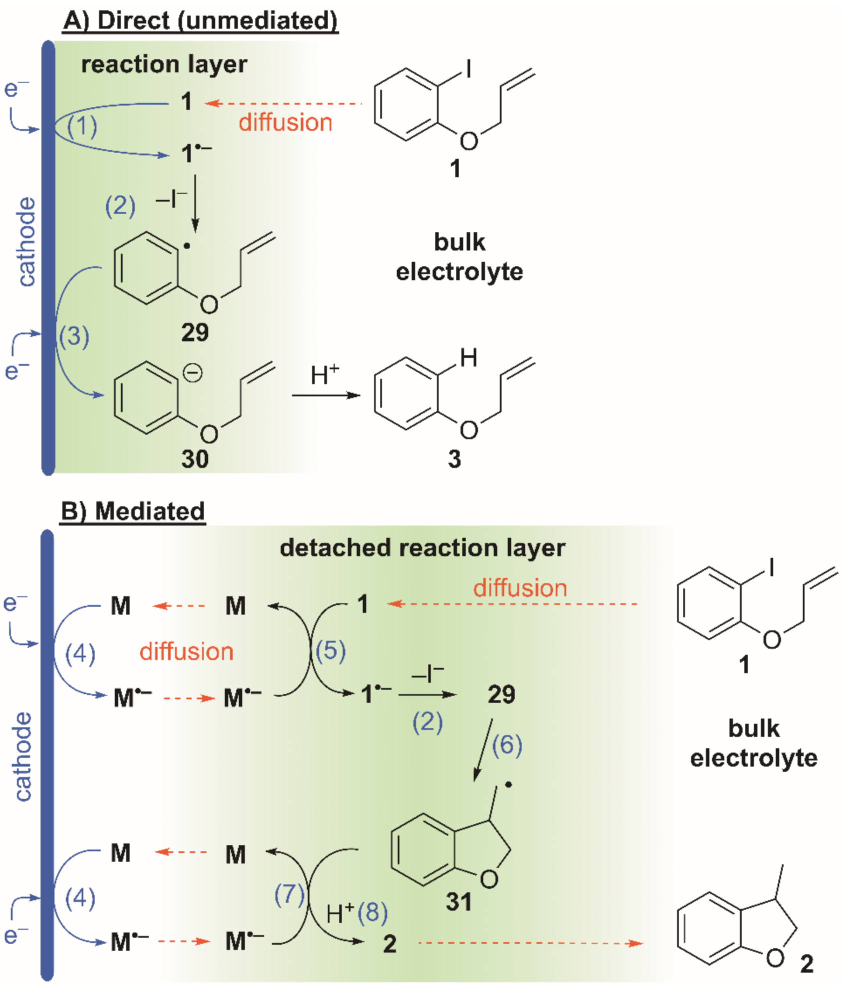

Figure 1 from Main Paper. Electrochemical and Chemical Reactions for unmediated (A) and mediated (B) electroreduction of aryl iodide **1**.

7.3 Simulated concentration profiles

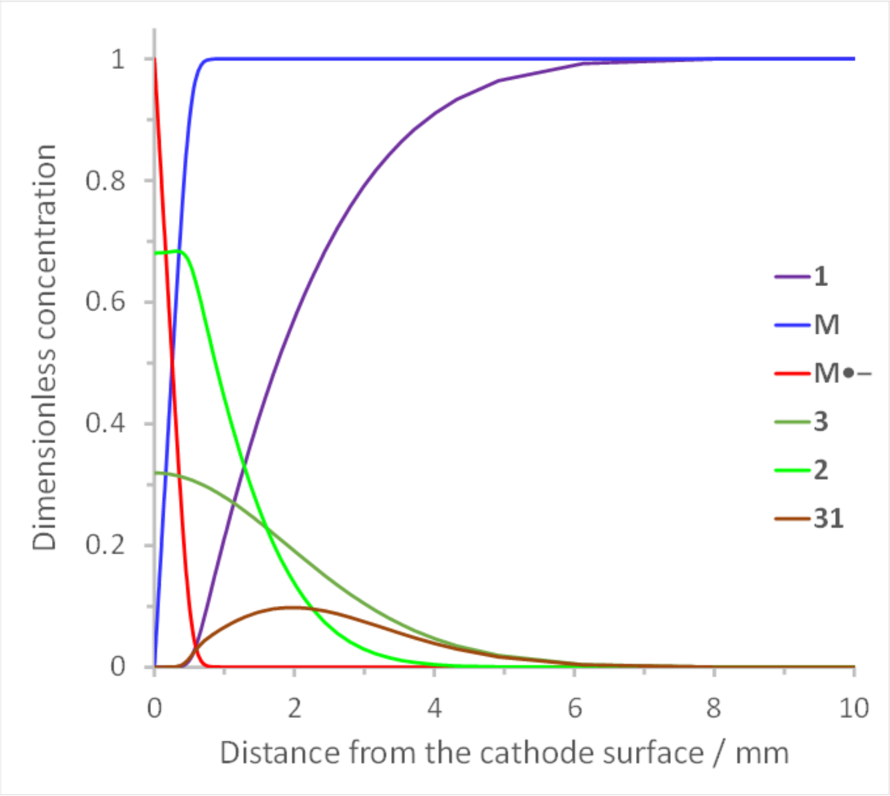

Figure S9: Same as Figure 2 in the main paper but showing all the species in the mechanism.

8 Flow Setup

8.1 Calculation for the current needed in the flow cell (***I_{theo}***) and charge applied (F)

The *theoretical current* (I_{theo}) needed for full conversion in a flow electrochemical process can be calculated using the following equation:

Faraday's law applied to flow conditions: $I_{theo} = n\ F\ C\ Q_v$ (eq 1)

- I_{theo} = Theoretical current (A)
- n = number of electrons involved in the electrochemical process
- F = Faraday's constant (96 485 s A mol^{-1})
- C = Concentration (mol mL^{-1})
- Q_v = Flow rate (mL s^{-1})

This assumes that the reaction is under conditions of mass transfer control, and is a selective process. The *current efficiency (CE)* of the electrochemical process can be calculated using the following equation:

Current efficiency: $CE = \frac{I_{theo}}{I_{cell}} \times yield\%$ (eq 2)

8.2 General method A: stoichiometric phenanthrene (1 equiv.)

The reductive cyclisation herein presented is assumed to be a 2-electron reduction process (n = 2, see Figure 2 of the main paper). The optimised conditions for general method A are the following:

Concentration = 0.025 mol L^{-1} = 0.000025 mol mL^{-1}

Flow rate (Q_v) = 16.0 mL min^{-1} = 0.267 mL s^{-1}

Therefore, with the equation shown above, the theoretical current (I_{theo}) required for full conversion under the conditions (16.0 mL min^{-1}, 0.025 M) can be calculated:

I_{theo} = 1.28 A

Where the reaction solution was passed through the reactor twice, therefore the total applied cell current (I_{cell}) is 2 x 1.28 A, which means that a 2.0 fold excess of charge is applied. This is the stoichiometry of current required, and is represented by "*a*":

$I_{cell} = x\ F\ C\ Q_v$ Where: $x = n\ a$ (eq 3)

$$x = \frac{2x1.28}{96485 \times 0.000025 \times 0.267} = 4.0$$

"4.0 F is the charge applied to the electrochemical reaction under the conditions of general method A"

Yield of **2** = 72%

$$CE = \frac{1.28}{2x1.28} \times 72\% = \mathbf{36\%}$$

8.3 General method B: catalytic phenanthrene (0.05 equiv.)

The reductive cyclisation herein presented is assumed to be a 2-electron reduction process (n = 2). The optimised conditions for general method B are the following:

Concentration = 0.025 mol L^{-1} = 0.000025 mol mL^{-1}

Flow rate = 2.0 mL min^{-1} = 0.0333 mL s^{-1}

Therefore, with the equation shown above (eq 1), the theoretical current (I_{theo}) for this transformation can be calculated:

I_{theo} = 0.160 A

But the applied cell current (I_{cell}) is 0.240 A, which means that a 1.5 fold excess of charge is used. This is the stoichiometry of current required, and is represented by "*a*" (see eq 3 above):

$$x = \frac{0.240}{0.000025 \times 0.0333 \times 96485} = 3.0$$

"3.0 F is the charge applied to the electrochemical reaction under the conditions of general method B"

Yield of **2** = 82%

$$\mathrm{CE} = \frac{0.160}{0.240} \times 82\% = \mathbf{55\%}$$

9 Batch Setup

9.1 Calculation of the current needed in the batch cell (I_{theo}) and charge applied (F)

The *theoretical electrolysis time* (t_{theo}) needed in a batch electrochemical process can be calculated using the following equation:

Faraday's law applied to batch conditions: $$t_{theo} = \frac{n\,mF}{I} \qquad \text{(eq 4)}$$

- t_{theo} = time of electrolysis (s)
- I = Current applied (A)
- n = number of electrons involved in the electrochemical process
- m = moles of substrate to be electrolysed (mol)
- F = Faraday's constant (96 485 s A mol^{-1})

The *current efficiency (CE)* of the electrochemical process for a batch-type reaction can be calculated using:

Current efficiency: $$CE = \frac{t_{theo}}{t_{real}} \times \text{yield\%} \qquad \text{(eq 5)}$$

The reductive cyclisation herein presented is a 2-electron reduction process (n = 2).

- *Surface area:* The batch electrode was used with a surface area of 1.65 cm^2 (1.2 (w) x 1.4 (h) cm).
- *Current density:* The cell averaged current density used was the same as for the reaction in the Ammonite 8: 12 mA/cm^2. Therefore:
- *Current applied (I):* 12 mA/cm^2 x 1.65 cm^2 = 20 mA.
- *Moles of substrate (n):* 0.2 mmol (in 8 mL)

Therefore, with the equation shown above (eq 4), the electrolysis time (t_{theo}) for this transformation can be calculated:

t_{theo} = 1930 s (32 min)

9.2 Reaction with catalytic phenanthrene (0.05 equiv.) in a batch cell

The reaction required 96 min (5760 s) to achieve > 94% conversion, using a 3 fold excess of charge. This is the stoichiometry of current needed, and is represented by "*a*":

$$t = \frac{x\,m\,F}{I} \qquad \text{Where:} \quad x = n\,a \qquad \text{(eq 6)}$$

$$x = \frac{0.020 \times 5760}{0.0002 \times 96485} = 6.0$$

"6.0 F is the charge applied to the batch electrochemical reaction"

GC Yield of **2** = 64%

$$CE = \frac{1930}{5760} \times 64\% = \mathbf{21\%}$$

9.3 Reaction with stoichiometric phenanthrene (1 equiv.) in a batch cell

The reaction required 80 min (4800 s) to achieve > 94% conversion, using 2.5 fold excess of charge. This is the stoichiometry of current needed, and is represented by "*a*":

$$t = \frac{x\,m\,F}{I} \qquad \text{Where:} \quad x = n\,a \qquad \text{(eq 6)}$$

$$\text{x} = \frac{0.020 \times 4800}{0.0002 \times 96485} = 5.0$$

"5.0 F is the charge applied to the batch electrochemical reaction in batch"

GC Yield of **2** = 73%

$$\text{CE} = \frac{1930}{4800} \times 73\% = \mathbf{29\%}$$

10 Reductive radical cyclisation in batch

10.1 Experimental procedure for the catalytic phenanthrene method:

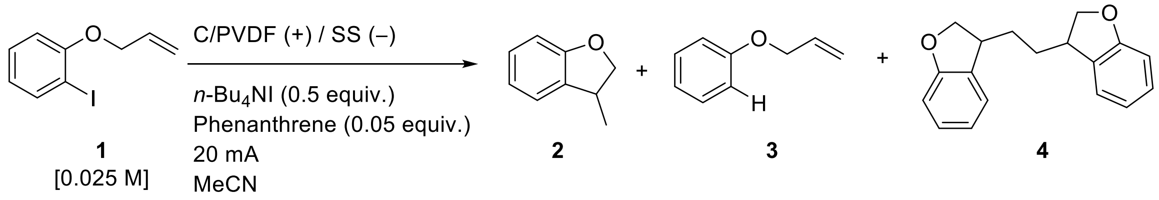

A stock solution of phenanthrene (**M**, 11.1 mg, 0.062 mmol) in MeCN (50 mL) was prepared. 1-(Allyloxy)-2-iodobenzene (**1**, 52 mg, 0.2 mmol) and *n*-Bu_4NI (37 mg, 0.5 equiv., 0.1 mmol) were dissolved in 8 mL of the stock solution of phenanthrene in MeCN (1.8 mg, 0.05 equiv., 0.01 mmol) in the vial used as the batch cell. A C/PVDF anode and a stainless steel cathode (12 mm wide) were submerged in the solution, having a working surface of 1.65 cm^2. The solution was stirred under a flow of nitrogen and a constant current of 20 mA was applied. An aliquot was analyzed by GC every 16 min (1.0 F) until 6.0 F (96 min) of charge were passed. The amount of cyclised product **2** was estimated to be 64% (calibrated GC) after 6.0 F of charge were passed. The amount of dehalogenated product **3** was estimated to be 23% (calibrated GC).

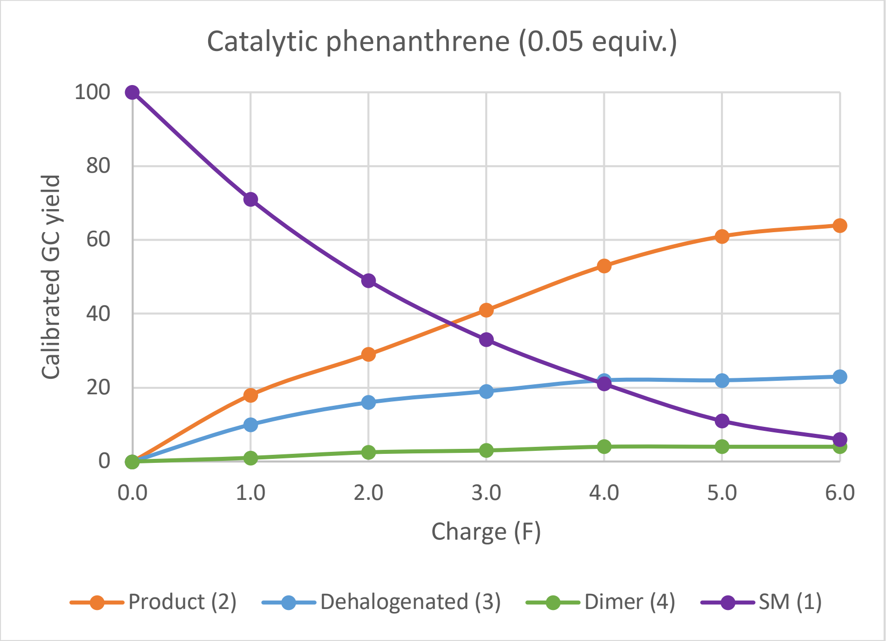

10.2 Experimental procedure using stoichiometric phenanthrene in a batch cell:

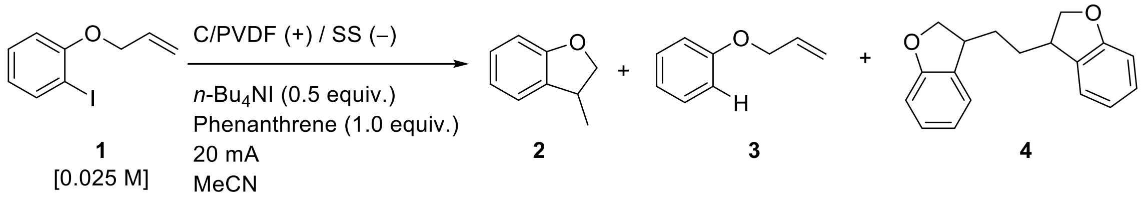

1-(Allyloxy)-2-iodobenzene (**1**, 52 mg, 0.2 mmol), *n*-Bu_4NI (37 mg, 0.5 equiv., 0.1 mmol) and phenanthrene (**M**, 36 mg, 1.0 equiv., 0.2 mmol) were dissolved MeCN (8 mL) in the vial used as the

batch cell. A C/PVDF anode and a stainless steel cathode (12 mm wide) were submerged in the solution, having a working surface of 1.65 cm^2. The solution was stirred under a flow of nitrogen and a constant current of 20 mA was applied. An aliquot was analyzed by GC every 16 min (1.0 F) until 6.0 F (96 min) of charge were passed. The amount of cyclised product **2** was estimated to be 74% (calibrated GC) after 6.0 F of charge were passed. The amount of dehalogenated product **3** was estimated to be 3% (calibrated GC).

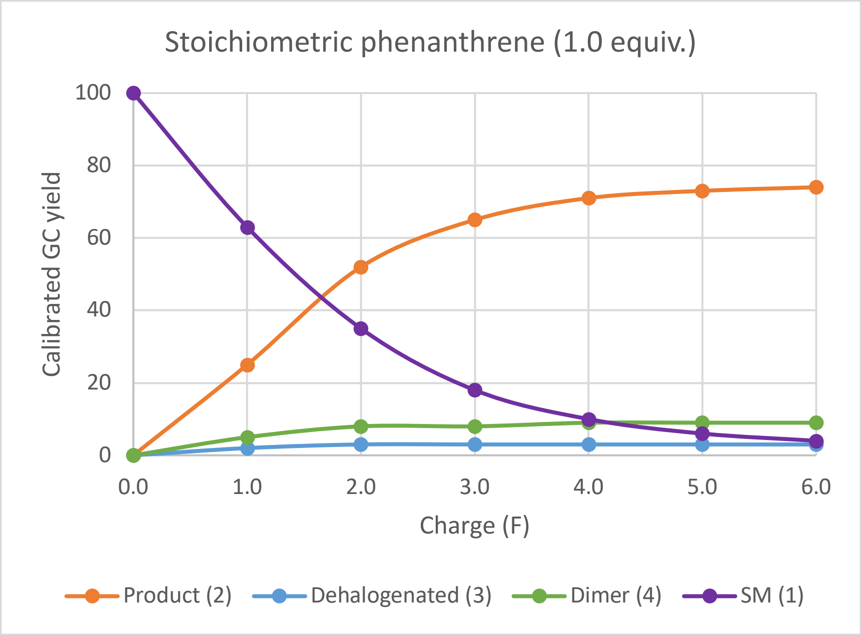

The results of the reactions run in batch showed greater variability with regards to the mediator loading, giving lower yield and selectivity for **2** using 0.05 equiv. of the mediator. When 0.05 equiv. of phenanthrene was used, the reaction needed a charge of 5.0 F to achieve 60% of the product **2**, while with 1 equiv. of phenanthrene a yield of 60% could be obtained with less than 3.0 F of charge. A significantly increased amount (23%) of the dehalogenated product **3** was observed using the low phenanthrene loading, while with the stoichiometric method only 3% of the dehalogenated compound was observed.

Using 0.05 equiv. of phenanthrene, cyclisation in the batch cell showed a lower selectivity for the cyclised product **2** to the dehalogenated (hydrogenolysis) product **3** (~3:1) compared to the flow cell (~40:1). The reasons for this are complex, and likely to be related to the effect of the different mass transport regimes in the two cells, and the effect upon the reaction layer. Mass transfer in the flow cell is more efficient than in the batch cell used in this study, as expected.

11 Synthetic procedures

11.1 General Method A: Reductive cyclisation with stoichiometric phenanthrene

A solution of aryl halide (1.0 equiv.), phenanthrene (**M**, 1.0 equiv.) and *n*Bu_4NI (0.5 equiv.) in MeCN (0.025 M) was degassed under nitrogen or argon for 20 min. The solution was pumped through the Ammonite 8 reactor (glassy carbon anode, stainless steel cathode; internal volume = 1 mL) at a fixed flow rate of 16 mL min^{-1} with an applied current of 1.28 A (2.0 F). Both the reservoir with the starting material solution and the collecting reservoir were kept under a flow of nitrogen or argon while it was being pumped to the reactor. The degassed crude solution was submitted to a second pass through the Ammonite 8 reactor under the same conditions (16 mL min^{-1}, 1.28 A (2.0 F)), resulting in a total applied charged of 4.0 F. The solvent was removed under reduced pressure and the crude mixture was purified by column chromatography.

11.2 General Method B: Reductive cyclisation with catalytic phenanthrene

A solution of aryl halide (1.0 equiv.), phenanthrene (**M**, 0.05 equiv.) and *n*Bu_4NI (0.5 equiv.) in MeCN (0.025 M) was degassed under nitrogen or argon for 20 min. The solution was pumped through the Ammonite 8 reactor (glassy carbon anode, stainless steel cathode; internal volume = 1 mL) at a fixed flow rate of 2.0 mL min^{-1} and an applied current of 0.24 A (3.0 F). The reservoir with the starting material solution was kept under a flow of nitrogen or argon while it was being pumped to the reactor. The solvent was removed under removed pressure and the crude mixture was purified by column chromatography.

11.3 General Method C: Preparation of aryl ethers[8]

The corresponding alkyl bromide (1.1 – 1.5 equiv.) was added to a mixture of the corresponding aryl alcohol (1.0 equiv.) and potassium carbonate (1.2 equiv.) in acetone to give an initial concentration of 0.20 M (with respect to ArOH). The resulting solution was heated under reflux for 16 h. After cooling to rt the solid was filtered off and the solvent evaporated under reduced pressure. The crude mixture was filtered through a short plug of silica using hexane as eluent to afford the aryl ether.

1-(Allyloxy)-2-iodobenzene (1)

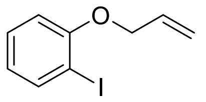

1-(Allyloxy)-2-iodobenzene (**1**) was prepared by **General Method C** from 2-iodophenol (3.30 g, 15.0 mmol) and allyl bromide (1.95 mL, 22.5 mmol). The crude mixture was filtered through a short plug of silica using hexane as eluent to afford 1-(allyloxy)-2-iodobenzene (**1**, 3.89 g, quantitative) as a colourless oil.

Spectroscopic data are consistent with those reported.[9]

^{1}H NMR (400 MHz, $CDCl_3$) δ =7.79 (dd, *J* = 7.8, 1.6 Hz, 1H), 7.29 (ddd, *J* = 8.2, 7.5, 1.6 Hz, 1H), 6.82 (dd, *J* = 8.2, 1.3 Hz, 1H), 6.73 (td, *J* = 7.6, 1.3 Hz, 1H), 6.08 (ddt, *J* = 17.3, 10.6, 4.8 Hz, 1H), 5.54 (dq, *J* = 17.3, 1.7 Hz, 1H), 5.32 (dq, *J* = 10.6, 1.7 Hz, 1H), 4.61 (dt, *J* = 4.8, 1.7 Hz, 2H) ppm.

^{13}C NMR (101 MHz, $CDCl_3$) δ = 157.1, 139.5, 132.6, 129.3, 122.6, 117.6, 112.5, 86.7, 69.6 ppm.

LRMS (EI$^+$) *m/z* (relative intensity): 260.2 (100%) $[M]^{+\bullet}$.

1-(Allyloxy)-2-bromobenzene

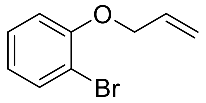

1-(Allyloxy)-2-bromobenzene was prepared by **General Method C** from 2-bromophenol (3.46 g, 20.0 mmol) and allyl bromide (2.60 mL, 30.0 mmol). The crude mixture was filtered through a short plug of silica using hexane as eluent to afford 1-(allyloxy)-2-bromobenzene (4.20 g, quantitative) as a colourless oil.

Spectroscopic data are consistent with those reported.[10,11]

^{1}H NMR (400 MHz, $CDCl_3$) δ = 7.56 (dd, *J* = 7.8, 1.6 Hz, 1H), 7.26 (ddd, *J* = 8.3, 7.4, 1.6 Hz, 1H), 6.91 (dd, *J* = 8.3, 1.4 Hz, 1H), 6.85 (ddd, *J* = 7.8, 7.4, 1.4 Hz, 1H), 6.08 (ddt, *J* = 17.3, 10.6, 5.0 Hz, 1H), 5.50 (dq, *J* = 17.3, 1.7 Hz, 1H), 5.33 (dq, *J* = 10.6, 1.7 Hz, 1H), 4.63 (dt, *J* = 5.0, 1.6 Hz, 2H) ppm.

^{13}C NMR (101 MHz, $CDCl_3$) δ = 154.9, 133.4, 132.6, 128.3, 122.0, 117.7, 113.6, 112.3, 69.6 ppm.

LRMS (EI$^+$) *m/z* (relative intensity): 214.0 (41%) $[M^{81}Br]^{+\bullet}$, 212.0 (42%) $[M^{79}Br]^{+\bullet}$, 41.2 (100%).

1-(Allyloxy)-2-chlorobenzene

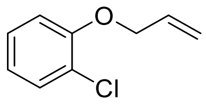

1-(Allyloxy)-2-chlorobenzene was prepared by **General Method C** from 2-chlorophenol (0.643 g, 5.00 mmol) and allyl bromide (0.650 mL, 7.50 mmol). The crude mixture was filtered through a short plug

of silica using hexane as eluent to afford 1-(allyloxy)-2-chlorobenzene (0.801 g, 95%) as a colourless oil.

Spectroscopic data are consistent with those reported.[12,13]

^{1}H NMR (400 MHz, $CDCl_3$) δ = 7.38 (dd, *J* = 7.8, 1.6 Hz, 1H), 7.21 (ddd, *J* = 8.3, 7.4, 1.7 Hz, 1H), 6.92 (ddd, *J* = 15.2, 7.7, 1.4 Hz, 2H), 6.09 (ddt, *J* = 17.3, 10.3, 5.1 Hz, 1H), 5.49 (dq, *J* = 17.3, 1.7 Hz, 1H), 5.33 (dq, *J* = 10.6, 1.5 Hz, 1H), 4.63 (dt, *J* = 5.1, 1.6 Hz, 2H) ppm.

^{13}C NMR (101 MHz, $CDCl_3$) δ = 154.1, 132.7, 130.3, 127.6, 123.0, 121.5, 117.8, 113.8, 69.6 ppm.

LRMS (EI$^+$) *m/z* (relative intensity): 170.1 (15%) $[M^{37}Cl]^{+\bullet}$, 168.0 (48%) $[M^{35}Cl]^{+\bullet}$, 41.2 (100%).

Cyclisation of 1-(allyloxy)-2-iodobenzene (1) to give 3-methyl-2,3-dihydrobenzofuran (2), (allyloxy)benzene (3) and 1,2-bis(2,3-Dihydrobenzofuran-3-yl)ethane (4)

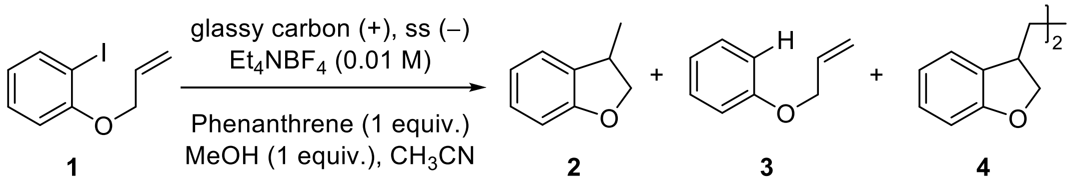

The following gramme-scale experiment was carried out while optimisation of the reaction conditions was ongoing, and allowed isolation of compounds **2**, **3**, **4** and recovery of phenthrene. The conditions are different from the optimised ones described in the main paper. In general, the cyclised product **2** was not isolated due to its volatility.

A solution of 1-(allyloxy)-2-iodobenzene (**1**, 1.30 g, 5.00 mmol), phenanthrene (0.890 g, 5.00 mmol) and Et_4NBF_4 (0.870 g, 4.00 mmol) in MeCN (400 mL) and MeOH (0.20 mL, 5.0 mmol) was degassed under argon for 20 min, and passed through the Ammonite 8 flow reactor (glassy carbon anode / SS cathode) at a flow rate of 16 mL min^{-1}, and cell current of 640 mA. The collected solution was passed through the reactor a second time using the same conditions (overall: 2 x 2.0 F). Steady state terminal voltage was 8.0 to 11.0 V. The effluent solution was concentrated under reduced pressure and the crude was taken into EtOAc (10 mL), allowing the Et_4NBF_4 supporting electrolyte to be recovered by filtration. The crude mixture was purified by flash chromatography (0 – 5% Et_2O in hexane) to afford 3-Methyl-2,3-dihydrobenzofuran (**2**, 0.490 g, 3.7 mmol, 73%) as a colourless oil, recovered phenanthrene (0.873 g, 98%) and 1,2-bis(2,3-dihydrobenzofuran-3-yl)ethane (**4**, 0.137 g, 0.5 mmol, 21%), both isolated as white solids. A small amount of (allyloxy)benzene (**3**, 0.020 g, 3%) was also isolated as a colourless oil.

Using the same method, following purification by Kugelrohr distillation (40 °C, 0.1 mbar), 3-methyl-2,3-dihydrobenzofuran (**2**, 0.456 g, 68%) was isolated as a colourless oil.

Characterisation data for 3-methyl-2,3-dihydrobenzofuran (2)

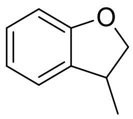

Spectroscopic data are consistent with those reported.[14]

^{1}H NMR (400 MHz, $CDCl_3$) δ =7.16 (m, 1H), 7.13 (m, 1H), 6.88 (td, *J* = 7.4, 1.0 Hz, 1H), 6.79 (m, 1H), 4.69 (dd, *J* = 8.9, 8.6 Hz, 1H), 4.08 (dd, *J* = 8.6, 7.5 Hz, 1H), 3.55 (m, 1H), 1.34 (d, *J* = 6.9 Hz, 3H) ppm.

^{13}C NMR (101 MHz, $CDCl_3$) δ = 159.7, 132.2, 128.0, 123.8, 120.4, 109.4, 78.4, 36.5, 19.3 ppm.

LRMS (EI$^+$) *m/z* (relative intensity): 134.1 (86%) $[M]^{+\bullet}$, 119.1 (100%)

bp: 40 °C at 0.1 mbar (Lit:[15] 60 °C at 5 mbar)

Characterisation data for (allyloxy)benzene (3)

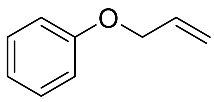

Spectroscopic and physical data (^{1}H NMR, ^{13}C NMR and IR) are consistent with those reported.[11]

^{1}H NMR (400 MHz, $CDCl_3$) δ = 7.33 – 7.27 (m, 2H), 6.99 – 6.91 (m, 3H), 6.08 (ddt, *J* = 17.2, 10.5, 5.3 Hz, 1H), 5.43 (dq, *J* = 17.2, 1.6 Hz, 1H), 5.30 (dq, *J* = 10.5, 1.6 Hz, 1H), 4.56 (dt, *J* = 5.3, 1.5 Hz, 2H) ppm.

^{13}C NMR (101 MHz, $CDCl_3$) δ = 158.6, 133.3, 129.4, 120.8, 117.6, 114.7, 68.7 ppm.

LRMS (EI$^+$) *m/z* (relative intensity): 134.1 (100%) $[M]^{+\bullet}$

HRMS (EI$^+$) *m/z*: $[M]^{+\bullet}$ Calcd for $C_9H_{10}O$: 134.0726; found 134.0726

IR ν_{max} (neat) 3040, 2865, 1598, 1494, 1240, 1032, 924, 752 cm^{-1}

Characterisation data for 1,2-bis(2,3-dihydrobenzofuran-3-yl)ethane (4)

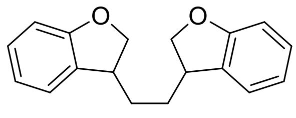

Spectroscopic data (^{1}H NMR, ^{13}C NMR and LRMS) are consistent with those reported.[16]

^{1}H NMR (400 MHz, $CDCl_3$) δ =7.20 – 7.10 (m, 4H), 6.87 (tdd, *J* = 7.4, 1.5, 1.0 Hz, 2H), 6.80 (d, *J* = 8.0, 2H), 4.64 (t, *J* = 8.9 Hz, 2H), 4.22 (ddd, *J* = 8.9, 6.2, 1.4 Hz, 2H), 3.50 – 3.40 (m, 2H), 1.97 – 1.57 (m, 4H) ppm.

^{13}C NMR (101 MHz, $CDCl_3$) δ = 159.9 (2C), 130.4, 130.3, 128.3 (2C), 124.3, 124.2, 120.4, 120.4, 109.6 (2C), 76.6, 76.5, 41.9, 41.9, 32.3, 32.2 ppm.

LRMS (EI$^+$) *m/z* (relative intensity): 266.1 (74%) $[M]^{+\bullet}$, 119.1 (100%)

HRMS (EI⁺) *m/z*: $[M]^{+•}$ Calcd for $C_{18}H_{18}O_2$: 266.1301; found 266.1302.

IR ν_{max} (neat) 2223, 2870, 1593, 1479, 1231, 1015, 954, 752 cm^{-1}.

mp: 84 – 85 °C.

Methyl 4-(allyloxy)-3-bromobenzoate

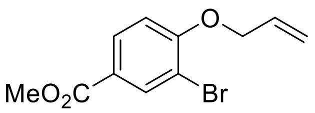

Methyl 4-(allyloxy)-3-bromobenzoate was prepared by **General Method C** from methyl 3-bromo-4-hydroxybenzoate (2.31 g, 10.0 mmol) and allyl bromide (1.30 mL, 15.0 mmol). The crude mixture was filtered through a short plug of silica (hexane:EtOAc = 19:1) to afford methyl 4-(allyloxy)-3-bromobenzoate (2.71 g, quantitative) as a colourless solid.

Spectroscopic data (^{1}H NMR in DMSO-d_6 and LRMS) are consistent with those reported.[17]

^{1}H NMR (400 MHz, $CDCl_3$) δ = 8.25 (d, *J* = 2.1 Hz, 1H), 7.96 (dd, *J* = 8.6, 2.1 Hz, 1H), 6.91 (d, *J* = 8.6 Hz, 1H), 6.07 (m, 1H), 5.51 (dd, *J* = 17.3, 1.2 Hz, 1H), 5.37 (dd, *J* = 10.6, 1.2 Hz, 1H), 4.69 (d, *J* = 5.0 Hz, 2H), 3.90 (s, 3H) ppm.

^{13}C NMR (101 MHz, $CDCl_3$) δ = 165.7, 158.5, 134.9, 131.8, 130.4, 123.8, 118.3, 112.2, 111.9, 69.7, 52.1 ppm.

LRMS (EI⁺) *m/z* (relative intensity): 271.9 (93%) $[M^{81}Br]^{+•}$, 269.9 (100%) $[M^{79}Br]^{+•}$.

HRMS (EI⁺) *m/z*: $[M^{79}Br]^{+•}$ calcd. for $C_{11}H_{11}BrO_3$: 269.9886; found 269.9885.

IR ν_{max} (neat) 1712, 1596, 1434, 1294, 1264, 1231 cm^{-1}.

mp: 45 – 46 °C.

Methyl 3-methyl-2,3-dihydrobenzofuran-5-carboxylate (5)

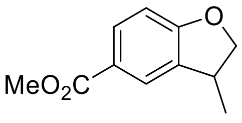

Methyl 3-methyl-2,3-dihydrobenzofuran-5-carboxylate (**5**) was prepared by **General Method A** from methyl 4-(allyloxy)-3-bromobenzoate (0.271 g, 1.00 mmol). The crude mixture was purified by flash chromatography (10% toluene in hexane to Et_2O:toluene:hexane = 5:10:85) to afford methyl 3-methyl-2,3-dihydrobenzofuran-5-carboxylate (**5**, 0.140 g, 73%) as a colourless oil.

Spectroscopic data are consistent with those reported.[18]

^{1}H NMR (400 MHz, $CDCl_3$) δ = 7.90 – 7.83 (m, 2H), 6.79 (d, *J* = 8.3 Hz, 1H), 4.77 (t, *J* = 8.8 Hz, 1H), 4.16 (dd, *J* = 8.8, 7.5 Hz, 1H), 3.88 (s, 3H), 3.62 – 3.52 (m, 1H), 1.36 (d, *J* = 6.9 Hz, 3H) ppm.

^{13}C NMR (101 MHz, $CDCl_3$) δ = 167.0, 163.9, 132.7, 131.1, 125.7, 122.7, 109.1, 79.4, 51.8, 35.9, 19.3 ppm.

LRMS (EI$^+$) *m/z* (relative intensity): 192.0 (100%) $[M]^{+\bullet}$.

1-(Allyloxy)-2-bromo-4-methoxybenzene

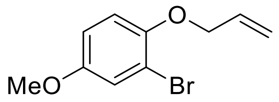

1-(Allyloxy)-2-bromo-4-methoxybenzene was prepared by **General Method C** from 4-methoxy-2-bromophenol (3.65 g, 15.0 mmol) and allyl bromide (1.94 mL, 22.5 mmol). The crude mixture was filtered through a short plug of silica (2.5 – 5% Et_2O in hexane) to afford 1-(allyloxy)-2-bromo-4-methoxybenzene (3.46 g, 95%) as a pale yellow oil.

Spectroscopic data (^{1}H NMR, ^{13}C NMR and LRMS) are consistent with those reported.[19]

^{1}H NMR (400 MHz, $CDCl_3$) δ = 7.13 (d, *J* = 2.9 Hz, 1H), 6.86 (d, *J* = 9.0 Hz, 1H), 6.80 (dd, *J* = 9.0, 2.9 Hz, 1H), 6.07 (ddt, *J* = 17.3, 10.5, 5.1 Hz, 1H), 5.46 (dq, *J* = 17.3, 1.6 Hz, 1H), 5.30 (dq, *J* = 10.5, 1.6 Hz, 1H), 4.55 (dt, *J* = 5.1, 1.6 Hz, 2H), 3.76 (s, 3H) ppm.

^{13}C NMR (101 MHz, $CDCl_3$) δ = 154.2, 149.3, 133.0, 118.8, 117.6, 115.1, 113.6, 112.9, 70.7, 55.8 ppm.

LRMS (EI$^+$) *m/z* (relative intensity): 244.0 (85%) $[M^{81}Br]^{+\bullet}$, 241.9 (93%) $[M^{79}Br]^{+\bullet}$, 201.0 (100%).

HRMS (EI$^+$) *m/z*: $[M^{79}Br]^{+\bullet}$ calcd. for $C_{10}H_{11}BrO_2$: 241.9937; found 241.9938.

IR ν_{max} (neat) 2957, 1488, 1456, 1209, 1036, 994 cm^{-1}.

5-Methoxy-3-methyl-2,3-dihydrobenzofuran (6)

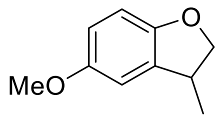

5-Methoxy-3-methyl-2,3-dihydrobenzofuran (**6**) was prepared by **General Method A** from 1-(allyloxy)-2-bromo-4-methoxybenzene (0.243 g, 1.00 mmol). The crude mixture was purified by flash chromatography (10% toluene in hexane to Et_2O:toluene:hexane = 5:10:85) to afford 5-methoxy-3-methyl-2,3-dihydrobenzofuran (**6**, 0.149 g, 91%) as a colourless oil.

5-Methoxy-3-methyl-2,3-dihydrobenzofuran (**6**) was also prepared by **General Method B** from 1-(allyloxy)-2-bromo-4-methoxybenzene (0.239 g, 0.983 mmol). The crude mixture was purified by flash chromatography (15 – 30% CH_2Cl_2 in hexane) to afford 5-methoxy-3-methyl-2,3-dihydrobenzofuran (**6**, 0.084 g, 52%) as a colourless oil.

Spectroscopic data (^{1}H NMR, ^{13}C NMR and LRMS) are consistent with those reported.[19]

^{1}H NMR (400 MHz, $CDCl_3$) δ = 6.80 – 6.62 (m, 3H), 4.67 (t, *J* = 8.4 Hz, 1H), 4.06 (t, *J* = 8.4 Hz, 1H), 3.78 (s, 3H), 3.52 (m, 1H), 1.33 (d, *J* = 6.9 Hz, 3H) ppm.

^{13}C NMR (101 MHz, $CDCl_3$) δ = 154.2, 153.8, 133.3, 112.6, 110.2, 109.2, 78.7, 56.0, 37.0, 19.0 ppm.

LRMS (EI$^+$) *m/z* (relative intensity): 164.1 (97%) $[M]^{+•}$, 148.9 (100%).

HRMS (EI$^+$) *m/z*: $[M]^{+•}$ calcd. for $C_{10}H_{12}O_2$: 164.0832; found 164.0831.

IR $ν_{max}$ (neat) 2924, 2877, 1484, 1451, 1218, 1192, 1018, 967, 755 cm^{-1}.

4-(Allyloxy)-3-bromobenzonitrile

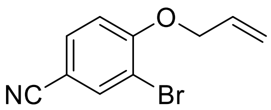

4-(Allyloxy)-3-bromobenzonitrile was prepared by **General Method C** from 4-cyano-2-bromophenol (2.97 g, 15.0 mmol) and allyl bromide (1.94 mL, 22.5 mmol). The crude mixture was filtered through a short plug of silica (hexane:EtOAc = 19:1) to afford 4-(allyloxy)-3-bromobenzonitrile (3.46 g, 97%) as a colourless solid.

Spectroscopic data are consistent with those reported.[19]

^{1}H NMR (400 MHz, $CDCl_3$) δ = 7.84 (d, *J* = 2.0 Hz, 1H), 7.57 (dd, *J* = 8.6, 2.0 Hz, 1H), 6.93 (d, *J* = 8.6 Hz, 1H), 6.05 (ddt, *J* = 17.3, 10.6, 5.0 Hz, 1H), 5.50 (dq, *J* = 17.3, 1.6 Hz, 1H), 5.37 (dd, *J* = 10.6, 1.6 Hz, 1H), 4.69 (dt, *J* = 5.0, 1.6 Hz, 2H) ppm.

^{13}C NMR (101 MHz, $CDCl_3$) δ = 158.5, 136.8, 132.9, 131.3, 118.6, 117.7, 113.1, 112.7, 105.3, 69.9 ppm.

LRMS (EI$^+$) *m/z* (relative intensity): 239.1 (19%) $[M^{81}Br]^{+•}$, 237.1 (20%) $[M^{79}Br]^{+•}$, 41.2 (100%).

IR $ν_{max}$ (neat) 3085, 2226, 1594, 1489, 1260, 1048, 991, 812, 588 cm^{-1}.

3-Methyl-2,3-dihydrobenzofuran-5-carbonitrile (7)

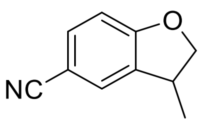

3-Methyl-2,3-dihydrobenzofuran-5-carbonitrile (**7**) was prepared by **General Method A** from 4-(Allyloxy)-3-bromobenzonitrile (0.238 g, 1.00 mmol). The crude mixture was purified by flash chromatography (0 – 5% Et_2O in hexane) to afford 3-methyl-2,3-dihydrobenzofuran-5-carbonitrile (**7**, 0.115 g, 72%) as a colourless solid.

3-Methyl-2,3-dihydrobenzofuran-5-carbonitrile (**7**) was also prepared by **General Method B** from 4-(Allyloxy)-3-bromobenzonitrile (0.238 g, 1.00 mmol). The crude mixture was purified by flash chromatography (0 – 5% CH_2Cl_2 in hexane) to afford 3-methyl-2,3-dihydrobenzofuran-5-carbonitrile (**7**, 0.116 g, 73%) as a colourless solid.

Large scale reaction: The same reaction was performed on a 25 mmol scale by **General Method B** from 4-(Allyloxy)-3-bromobenzonitrile (5.96 g, 25.0 mmol). The solution coming from the electrochemical reactor was collected over $Na_2S_2O_3$ (aq.). The solvent (MeCN) was evaporated under reduced pressure, the crude mixture was extracted with CH_2Cl_2 (3 x 20 mL). The combined organic layers were washed with brine (1x 30 mL) and concentrated under vacuum. The crude mixture was purified by flash chromatography (0 – 5% CH_2Cl_2 in hexane) to afford 3-methyl-2,3-dihydrobenzofuran-5-carbonitrile (**7**, 2.83 g, 71%) as a colourless solid.

Spectroscopic data are consistent with those reported.[19]

^{1}H NMR (400 MHz, $CDCl_3$) δ = 7.48 – 7.40 (m, 2H), 6.83 (d, *J* = 8.3 Hz, 1H), 4.79 (dd, *J* = 9.0, 9.0 Hz, 1H), 4.19 (dd, *J* = 8.9, 7.3 Hz, 1H), 3.58 (m, 1H), 1.36 (d, *J* = 6.9 Hz, 2H) ppm.

^{13}C NMR (101 MHz, $CDCl_3$) δ = 163.4, 133.9, 133.6, 128.0, 119.6, 110.4, 103.7, 79.4, 35.9, 19.3 ppm.

LRMS (EI$^+$) *m/z* (relative intensity): 159.1 (82%) $[M]^{+\bullet}$, 144.1 (100%).

IR ν_{max} (neat) 2970, 2889, 2216, 1738, 1608, 1483, 1365, 1242, 956, 826, 700 cm^{-1}.

1-Iodo-2-((3-methylbut-2-en-1-yl)oxy)benzene

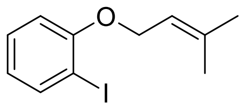

1-Iodo-2-((3-methylbut-2-en-1-yl)oxy)benzene was prepared by **General Method C** from 2-iodophenol (3.30 g, 15.0 mmol) and 3,3-dimethylallyl bromide (3.00 mL, 22.5 mmol). The crude mixture was purified by flash chromatography (hexane:Et_2O 19:1) to afford 1-iodo-2-((3-methylbut-2-en-1-yl)oxy)benzene (3.97 g, 92%) as a colourless oil.

Spectroscopic data are consistent with those reported ((^{1}H NMR and IR)[15]; (^{1}H NMR and ^{13}C NMR)[20]).

^{1}H NMR (400 MHz, $CDCl_3$) δ = 7.78 (dd, *J* = 7.6, 1.6 Hz, 1H), 7.29 (ddd, *J* = 8.2, 7.6, 1.6 Hz, 1H), 6.84 (dd, *J* = 8.2, 1.3 Hz, 1H), 6.71 (td, *J* = 7.6, 1.3 Hz, 1H), 5.52 (m, 1H), 4.60 (d, *J* = 6.5 Hz, 2H), 1.81 (s, 3H), 1.76 (s, 3H) ppm.

^{13}C NMR (101 MHz, $CDCl_3$) δ = 157.5, 139.5, 137.9, 129.3, 122.4, 119.5, 112.7, 86.9, 66.3, 25.8, 18.4 ppm.

LRMS (EI$^+$) *m/z* (relative intensity): 288.1 (5%) $[M]^{+\bullet}$, 219.9 (100%).

HRMS (EI$^+$) *m/z*: $[M]^{+\bullet}$ Calcd for $C_{11}H_{13}IO$: 288.0011; found 288.0005.

IR ν_{max} (neat) 2972, 2912, 1581, 1468, 1274, 1229, 1016,992, 745 cm^{-1}.

3-Isopropyl-2,3-dihydrobenzofuran (8)

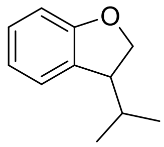

3-Isopropyl-2,3-dihydrobenzofuran (**8**) was prepared by **General Method A** from 1-iodo-2-((3-methylbut-2-en-1-yl)oxy)benzene (0.288 g, 1.00 mmol). The crude mixture was purified by flash chromatography using hexane as eluent to afford 3-Isopropyl-2,3-dihydrobenzofuran (**8**, 0.016 g, 10%) as a colourless oil.

3-Isopropyl-2,3-dihydrobenzofuran (**8**) was also prepared by **General Method B** from 1-iodo-2-((3-methylbut-2-en-1-yl)oxy)benzene (0.288 g, 1.00 mmol). The crude mixture was purified by flash chromatography (hexane:toluene = 19:1) to afford 3-isopropyl-2,3-dihydrobenzofuran (**8**, 0.066 g, 41%) as a colourless oil.

Spectroscopic data are consistent with those reported.[21]

^{1}H NMR (400 MHz, $CDCl_3$) δ = 7.19 (m, 1H), 7.15 – 7.11 (m, 1H), 6.85 (td, *J* = 7.4, 1.0 Hz, 1H), 6.78 (m, 1H), 4.53 (dd, *J* = 9.1, 9.1 Hz, 1H), 4.39 (dd, *J* = 9.1, 5.2 Hz, 1H), 3.33 (m, 1H), 1.98 (dsept, *J* = 6.8, 5.2 Hz, 1H), 0.97 (d, *J* = 6.8 Hz, 3H), 0.89 (d, *J* = 6.8 Hz, 3H) ppm.

^{13}C NMR (101 MHz, $CDCl_3$) δ = 160.4, 129.4, 128.1, 125.1, 120.0, 109.3, 73.8, 48.2, 31.7, 19.8, 18.4 ppm.

LRMS (EI$^+$) *m/z* (relative intensity): 162.2 (37%) $[M]^{+\bullet}$, 119.1 (100%).

2-Iodo-3-((3-methylbut-2-en-1-yl)oxy)pyridine

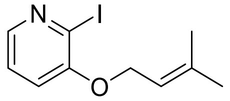

2-Iodo-3-((3-methylbut-2-en-1-yl)oxy)pyridine was prepared by **General Method C** from 2-iodo-hydroxypyridine (1.66 g, 7.51 mmol) and 3,3-dimethylallyl bromide (1.18 g, 7.92 mmol). The crude mixture was purified by flash chromatography (10 – 20% EtOAc in petroleum ether) to afford 2-iodo-3-((3-methylbut-2-en-1-yl)oxy)pyridine (1.76 g, 81%) as a yellow oil.

^{1}H NMR (400 MHz, $CDCl_3$) δ = 7.99 (dd, *J* = 4.6, 1.5 Hz, 1H), 7.17 (dd, *J* = 8.2, 4.6 Hz, 1H), 6.99 (dd, *J* = 8.2, 1.5 Hz, 1H), 5.48 (m, 1H), 4.61 (d, *J* = 6.5 Hz, 2H), 1.80 (s, 3H), 1.76 (s, 3H) ppm.

^{13}C NMR (101 MHz, $CDCl_3$) δ = 154.6, 142.4, 139.1, 123.3, 118.6, 118.5, 112.5, 66.3, 25.8, 18.4 ppm.

LRMS (ESI$^+$) *m/z*: 290.2 $[M+H]^+$

HRMS (ESI$^+$) *m/z*: $[M+H]^+$ Calcd. for $C_{10}H_{13}INO$: 290.0036; found 290.0030.

IR ν_{max} (neat) 2980, 2932, 2911, 1556, 1440, 1402, 1277 cm^{-1}.

3-Isopropyl-2,3-dihydrofuro[3,2-b]pyridine (9)

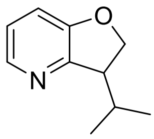

3-Isopropyl-2,3-dihydrofuro[3,2-b]pyridine (**9**) was prepared by **General Method B** from 2-iodo-3-((3-methylbut-2-en-1-yl)oxy)pyridine (0.293 g, 1.0 mmol). The crude mixture was purified by flash column chromatography (10% EtOAc in hexane) to afford 3-isopropyl-2,3-dihydrofuro[3,2-b]pyridine (**9**, 0.073 g, 45%) as a yellow oil.

Spectroscopic data (^{1}H NMR) are consistent with those reported.[22]

^{1}H NMR (400 MHz, $CDCl_3$) δ = 8.08 (dd, *J* = 3.7, 2.5 Hz, 1H), 7.04 – 7.00 (m, 2H), 4.64 (t, *J* = 9.5 Hz, 1H), 4.48 (dd, *J* = 9.5, 5.8 Hz, 1H), 3.41 (ddd, *J* = 9.5, 5.8, 4.9 Hz, 1H), 2.24 (dsept, *J* = 6.9, 4.9 Hz, 1H), 1.04 (d, *J* = 6.9 Hz, 3H), 0.88 (d, *J* = 6.9 Hz, 3H) ppm.

^{13}C NMR (101 MHz, $CDCl_3$) δ = 154.2, 152.8, 141.2, 122.4, 115.6, 73.2, 47.9, 30.8, 20.1, 17.8 ppm.

LRMS (EI$^+$) *m/z*: 163.1 (30%) $[M]^{+\bullet}$, 120.1 (100%)

HRMS (EI$^+$) *m/z*: $[M]^{+\bullet}$ calcd. for $C_{10}H_{13}NO$: 163.0992; found 163.0991.

IR ν_{max} (neat) 2960, 2873, 1576, 1428, 1262 cm^{-1}.

2-Iodo-3-((2-methylallyl)oxy)pyridine

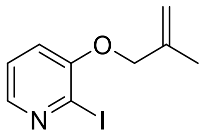

2-Iodo-3-((2-methylallyl)oxy)pyridine was prepared by **General Method C** from 3-hydroxy-2-iodopyridine (2.21 g, 10.0 mmol) and 3-bromo-2-methylprop-1-ene (2.03 g, 15.0 mmol). The crude mixture was purified by flash column chromatography (10% Et_2O in hexane) to afford 2-iodo-3-((2-methylallyl)oxy)pyridine (1.66 g, 95%) as a yellow oil.

^{1}H NMR (400 MHz, $CDCl_3$) δ = 8.00 (dd, *J* = 4.6, 1.5 Hz, 1H), 7.17 (dd, *J* = 8.2, 4.6 Hz, 1H), 6.98 (dd, *J* = 8.2, 1.5 Hz, 1H), 5.20 – 5.16 (m, 1H), 5.08 – 5.04 (m, 1H), 4.52 (s, 2H), 1.88 (s, 3H) ppm.

^{13}C NMR (101 MHz, $CDCl_3$) δ = 154.2, 142.7, 139.3, 123.3, 118.1, 113.7, 112.1, 72.6, 19.3 ppm.

LRMS (EI$^+$) *m/z* (relative intensity): 274.9 (100%) $[M+H]^{+\bullet}$.

HRMS (ESI$^+$) *m/z*: $[M+H]^+$ Calcd for $C_9H_{11}INO$: 275.9880, found 275.9881.

IR ν_{max} (neat) 3056, 2974, 2914, 1657, 1439, 1362 cm^{-1}.

3,3-Dimethyl-2,3-dihydrofuro[3,2-*b*]pyridine (10)

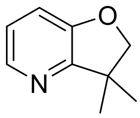

3,3-Dimethyl-2,3-dihydrofuro[3,2-*b*]pyridine (**10**) was prepared by **General Method A** from 2-iodo-3-((2-methylallyl)oxy)pyridine (0.275 g, 1.00 mmol). The crude oil was treated with 2M HCl (3 x 10 mL) and the organic phase set aside. The combined aqueous phase was adjusted to pH 12 by addition of 5% aq. NaOH solution. The product was extracted with Et_2O (3 x 10 mL). The combined organic phase was washed with brine (20 mL), dried ($MgSO_4$) and the solvent was removed under reduced pressure. The crude mixture was purified by flash chromatography (30% Et_2O in hexane) to afford 3,3-dimethyl-2,3-dihydrofuro[3,2-*b*]pyridine (**10**, 0.112 g, 75%) as an amber oil.

^{1}H NMR (400 MHz, $CDCl_3$) δ = 8.06 (dd, *J* = 4.2, 2.1 Hz, 1H), 7.03 – 6.97 (m, 2H), 4.32 (s, 2H), 1.38 (s, 6H) ppm.

^{13}C NMR (101 MHz, $CDCl_3$) δ = 157.4, 152.6, 141.7, 122.1, 115.9, 84.2, 41.3, 25.9 ppm.

LRMS (EI$^+$) *m/z* (relative intensity): 149.0 (99%) $[M]^{+\bullet}$, 133.1 (100%).

HRMS (EI$^+$) *m/z*: $[M+H]^+$ Calcd for $C_9H_{12}NO$: 150.0913, found 150.0912.

IR ν_{max} (neat) 1475, 1453, 1220, 1059, 705 cm^{-1}.

1-(Cinnamyloxy)-2-iodobenzene (*E:Z* ~ 14:1)

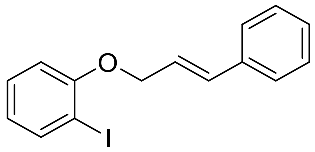

1-(Cinnamyloxy)-2-iodobenzene was prepared by **General Method C** from 2-iodophenol (2.20 g, 10.0 mmol) and cinnamyl bromide (1.63 mL, 11.0 mmol). The crude mixture was filtered through a short plug of silica using hexane as eluent to afford 1-(cinnamyloxy)-2-iodobenzene (3.09 g, 92%) as a colourless oil (*E:Z* ~ 14:1, ^{1}H NMR).

Spectroscopic data are consistent with those reported.[8] (Signals reported are for the major *E* isomer).

^{1}H NMR (400 MHz, $CDCl_3$) δ = 7.81 (dd, *J* = 7.8, 1.6 Hz, 1H), 7.46 – 7.42 (m, 2H), 7.38 – 7.22 (m, 4H), 6.89 (dd, *J* = 8.2, 1.3 Hz, 1H), 6.84 (dt, *J* = 16.0, 1.5 Hz, 1H), 6.74 (m, 1H), 6.43 (dt, *J* = 16.0, 5.4 Hz, 1H), 4.78 (dd, *J* = 5.4, 1.6 Hz, 2H) ppm.

^{13}C NMR (101 MHz, $CDCl_3$) δ = 157.4, 139.7, 136.6, 133.1, 129.6, 128.7, 128.1, 126.8, 124.1, 122.9, 112.9, 87.0, 69.9 ppm.

LRMS (EI$^+$) *m/z* (relative intensity): 336.2 (1%) $[M]^{+\bullet}$, 117.2 (100%).

1-Chloro-2-(cinnamyloxy)benzene

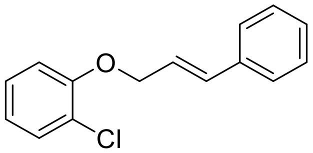

1-Chloro-2-(cinnamyloxy)benzene was prepared by **General Method C** from 2-chlorophenol (0.643 g, 0.52 mL, 5.0 mmol) and cinnamyl bromide (0.74 mL, 5.0 mmol). The crude mixture was filtered through a short plug of silica using hexane as eluent to afford 1-chloro-2-(cinnamyloxy)benzene (0.900 g, 74%) as a colourless oil.

Spectroscopic data are consistent with those reported.[43]

^{1}H NMR (400 MHz, $CDCl_3$) δ = 7.44 – 7.40 (m, 2H), 7.39 (dd, J = 7.9, 1.6 Hz, 1H), 7.36 – 7.30 (m, 2H), 7.26 (m, 1H), 7.21 (ddd, J = 8.2, 7.6, 1.6 Hz, 1H), 6.99 (dd, J = 8.2, 1.6 Hz, 1H), 6.91 (td, J = 7.6, 1.6 Hz, 1H), 6.78 (d, J = 16.0 Hz, 1H), 6.43 (dt, J = 16.0, 5.7 Hz, 2H), 4.79 (dd, J = 5.7, 1.6 Hz, 1H) ppm.

^{13}C NMR (101 MHz, $CDCl_3$) δ = 154.2, 136.3, 133.2, 130.4, 128.6, 128.0, 127.7, 126.6, 123.9, 123.2, 121.6, 114.0, 69.7 ppm.

LRMS (EI$^+$) *m/z* (relative intensity): 246.1 (20%) $[M^{37}Cl]^{+\bullet}$, 244.1 (59%) $[M^{35}Cl]^{+\bullet}$, 207.1 (100%).

3-Benzyl-2,3-dihydrobenzofuran (11)

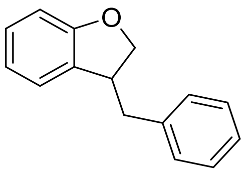

3-Benzyl-2,3-dihydrobenzofuran (**11**) was prepared by **General Method B** from 1-(cinnamyloxy)-2-iodobenzene (0.336 g, 1.00 mmol). The crude mixture was purified by flash chromatography (hexane:toluene 19:1) to afford 3-benzyl-2,3-dihydrobenzofuran (**11**, 0.155 g, 74%) as a colourless solid.

The same reaction was attempted starting from 1-(cinnamyloxy)-2-chlorobenzene (0.245 g, 1.00 mmol). It led to electrode fouling (anode) and only starting material (185 mg, 76%) and supporting electrolyte was observed in the crude ^{1}H NMR. The starting material 1-(cinnamyloxy)-2-chlorobenzene was recovered in 76% yield (0.185 g).

Spectroscopic data are consistent with those reported.[21]

^{1}H NMR (400 MHz, $CDCl_3$) δ = 7.37 – 7.31 (m, 2H), 7.27 (m, 1H), 7.24 – 7.19 (m, 2H), 7.15 (m, 1H), 7.00 (d, *J* = 7.3 Hz, 1H), 6.87 – 6.80 (m, 2H), 4.55 (t, *J* = 9.0 Hz, 1H), 4.31 (dd, *J* = 9.0, 6.0 Hz, 1H), 3.78 (m, 1H), 3.09 (dd, *J* = 13.8, 6.3 Hz, 1H), 2.87 (dd, *J* = 13.8, 9.0 Hz, 1H) ppm.

^{13}C NMR (101 MHz, $CDCl_3$) δ = 160.1, 139.3, 130.4, 129.1, 128.7, 128.5, 126.6, 124.7, 120.4, 109.8, 76.4, 43.6, 41.2 ppm.

LRMS (EI$^+$) *m/z* (relative intensity): 210.2 (9%) $[M]^{+\bullet}$, 119.1 (100%).

3-Bromo-4-(cyclohex-2-en-1-yloxy)benzonitrile

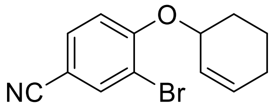

3-Bromo-4-(cyclohex-2-en-1-yloxy)benzonitrile was prepared by **General Method C** from 4-cyano-2-bromophenol (9.90 g, 50.0 mmol) and 3-bromocyclohexene (6.90 mL, 60.0 mmol). The crude mixture was filtered through a short plug of silica (hexane:EtOAc = 19:1) to afford 3-bromo-4-(cyclohex-2-en-1-yloxy)benzonitrile (13.2 g, 95%) as a colourless oil.

^{1}H NMR (400 MHz, $CDCl_3$) δ = 7.83 (d, *J* = 2.1 Hz, 1H), 7.56 (dd, *J* = 8.6, 2.1 Hz, 1H), 6.98 (d, *J* = 8.6 Hz, 1H), 6.05 (m, 1H), 5.85 (m, 1H), 4.90 (m, 1H), 2.19 (m, 1H), 2.07 (dddt, *J* = 8.7, 5.3, 3.5, 1.8 Hz, 1H), 1.99 – 1.86 (m, 3H), 1.68 (m, 1H) ppm.

^{13}C NMR (101 MHz, $CDCl_3$) δ = 158.2, 137.0, 133.9, 132.8, 124.4, 117.9, 114.1, 113.6, 104.8, 72.9, 28.2, 25.0, 18.7 ppm.

LRMS (EI$^+$) *m/z* (relative intensity): 279.1 (97%) $[M^{81}Br]^{+\bullet}$, 277.1 (100%) $[M^{79}Br]^{+\bullet}$.

HRMS (EI$^+$) *m/z*: $[M^{79}Br]^{+\bullet}$ Calcd for $C_{13}H_{12}{}^{79}BrNO$: 277.0097; found 277.0098.

IR ν_{max} (neat) 2934, 2833, 2225, 1593, 1487, 1257, 1045, 938, 813, 726 cm^{-1}.

5a,6,7,8,9,9a-Hexahydrodibenzo[*b*,*d*]furan-2-carbonitrile (12)

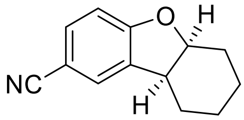

5a,6,7,8,9,9a-Hexahydrodibenzo[*b*,*d*]furan-2-carbonitrile (**12**) was prepared by **General Method B** from 3-Bromo-4-(cyclohex-2-en-1-yloxy)benzonitrile (0.278 g, 1.00 mmol) and phenanthrene (0.036 g, 0.20 mmol). The crude mixture was purified by flash chromatography (0 – 5% CH_2Cl_2 in hexane) to afford 5a,6,7,8,9,9a-hexahydrodibenzo[*b*,*d*]furan-2-carbonitrile (**12**, 0.137 g, 69%) as a colourless oil.

^{1}H NMR (400 MHz, $CDCl_3$) δ = 7.44 (ddd, *J* = 8.3, 1.8, 0.5 Hz, 1H), 7.40 (m, 1H), 6.84 (d, *J* = 8.3 Hz, 1H), 4.77 (dt, *J* = 7.1, 5.1 Hz, 1H), 3.25 (dt, *J* = 7.1, 7.0 Hz, 1H), 1.99 – 1.80 (m, 3H), 1.63 – 1.44 (m, 5H), 1.38 (m, 1H) ppm.

^{13}C NMR (101 MHz, $CDCl_3$) δ = 163.3, 135.0, 133.6, 127.7, 119.9, 111.0, 103.7, 84.1, 40.1, 28.0, 27.4, 21.7, 20.2 ppm.

LRMS (EI$^+$) *m/z* (relative intensity): 199.2 (6%) $[M]^{+\bullet}$, 41.1 (100%).

HRMS (EI$^+$) *m/z*: $[M]^{+\bullet}$ Calcd for $C_{13}H_{13}NO$: 199.0992; found 199.0992.

IR ν_{max} (neat) 2934, 2860, 2221, 1610, 1476, 1241, 1123, 941, 825 cm^{-1}.

3-(Cyclohex-2-en-1-yloxy)-2-iodopyridine

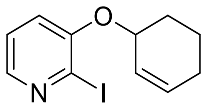

3-(Cyclohex-2-en-1-yloxy)-2-iodopyridine was prepared by **General Method C** from 3-hydroxy-2-iodopyridine (3.32 g, 15.0 mmol) and 3-bromocyclohexene (3.62 g, 22.5 mmol). The crude mixture was filtered through a short plug of silica (30% Et_2O in hexane) to afford 3-(cyclohex-2-en-1-yloxy)-2-iodopyridine (2.72 g, 60%) as a colourless solid.

Spectroscopic data are consistent with those reported (^{1}H NMR and LRMS).[23]

^{1}H NMR (400 MHz, $CDCl_3$) δ = 7.99 (dd, *J* = 4.5, 1.4 Hz, 1H), 7.16 (dd, *J* = 8.3, 4.5 Hz, 1H), 7.05 (dd, *J* = 8.3, 1.4 Hz, 1H), 6.05 (m, 1H), 5.87 (m, 1H), 4.80 (m, 1H), 2.20 (m, 1H), 2.07 (m, 1H), 2.02 – 1.90 (m, 3H), 1.70 (m, 1H) ppm.

^{13}C NMR (101 MHz, $CDCl_3$) δ = 153.9, 142.7, 133.5, 124.8, 123.2, 119.8, 113.9, 73.0, 28.2, 25.0, 18.7 ppm.

LRMS (EI$^+$) *m/z* (relative intensity): 300.9 (83%) $[M]^{+\bullet}$, 221.9 (100%).

HRMS (ESI$^+$) *m/z*: $[M+H]^+$ Calcd for $C_{11}H_{13}INO$: 302.0036, found 302.0038.

IR ν_{max} (neat) 3057, 2932, 1602, 1448, 1420, 1213, 856 cm^{-1}.

(5a*S*,9a*S*)-5a,6,7,8,9,9a-Hexahydrobenzofuro[3,2-*b*]pyridine (13)

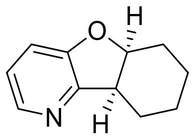

(5a*S*,9a*S*)-5a,6,7,8,9,9a-Hexahydrobenzofuro[3,2-*b*]pyridine (**13**) was prepared by **General Method A** from 3-(cyclohex-2-en-1-yloxy)-2-iodopyridine (0.301 g, 1.00 mmol). The crude mixture was purified by flash chromatography (50% Et_2O in hexane) to afford (5a*S*,9a*S*)-5a,6,7,8,9,9a-hexahydrobenzofuro[3,2-*b*]pyridine (**13**, 0.161 g, 92%) as an amber oil.

Spectroscopic data are consistent with those reported (^{1}H NMR and LRMS).[23]

^{1}H NMR (400 MHz, $CDCl_3$) δ = 8.05 (dd, *J* = 4.4, 1.8 Hz, 1H), 7.07 – 6.99 (m, 2H), 4.80 (dt, *J* = 7.2, 5.1 Hz, 1H), 3.27 (dd, *J* = 14.6, 7.2 Hz, 1H), 2.04 – 1.86 (m, 3H), 1.68 (m, 1H), 1.61 – 1.47 (m, 3H), 1.41 (m, 1H) ppm.

^{13}C NMR (101 MHz, $CDCl_3$) δ = 155.9, 153.2, 141.4, 122.2, 116.0, 82.5, 41.1, 27.6, 26.4, 21.8, 20.3 ppm.

LRMS (EI$^+$) *m/z* (relative intensity): 175.1 (75%) $[M]^{+\bullet}$, 146.0 (100%).

HRMS (ESI$^+$) *m/z*: $[M+H]^+$ Calcd for $C_{11}H_{14}NO$: 176.1070, found 176.1070.

IR ν_{max} (neat) 1448, 1420, 1213, 1186, 855 cm^{-1}.

1-(Cyclohex-2-en-1-yloxy)-2-iodobenzene

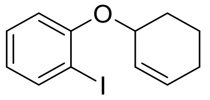

To a solution of 2-iodophenol (20.9 g, 69.5 mmol) in DMF (100 mL) was added K_2CO_3 (19.2 g, 139 mmol) at rt. The resulting suspension was heated at 80 °C for 90 min, then allowed to cool to rt. 3-Bromocyclohexene (11.2 g, 69.5 mmol) was added dropwise, then the suspension was stirred for 16 h at 60 °C and then allowed to cool to rt. H_2O (100 mL) was added to the mixture, which was extracted with Et_2O (3 × 250 mL). The combined organic extracts were washed with water (3 × 100 mL), brine (100 mL), dried ($MgSO_4$) and concentrated under reduced pressure. The crude mixture was purified by flash chromatography (2% Et_2O in hexane) to afford 1-(cyclohex-2-en-1-yloxy)-2-iodobenzene (16.9 g, 81%) as a colourless oil.

Spectroscopic data are consistent with those reported.[24]

^{1}H NMR (400 MHz, $CDCl_3$) δ = 7.79 (dd, *J* = 7.8, 1.6 Hz, 1H), 7.28 (ddd, *J* = 8.2, 7.5, 1.6 Hz, 1H), 6.90 (dd, *J* = 8.2, 1.0 Hz, 1H), 6.71 (dt, *J* = 7.5, 1.4 Hz, 1H), 6.00 (m, 1H), 5.92 (m, 1H), 4.84 – 4.77 (m, 1H), 2.19 (m, 1H), 2.06 (m, 1H), 1.99 – 1.91 (m, 3H), 1.67 (m, 1H) ppm.

^{13}C NMR (101 MHz, $CDCl_3$) δ = 156.8, 139.6, 132.5, 129.2, 125.8, 122.6, 114.4, 88.5, 72.7, 28.4, 25.1, 18.9 ppm.

LRMS (EI$^+$) *m/z* (relative intensity): 299.9 (49%) $[M]^{+\bullet}$, 80.6 (100%).

1-Chloro-2-(cyclohex-2-en-1-yloxy)benzene

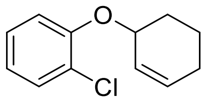

1-Chloro-2-(cyclohex-2-en-1-yloxy)benzene was prepared by **General Method C** from 2-chlorophenol (0.643 g, 0.52 mL, 5.0 mmol) and 3-bromocyclohexene (0.63 mL, 5.5 mmol). The crude mixture was filtered through a short plug of silica using hexane as eluent to afford 1-chloro-2-(cyclohex-2-en-1-yloxy)benzene (0.652 g, 63%) as a colourless oil.

^{1}H NMR (400 MHz, $CDCl_3$) δ = 7.36 (dd, J = 7.8, 1.7 Hz, 1H), 7.19 (ddd, J = 8.3, 7.5, 1.7 Hz, 1H), 7.00 (dd, J = 8.3, 1.4 Hz, 1H), 6.89 (ddd, J = 7.8, 7.5, 1.4 Hz, 1H), 5.99 (dtd, J = 10.1, 3.6, 1.2 Hz, 1H), 5.90 (m, 1H), 4.79 (m, 1H), 2.16 (m, 1H), 2.03 (m, 1H), 1.96 – 1.86 (m, 3H), 1.65 (m, 1H) ppm.

^{13}C NMR (101 MHz, $CDCl_3$) δ 153.9, 132.7, 130.6, 127.6, 126.1, 124.6, 121.8, 116.4, 73.0, 28.5, 25.3, 19.1 ppm.

LRMS (EI$^+$) *m/z* (relative intensity): 210.1 (3%) $[M^{37}Cl]^{+\bullet}$, 208.1 (10%) $[M^{35}Cl]^{+\bullet}$, 80.1 (100%).

IR ν_{max} (neat) 2981, 2889, 1476, 1239, 1058, 949, 744, 722 cm^{-1}.

(4aS,9bS)-1,2,3,4,4a,9b-Hexahydrodibenzo[*b*,*d*]furan (14)

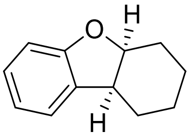

(4aS,9bS)-1,2,3,4,4a,9b-Hexahydrodibenzo[*b*,*d*]furan (**14**) was prepared by **General Method A** from 1-(cyclohex-2-en-1-yloxy)-2-iodobenzene (0.30 g, 1.00 mmol). The crude mixture was purified by flash chromatography (0 to 0.5% Et_2O in hexane) to afford (4aS,9bS)-1,2,3,4,4a,9b-hexahydrodibenzo[*b*,*d*]furan (**14**, 0.145 g, 84%) as a colourless oil.

(4aS,9bS)-1,2,3,4,4a,9b-Hexahydrodibenzo[*b*,*d*]furan (**14**) was also prepared by **General Method B** from 1-(cyclohex-2-en-1-yloxy)-2-iodobenzene (1.50 g, 5.00 mmol). The crude mixture was purified by flash chromatography (5% toluene in hexane) to afford (4aS,9bS)-1,2,3,4,4a,9b-hexahydrodibenzo[*b*,*d*]furan (**14**, 0.281 g, 32%) as a colourless oil.

(4aS,9bS)-1,2,3,4,4a,9b-Hexahydrodibenzo[*b*,*d*]furan (**14**) was also prepared by **General Method B** from 1-chloro-2-(cyclohex-2-en-1-yloxy)benzene (0.209 g, 1.00 mmol). The crude mixture was purified by flash chromatography (5% toluene in hexane) to afford (4aS,9bS)-1,2,3,4,4a,9b-hexahydrodibenzo[*b*,*d*]furan (**14**, 0.046 g, 27%) as a colourless oil.

Spectroscopic data (^{1}H NMR and ^{13}C NMR) are consistent with those reported.[15,25]

^{1}H NMR (400 MHz, $CDCl_3$) δ = 7.17 – 7.11 (m, 2H), 6.87 (td, *J* = 7.4, 1.0 Hz, 1H), 6.83 (m, 1H), 4.68 (dt, *J* = 6.9, 5.0 Hz, 1H), 3.21 (dd, *J* = 14.2, 7.0 Hz, 1H), 1.99 (m, 1H), 1.92 – 1.78 (m, 2H), 1.60 – 1.48 (m, 4H), 1.38 (m, 1H) ppm.

^{13}C NMR (101 MHz, $CDCl_3$) δ = 159.3, 133.5, 127.7, 123.5, 120.4, 110.0, 82.5, 40.6, 28.3, 27.5, 22.0, 20.6 ppm.

LRMS (ESI$^+$) *m/z*: 175.2 $[M+H]^+$.

***tert*-Butyl 4-hydroxy-4-((phenylsulfinyl)methyl)piperidine-1-carboxylate**

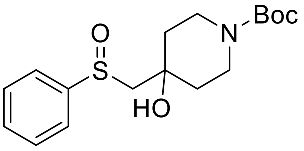

To a solution of diisopropylamine (2.24 mL, 16.0 mmol) in THF (5 mL) was added *n*-BuLi (6 mL of 2.5 M in hexanes, 15 mmol) dropwise at −78 °C, with mechanical stirring under nitrogen for 30 min. To this solution was added a solution of (±)-methyl phenyl sulfoxide (1.40 g, 10.0 mmol) in THF (20 mL) dropwise over 5 min at −78 °C. The reaction mixture was allowed to warm to 5 °C, and left to stir for 20 min. The resulting milky suspension was cooled to −78 °C and treated with a solution of 1-boc-4-piperidone (2.39 g, 12.0 mmol) in THF (24 mL) and the solution was allowed to slowly warm to rt. After 18 h the resulting light-yellow suspension was quenched by the addition of solid NH_4Cl (1.60 g, 30.0

mmol) and concentrated under reduced pressure. H_2O (100 mL) was added to the mixture, which was extracted with EtOAc (3 × 100 mL). The combined organic extracts were washed with water (3 × 100 mL), brine (100 mL), dried ($MgSO_4$) and concentrated under reduced pressure to afford an off-white solid. The crude mixture was purified by flash chromatography (50% EtOAc in petrol ether) to afford *tert*-butyl 4-hydroxy-4-((phenylsulfinyl)methyl)piperidine-1-carboxylate (3.35 g, 80%) as an off-white solid.

Spectroscopic data (^{1}H NMR) are consistent with those reported.[26]

^{1}H NMR (400 MHz, $CDCl_3$) δ = 7.68 – 7.63 (m, 2H), 7.60 – 7.51 (m, 3H), 4.17 (s, 1H) 4.02 (m, 1H) 3.84 (m, 1H), 3.37 – 3.19 (m, 2H), 3.05 (d, *J* = 13.3 Hz, 1H), 2.69 (d, *J* = 13.3 Hz, 1H), 2.19 (dq, *J* = 13.2, 2.8 Hz, 1H), 1.84 (td, *J* = 13.1, 4.7 Hz, 1H), 1.74 – 1.62 (m, 2H), 1.47 (s, 9H) ppm.

^{13}C NMR (101 MHz, $CDCl_3$) δ = 154.8, 143.5, 131.5, 129.6, 123.9, 79.5, 70.3, 66.6, 38.2, 36.1, 28.4 ppm.

LRMS (ESI$^+$) *m/z*: 362.4 $[M+Na]^+$.

tert-Butyl 3-hydroxy-4-methylenepiperidine-1-carboxylate

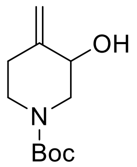

To a solution of *tert*-butyl 4-hydroxy-4-((phenylsulfinyl)methyl)piperidine-1-carboxylate (3.35 g, 12.1 mmol) in *tert*-butanol (30 mL) was added *t*-BuOK (2.03 g, 18.1 mmol) at rt. The resulting yellow suspension was heated at reflux for 16 h. The reaction mixture was then cooled to rt and quenched by the addition of NH_4Cl (1.94 g, 36.3 mmol), stirring for 10 min, before concentration under reduced pressure. H_2O (100 mL) was added to the crude mixture, which was extracted with EtOAc (3 × 100 mL). The combined organic layers were washed with water (2 × 100 mL), brine (100 mL), dried ($MgSO_4$) and concentrated under reduced pressure to afford a brown oil. The crude mixture was purified by flash chromatography (20% EtOAc in hexane) to afford *tert*-butyl 3-hydroxy-4-methylenepiperidine-1-carboxylate (1.86 g, 72%) as an amber solid.

^{1}H NMR data are consistent with those reported.[26]

^{1}H NMR (400 MHz, $CDCl_3$) δ = 5.03 (s, 1H), 4.88 (m, 1H), 4.12 (br s, 1H), 3.76 (ddd, *J* = 12.9, 4.1, 0.9 Hz, 1H), 3.56 (m, 1H), 3.32 – 3.14 (m, 2H), 2.45 (m, 1H), 2.21 – 1.87 (m, 2H), 1.48 (s, 9H) ppm.

^{13}C NMR (101 MHz, $CDCl_3$) δ = 155.1, 147.0, 108.3, 80.0, 70.1, 51.5, 45.0, 32.1, 28.4 ppm.

LRMS (EI$^+$) *m/z* (relative intensity): 157.0 (65%) $[M–C_4H_8]^{+\bullet}$, 138.9 (66%) $[M–C_4H_8–H_2O]^{+\bullet}$, 111.9 (34%), 56.8 (100%).

tert-Butyl 4-(chloromethyl)-3,6-dihydropyridine-1(2H)-carboxylate

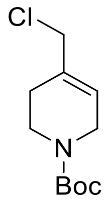

To a solution of *tert*-butyl 3-hydroxy-4-methylenepiperidine-1-carboxylate (2.50 g, 11.7 mmol) in toluene (60 mL) at 60 °C was added thionyl chloride (1.02 mL, 14.1 mmol). After 30 min the reaction mixture was then cooled to 5 °C, then quenched with sat. aq. $NaHCO_3$ (100 mL) at 5 °C and the layers were separated. The organic extract was washed with $NaHCO_3$ (2 × 100 mL), brine (2 x 100 mL), dried ($MgSO_4$) and concentrated under reduced pressure to afford *tert*-butyl 4-(chloromethyl)-3,6-dihydropyridine-1(2*H*)-carboxylate (1.05 g, 39%) as a brown oil that was used in the next step without further purification.

^1H NMR data are consistent with those reported.[26]

^{1}H NMR (400 MHz, $CDCl_3$) δ = 5.84 (br s, 1H), 4.11 (s, 2H), 4.02 (br s, 2H), 3.62 (t, *J* = 5.6 Hz, 2H), 2.30 (br s, 2H), 1.57 (s, 9H) ppm.

^{13}C NMR (101 MHz, $CDCl_3$) δ = 154.7, 133.0, 123.4, 79.6, 48.6, 43.2, 39.4, 28.4, 26.0 ppm.

LRMS (EI$^+$) *m/z* (relative intensity): 233.2 (0.34%) $[M^{37}Cl]^{+\bullet}$, 231.2 (1.1%) $[M^{35}Cl]^{+\bullet}$, 176.1 (32%) $[M^{37}Cl–C_4H_8]^{+\bullet}$, 174.8 (79%) $[M^{35}Cl–C_4H_8]^{+\bullet}$, 56.9 (100%).

tert-Butyl 4-((2-bromo-4-cyanophenoxy)methyl)-3,6-dihydropyridine-1(2H)-carboxylate

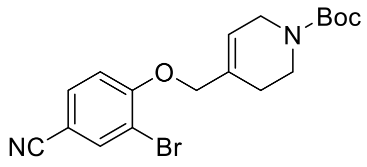

To a solution of 3-bromo-4-hydroxylbenzonitrile (1.03 g, 4.98 mmol) and *tert*-butyl 4-(chloromethyl)-3,6-dihydropyridine-1(2*H*)-carboxylate (1.05 g, 4.53 mmol) in acetone (100 mL) was added K_2CO_3 (1.88 g, 13.6 mmol). The resulting suspension was stirred for 60 h at 60 °C. The solution was then filtered and concentrated under reduced pressure to furnish a dark tan solid. Recrystallised from EtOH/H_2O afforded *tert*-butyl 4-((2-bromo-4-cyanophenoxy)methyl)-3,6-dihydropyridine-1(2*H*)-carboxylate (1.72 g, 97%) as tan crystals.

^1H NMR data are consistent with those reported.[26]

^{1}H NMR (400 MHz, $CDCl_3$, 65 °C) δ = 7.84 (d, *J* = 2.0 Hz, 1H), 7.56 (dd, *J* = 8.6, 2.0 Hz, 1H), 6.93 (d, *J*= 8.6 Hz, 1H), 5.85 (m, 1H), 4.57 (s, 2H), 3.97 (br d, *J* = 2.3 Hz, 2H), 3.57 (t, *J* = 5.8 Hz, 2H), 2.23 (m, 1H), 1.49 (s, 9H) ppm.

^{13}C NMR (101 MHz, $CDCl_3$, 65 °C) δ = 158.7, 154.9, 136.9, 132.9, 131.5, 123.1, 117.5, 113.4, 113.1, 105.9, 79.8, 72.6, 43.2, 40.2, 28.5, 25.8 ppm.

LRMS (ESI⁺) *m/z*: 337.3 [$M^{79}Br-C_4H_8+H]^+$, 339.3 [$M^{81}Br-C_4H_8+H]^+$

HRMS (ESI⁺) *m/z*: [$M^{79}Br+Na]^+$ Calcd for $C_{18}H_{21}{}^{79}BrN_2NaO_3$: 415.0628; found 415.0634.

IR ν_{max} (neat) 2928, 2230, 1675, 1595, 1450, 1410, 1238, 987, 818, 589 cm^{-1}.

mp: 135 – 136 °C.

***tert*-Butyl 5-cyano-2*H*-spiro[benzofuran-3,4'-piperidine]-1'-carboxylate (15)**

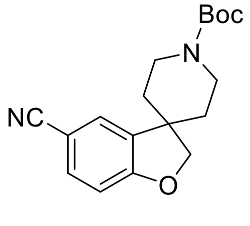

tert-Butyl 5-cyano-2*H*-spiro[benzofuran-3,4'-piperidine]-1'-carboxylate (**15**) was prepared by **General Method B** from *tert*-Butyl 4-((2-bromo-4-cyanophenoxy)methyl)-3,6-dihydropyridine-1(2*H*)-carboxylate (0.393 g, 1.00 mmol). The crude mixture was purified by flash chromatography (20% EtOAc in hexane) to afford the spiro carbamate **15** (0.113 g, 36%) as an amber solid.

^{1}H NMR data are consistent with those reported.[26]

^{1}H NMR (400 MHz, $CDCl_3$) δ = 7.47 (dd, *J* = 8.4, 1.8 Hz, 1H), 7.37 (d, *J* = 1.8 Hz, 1H), 6.86 (d, *J* = 8.4 Hz, 1H), 4.50 (s, 2H), 4.17 – 4.04 (m, 2H), 2.88 (br t, *J* = 12.2 Hz, 2H), 1.88 – 1.79 (m, 2H), 1.77 – 1.70 (m, 2H) 1.49 (s, 9H) ppm.

^{13}C NMR (101 MHz, $CDCl_3$) δ = 163.0, 154.6, 136.0, 133.9, 127.2, 119.3, 110.9, 104.0, 80.8, 80.0, 44.5, 40.7, 35.9, 28.4 ppm .

LRMS (ESI⁺) *m/z*: 315.4 $[M+H]^+$.

HRMS (ESI⁺) *m/z*: $[M+Na]^+$ Calcd for $C_{18}H_{22}N_2NaO_3$: 337.1523; found 337.1523.

IR ν_{max} (neat) 2976, 2932, 2222, 1683, 1484, 1422, 1242, 1148, 970, 730 cm^{-1}.

Cyclohex-1-en-1-ylmethanol

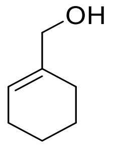

To a solution of methyl 1-cyclohexene-1-carboxylate (2.50 g, 17.8 mmol) in anhydrous CH_2Cl_2 (50 mL) at −78 °C was added DIBAL-H (36.0 mL of 1.0 M in CH_2Cl_2, 36.0 mmol). The reaction mixture was stirred at this temperature for 2 h before careful dropwise addition of MeOH (70 mL) followed by saturated aqueous Rochelle's salt (70 mL). The mixture was allowed to warm to rt, and stirring was continued for 10 h before the resulting layers were separated. The aqueous layer was extracted with EtOAc (2 × 50 mL). The combined organic extracts were washed with brine (50 mL), Rochelle's salt (50 mL), dried

(Na_2SO_4) and concentrated under reduced pressure. The crude mixture was purified by flash chromatography (11 – 20% Et_2O in hexane) to afford cyclohex-1-en-1-ylmethanol (1.83 g, 91%) as a colourless oil.

Spectroscopic data are consistent with those reported.[27,28]

^{1}H NMR (400 MHz, $CDCl_3$) δ = 5.67 (m, 1H), 3.97 (s, 2H), 2.06 – 1.97 (m, 4H), 1.69 – 1.55 (m, 4H) ppm.

^{13}C NMR (101 MHz, $CDCl_3$) δ = 137.5, 122.9, 67.6, 25.5, 24.9, 22.5, 22.4 ppm.

LRMS (ESI$^+$) *m/z*: 95.1 $[M–OH]^+$.

1-(Bromomethyl)cyclohex-1-ene

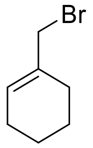

To a solution of cyclohex-1-en-1-ylmethanol (1.30 g, 11.6 mmol) in Et_2O (40 mL) at 0 °C was added PBr_3 (1.57 g, 5.80 mmol). The reaction mixture was allowed to warm to rt, and stirring was continued for 18 h. The mixture was poured into a solution of K_2CO_3 (1.59 g, 11.6 mmol) in H_2O (80 mL), and the phases were separated, re-extracting the aqueous layer with Et_2O (2 × 50 mL). The combined organic extracts were washed with brine (75 mL), dried (Na_2SO_4) and concentrated under reduced pressure to afford 1-(bromomethyl)cyclohex-1-ene (1.52 g, 75%) as a colourless oil that was used in the next step without further purification.

Spectroscopic data are consistent with those reported.[29,30]

^{1}H NMR (400 MHz, $CDCl_3$) δ = 5.89 (m, 1H), 3.95 (s, 2H), 2.17 – 2.10 (m, 2H), 2.08 – 2.01 (m, 2H), 1.72 – 1.64 (m, 2H), 1.61 – 1.54 (m, 2H) ppm.

^{13}C NMR (101 MHz, $CDCl_3$) δ = 134.6, 128.1, 39.9, 26.3, 25.4, 22.4, 21.8 ppm.

LRMS (EI$^+$) *m/z* (relative intensity): 175.8 (56%) $[M^{81}Br]^{+\bullet}$, 173.8 (51%) $[M^{79}Br]^{+\bullet}$, 95.1 (100%).

2-Bromo-1-(cyclohex-1-en-1-ylmethoxy)-4-methoxybenzene

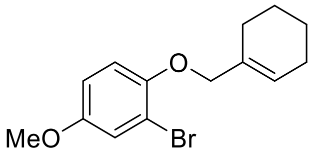

2-Bromo-1-(cyclohex-1-en-1-ylmethoxy)-4-methoxybenzene was prepared by **General Method C** from 2-bromo-4-methoxyphenol (0.406 g, 2.00 mmol) and 1-(bromomethyl)cyclohex-1-ene (0.350 g, 2.00 mmol). The title ether (0.584 g, 98%) was obtained as a colourless oil that was used in the next step without further purification.

^{1}H NMR (400 MHz, $CDCl_3$) δ = 7.12 (d, *J* = 2.9 Hz, 1H), 6.86 (d, *J* = 9.0 Hz, 1H), 6.79 (dd, *J* = 9.0, 2.9 Hz, 1H), 5.83 (m, 1H), 4.38 (s, 2H), 3.76 (s, 3H), 2.16 – 2.10 (m, 2H), 2.10 – 2.04 (m, 2H), 1.73 – 1.66 (m, 2H), 1.65 – 1.58 (m, 2H) ppm.

^{13}C NMR (101 MHz, $CDCl_3$) δ = 154.1, 149.6, 133.6, 125.6, 118.7, 115.2, 113.6, 113.0, 74.7, 55.9, 25.7, 25.0, 22.4, 22.3 ppm.

HRMS (ESI$^+$) *m/z*: [M^{79}Br+Na]$^+$ Calcd for $C_{14}H_{17}{}^{79}Br\ NaO_2$ 319.0304; found 319.0313

IR ν_{max} (neat) 2927, 1488, 1272, 1209, 1035, 799, 731 cm^{-1}

5-Methoxy-2*H*-spiro[benzofuran-3,1'-cyclohexane] (16)

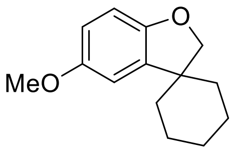

5-Methoxy-2*H*-spiro[benzofuran-3,1'-cyclohexane] (**16**) was prepared by **General Method B** from 2-bromo-1-(cyclohex-1-en-1-ylmethoxy)-4-methoxybenzene (0.297 g, 1.00 mmol). The crude mixture was purified by flash chromatography (25% CH_2Cl_2 in hexane) to afford the title spirocycle **16** (0.102 g, 47%) as a colourless oil.

^{1}H NMR (400 MHz, $CDCl_3$) δ = 6.72 – 6.64 (m, 3H), 4.35 (s, 2H), 3.77 (s, 3H), 1.83 – 1.61 (m, 7H), 1.42 – 1.27 (m, 3H) ppm.

^{13}C NMR (101 MHz, $CDCl_3$) δ = 154.1, 153.3, 137.4, 112.5, 109.5, 109.4, 81.2, 56.0, 46.5, 36.5, 25.4, 23.2 ppm.

LRMS (ESI$^+$) *m/z*: 219.2 [M+H]$^+$

HRMS (ESI$^+$) *m/z*: [M+H]$^+$ Calcd for $C_{14}H_{195}O_2$ 219.1380; found 219.1379

IR ν_{max} (neat) 2925, 1487, 1199, 1029, 980, 802, 766 cm^{-1}.

3-Bromo-4-(cyclohex-1-en-1-ylmethoxy)benzonitrile

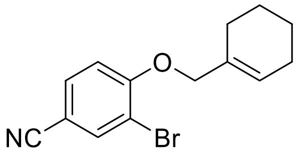

3-Bromo-4-(cyclohex-1-en-1-ylmethoxy)benzonitrile was prepared by **General Method C** from 3-bromo-4-hydroxybenzonitrile (0.396 g, 2.00 mmol) and 1-(bromomethyl)cyclohex-1-ene (0.350 g, 2.00 mmol). The title ether (0.582 g, 99%) was obtained as a colourless oil that was used in the next step without further purification.

^{1}H NMR (400 MHz, $CDCl_3$) δ = 7.83 (d, *J* = 2.0 Hz, 1H), 7.56 (dd, *J* = 8.6, 2.1 Hz, 1H), 6.94 (d, *J* = 8.6 Hz, 1H), 5.87 (m, 1H), 4.51 (s, 2H), 2.12 – 2.05 (m, 4H), 1.74 – 1.59 ppm (m, 4H) ppm.

^{13}**C NMR** (101 MHz, $CDCl_3$) δ = 158.8, 136.7, 132.9, 132.2, 126.6, 117.9, 113.2, 112.8, 105.0, 73.9, 25.5, 25.0, 22.2, 22.1 ppm.

LRMS (ESI$^+$) *m/z*: 316.2 [$M^{81}Br+Na$]$^+$

HRMS (ESI$^+$) *m/z*: [M+Na]$^+$ Calcd for $C_{14}H_{14}BrNNaO$ 314.0151; found 314.0156

IR ν_{max} (neat) 2928, 2226, 1594, 1490, 1258, 982, 811, 588 cm^{-1}

2*H*-Spiro[benzofuran-3,1'-cyclohexane]-5-carbonitrile (17)

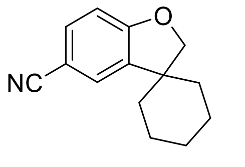

2*H*-Spiro[benzofuran-3,1'-cyclohexane]-5-carbonitrile (**17**) was prepared by **General Method B** from 3-bromo-4-(cyclohex-1-en-1-ylmethoxy)benzonitrile (0.292 g, 1.00 mmol). The crude mixture was purified by flash chromatography (25% Et_2O in hexane) to afford the title spirocycle **17** (0.096 g, 45%) as a colourless solid.

^{1}**H NMR** (400 MHz, $CDCl_3$) δ = 7.45 (dd, *J* = 8.3, 1.8 Hz, 1H), 7.38 (d, *J* = 1.8 Hz, 1H), 6.82 (d, *J* = 8.3 Hz, 1H), 4.45 (s, 2H), 1.82 – 1.76 (m, 5H), 1.68 – 1.58 (m, 2H), 1.41 – 1.31 (m, 3H) ppm.

^{13}**C NMR** (101 MHz, $CDCl_3$) δ = 163.10, 137.85, 133.51, 127.25, 119.71, 110.58, 103.56, 82.07, 45.94, 36.72, 25.12, 23.02 ppm.

LRMS (ESI$^+$) *m/z*: 214.3 [M+H]$^+$

HRMS (ESI$^+$) *m/z*: [M+H]$^+$ Calcd for $C_{14}H_{15}NO$ 214.1226; found 214.1229

IR ν_{max} (neat) 2926, 2219, 1608, 1485, 1245, 973, 824 cm^{-1}

mp: 111.2 – 112.0 °C

1-Iodo-2-(oct-1-en-3-yloxy)benzene

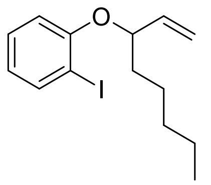

Procedure adapted from literature:[31] A solution of 2-iodophenol (3.30 g, 15 mmol), 1-octen-3-ol (1.90 g, 15 mmol), PPh3 4.72 g, 18 mmol) in THF (4 mL) was sonicated (40 KHz) until well mixed. Whilst sonicating, DIAD (3.64 g, 18 mmol) was added dropwise over 2 minutes and the reaction mixture was sonicated for a further 15 mintues and then triturated with cold hexane (10 mL). The crude mixture was then purified by flash column chromatography (100% hexane) to furnish the title product (1.215 g, 3.67 mmol, 25%) as a colourless oil.

^{1}H NMR (400 MHz, $CDCl_3$) δ = 7.77 (dd, *J* = 1.7, 7.8 Hz, 1H), 7.24 (ddd, *J* = 1.6, 7.3, 8.2 Hz, 1H), 6.81 (dd, *J* = 1.3, 8.4 Hz, 1H), 6.68 (dt, *J* = 1.4, 7.5 Hz, 1H), 5.89 (ddd, *J* = 6.2, 10.6, 17.4 Hz, 1H), 5.26 (td, *J* = 1.3, 17.4 Hz, 1H), 5.22 (td, *J* = 1.2, 10.5 Hz, 1H), 4.65 (q, *J* = 5.9 Hz, 1H), 1.88 (dddd, *J* = 5.3, 7.0, 10.4, 13.6 Hz, 1H), 1.73 (tdd, *J* = 5.7, 10.0, 13.6 Hz, 1H), 1.62 – 1.42 (m, 2H), 1.41 – 1.29 (m, 4H), 0.96 – 0.86 (m, 3H) ppm.

^{13}C NMR (101 MHz, $CDCl_3$) δ = 156.9, 139.4, 137.6, 129.1, 122.4, 116.6, 114.3, 87.6, 80.5, 35.5, 31.7, 24.9, 22.5, 14.0 ppm.

LRMS (EI$^+$) *m/z* (relative intensity): 330.1 (40%) $[M]^{+\bullet}$, 232.9 (100%) $[C_7H_6IO]^{+\bullet}$.

HRMS (EI$^+$) *m/z*: $[M]^{+\bullet}$ Calcd for $C_{14}H_{19}IO$: 330.0475; found 330.0476.

IR ν_{max} (neat) 2928, 2858, 1468, 1241, 924 cm^{-1}.

3-Methyl-2-pentyl-2,3-dihydrobenzofuran (18, trans:cis ~ 88:12)

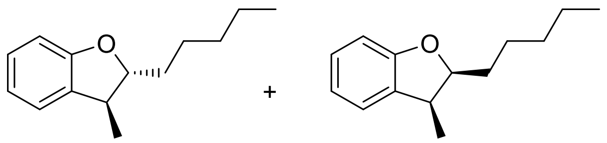

dr: ~ 82:12 (^{1}H NMR)

3-Methyl-2-pentyl-2,3-dihydrobenzofuran (**18**) was prepared by **General Method B** from 1-iodo-2-(oct-1-en-3-yloxy)benzene (0.330 g, 1.00 mmol). The crude mixture was purified by flash column chromatography (100% hexane) to afford 3-methyl-2-pentyl-2,3-dihydrobenzofuran (**18**, 0.125 g, 61%) as a mixture of diastereoisomers (trans:cis ~ 88:12 by ^{1}H NMR)[44,45] as a colourless oil.

^{1}H NMR (400 MHz, $CDCl_3$) Spectra presented as a mixture of diastereoisomers (~ 88:12) δ = 7.18 – $7.10_{\text{maj and min}}$ (m, 2H), $6.87_{\text{maj and min}}$ (dt, *J* = 0.9, 7.4 Hz, 1H), $6.79_{\text{maj and min}}$ (m, 1H), 4.65_{min} (ddd, *J* = 4.4, 8.1, 9.0 Hz, 1H), 4.26_{maj} (dt, *J* = 5.1, 7.8 Hz, 1H), 3.39_{min} (quin, *J* = 7.3 Hz, 1H), 3.15_{maj} (br quin, *J* = 7.0 Hz, 1H), 1.87 – $1.68_{\text{maj and min}}$ (m, 2H), 1.65 – $1.44_{\text{maj and min}}$ (m, 2H), 1.43 – $1.29_{\text{maj and min}}$ (m, 7H), 1.17_{min} (d, *J* = 7.1 Hz, 3H), 0.99 - $0.90_{\text{maj and min}}$ (m, 3H) ppm.

^{13}C NMR (101 MHz, $CDCl_3$) δ = 159.2_{maj}, 158.7_{min}, 133.6_{min}, 132.4_{maj}, 127.9_{maj}, 127.9_{min}, 124.0_{min}, 123.7_{maj}, 120.3_{min}, 120.1_{maj}, 109.4_{min}, 109.3_{maj}, 91.2_{maj}, 86.6_{min}, 42.1_{maj}, 39.0_{min}, 35.1_{maj}, 31.9_{min}, 31.8_{maj}, 29.9_{min}, 26.2_{min}, 25.2_{maj}, 22.6_{maj}, 19.0_{maj}, 15.2_{min}, 14.0_{maj} ppm.

LRMS (EI$^+$) *m/z* (relative intensity): 204.2 (91%) $[M]^{+\bullet}$, 133.2 (100%) $[C_9H_9O]^{+\bullet}$.

HRMS (EI$^+$) *m/z*: $[M]^{+\bullet}$ Calcd for $C_{14}H_{20}O$: 204.1509; found 204.1510.

IR ν_{max} (neat) 2954, 2923, 2853, 1470, 1077 cm^{-1}.

(*E*)-1-(Dec-2-en-1-yloxy)-2-iodobenzene

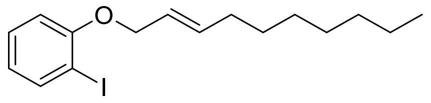

To a solution of *trans*-2-decenol (2.34 g, 15 mmol) and 2-iodophenol (3.30 g, 15 mmol) in THF (45 mL) was added PPh_3 (5.90g, 22.5 mmol) at 0 °C, followed by slow addition of DIAD (4.549 g, 22.5 mmol) over 20 min under N_2. The reaction mixture was allowed to warm up to rt and stirred for 16 hours. The reaction mixture was quenched with water (40 mL) and extracted with ethyl acetate (3 x 30 mL). The combined organic layers were washed with aq $Na_2S_2O_3$ (30 mL), brine (30 mL), dried over Na_2SO_4 and concentrated under reduced pressure. The crude mixture was then purified by flash chromatography (100% hexane) to furnish the title ether as a colourless oil (3.38 g, 9.43 mmol, 63%).

^{1}H NMR (400 MHz, $CDCl_3$) δ = 7.78 (dd, *J* = 7.8, 1.7 Hz, 1H), 7.28 (ddd, *J* = 8.2, 1.6, 7.5 Hz, 1H), 6.83 (dd, *J* = 8.3, 1.3 Hz, 1H), 6.71 (dt, *J* =7.6, 1.3 Hz, 1H), 5.89 (dtt, *J* = 15.0, 6.8, 1.3 Hz, 1H), 5.70 (dtt, *J* = 15.4, 1.4 Hz, 1H), 4.57 – 4.53 (m, 2H), 2.15 – 2.06 (m, 2H), 1.46 – 1.36 (m, 2H), 1.34 – 1.24 (m, 8H), 0.90 (t, *J* = 6.8 Hz, 3H) ppm.

^{13}C NMR (101 MHz, $CDCl_3$) δ = 157.3, 139.4, 135.5, 129.3, 124.2, 122.5, 112.8, 86.9, 69.9, 32.3, 31.8, 29.1, 29.1, 29.0, 22.6, 14.1 ppm.

LRMS (EI$^+$) *m/z* (relative intensity): 358.1 (10%) $[M]^{+\bullet}$, 132.1 (100%) $[C_9H_9O]^{+\bullet}$.

HRMS (EI$^+$) *m/z*: $[M]^{+\bullet}$ Calcd for $C_{16}H_{23}IO$: 358.0877; found 358.0787.

IR ν_{max} (neat) 2954, 2923, 2853, 1470, 1048, 744 cm^{-1}.

(*E*)-1-Chloro-2-(dec-2-en-1-yloxy)benzene

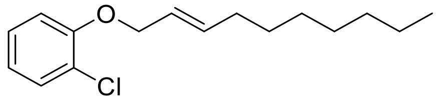

To a solution of *trans*-2-decenol (0.782 g, 0.93 mL, 5 mmol) and 2-chlorophenol (0.643 g, 0.52 mL, 5 mmol) in THF (15 mL) was added PPh_3 (1.97 g, 7.5 mmol) at 0 °C, followed by slow addition of DIAD (1.52 g, 1.48 mL, 7.5 mmol) over 20 min under N_2. The reaction mixture was allowed to warm up to rt and stirred for 16 hours. The solvent was evaporated under reduced pressure and the crude mixture was purified by flash chromatography (hexane) to afford (*E*)-1-chloro-2-(dec-2-en-1-yloxy)benzene (1.25 g, 94%) as a colourless oil.

^{1}H NMR (400 MHz, $CDCl_3$) δ = 7.36 (dd, *J* = 7.9, 1.5 Hz, 1H), 7.19 (ddd, *J* = 8.3, 7.5, 1.7 Hz, 1H), 6.93 (dd, *J* = 8.3, 1.5 Hz, 1H), 6.88 (ddd, *J* = 7.9, 7.5, 1.7 Hz, 1H), 5.86 (dtt, *J* = 15.6, 6.6, 1.2 Hz, 1H), 5.70 (dtt, *J* = 15.6, 5.9, 1.2 Hz, 1H), 4.56 (dd, *J* = 5.9, 1.2 Hz, 2H), 2.12 – 2.04 (m, 2H), 1.44 – 1.35 (m, 2H), 1.33 – 1.22 (m, 8H), 0.88 (t, *J* = 6.9 Hz, 3H) ppm.

^{13}C NMR (101 MHz, $CDCl_3$) δ = 154.3, 135.9, 130.3, 127.5, 124.2, 123.1, 121.3, 114.0, 69.9, 32.3, 31.8, 29.1, 29.1, 28.9, 22.6, 14.1 ppm.

LRMS (EI$^+$) *m/z* (relative intensity): 268.2 (8%) $[M^{37}Cl]^{+\bullet}$, 266.2 (25%) $[M^{35}Cl]^{+\bullet}$, 55.1 (100%).

IR ν_{max} (neat) 2981, 2889, 1382, 1265, 1151, 1072, 953, 709 cm^{-1}.

3-Octyl-2,3-dihydrobenzofuran (19)

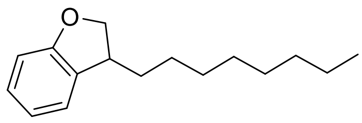

3-Octyl-2,3-dihydrobenzofuran (**19**) was prepared by **General Method B** from (*E*)-1-(dec-2-en-1-yloxy)-2-iodobenzene (0.358 g, 1.00 mmol). The crude mixture was purified by flash column chromatography (100% hexane) to afford ethyl 3-octyl-2,3-dihydrobenzofuran (**19**, 0.135 g, 58%) as a colourless oil.

3-Octyl-2,3-dihydrobenzofuran (**19**) was also prepared by **General Method B** from (*E*)-1-(dec-2-en-1-yloxy)-2-chlorobenzene (0.267 g, 1.00 mmol). The crude mixture was purified by flash column chromatography (100% hexane) to afford ethyl 3-octyl-2,3-dihydrobenzofuran (**19**, 0.078 g, 34%) as a colourless oil.

^{1}H NMR (400 MHz, $CDCl_3$) δ = 7.18 (br d, *J* = 7.3 Hz, 1H), 7.13 (br ddt, *J* = 0.7, 1.5, 7.8 Hz, 1H), 6.87 (dt, *J* = 1.0, 7.4 Hz, 1H), 6.80 (d, *J* = 8.1 Hz, 1H), 4.65 (t, *J* = 8.9 Hz, 1H), 4.22 (dd, *J* = 6.6, 8.7 Hz, 1H), 3.44 (m, 1H), 1.80 (m, 1H), 1.57 (m, 1H), 1.47 – 1.23 (m, 12H), 0.91 (t, *J* = 6.7 Hz, 3H) ppm.

^{13}C NMR (101 MHz, $CDCl_3$) δ = 159.9, 131.2, 128.0, 124.3, 120.2, 109.4, 76.9, 41.9, 34.9, 31.8, 29.7, 29.5, 29.3, 27.2, 22.6, 14.1 ppm.

LRMS (EI$^+$) *m/z* (relative intensity): 232.2 (10%) $[M]^{+\bullet}$, 118.9 (100%) $[C_8H_7O]^{+\bullet}$.

HRMS (EI$^+$) *m/z*: $[M]^{+\bullet}$ Calcd for $C_{16}H_{24}O$: 232.1822; found 232.1821.

IR ν_{max} (neat) 2923, 2853, 1612, 1481, 747 cm^{-1}.

1-(But-3-en-1-yloxy)-2-iodobenzene

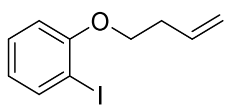

1-(But-3-en-1-yloxy)-2-iodobenzene was prepared by **General Method C** from 2-iodophenol (2.20 g, 10.0 mmol) and 4-bromobut-1-ene (1.39 g, 10.3 mmol). The crude mixture was purified by flash chromatography (petroleum ether) to afford 1-(but-3-en-1-yloxy)-2-iodobenzene (1.87 g, 68%) as a pale yellow oil.

Spectroscopic data are consistent with those reported.[32]

^{1}H NMR (400 MHz, $CDCl_3$) δ = 7.78 (dd, *J* = 7.6, 1.6 Hz, 1H), 7.29 (ddd, *J* = 8.2, 7.6, 1.6 Hz, 1H), 6.81 (dd, *J* = 8.2, 1.3 Hz, 1H), 6.71 (td, *J* = 7.6, 1.3 Hz, 1H), 5.99 (ddt, *J* = 17.2, 10.2, 6.7 Hz, 1H), 5.22 (dq, *J* = 17.2, 1.6 Hz, 1H), 5.15 (ddt, *J* = 10.2, 1.9, 1.2 Hz, 1H), 4.07 (t, *J* = 6.6 Hz, 2H), 2.62 (qt, *J* = 6.7, 1.3 Hz, 2H) ppm.

^{13}C NMR (101 MHz, $CDCl_3$) δ = 157.4, 139.4, 134.3, 129.4, 122.5, 117.3, 112.2, 86.7, 68.6, 33.6 ppm.

LRMS (EI$^+$) *m/z* (relative intensitiy): $[M]^{+\bullet}$ 274.0 (100%).

4-Methylchromane (20)

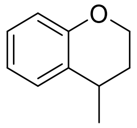

4-Methylchromane (**20**) was prepared by **General Method A** from 1-(but-3-en-1-yloxy)-2-iodobenzene (0.274 g, 1.00 mmol). The crude mixture was purified by flash chromatography (10% toluene in hexane to Et_2O:toluene:hexane = 5:10:85) to afford 4-methylchromane (**20**, 0.123 g, 83%) as a colourless oil.

4-Methylchromane (**20**) was also prepared by **General Method B** from 1-(but-3-en-1-yloxy)-2-iodobenzene (0.277 g, 1.01 mmol). The crude mixture was purified by flash chromatography (10 – 20% CH_2Cl_2 in hexane) to afford 4-methylchromane (**20**, 0.065 g, 43%) as a colourless oil.

Spectroscopic data are consistent with those reported.[32]

^{1}H NMR (400 MHz, $CDCl_3$) δ = 7.17 (d, *J* = 7.6 Hz, 1H), 7.10 (td, *J* = 7.6, 1.3 Hz, 1H), 6.88 (td, *J* = 7.6, 1.2 Hz, 1H) 6.80 (dd, *J* = 7.6, 1.3 Hz, 1H), 4.26 - 4.14 (m, 2H), 2.97 (sxt, *J* = 6.8 Hz, 1H), 2.10 (m, 1H), 1.74 (dtd, *J* = 13.7, 6.8, 3.4 Hz, 1H), 1.35 (d, *J* = 6.8 Hz, 3H) ppm.

^{13}C NMR (101 MHz, $CDCl_3$) δ = 154.3, 128.6, 127.6, 127.2, 120.2, 116.7, 63.8, 30.3, 28.5, 22.2 ppm.

LRMS (EI$^+$) *m/z* (relative intensity): 148.1 (82%) $[M]^{+\bullet}$, 133.0 (100%).

1,2-Di(chroman-4-yl)ethane

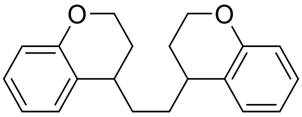

1,2-Di(chroman-4-yl)ethane was obtained as a byproduct from the reductive cyclisation of 1-(but-3-en-1-yloxy)-2-iodobenzene (0.274 g, 1.00 mmol, **General Method A**). The crude mixture was purified by flash chromatography (10% Et_2O in hexane to Et_2O:toluene:hexane = 5:10:85) to afford 1,2-di(chroman-4-yl)ethane (0.009 g, 6%) as a colourless oil.

^{1}H NMR (400 MHz, $CDCl_3$) δ = 7.17 – 7.08 (m, 4H), 6.91 – 6.84 (m, 2H), 6.81 (dd, *J* = 8.1, 1.1 Hz, 2H), 4.26 – 4.14 (m, 4H), 2.91 – 2.78 (m, 2H), 2.16 – 2.05 (m, 2H), 2.00 – 1.80 (m, 4H), 1.75 – 1.61 (m, 2H) ppm.

^{13}C NMR (101 MHz, $CDCl_3$) δ = 154.5, 154.5, 129.0, 128.9, 127.4, 127.4, 126.3, 126.2, 120.2, 120.1, 116.9, 116.9, 63.5, 63.5, 34.0, 33.8, 33.6, 33.4, 27.1, 26.8 ppm.

LRMS (EI$^+$) *m/z* (relative intensity): 294.1 (56%) $[M]^{+\bullet}$, 133.0 (100%).

HRMS (EI$^+$) *m/z*: $[M]^{+\bullet}$ Calcd for $C_{20}H_{22}O_2$: 294.1614; found 294.1616.

IR ν_{max} (neat) 2923, 2876, 1594, 1482, 1450, 1219, 1015, 751 cm^{-1}.

mp: 153 – 155 °C

3-(But-3-en-1-yloxy)-2-iodopyridine

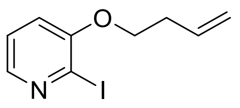

3-(But-3-en-1-yloxy)-2-iodopyridine was prepared by **General Method C** from 3-hydroxy-2-iodopyridine (2.21 g, 10.0 mmol) and 4-bromobut-1-ene (1.49 g, 12.0 mmol). The crude mixture was purified by flash column chromatography (10% EtOAc in hexane) to afford 3-(but-3-en-1-yloxy)-2-iodopyridine (2.54 g, 92%) as a colourless oil.

Spectroscopic data (^{1}H NMR and LRMS) are consistent with those reported.[23]

^{1}H NMR (400 MHz, $CDCl_3$) δ = 7.95 (m, 1H), 7.15 (m, 1H), 6.95 (m, 1H), 5.93 (m, 1H), 5.24 – 5.10 (m, 2H), 4.09 – 4.00 (m, 2H), 2.63 – 2.55 (m, 2H) ppm.

^{13}C NMR (101 MHz, $CDCl_3$) δ = 154.5, 142.5, 133.7, 123.3, 117.8, 117.7, 112.3, 68.7, 33.3 ppm.

LRMS (ESI$^+$) *m/z*: 276.2 $[M+H]^+$.

HRMS (ESI$^+$) *m/z*: $[M+H]^+$ Calcd for $C_9H_{11}INO$: 275.9880; found 275.9886.

IR ν_{max} (neat) 2977, 1680, 1348, 1321, 799 cm^{-1}.

4-Methyl-3,4-dihydro-2*H*-pyrano[3,2-*b*]pyridine (21)

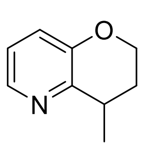

4-Methyl-3,4-dihydro-2*H*-pyrano[3,2-*b*]pyridine (**21**) was prepared by **General Method A** from 3-(but-3-en-1-yloxy)-2-iodopyridine (0.275 g, 1.00 mmol). The crude mixture was purified by flash chromatography (7% EtOAc in hexane) to afford 4-methyl-3,4-dihydro-2*H*-pyrano[3,2-*b*]pyridine (**21**, 0.067 g, 45%) as a pale green oil.

Spectroscopic data are consistent with those reported (^{1}H NMR and LRMS).[23]

^{1}H NMR (400 MHz, $CDCl_3$) δ = 8.16 (dd, *J* = 4.5, 1.5 Hz, 1H), 7.10 – 7.01 (m, 2H), 4.26 – 4.16 (m, 2H), 3.08 (sxt, *J* = 6.7 Hz, 1H), 2.21 (dddd, *J* = 13.8, 7.5, 5.9, 3.8 Hz, 1H), 1.85 (tdd, *J* = 10.3, 6.5, 3.5 Hz, 1H), 1.43 (d, *J* = 7.1 Hz, 3H) ppm.

^{13}C NMR (101 MHz, $CDCl_3$) δ = 151.0, 148.0, 141.5, 123.7, 122.4, 64.0, 31.8, 30.1, 20.4 ppm.

LRMS (EI$^+$) *m/z* (relative intensity): 149.1 (78%) $[M]^{+\bullet}$; 119.9 (100%).

Ethyl (*E*)-5-(2-bromophenyl)pent-2-enoate

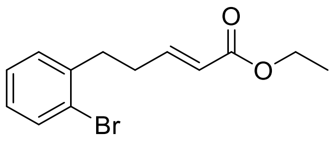

3-(2-Bromophenyl)propanal (1.00 g, 4.69 mmol) and (carbethoxymethlene)triphenylphosporane (1.63 g, 4.69 mmol) were dissolved in anhydrous toluene (20 mL), and the suspension heated under reflux for 8 h. After removal of solvent under reduced pressure, the residue was suspended in a 4:1 hexane-ether mixture (20 mL), and passed through a plug of silica, washing with the same eluent (10 mL x 2). The combined filtrate was concentrated and the crude mixture was purified by flash chromatography (8% EtOAc in hexane) to afford ethyl (*E*)-5-(2-bromophenyl)pent-2-enoate (1.19 g , 90%) as a colourless oil.

Spectroscopic data (^{1}H NMR) are consistent with those reported.[33]

^{1}H NMR (400 MHz, $CDCl_3$) δ = 7.55 (d, *J* = 7.7 Hz, 1H), 7.27 – 7.19 (m, 2H), 7.11 – 6.98 (m, 1H). 7.03 (d, *J* = 15.6 Hz, 1H), 5.87 (dt, *J* = 15.6, 1.5 Hz, 1H), 4.20 (q, *J* = 7.2 Hz, 2H), 2.90 (t, *J* = 7.5 Hz, 2H), 2.56 – 2.49 (m, 2H), 1.30 (t, *J* = 7.2 Hz, 3H) ppm.

^{13}C NMR (101 MHz, $CDCl_3$) δ = 166.5, 147.5, 140.0, 132.9, 130.3, 127.9, 127.5, 124.3, 122.0, 60.2, 34.7, 32.2, 14.2 ppm.

LRMS (ESI$^+$) *m/z*: 283.2 $[M^{79}Br+H]^+$, 285.2 $[M^{81}Br+H]^+$

HRMS (ESI$^+$) *m/z*: $[M^{79}Br+H]^+$ Calcd for $C_{13}H_{16}{}^{79}BrO_2$: 283,0328; found 283.0328

IR ν_{max} (neat) 2980, 1715, 1653, 1391, 1264, 1071, 749 cm^{-1}

Ethyl 2-(2,3-dihydro-1*H*-inden-1-yl)acetate (22)

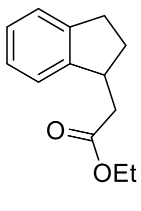

Ethyl 2-(2,3-dihydro-1*H*-inden-1-yl)acetate (**22**) was prepared by **General Method B** from ethyl (*E*)-5-(2-bromophenyl)pent-2-enoate (0.283 g, 1.00 mmol). The crude mixture was purified by flash column

chromatography (5% Et_2O in hexane) to afford ethyl 2-(2,3-dihydro-1*H*-inden-1-yl)acetate (**22**, 0.126 g, 62%) as a pale yellow oil.

Spectroscopic data are consistent with those reported.[34]

^{1}H NMR (400 MHz, $CDCl_3$) δ = 7.23 (m, 1H), 7.21 – 7.15 (m, 3H), 4.20 (q, *J* = 7.2 Hz, 2H), 3.60 (m, 1H), 3.00 – 2.83 (m, 2H), 2.79 (dd, *J* = 15.4, 5.6 Hz, 1H), 2.45 (dd, *J* = 15.4, 9.1 Hz, 1H), 2.40 (m, 1H), 1.76 (m, 1H), 1.30 ppm (t, *J* = 7.2 Hz, 3H) ppm.

^{13}C NMR (101 MHz, $CDCl_3$) δ = 172.8, 145.7, 143.9, 126.7, 126.2, 124.6, 123.4, 60.4, 41.3, 39.9, 32.3, 31.2, 14.3 ppm.

LRMS (ESI$^+$) *m/z*: 205.2 $[M+H]^+$

1-(6-Bromobenzo[*d*][1,3]dioxol-5-yl)-2,2-dimethylbut-3-en-1-ol

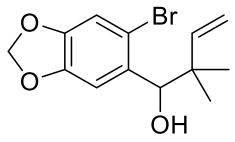

To a solution of 6-bromopiperonal (3.44 g, 15 mmol) and prenyl bromide (3.35 g, 22.5 mmol) in sat. NH_4Cl/THF (100 mL, 75:25) was added zinc powder (1.96 g, 30 mmol) at 0 °C. The resulting suspension was stirred for 30 min, then allowed to warm to rt and stirring was continued for 16 h. The reaction was quenched with 1 M HCl (15 mL) and the layers separated. The aqueous layer was extracted with Et_2O (3 × 50 mL). The combined organic extracts were washed with water (3 × 50 mL), brine (50 mL), dried ($MgSO_4$) and concentrated under reduced pressure. The crude mixture was purified by flash chromatography (10% Et_2O in hexane) to afford the title alcohol (3.28 g, 73%) as a brown syrup.

^{1}H NMR (400 MHz, $CDCl_3$) δ = 6.97 (2 x s, 2H), 6.02 (dd, *J* = 17.6, 10.8 Hz, 1H), 5.97 (brs, 2H), 5.16 (brd*, *J* = 10.8, 1H), 5.08 (brd*, *J* = 17.6, 1H), 4.96 (m, 1H), 1.12 (s, 3H), 1.03 (s, 3H) ppm.

^{13}C NMR (101 MHz, $CDCl_3$) δ = 147.5, 147.0, 144.6, 133.6, 114.5, 114.0, 112.1, 109.2, 101.7, 77.6, 43.4, 24.7, 21.2 ppm.

LRMS (ESI$^+$) *m/z*: 281.2 $[M^{79}Br–OH]^+$, 283.2 $[M^{81}Br–OH]^+$.

HRMS (ESI$^+$) *m/z*: $[M^{79}Br+Na]^+$ Calcd for $C_{13}H_{15}{}^{79}BrNaO_3$ 321.0097; found 321.0094.

IR ν_{max} (neat) 3439, 2968, 2902, 1636, 1472, 1230, 1036, 931, 693 cm^{-1}.

6,6,7-Trimethyl-6,7-dihydro-5*H*-indeno[5,6-*d*][1,3]dioxol-5-ol (23)

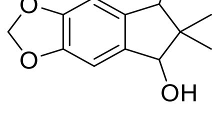

* Unresolved couplings

6,6,7-Trimethyl-6,7-dihydro-5*H*-indeno[5,6-*d*][1,3]dioxol-5-ol (**23**) was prepared by **General Method B** from 1-(6-bromobenzo[*d*][1,3]dioxol-5-yl)-2,2-dimethylbut-3-en-1-ol (0.299 g, 1.00 mmol). The crude mixture was purified by flash chromatography (20% Et_2O in hexane) to afford the title cyclopentanol **23** (0.087 g, 40%) as a dark green oil.

^{1}H NMR (400 MHz, $CDCl_3$) Spectrum collected from a mixture of diastereoisomers (~3:2) δ = 6.83_{maj} and 6.81_{min} (s, 1H), $6.62_{maj\ and\ min}$ (s, 1H), 5.94 – $5.90_{maj\ and\ min}$ (m, 2H), 4.59_{min} and 4.46_{maj} (s, 1H), 2.91_{maj} and 2.63_{min} (q, *J* = 7.1 Hz, 1H), 1.88_{min} and 1.74_{maj} (s, 1H), 1.18 – $1.09_{maj\ and\ min}$ (m, 6H), 0.86_{maj} and 0.72_{min} (s, 3H) ppm.

^{13}C NMR (101 MHz, $CDCl_3$) Spectrum collected from a mixture of diastereoisomers (~3:2) δ = 148.0, 147.4, 146.4, 146.3, 141.3, 139.0, 136.7, 136.3, 105.4, 104.6, 104.2, 104.0, 100.9, 100.8, 83.1, 82.8, 49.3, 46.8, 45.7, 45.6, 24.9, 21.7, 21.3, 15.0, 13.3, 13.2 ppm.

LRMS (ESI$^+$) *m/z*: 203.2 $[M–OH]^+$.

HRMS (ESI$^+$) *m/z*: $[M+H]^+$ Calcd for $C_{13}H_{17}O_3$ 221.1178; found 221.1123.

1-(6-Bromobenzo[*d*][1,3]dioxol-5-yl)-2,2-dimethylbut-3-en-1-one

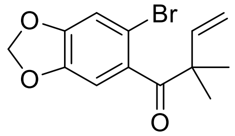

To a solution of 1-(6-bromobenzo[*d*][1,3]dioxol-5-yl)-2,2-dimethylbut-3-en-1-ol (898 mg, 3.00 mmol) in dry CH_2Cl_2 (20 mL) under Ar was added DMP (1.27 g, 3.00 mmol). The mixture was stirred at rt for 3 h, then quenched by the addition of sat. $NaHCO_3$ (10 mL) and $Na_2S_2O_3$ (10 mL). After 1 h brine (10 mL) and CH_2Cl_2 (10 mL) were added and the reaction mixture was filtered, and the layers separated. The aqueous layer was extracted with CH_2Cl_2 (3 × 20 mL). The combined organic extracts were dried ($MgSO_4$) and concentrated under reduced pressure to furnish the title aryl ketone as a dark green syrup (825 mg , 93%)

^{1}H NMR (400 MHz, $CDCl_3$) δ = 7.00 (s, 1H), 6.67 (s, 1H), 6.02 (dd, *J* = 17.5, 10.5 Hz, 1H), 6.01 (s, 2H), 5.19 (dd, *J* = 17.5, 0.7 Hz, 1H), 5.19 (dd, *J* = 10.5, 0.7 Hz, 1H), 1.36 (s, 6H) ppm.

^{13}C NMR (101 MHz, $CDCl_3$) δ = 207.7, 148.6, 146.6, 142.3, 135.3, 114.8, 113.2, 109.3, 106.9, 102.1, 51.4, 24.2 ppm.

LRMS (ESI$^+$) *m/z*: 297.2 $[M^{79}Br+H]^+$, 299.2 $[M^{81}Br+H]^+$.

HRMS (ESI$^+$) *m/z*: $[M^{79}Br+Na]^+$ Calcd for $C_{13}H_{13}BrNaO_3$ 318.9940; found 318.9943.

IR ν_{max} (neat) 2973, 1697, 1632, 1475, 1236, 1112, 1035, 857 cm^{-1}.

6,6,7-Trimethyl-6,7-dihydro-5*H*-indeno[5,6-*d*][1,3]dioxol-5-one (24)

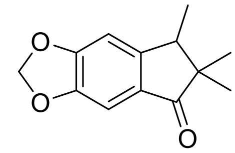

6,6,7-Trimethyl-6,7-dihydro-5*H*-indeno[5,6-*d*][1,3]dioxol-5-one (**24**) was prepared by **General Method B** from 1-(6-bromobenzo[*d*][1,3]dioxol-5-yl)-2,2-dimethylbut-3-en-1-one (0.297 g, 1.00 mmol). The crude mixture was purified by flash chromatography (20% Et_2O in hexane) to afford the title cyclopentanone **24** (0.083 g, 38%) as a dark green oil.

^{1}H NMR (400 MHz, $CDCl_3$) δ = 7.09 (s, 1H), 6.84 (s, 1H), 6.06 (s, 2H), 2.97 (q, *J* = 7.3 Hz, 1H), 1.25 (d, *J* = 7.3 Hz, 3H), 1.20 (s, 3H), 1.04 (s, 3H) ppm.

^{13}C NMR (101 MHz, $CDCl_3$) δ = 209.3, 154.8, 154.3, 148.2, 128.8, 104.4, 102.6, 102.1, 49.5, 44.5, 24.9, 21.0, 15.3 ppm.

LRMS (ESI$^+$) *m/z*: 219.2 $[M+H]^+$.

HRMS (ESI$^+$) *m/z*: $[M+H]^+$ Calcd for $C_{13}H_{15}O_3$ 219.1016; found 219.1014.

IR ν_{max} (neat) 2966, 2918, 1693, 1468, 1309, 1259, 1034, 937 cm^{-1}.

***tert*-Butyl (2-iodophenyl)carbamate**

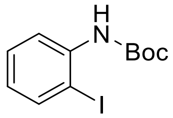

To a solution of 2-iodoanaline (6.00 g, 27.4 mmol) in THF (60 mL) was added di-*tert*-butyl dicarbonate (8.94 g, 41.1 mmol). The reaction mixture was refluxed for three days, then quenched with H_2O (45 mL). The solution was extracted with Et_2O (3 × 45 mL) and the combined organic extracts were dried ($MgSO_4$) and concentrated under reduced pressure. The crude mixture was purified by flash chromatography (1% EtOAc in petrol ether) to afford *tert*-butyl (2-iodophenyl)carbamate (5.93 g, 68%) as a colourless oil.

Spectroscopic data are consistent with those reported.[35,36]

^{1}H NMR (400 MHz, $CDCl_3$) δ = 8.06 (dd, *J* = 8.3, 1.4 Hz, 1H), 7.75 (dd, *J* = 8.0, 1.5 Hz, 1H), 7.32 (m, 1H), 6.83 (br s, 1H), 6.79 (ddd, *J* = 7.9, 7.3, 1.6 Hz, 1H), 1.55 (s, 9H) ppm.

^{13}C NMR (101 MHz, $CDCl_3$) δ = 152.6, 138.8, 129.2, 124.7, 120.2, 88.7, 81.1, 28.3 ppm.

LRMS (ESI$^+$) *m/z*: 305.2 $[M–C_4H_8+CH_3CN+H]^+$

tert-Butyl cyclohex-2-en-1-yl(2-iodophenyl)carbamate

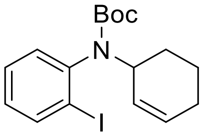

To a suspension of NaH (440 mg, 11.0 mmol) in THF (80 mL) at 0 °C was added *tert*-butyl (2-iodophenyl)carbamate (3.19 g, 10.0 mmol) dropwise (CAUTION: evolution of H_2 gas). The bath was removed, and after 2 h at rt, 3-bromocyclohexene (1.76 g, 11.0 mmol) was added dropwise and left to stir for 16 h. H_2O (50 mL) was added carefully to the reaction mixture, which was extracted with EtOAc (3 × 50 mL). The organic extracts were washed with water (3 × 50 mL), brine (50 mL), dried ($MgSO_4$) and concentrated under reduced pressure. The crude mixture was purified by flash chromatography (5% Et_2O in hexane) to afford *tert*-butyl cyclohex-2-en-1-yl(2-iodophenyl)carbamate (0.605 g, 15%) as a colourless solid.

^{1}H NMR (500 MHz, 363 K, DMSO-*d*$_6$) δ = 7.89 (m, 1H), 7.38 (td, *J* = 7.6, 1.4 Hz, 1H), 7.27 – 7.16 (m, 1H), 7.08 – 6.99 (m, 1H), 5.90 – 5.75 (m, 1H), 5.61 (s, 1H), 4.74 – 4.60 (br m, 1H), 2.16 – 1.72 (m, 4H), 1.63 – 1.45 (m, 2H), 1.36 (s, 9H) ppm.

^{13}C NMR (126 MHz, 363 K, DMSO-*d*$_6$) δ = 152.4, 138.7, 138.5, 129.8, 129.5, 129.3, 128.4, 128.3, 127.0, 27.6, 26.0, 23.7, 23.5, 20.5, 20.3 ppm.

LRMS (ESI$^+$) *m/z*: 344.2 $[M+H–C_4H_8]^+$

HRMS (ESI$^+$) *m/z*: $[M+Na]^+$ Calcd for $C_{17}H_{22}INNaO_2$: 422.0587; found 422.0593.

IR ν_{max} (neat) 2977, 1680, 1372, 1364, 1321, 799 cm^{-1}.

tert-Butyl 1,2,3,4,4a,9a-hexahydro-9*H*-carbazole-9-carboxylate (25)

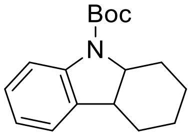

tert-Butyl 1,2,3,4,4a,9a-hexahydro-9*H*-carbazole-9-carboxylate (**25**) was prepared by **General Method A** from *tert*-butyl cyclohex-2-en-1-yl(2-iodophenyl)carbamate (0.399 g, 1.00 mmol). The crude mixture was purified by flash chromatography (10% EtOAc in hexane) to afford the title carbamate **25** (0.156 g, 57%) as a yellow oil.

tert-Butyl 1,2,3,4,4a,9a-hexahydro-9*H*-carbazole-9-carboxylate (**25**) was also prepared by **General Method B** from *tert*-butyl cyclohex-2-en-1-yl(2-iodophenyl)carbamate (0.399 g, 1.00 mmol). The crude mixture was purified by flash chromatography (10% EtOAc in hexane) to afford the title carbamate **25** (0.131 g, 48%) as a yellow oil.

^{1}H NMR (400 MHz, $CDCl_3$) δ = 7.74 (br s, 1H), 7.22 – 7.09 (m, 2H), 6.99 (td, *J* = 7.4, 0.9 Hz, 1H), 4.36 (br d, *J* = 4.0 Hz, 1H), 3.43 (br t, *J* = 6.5 Hz, 1H), 2.26 (m, 1H), 2.09 (m, 1H), 1.82 (m, 1H), 1.66 – 1.48 (m, 11H), 1.31 – 1.06 (m, 3H) ppm.

^{1}H NMR (500 MHz, 323 K, DMSO-d_6) δ = 7.60 (d, *J* = 7.7 Hz, 1H), 7.20 – 7.16 (m, 1H), 7.16 – 7.12 (m, 1H), 6.96 (td, *J* = 7.4, 1.0 Hz, 1H), 4.28 (ddd, *J* = 10.1, 8.1, 5.9 Hz, 1H), 3.38 (br t, *J* = 5.8 Hz, 1H), 2.22 – 2.15 (m, 1H), 2.03 – 1.97 (m, 1H), 1.78 (dddd, *J* = 14.2, 12.4, 5.9, 4.2 Hz, 1H), 1.53 – 1.48 (s, 11H), 1.24 – 0.97 (m, 3H).

^{13}C NMR (101 MHz, $CDCl_3$) δ = 152.2, 141.9, 133.7, 127.1, 122.5, 122.2, 115.4, 80.3, 60.3, 39.2, 28.4, 27.0, 24.0, 22.2, 20.9 ppm.

^{13}C NMR (126 MHz, 323 K, DMSO-d_6) δ = 151.2, 141.5, 133.3, 126.8, 122.5, 122.1, 114.6, 79.7, 59.6, 38.5, 27.8, 26.5, 23.2, 21.3, 20.4 ppm.

LRMS (ESI$^+$) *m/z*: 218.2 $[M+H–C_4H_8]^+$.

HRMS (ESI$^+$) *m/z*: $[M+Na]^+$ Calcd for $C_{17}H_{23}NNaO_2$: 296.1621; found 296.1618.

IR ν_{max} (neat) 3057, 2932, 2857, 1602, 1420, 1257, 937 cm^{-1}.

***tert*-Butyl but-3-en-1-yl(2-iodophenyl)carbamate**

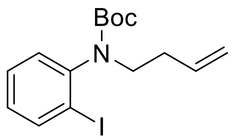

To a solution of *tert*-butyl (2-iodophenyl)carbamate (1.91 g, 6.00 mmol) in DMF (40 mL) was added NaH (288 mg, 7.20 mmol) at 0 °C (CAUTION: evolution of H_2 gas). After stirring for 30 min, homo-allyl bromide (0.73 mL, 7.20 mmol) was added, the mixture was allowed to warm to rt, and stirring was continued for 4 h. The reaction mixture was quenched by careful addition of H_2O (20 mL). The aqueous layer was extracted with Et_2O (3 × 20 mL), the combined organic layers were washed with H_2O (5 × 20 mL), brine (20 mL), dried ($MgSO_4$) and concentrated under reduced pressure. The crude mixture was purified by flash chromatography (5% EtOAc in hexane) to afford *tert*-butyl but-3-en-1-yl(2-iodophenyl)carbamate (0.977 g, 44%) as a yellow oil.

^{1}H NMR (500 MHz, 353 K, DMSO-d_6) δ = 7.91 (dd, *J* = 7.9, 1.5 Hz, 1H), 7.42 (ddd, *J* = 7.8, 7.4, 1.5 Hz, 1H), 7.26 (dd, *J* = 7.8, 1.6 Hz, 1H), 7.06 (ddd, *J* = 7.9, 7.4, 1.6 Hz, 1H), 5.79 (ddt, *J* = 17.2, 10.4, 6.7 Hz, 1H), 5.07 (dq, *J* = 17.2, 1.7 Hz, 1H), 5.02 (ddt, *J* = 10.4, 2.1, 1.1 Hz, 1H), 3.79 (m, 1H), 3.26 (m, 1H), 2.28 (quin, *J* = 7.3 Hz, 2H), 1.36 (br s, 9H) ppm.

^{13}C NMR (126 MHz, 353 K, DMSO-d_6) δ = 152.7, 143.9, 138.8, 135.2, 129.7, 128.6, 128.5, 115.9, 100.1, 79.0, 48.3, 32.0, 27.6 ppm.

LRMS (ESI$^+$) *m/z*: 318.2 $[M+H–C_4H_8]^+$.

HRMS (ESI$^+$) *m/z*: $[M+Na]^+$ Calcd for $C_{15}H_{20}INNaO_2$: 396.0431; found 396.0435.

IR ν_{max} (neat) 2987, 1681, 1366, 1346, 1296, 1148 cm^{-1}.

***tert*-Butyl 4-methyl-3,4-dihydroquinoline-1(2*H*)-carboxylate (26)**

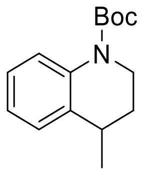

tert-Butyl 4-methyl-3,4-dihydroquinoline-1(2*H*)-carboxylate (**26**) was prepared by **General Method B** from *tert*-butyl but-3-en-1-yl(2-iodophenyl)carbamate (0.373 g, 1.00 mmol). The crude mixture was purified by flash chromatography (10% Et_2O in hexane) to afford the title carbamate **26** (0.095 g, 38%) as a colourless oil.

^{1}H NMR (500 MHz, 303 K, DMSO-d_6) δ = 7.53 (dd, *J* = 8.2, 1.0 Hz, 1H), 7.19 (d, *J* = 7.7 Hz, 1H), 7.11 (m, 1H), 7.00 (td, *J* = 7.4, 1.3 Hz, 1H), 3.68 – 3.57 (m, 2H), 2.85 (sxt, *J* = 6.3 Hz, 1H), 2.00 – 1.93 (m, 1H), 1.55 – 1.48 (m, 1H), 1.46 (s, 9H), 1.23 (d, *J* = 7.0 Hz, 3H) ppm.

^{13}C NMR (126 MHz, 303 K, DMSO-d_6) δ = 153.0, 137.5, 134.5, 126.9, 125.4, 123.8, 123.2, 80.0, 42.3, 31.1, 30.4, 27.9, 21.0 ppm.

LRMS (ESI$^+$) *m/z*: 192.3 $[M+H–C_4H_8]^+$.

HRMS (ESI$^+$) *m/z*: $[M+Na]^+$ Calcd for $C_{15}H_{21}NNaO_2$: 270.1465; found 270.1463.

IR ν_{max} (neat) 2971, 2930, 1691, 1365, 1141, 1044, 752 cm^{-1}.

***tert*-Butyl allyl(2-iodophenyl)carbamate**

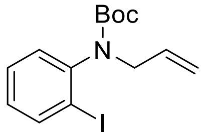

To a solution of *tert*-butyl (2-iodophenyl)carbamate (1.91 g, 6.00 mmol) in DMF (40 mL) was added NaH (288 mg, 7.20 mmol) at 0 °C and stirred for 30 min (CAUTION: evolution of H_2). Allyl bromide (0.62 mL, 7.20 mmol) was added, and the mixture was allowed to warm to rt, and stirring was continued for 4 h. The reaction mixture was quenched by the addition of water (20 mL). The aqueous layer was extracted with Et_2O (3 × 20 mL), the combined organic layers were washed with water (5 × 20 mL), brine (20 mL), dried ($MgSO_4$) and concentrated under reduced pressure to furnish the title *tert*-butyl allyl(2-iodophenyl)carbamate (1.86 g, 86%) as a yellow solid that was used in the next step without further purification.

Spectroscopic data previously reported in $CDCl_3$ (^{1}H NMR, ^{13}C NMR).[37]

^{1}H NMR (500 MHz, 353 K, DMSO-d_6): δ = 7.90 (dd, *J* = 7.9, 1.4 Hz, 1H), 7.40 (ddd, *J* = 7.8, 7.4, 1.5 Hz, 1H), 7.23 (dd, *J* = 7.9, 1.6 Hz, 1H), 7.05 (ddd, *J* = 7.9, 7.4, 1.6 Hz, 1H), 5.90 (ddt, *J* = 16.9, 10.5, 6.3 Hz, 1H), 5.12 – 5.06 (m, 2H), 4.35 (br d, *J* = 15.1 Hz, 1H), 3.80 (br d, *J* = 15.1 Hz, 1H), 1.36 (s, 9H) ppm.

^{13}C NMR (126 MHz, 353 K, DMSO-*d_6*): δ = 152.6, 143.8, 138.7, 133.4, 129.6, 128.5, 128.5, 117.0, 100.2, 79.2, 51.5, 27.6 ppm.

LRMS (ESI$^+$) *m/z*: 304.2 $[M+H–C_4H_8]^+$.

HRMS (ESI$^+$) *m/z*: $[M+Na]^+$ Calcd for $C_{14}H_{18}INNaO_2$: 382.0274; found 382.0280.

IR ν_{max} (neat) 3084, 2972, 1680, 1366, 1296, 1149, 780 cm^{-1}.

***tert*-Butyl 3-methylindoline-1-carboxylate (27)**

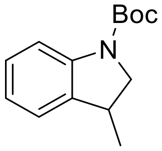

tert-Butyl 3-methylindoline-1-carboxylate (**27**) was prepared by **General Method B** from *tert*-butyl allyl(2-iodophenyl)carbamate (0.359 g, 1.00 mmol). The crude mixture was purified by flash chromatography (10% Et_2O in hexane) to afford the title carbamate **27** (0.150 g, 64%) as a colourless oil.

Spectroscopic data previously reported in $CDCl_3$ (^{1}H NMR, ^{13}C NMR).[38]

^{1}H NMR (400 MHz, DMSO-*d_6*): δ = 7.65 (br s, 1H), 7.20 (d, *J* = 7.5 Hz, 1H), 7.10 – 7.17 (m, 1H), 6.94 (td, *J* = 7.4, 1.0 Hz, 1H), 4.13 – 4.04 (m, 1H), 3.44 – 3.32 (m, 2H), 1.50 (s, 9H), 1.25 (d, *J* = 6.8 Hz, 3H) ppm.

^{13}C NMR (101 MHz, DMSO-*d_6*): δ = 151.7, 141.6, 136.3, 127.2, 123.8, 122.2, 113.9, 80.1, 55.2, 33.3, 28.0, 20.0 ppm.

^{1}H NMR (500 MHz, 353 K, DMSO-*d_6*) δ = 7.60 (d, *J* = 7.9 Hz, 1H), 7.20 – 7.17 (m, 1H), 7.14 (dddd, *J* = 8.1, 7.5, 1.4, 0.8 Hz, 1H), 6.96 – 6.92 (m, 1H), 4.10 (dd, *J* = 10.6, 9.3 Hz, 1H), 3.46 – 3.34 (m, 2H), 1.53 (s, 9H), 1.27 (d, *J* = 6.8 Hz, 3H) ppm.

^{13}C NMR (126 MHz, 353 K, DMSO-*d_6*) δ = 151.4, 141.4, 136.1, 126.8, 123.2, 121.7, 113.6, 79.8, 55.0, 33.0, 27.7, 19.5 ppm.

LRMS (ESI$^+$) *m/z*: 178.2 $[M+H–C_4H_8]^+$.

HRMS (ESI$^+$) *m/z*: $[M+Na]^+$ Calcd for $C_{14}H_{19}NNaO_2$: 256.1308; found 256.1306.

IR ν_{max} (neat) 2966, 1697, 1484, 1389, 1142, 747 cm^{-1}.

2-Iodo-*N*-methylaniline

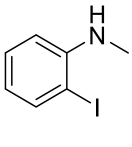

This compound was synthesised following a literature procedure.[39] To a solution of 2-iodoaniline (5.50 g, 25.0 mmol) in THF (50 mL) at –78 °C was added MeLi (1.1 M in diethyl ether solution, Br free,

22.9 mL, 25 mmol) dropwise over 1 hour and the mixture was stirred for 30 minutes. Iodomethane (2.00 mL, 33.0 mmol) in THF (10.0 mL) was added dropwise to the reaction mixture. The reaction mixture was stirred for 1 hour. The reaction mixture was allowed to warm to room temperature and stirred for 2 hours. After the reaction was completed, saturated aqueous NH_4Cl was added and the mixture was extracted with diethyl ether (3 x 60 mL). The combined organic layers were washed with brine and dried over Na_2SO_4. The solvent was then removed under reduced pressure and the residue was purified by flash column chromatography (1.6% EtOAc in hexane) to afford *N*-methyl-2-iodoaniline (5.65 g, 97%) as a pale yellow oil.

^{1}H NMR (400 MHz, $CDCl_3$) δ = 7.68 (m, 1H), 7.26 (m, 1H), 6.58 (m, 1H), 6.47 (m, 1H), 4.22 (s, 1H), 2.90 (s, 3H) ppm.

^{13}C NMR (101 MHz, $CDCl_3$) δ = 148.2, 138.9, 129.5, 118.5, 110.0, 85.1, 31.0 ppm.

LRMS (ESI$^+$) *m/z*: 234.1 $[M+H]^+$.

N-Allyl-2-iodo-N-methylaniline

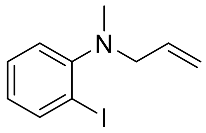

This compound was synthesised following a literature procedure.[40] To a solution of 2-Iodo-*N*-methylaniline (699 mg, 3.00 mmol) in EtOH (15.0 mL) was added Na_2CO_3 (334 mg, 3.15 mmol), and allyl bromide (363 mg, 3.00 mmol). The suspension was stirred at reflux for 14 h. The reaction mixture was quenched by addition of water (30 mL) and extracted with EtOAc (4 × 15 mL). The combined organic layers were washed with water (4 × 15 mL), brine (15 mL), dried over Na_2SO_4 and concentrated *in vacuo*. The crude mixture was purified by flash column chromatography (1% Et_2O in hexane) to afford the title aniline (637 mg, 2.69 mmol, 90%) as a colourless oil. Spectroscopic and physical data are consistent with the literature.

^{1}H NMR (400 MHz, $CDCl_3$) δ = 7.87 (dd, J = 7.8, 1.5 Hz, 1H), 7.31 (ddd, J = 8.0, 7.3, 1.5 Hz, 1H), 7.08 (dd, J = 8.0, 1.5 Hz, 1H), 6.79 (ddd, J = 7.8, 7.3, 1.5 Hz, 1H), 5.96 (ddt, J = 17.2, 10.2, 6.2 Hz, 1H), 5.26 (dq, J = 17.2, 1.6 Hz, 1H), 5.19 (dq, J = 10.2, 1.6 Hz, 1H), 3.57 (d, J = 6.2 Hz, 2H), 2.71 (s, 3H) ppm.

^{13}C NMR (101 MHz, $CDCl_3$) δ = 154.0, 140.1, 135.3, 128.8, 125.1, 121.8, 117.7, 98.3, 60.2, 41.0 ppm.

LRMS (ESI$^+$) *m/z*: 274.2 $[M+H]^+$.

1,3-Dimethylindoline (28)

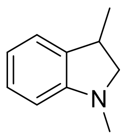

Modified procedure using THF/MeCN: A solution of *N*-allyl-2-iodo-*N*-methylaniline (1.911 g, 7.00 mmol), phenanthrene (0.062 g, 0.35 mmol) and *n*-Bu_4NI (1.294 g, 3.50 mmol) in THF/MeCN (280 mL, 7:1, 0.025 M) was sonicated for 5 min. The solution was pumped through the Ammonite 8 reactor (glassy carbon anode, stainless steel cathode; internal volume = 1 mL) with a fixed flow rate of 2.0 mL min^{-1} and an applied current of 0.24 A (3.0 F). The effluent solution was collected and solvent removed under reduced pressure. The crude mixture was purified by flash column chromatography (1% EtOAc in hexane) to afford 1,3-dimethylindoline (**28**, 0.435 g, 42%) as a colourless oil.

Spectroscopic data are consistent with those reported.[15]

^{1}H NMR (400 MHz, $CDCl_3$): δ = 7.12 (m, 1H), 7.08 (m, 1H), 6.73 (td, *J* = 7.4, 0.9 Hz, 1H), 6.52 (d, *J* = 7.8 Hz, 1H), 3.55 (t, *J* = 8.4 Hz, 1H), 3.30 (m, 1H), 2.82 (t, *J* = 8.6 Hz, 1H), 2.77 (s, 3H), 1.33 (d, *J* = 6.8 Hz, 3H) ppm.

^{13}C NMR (101 MHz, $CDCl_3$): δ = 152.9, 135.3, 127.4, 122.9, 117.9, 107.4, 64.1, 36.2, 35.3, 18.2 ppm.

LRMS (EI$^+$) *m/z* (relative intensity): 147.1 (89%) $[M]^{+•}$, 132.1 (100%) $[M–Me]^{+•}$.

Ethyl (*E*)-3-cyclopropylacrylate

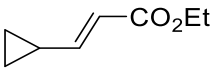

Ethyl (*E*)-3-cyclopropylacrylate was prepared following a literature procedure:[41] To a solution of triethyl phoshonoacetate (3.57 mL, 18.0 mmol) in dry THF (60 mL) was added NaH (0.720 g, 18.0 mmol) at 0 °C (CAUTION: liberation of H_2), and the mixture was stirred for 30 min at rt. Then, cyclopropane-carboxaldehyde (1.12 mL, 15.0 mmol) was slowly added at 0 °C, and the mixture was stirred at rt for 2 h. The reaction was carefully quenched with water and the solvent (THF) was evaporated under reduced pressure. The crude was extracted with EtOAc (3 x 20 mL). The combined organic layers were washed with brine (1x 30 mL), dried ($MgSO_4$) and concentrated under reduced pressure. The crude mixture was purified by flash chromatography (hexane:EtOAc = 9:1) to afford ethyl (*E*)-3-cyclopropylacrylate (2.03 g, 97%) as a colourless oil.

Spectroscopic data (^{1}H NMR) are consistent with those reported.[42]

^{1}H NMR (400 MHz, $CDCl_3$) δ = 6.43 (dd, *J* = 15.4, 10.0 Hz, 1H), 5.89 (d, *J* = 15.4 Hz, 1H), 4.18 (q, *J* = 7.1 Hz, 2H), 1.57 (m, 1H), 1.28 (t, *J* = 7.1 Hz, 3H), 0.98 – 0.91 (m, 2H), 0.67 – 0.61 (m, 2H) ppm.

^{13}C NMR (101 MHz, $CDCl_3$) δ = 166.8, 153.9, 118.2, 60.0, 14.3, 14.3, 8.6 ppm.

LRMS (EI$^+$) *m/z* (relative intensity): 140.1 (6%) $[M]^{+•}$, 67.0 (100%).

HRMS (EI$^+$) *m/z*: $[M]^{+•}$ Calcd for $C_8H_{12}O_2$: 140.0837; found 140.0832.

IR $ν_{max}$ (neat) 2980, 1711, 1644, 1377, 1264, 1144, 1036, 942, 810 cm^{-1}.

(*E*)-3-Cyclopropylprop-2-en-1-ol

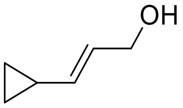

(*E*)-3-Cyclopropylprop-2-en-1-ol was prepared following a literature procedure:[41] To a solution of ethyl (*E*)-3-cyclopropylacrylate (1.96 g, 14.0 mmol) in dry Et_2O (28.0 mL) was added a solution of DIBAL (30.8 mL of 1.0 M, 30.8 mmol) at 0 °C and the mixture was stirred for 1 h at that temperature. The mixture was allowed to warm to rt and stirring was continued for 2 h. The reaction was quenched by careful addition of 1 N HCl at 0 °C. The organic layer was separated, washed with brine, dried ($MgSO_4$) and concentrated under reduced pressure. The crude mixture (1.08 g, 79%) was used in the next step without further purification.

^{1}H NMR (400 MHz, $CDCl_3$) δ = 5.74 (dt, *J* = 15.3, 6.2 Hz, 1H), 5.24 (ddt, *J* = 15.3, 8.9, 1.3 Hz, 1H), 4.08 (td, *J* = 6.0, 1.2 Hz, 2H), 1.42 (m, 1H), 1.22 (t, *J* = 5.8 Hz, 1H), 0.77 – 0.70 (m, 2H), 0.42 – 0.35 (m, 2H) ppm.

^{13}C NMR (101 MHz, $CDCl_3$) δ = 137.4, 126.4, 63.7, 13.4, 6.7 ppm.

LRMS (EI$^+$) *m/z* (relative intensity): 98.1 (17%) $[M]^{+\bullet}$, 67.0 (100%).

IR ν_{max} (neat) 3318, 2923, 2870, 1726, 1458, 1365, 1262, 1014, 738 cm^{-1}.

(*E*)-(3-Bromoprop-1-en-1-yl)cyclopropane

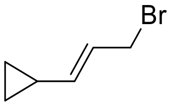

(*E*)-(3-Bromoprop-1-en-1-yl)cyclopropane was prepared following a literature procedure:[41] To a solution of (*E*)-3-cyclopropylprop-2-en-1-ol (0.982 g, 10.0 mmol) in dry Et_2O (40.0 mL) was added PBr_3 (1.41 mL, 15.0 mmol) at 0 °C. The reaction mixture was stirred for 2 h at 0 °C, then allowed to warm to rt and stirred for another 2 h at the same temperature. Ice water was added to the reaction mixture and the aqueous layer was extracted with Et_2O (3x 10 mL). The combined organic layers were washed with brine, dried ($MgSO_4$) and concentrated under reduced pressure to afford (*E*)-(3-bromoprop-1-en-1-yl)cyclopropane as a crude yellow oil that was used directly in the next step without further purification.

(*E*)-1-((3-Cyclopropylallyl)oxy)-2-iodobenzene (SI1) (*E:Z* ~ 7:1)

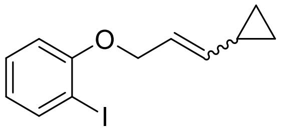

(*E*)-1-((3-Cyclopropylallyl)oxy)-2-iodobenzene (**SI1**) was prepared by **General Method C** from 2-iodophenol (1.50 g, 6.83 mmol) and (*E*)-(3-bromoprop-1-en-1-yl)cyclopropane (1.10 g, 6.83 mmol).

The crude mixture was purified by flash chromatography (hexane:toluene 19:1) to afford (*E*)-1-((3-cyclopropylallyl)oxy)-2-iodobenzene (**SI1**, 0.498 g, 25%) as a colourless oil. The product was isolated as a mixture of alkene isomers (*E:Z* ~ 7:1, ^{1}H NMR)

^{1}H NMR (400 MHz, $CDCl_3$) Recorded for a mixture of *E* and *Z* isomers (*E:Z* ~7:1) δ = 7.79$_Z$ and 7.78$_E$ (dd, *J* = 7.8, 1.6 Hz, 1H), 7.32 – 7.25$_{E/Z}$ (m, 1H), 6.89$_Z$ and 6.82$_E$ (dd, *J* = 8.2, 1.3 Hz, 1H), 6.74 – 6.68$_{E/Z}$ (m, 1H), 5.80$_E$ (dtd, *J* = 15.3, 5.8, 0.5 Hz, 1H (*E*)), 5.63$_Z$ (dtd, *J* = 10.9, 6.4, 0.8 Hz, 1H (*Z*)), 5.42$_E$ (ddt, *J* = 15.3, 8.9, 1.4 Hz, 1H (*E*)), 5.07 – 5.00$_Z$ (m, 1H (*Z*)), 4.80$_Z$ (dd, *J* = 6.4, 1.5 Hz, 2H (*Z*)), 4.53$_E$ (dd, *J* = 5.9, 1.4 Hz, 2H (*E*)), 1.70 – 1.59$_Z$ (m, 1H (*Z*)), 1.52 – 1.42$_E$ (m, 1H (*E*)), 0.87 – 0.81$_Z$ (m, 2H (*Z*)), 0.80 – 0.71$_E$ (m, 2H (*E*)), 0.47 – 0.41$_{E/Z}$ (m, 2H (*E/Z*)) ppm.

^{13}C NMR (101 MHz, $CDCl_3$) Recorded for a mixture of *E* and *Z* isomers (*E:Z* ~7:1) δ = 157.3$_{E/Z}$, 139.5$_Z$, 139.5$_E$, 139.2$_E$, 138.6$_Z$, 129.3$_E$, 122.5$_E$, 122.4$_Z$, 121.8$_E$, 112.7$_E$, 112.7$_Z$, 86.9$_{E/Z}$, 69.9$_E$, 65.8$_Z$, 13.6$_E$, 10.2$_Z$, 7.2$_Z$, 6.8$_E$ ppm.

LRMS (EI$^+$) *m/z* (relative intensity): 300.1 (1%) $[M]^{+•}$, 81.2 (100%).

HRMS (EI$^+$) *m/z*: $[M]^{+•}$ Calcd for $C_{12}H_{13}IO$: 300.0004; found 300.0006.

IR ν_{max} (neat) 3003, 2867, 1668, 1580, 1469, 1438, 1274, 1241, 1016, 962, 746 cm^{-1}.

3-(But-1-en-1-yl)-2,3-dihydrobenzofuran (SI2, *E:Z* ~ 3:1)

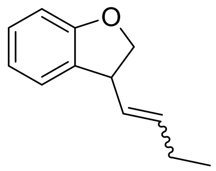

3-(But-1-en-1-yl)-2,3-dihydrobenzofuran (**SI2**) was prepared by **General Method B** from (*E*)-1-((3-cyclopropylallyl)oxy)-2-iodobenzene (**SI1**, *E/Z* ~7:1, 0.150 g, 0.500 mmol). The crude mixture was purified by flash chromatography (hexane:toluene = 19:1) to afford 3-(but-1-en-1-yl)-2,3-dihydrobenzofuran (**SI2**, 0.045 g, 52%) as a colourless oil. The product was isolated as a mixture of alkene isomers (*E:Z* ~ 3:1, ^{1}H NMR).

^{1}H NMR (400 MHz, $CDCl_3$) Spectra presented as a mixture of *E* and *Z* isomers (~ 3:1) δ = 7.28$_Z$ (m, 1H), 7.15 – 7.05$_{E/Z}$ (m, 2H (*E*), 1H (*Z*)), 6.91 – 6.83$_{E/Z}$ (m, 1H), 6.82 – 6.76$_{E/Z}$ (m, 1H), 5.68$_E$ (dt, *J* = 15.2, 6.3 Hz, 1H), 5.61$_Z$ (m, 1H), 5.50 – 5.37$_{E/Z}$ (m, 1H), 4.74 – 4.60$_{E/Z}$ (m, 1H), 4.21 – 4.03$_{E/Z}$ (m, 2H), 2.20$_Z$ (m, 1H), 2.12 – 2.03$_E$ (m, 2H), 1.06$_Z$ (t, *J* = 7.5 Hz, 1H), 1.01$_E$ (t, *J* = 7.5 Hz, 3H) ppm.

^{13}C NMR (101 MHz, $CDCl_3$) Spectra presented as a mixture of *E* and *Z* isomers (~ 3:1) δ = 159.9$_Z$, 159.8$_E$, 134.7$_E$, 134.1$_Z$, 130.1$_E$, 129.4$_Z$, 128.5$_Z$, 128.5$_E$, 128.3$_E$, 128.2$_Z$, 124.8$_E$, 124.5$_Z$, 120.6$_Z$, 120.5$_E$, 109.5$_E$, 109.4$_Z$, 76.8$_Z$, 76.7$_E$, 45.9$_E$, 40.6$_Z$, 25.4$_E$, 21.0$_Z$, 14.7$_Z$, 13.7$_E$ ppm.

LRMS (EI$^+$) *m/z* (relative intensity): 174.2 (62%) $[M]^{+•}$, 145.2 (100%).

HRMS (EI$^+$) *m/z*: $[M]^{+•}$ Calcd for $C_{12}H_{14}O$: 174.1039; found 174.1038.

IR ν_{max} (neat) 2961, 2873, 1596, 1479, 1458, 1221, 965, 748 cm^{-1}.

Tetrabutylammonium triiodide

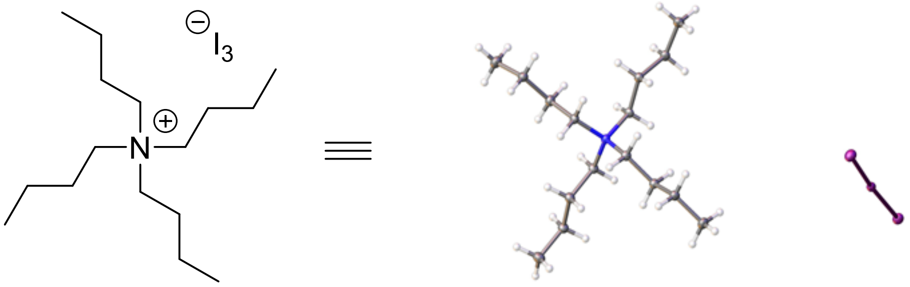

Tetrabutylammonium triiodide was isolated as an anodic oxidation product from the reaction to prepare 3-methyl-2,3-dihydrobenzofuran (**2**) using **General Method B** from 1-(allyloxy)-2-iodobenzene (**1**, 0.130 g, 0.50 mmol). The reaction was carried out in the absence of phenanthrene to facilitate isolation of the triiodide salt. Tetrabutylammonium triiodide was obtained as a black/dark purple solid (0.110 g, 70%) after recrystallization of the crude mixture from hot ethanol. The structure of the compound was confirmed by x-ray analysis.[46]

^{1}H NMR (400 MHz, $CDCl_3$) δ = 3.34 – 3.25 (m, 2H), 1.80 – 1.67 (m, 2H), 1.60 – 1.47 (m, 2H), 1.08 (t, *J* = 7.3 Hz, 3H) ppm.

^{13}C NMR (101 MHz, $CDCl_3$) δ = 59.5, 24.3, 20.0, 13.9 ppm.

IR ν_{max} (neat) 2952, 2924, 2867, 1435, 1370, 1021, 878, 741 cm^{-1}

mp: 70 – 72 °C (Lit:[2] 70.0 – 70.5 °C)

CCDC: 2018493[46]

12 References

[1] R. A. Green, R. C. D. Brown, D. Pletcher, B. Harji, *Electrochem. Commun.* **2016**, *73*, 63–66.

[2] R. E. Buckles, J. P. Yuk, *J. Am. Chem. Soc.* **1953**, *75*, 5048–5052.

[3] K. Mitsudo, Y. Nakagawa, J.-I. Mizukawa, H. Tanaka, R. Akaba, T. Okada, S. Suga, *Electrochim. Acta* **2012**, *82*, 444–449.

[4] C. Costentin, M. Robert, J. Save, *J. Am. Chem. Soc.* **2004**, *126*, 16051–16057.

[5] N. Takeda, P. V. Poliakov, A. R. Cook, J. R. Miller, *J. Am. Chem. Soc.* **2004**, *126*, 4301–4309.

[6] C. Chatgilialoglu, A. Studer, Eds. , *Encyclopedia of Radicals in Chemistry, Biology and Materials*, John Wiley & Sons, Ltd, **2012**.

[7] L. J. Johnston, J. Lusztyk, D. D. M. Wayner, A. N. Abeywickreyma, A. L. J. Beckwith, J. C. Scaiano, K. U. Ingold, *J. Am. Chem. Soc.* **1985**, *107*, 4594–4596.

[8] W. R. Bowman, S. L. Krintel, M. B. Schilling, *Org. Biomol. Chem.* **2004**, *2*, 585–592.

[9] E. M. Rochette, W. Lewis, A. G. Dossetter, R. A. Stockman, *Chem. Commun.* **2013**, *49*, 9395–9397.

[10] C. E. Anson, A. V. Malkov, C. Roe, E. J. Sandoe, G. R. Stephenson, *Eur. J. Org. Chem.* **2008**, 196–213.

[11] R. Trivedi, J. A. Tunge, *Org. Lett.* **2009**, *11*, 5650–5652.

[12] Y. L. Lin, J. Y. Cheng, Y. H. Chu, *Tetrahedron* **2007**, *63*, 10949–10957.

[13] V. Dichiarante, M. Fagnoni, M. Mella, A. Albini, *Chem. Eur. J.* **2006**, *12*, 3905–3915.

[14] J. A. Murphy, T. A. Khan, S. Z. Zhou, D. W. Thomson, M. Mahesh, *Angew. Chem. Int. Ed.* **2005**, *44*, 1356–1360.

[15] N. Kurono, E. Honda, F. Komatsu, K. Orito, M. Tokuda, *Tetrahedron* **2004**, *60*, 1791–1801.

[16] R. Lhermet, M. Durandetti, J. Maddaluno, *Beilstein J. Org. Chem.* **2013**, *9*, 710–716.

[17] S. P. Seitz, J. A. Markwalder, A. V. Purandare, *WO2015077194A1.Pdf*, **2015**, WO2015077194A1.

[18] B. Michelet, C. Deldaele, S. Kajouj, C. Moucheron, G. Evano, *Org. Lett.* **2017**, *19*, 3576–3579.

[19] Y. Yoshimi, H. Kanai, K. Nishikawa, Y. Ohta, Y. Okita, K. Maeda, T. Morita, *Tetrahedron Lett.* **2013**, *54*, 2419–2422.

[20] B. S. N. Huchenski, K. N. Robertson, A. W. H. Speed, *Eur. J. Org. Chem.* **2020**, *2020*, 5140–5144.

[21] N. Hayashi, I. Shibata, A. Baba, *Org. Lett.* **2004**, *6*, 4981–4983.

[22] C. Bolm, M. Ewald, M. Zehnder, M. A. Neuburger, *Chem. Ber.* **1992**, *125*, 453–458.

[23] A. Dahlén, A. Petersson, G. Hilmersson, *Org. Biomol. Chem.* **2003**, *1*, 2423–2426.

[24] L. F. Tietze, A. Düfert, F. Lotz, L. Sölter, K. Oum, T. Lenzer, T. Beck, R. Herbst-Irmer, *J. Am. Chem. Soc.* **2009**, *131*, 17879–17884.

[25] K. Murugesan, T. Senthamarai, A. S. Alshammari, R. M. Altamimi, C. Kreyenschulte, M. M. Pohl, H. Lund, R. V. Jagadeesh, M. Beller, *ACS Catal.* **2019**, *9*, 8581–8591.

[26] M. Costanzo, S. Yabut, B. Tounge, B. Maryanoff, H. Zhang, *WO 2009/067202 A1*, **2009**, WO2009/067202A1.

[27] G. Majetich, J. Song, C. Ringold, G. A. Nemeth, M. G. Newton, *J. Org. Chem.* **1991**, *56*, 3973–3988.

[28] B. M. Stadler, P. Puylaert, J. Diekamp, R. van Heck, Y. Fan, A. Spannenberg, S. Hinze, J. G. de Vries, *Adv. Synth. Catal.* **2018**, *360*, 1151–1158.

[29] L. Pagès, A. Llebaria, F. Camps, E. Molins, C. Miravitlles, J. M. Moretó, *J. Am. Chem. Soc.* **1992**, *114*, 10449–10461.

[30] I. R. Hazelden, R. C. Carmona, T. Langer, P. G. Pringle, J. F. Bower, *Angew. Chem. Int. Ed.* **2018**, *57*, 5124–5128.

[31] S. D. Lepore, Y. He, *J. Org. Chem.* **2003**, *68*, 8261–8263.

[32] R. Inoue, J. Nakao, H. Shinokubo, K. Oshima, *Bull. Chem. Soc. Jpn.* **1997**, *70*, 2039–2049.

[33] T. Seki, S. Tanaka, M. Kitamura, *Org. Lett.* **2012**, *14*, 608–611.

[34] H. Kim, C. Lee, *Org. Lett.* **2011**, *13*, 2050–2053.

[35] G. K. Jana, S. Sinha, *Tetrahedron Lett.* **2010**, *51*, 1994–1996.

[36] S. Rohrbach, R. S. Shah, T. Tuttle, J. A. Murphy, *Angew. Chem. Int. Ed.* **2019**, *58*, 11454–11458.

[37] T. Adler, J. Bonjoch, J. Clayden, M. Font-Bardía, M. Pickworth, X. Solans, D. Solé, L. Vallverdú, *Org. Biomol. Chem.* **2005**, *3*, 3173–3183.

[38] A. Minatti, S. L. Buchwald, *Org. Lett.* **2008**, *10*, 2721–2724.

[39] I. Nakamura, Y. Sato, S. Konta, M. Terada, *Tetrahedron Lett.* **2009**, *50*, 2075–2077.

[40] S. O'Sullivan, E. Doni, T. Tuttle, J. A. Murphy, *Angew. Chem. Int. Ed.* **2014**, *53*, 474–478.

[41] E. Ma, Y. Jiang, Y. Chen, L. Qi, X. Yan, Z. Li, *Asian J. Org. Chem.* **2018**, *7*, 914–917.

[42] S. Arai, Y. Koike, H. Hada, A. Nishida, *J. Org. Chem.* **2010**, *75*, 7573–7579.

[43] M. Rueping, M. Leiendecker, A. Das, T. Poisson, L. Bui, *Chem. Commun.* **2011**, *47*, 10629–10631.

[44] J. Barluenga, F. J. Fañanás, R. Sanz, C. Marcos, *Chem Eur. J* **2005**, *11*, 5397–5407.

[45] W. D. Crow, H. McNab, *Aust. J. Chem.* **1979**, *32*, 123–131.

[46] M. E. Light, R. C. D. Brown, A. Folgueiras- Amador, CCDC 2018493: *CSD Communication*, **2022**, doi: 10.5517/ccdc.csd.cc25rdp1

13 NMR spectra

13.1 1-(Allyloxy)-2-iodobenzene (1)

^{1}H NMR (400 MHz, $CDCl_3$):

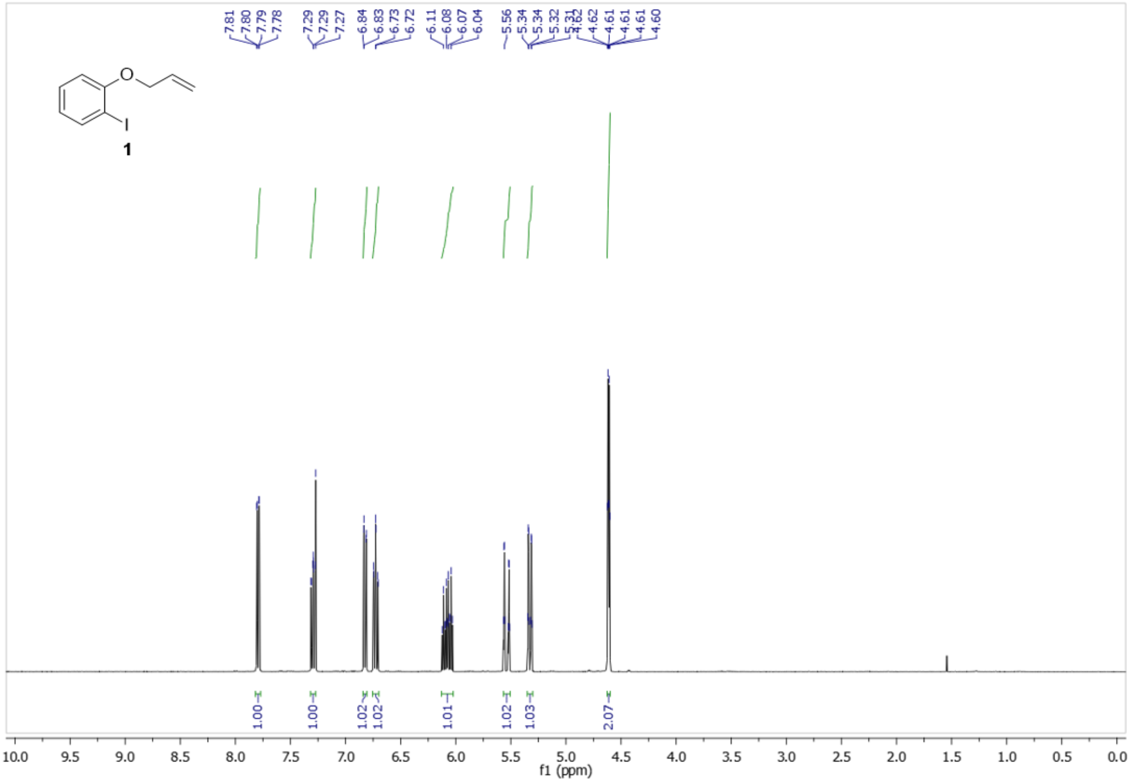

^{13}C NMR (101 MHz, $CDCl_3$):

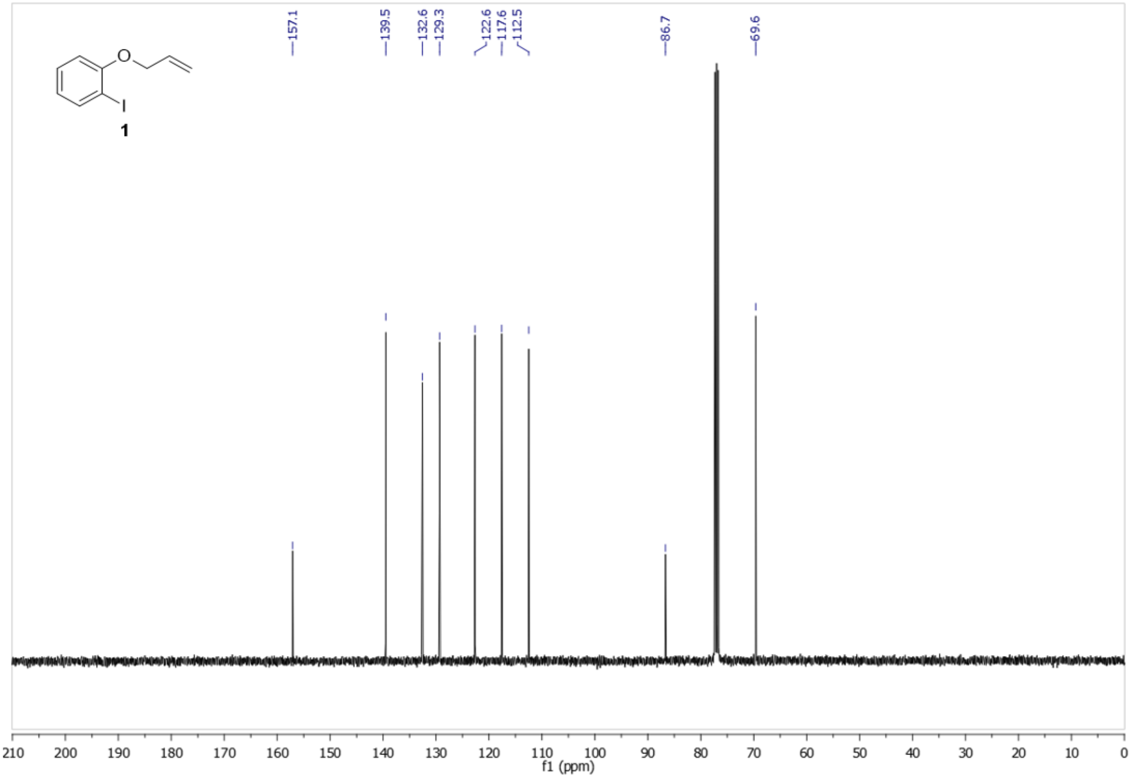

13.2 1-(Allyloxy)-2-bromobenzene

^{1}H NMR (400 MHz, $CDCl_3$):

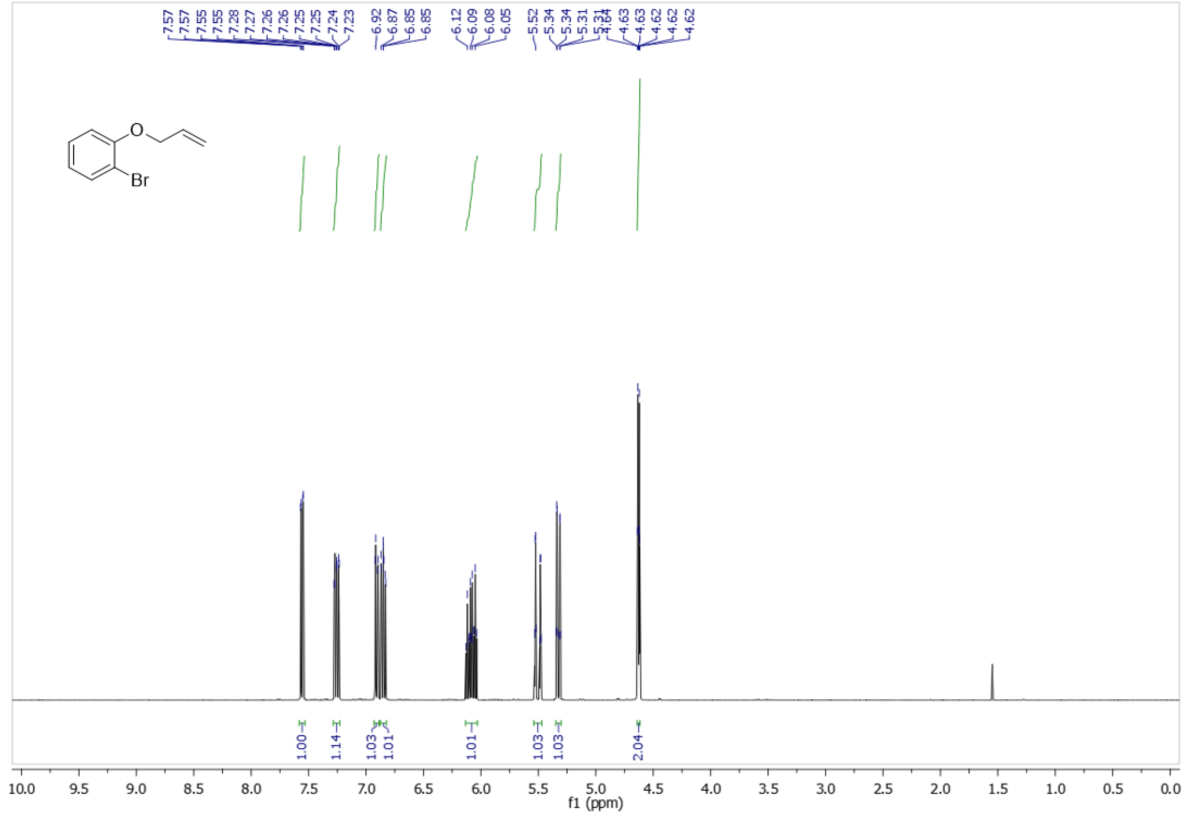

^{13}C NMR (101 MHz, $CDCl_3$):

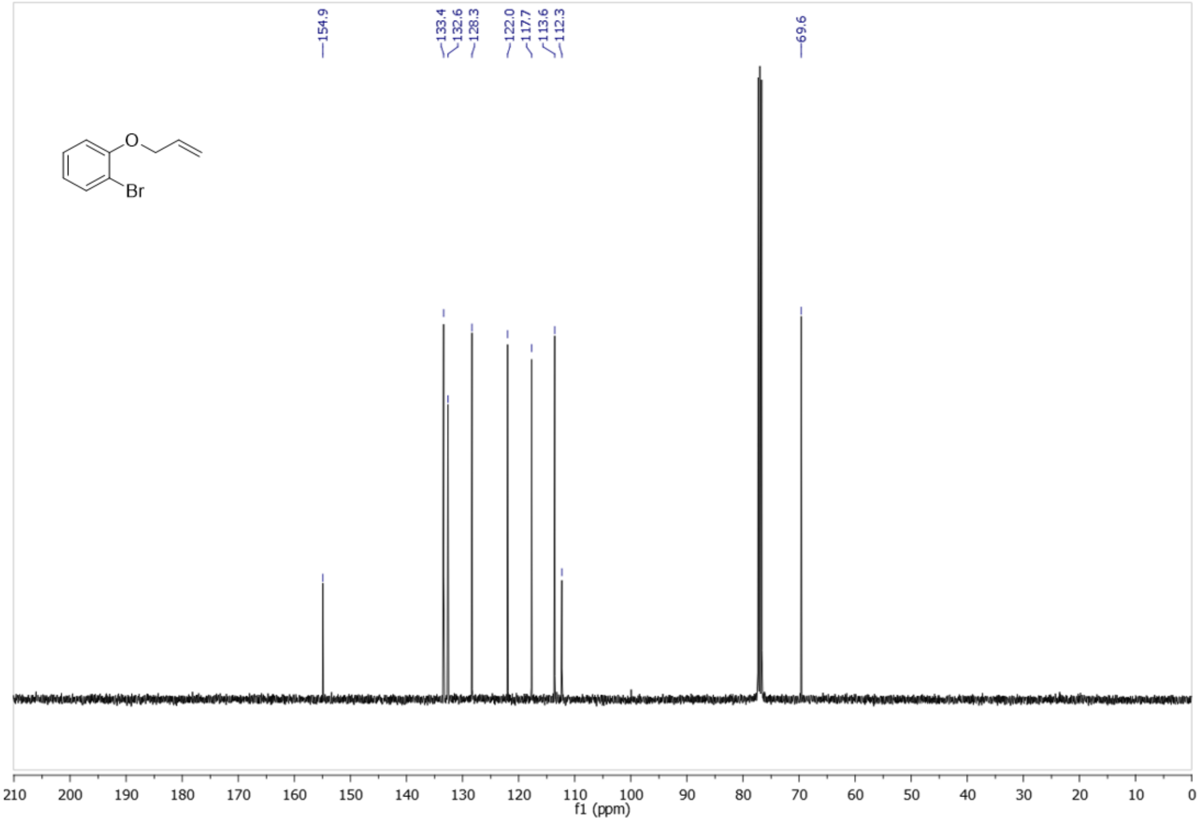

13.3 1-(Allyloxy)-2-chlorobenzene

^{1}H NMR (400 MHz, $CDCl_3$):

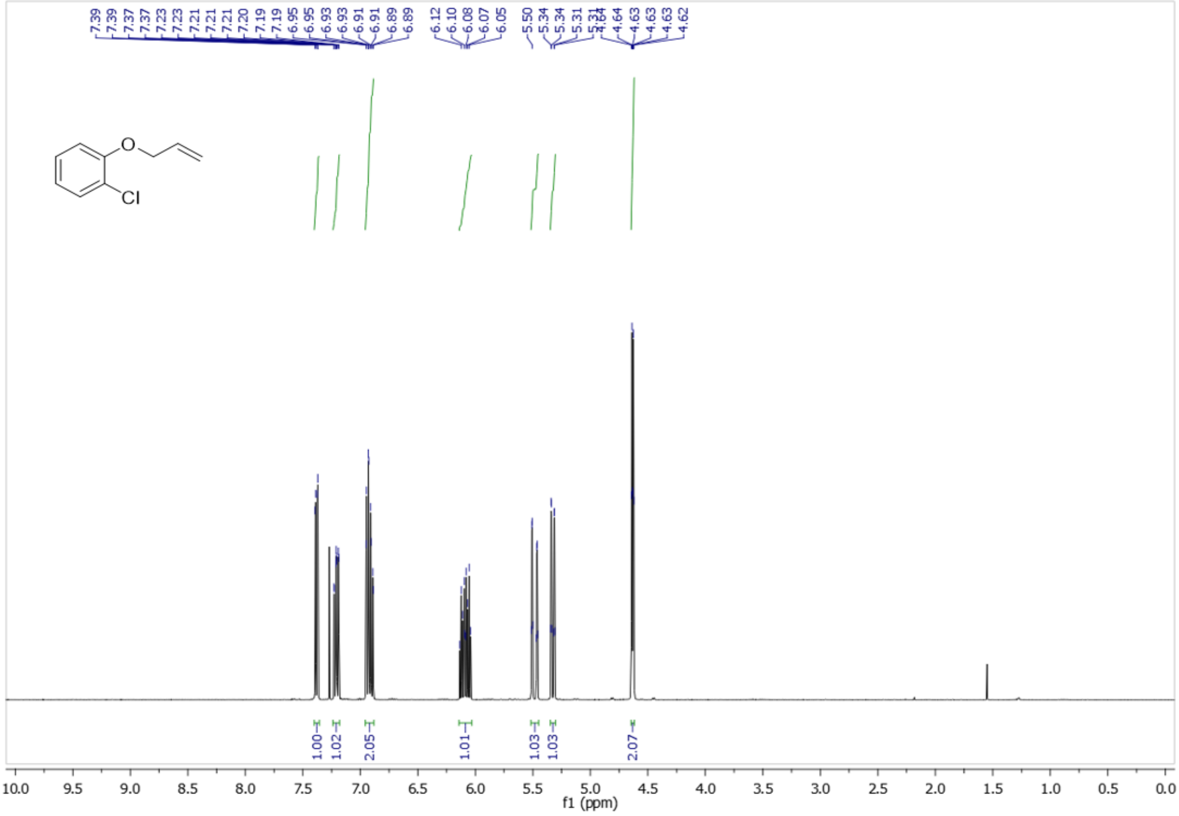

^{13}C NMR (101 MHz, $CDCl_3$):

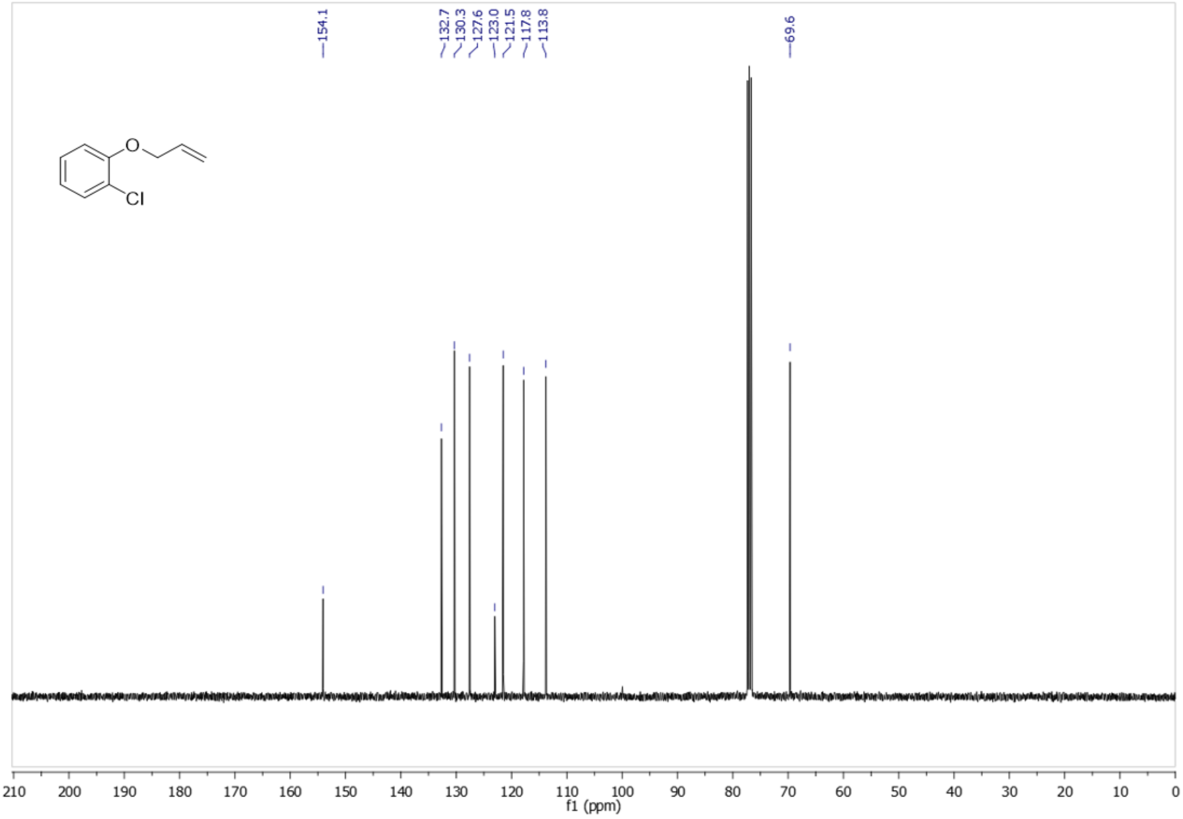

13.4 3-Methyl-2,3-dihydrobenzofuran (2)

^{1}H NMR (400 MHz, $CDCl_3$):

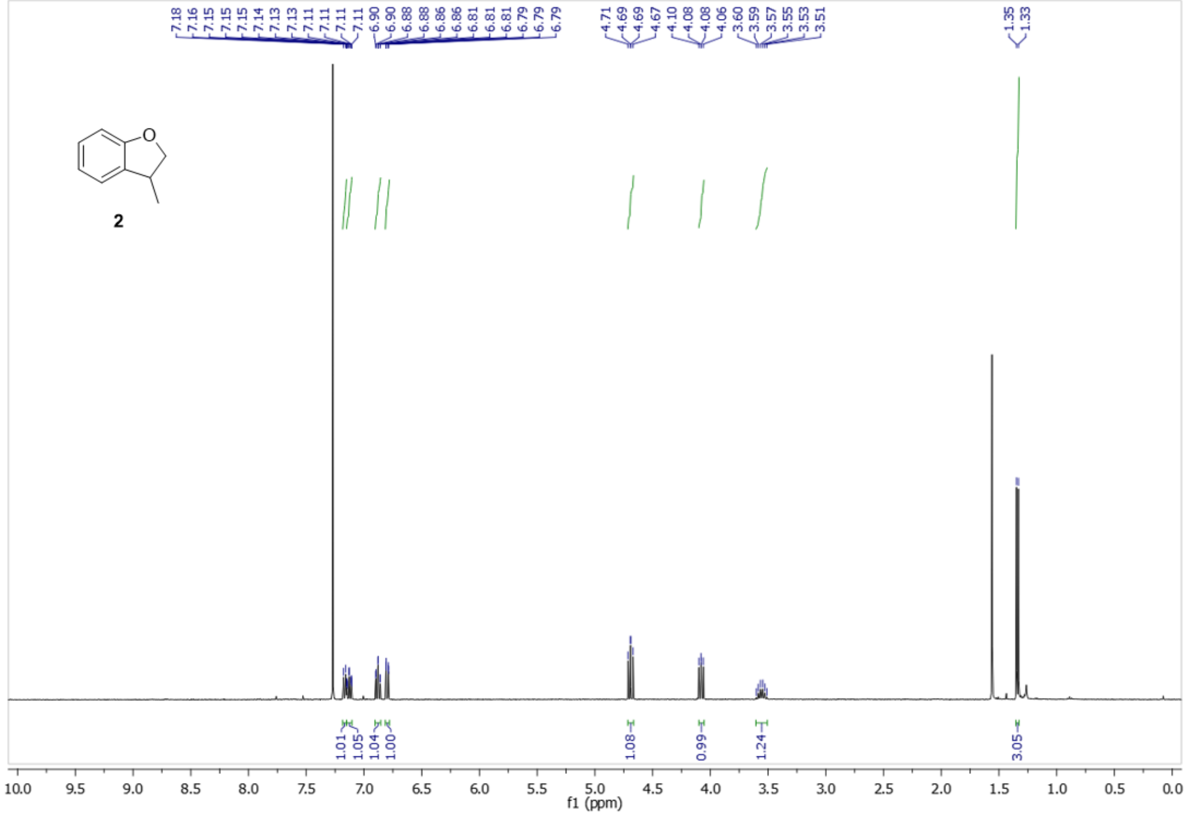

^{13}C NMR (101 MHz, $CDCl_3$):

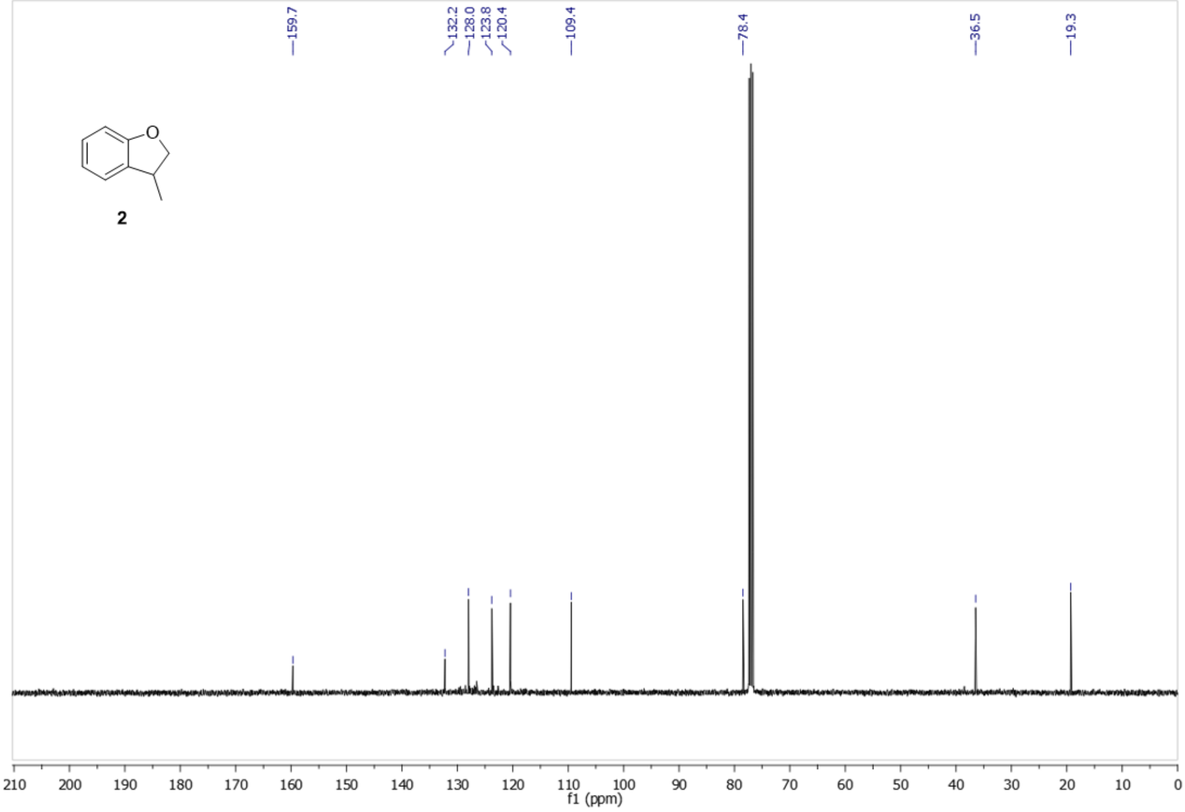

13.5 (Allyloxy)benzene (3)

^{1}H NMR (400 MHz, $CDCl_3$):

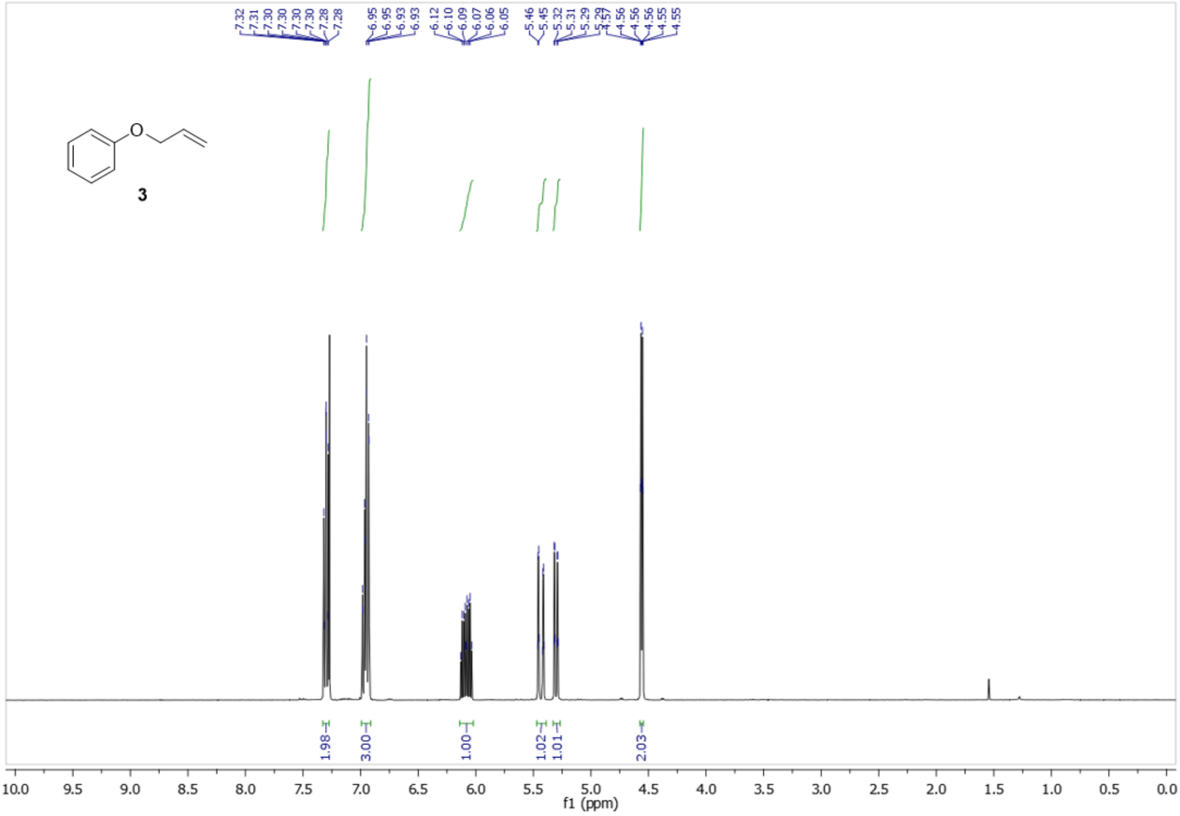

^{13}C NMR (101 MHz, $CDCl_3$):

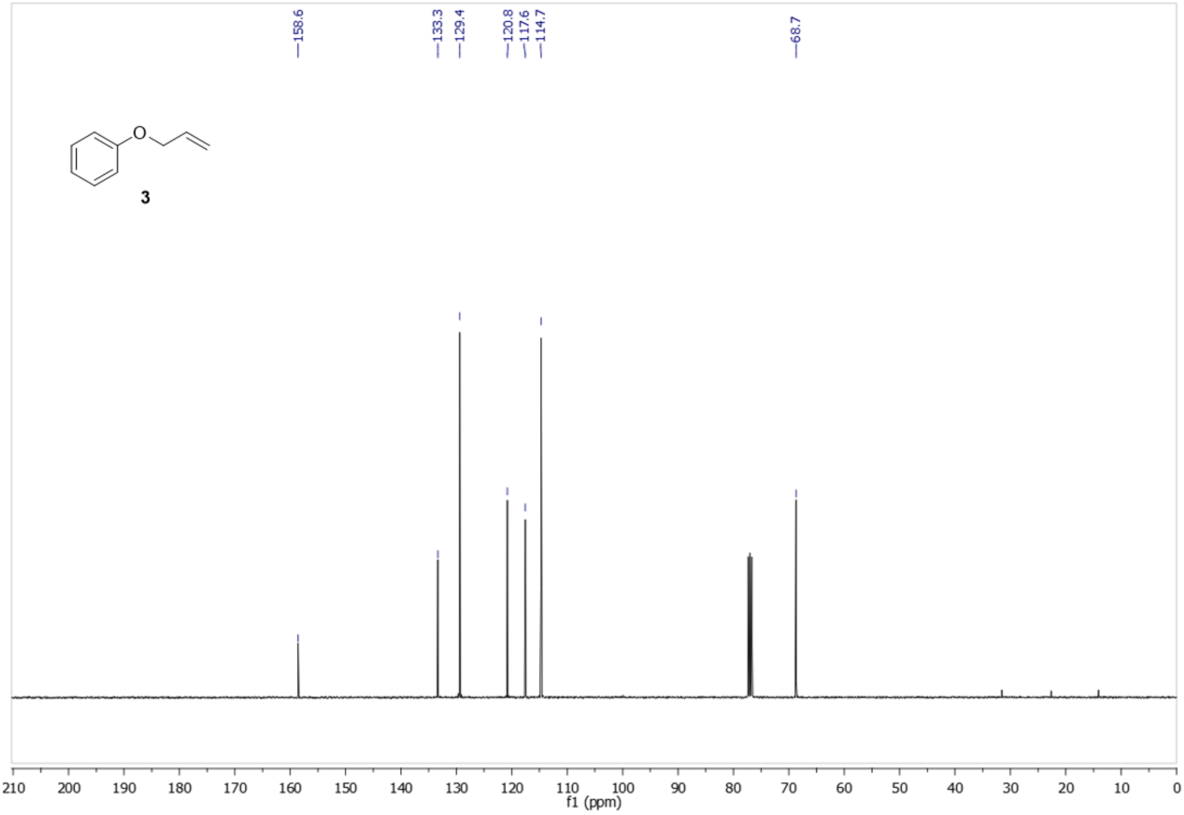

13.6 1,2-bis(2,3-dihydrobenzofuran-3-yl)ethane (4)

^{1}H NMR (400 MHz, $CDCl_3$):

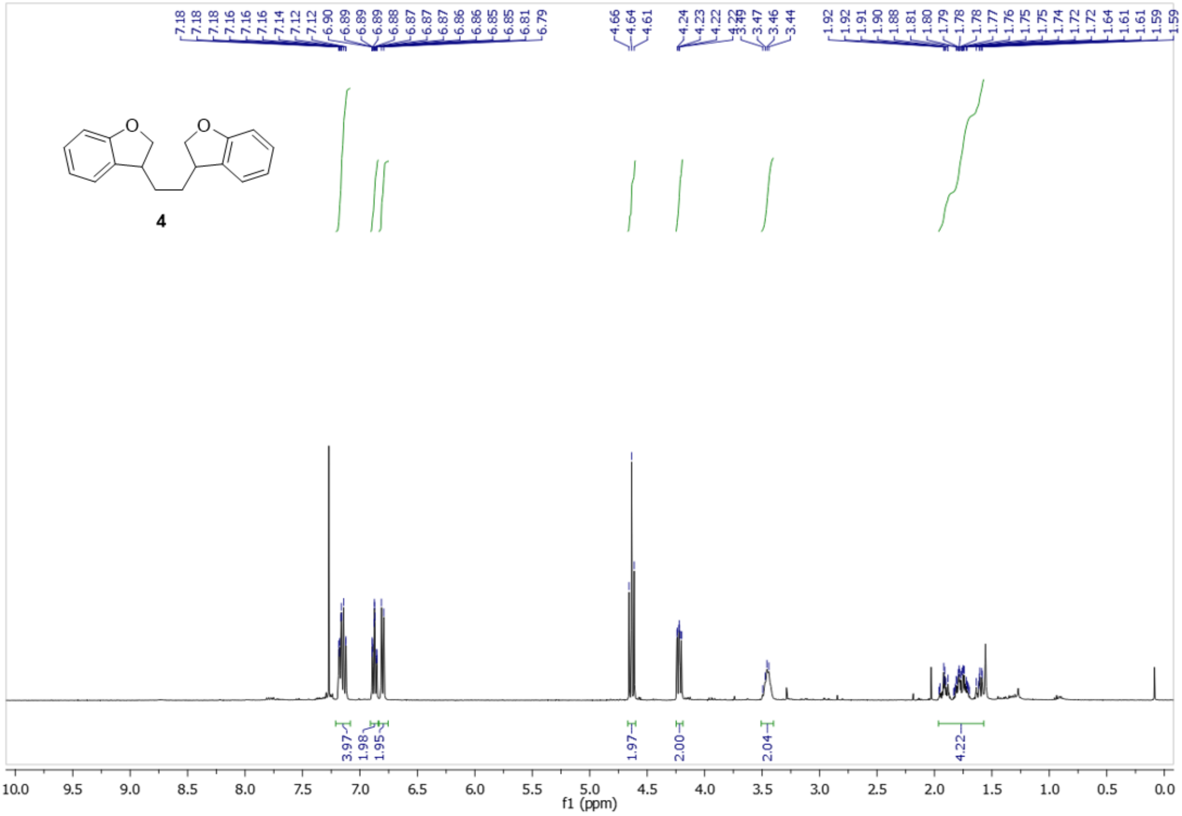

^{13}C NMR (101 MHz, $CDCl_3$):

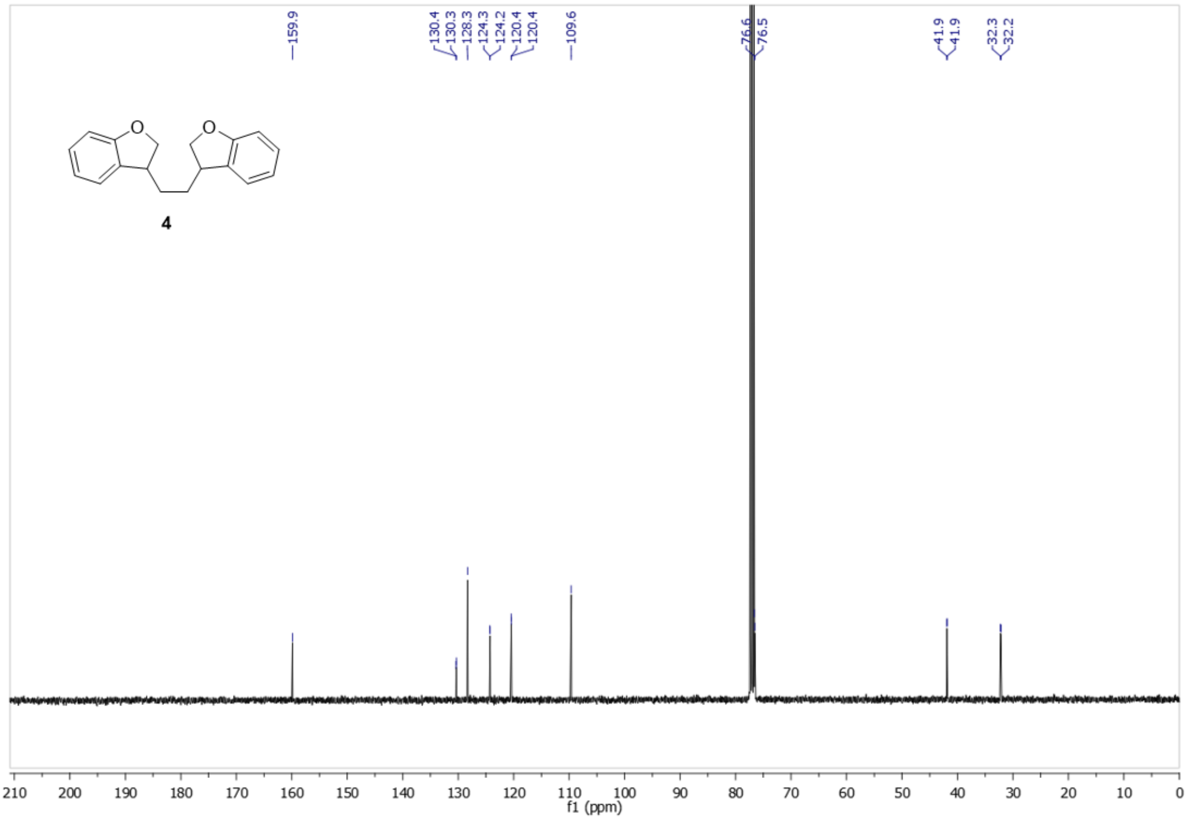

13.7 Methyl 4-(allyloxy)-3-bromobenzoate

^{1}H NMR (400 MHz, $CDCl_3$):

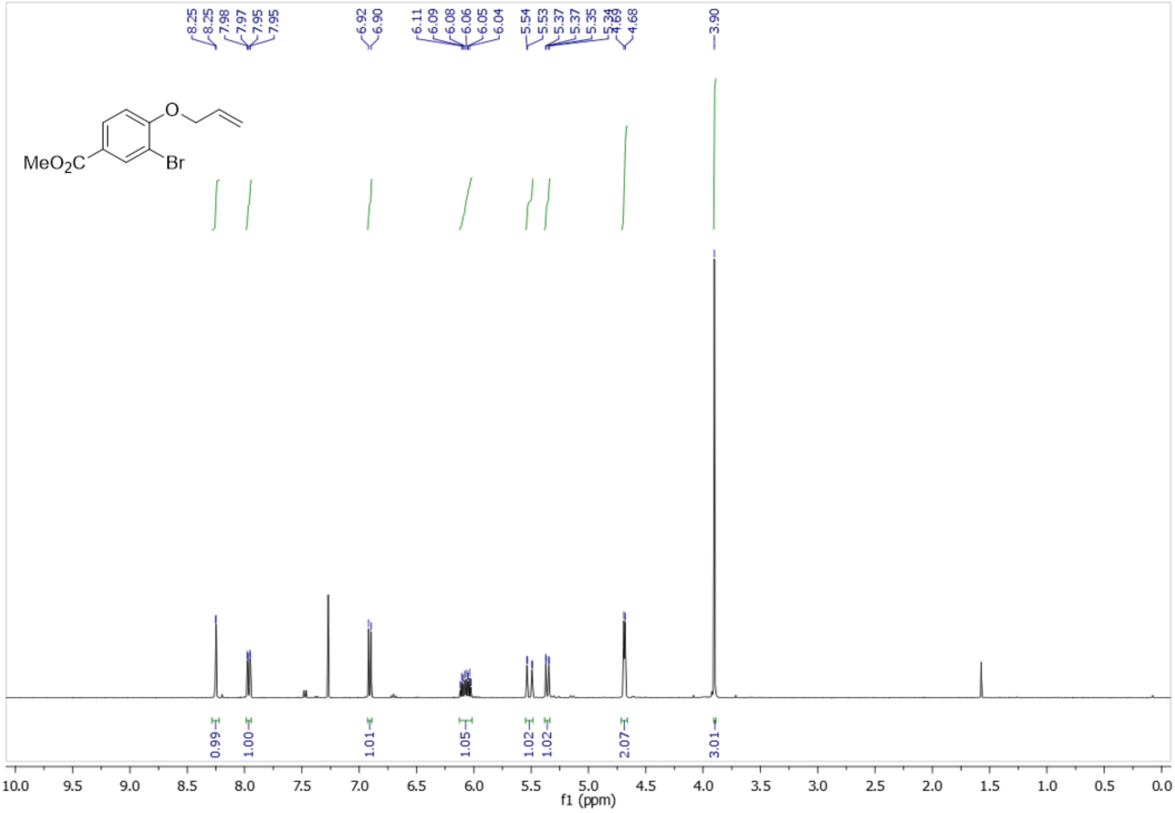

^{13}C NMR (101 MHz, $CDCl_3$):

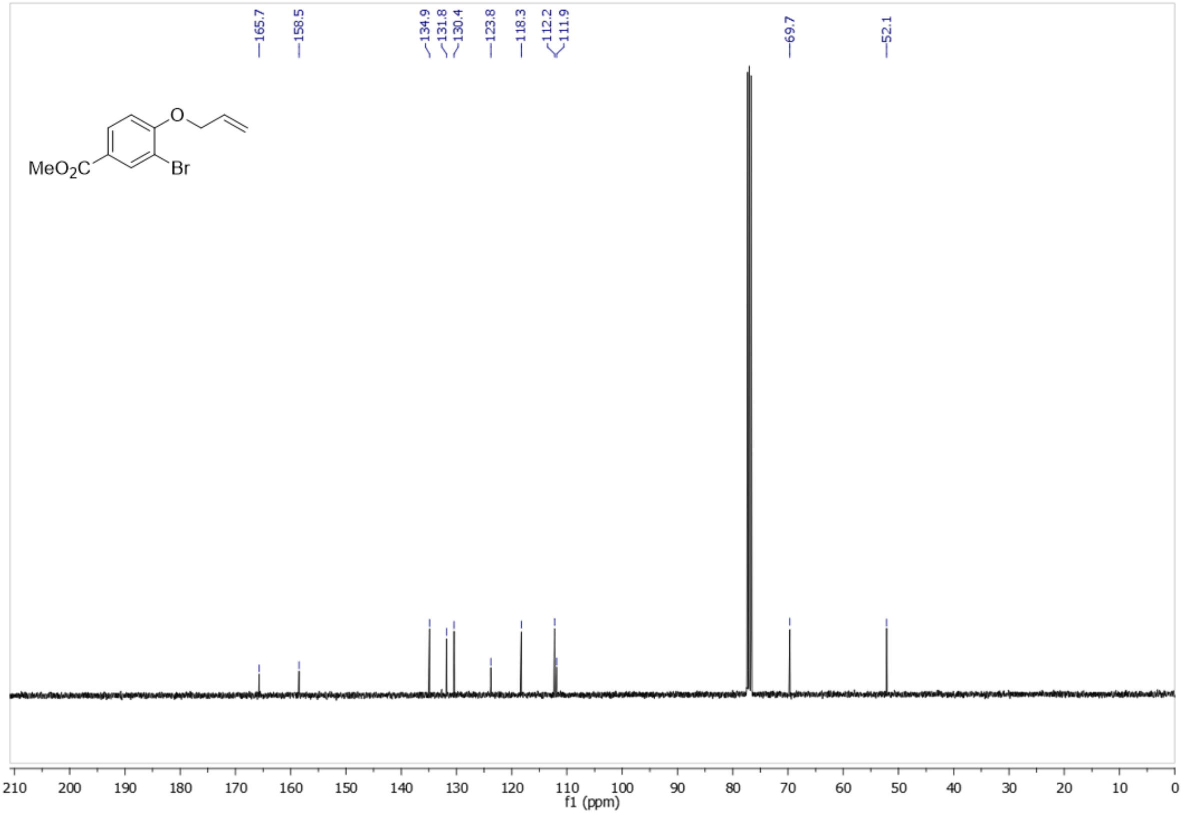

13.8 Methyl 3-methyl-2,3-dihydrobenzofuran-5-carboxylate (5)

^1H NMR (400 MHz, $CDCl_3$):

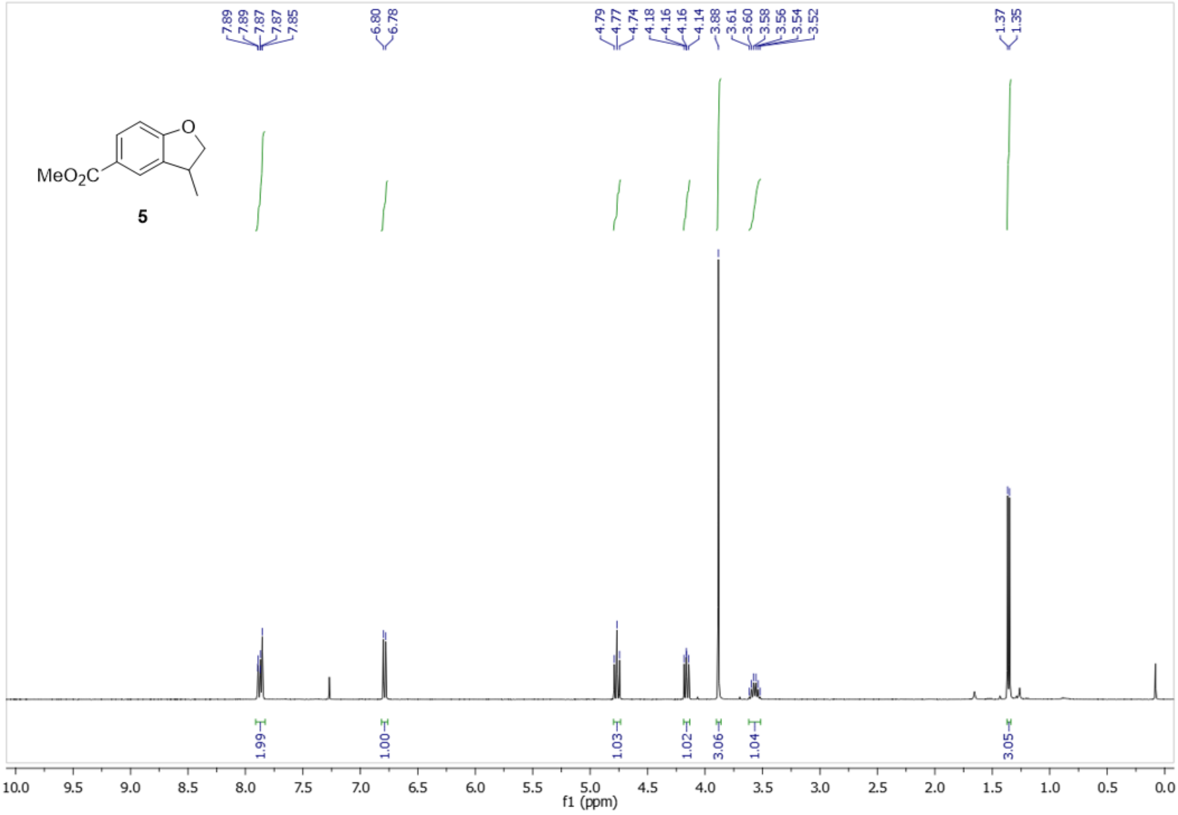

^{13}C NMR (101 MHz, $CDCl_3$):

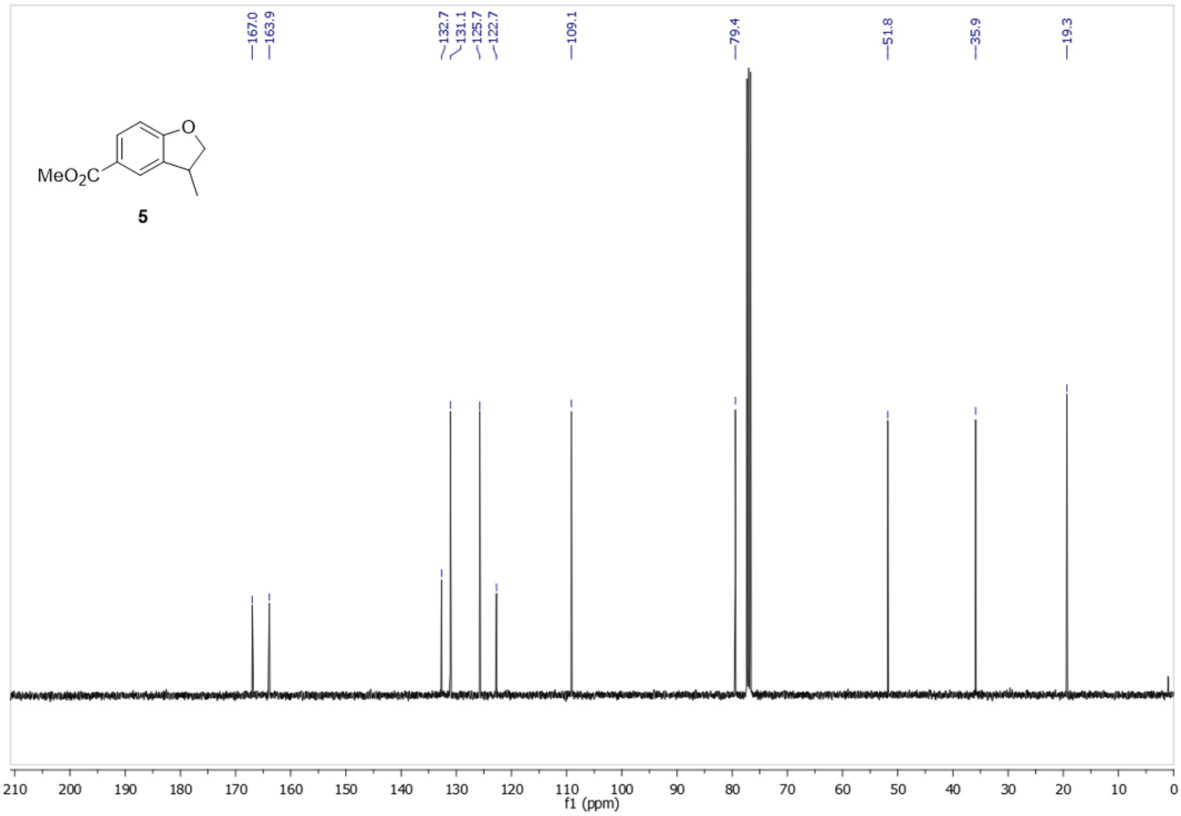

13.9 1-(Allyloxy)-2-bromo-4-methoxybenzene

^{1}H NMR (400 MHz, $CDCl_3$):

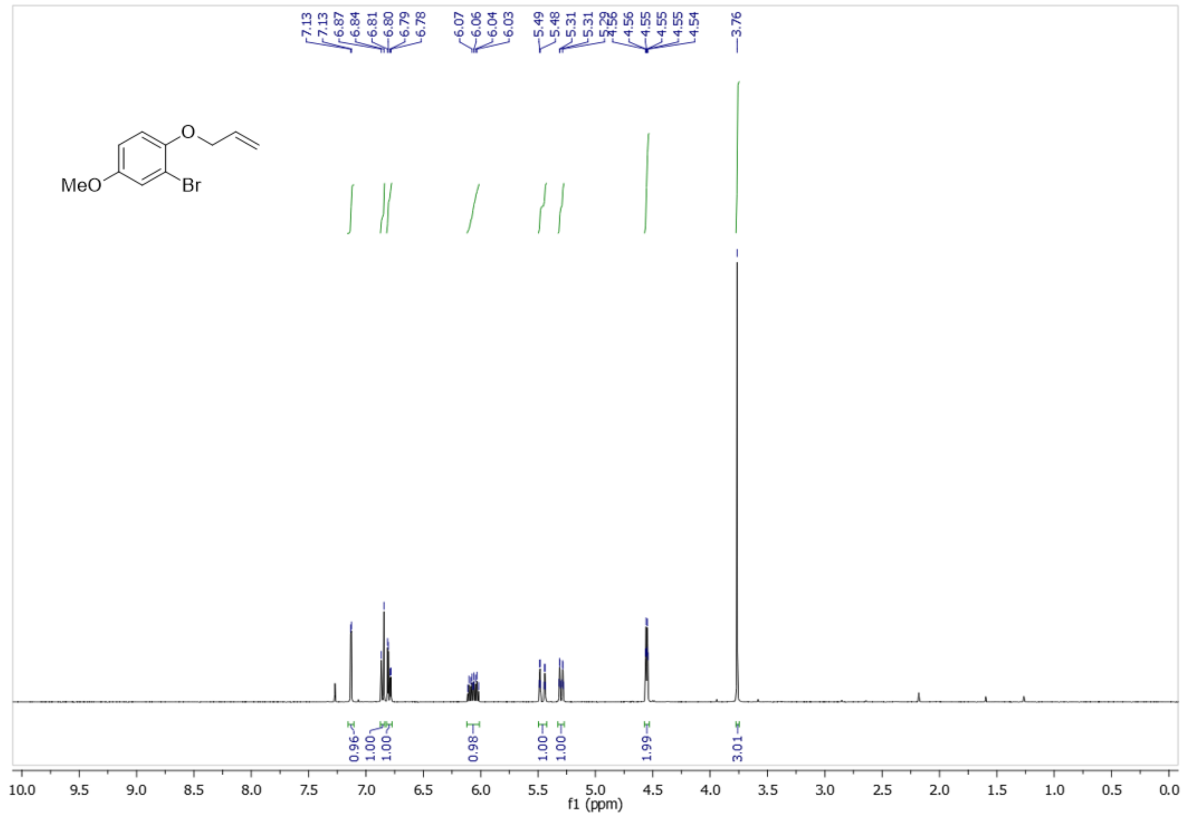

^{13}C NMR (101 MHz, $CDCl_3$):

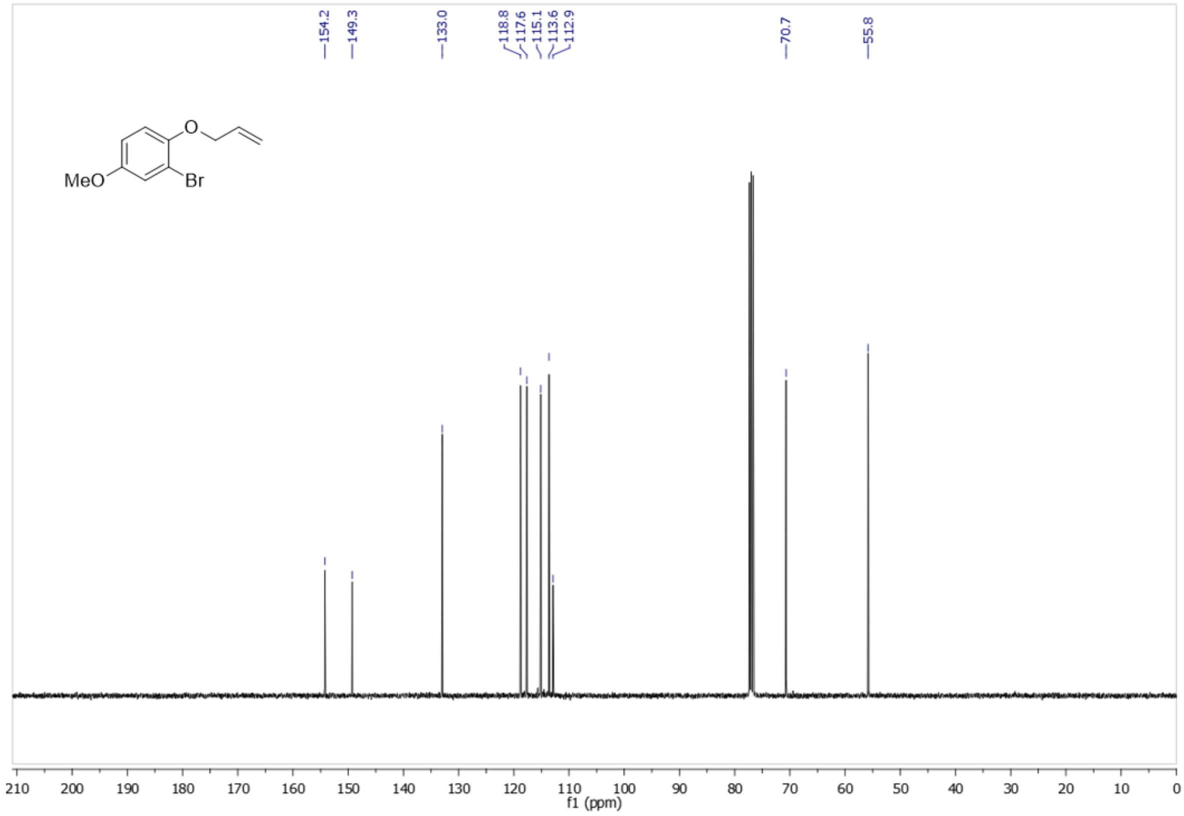

13.10 5-Methoxy-3-methyl-2,3-dihydrobenzofuran (6)

^{1}H NMR (400 MHz, $CDCl_3$):

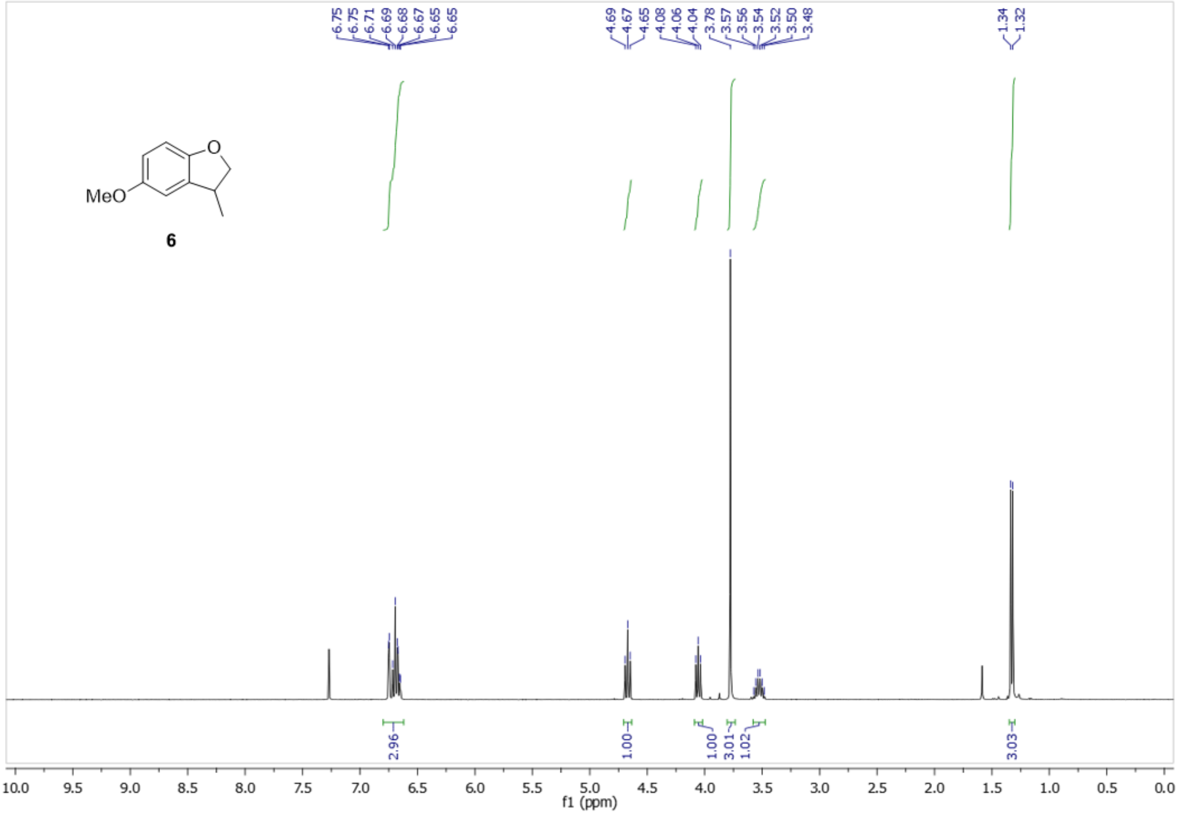

^{13}C NMR (101 MHz, $CDCl_3$):

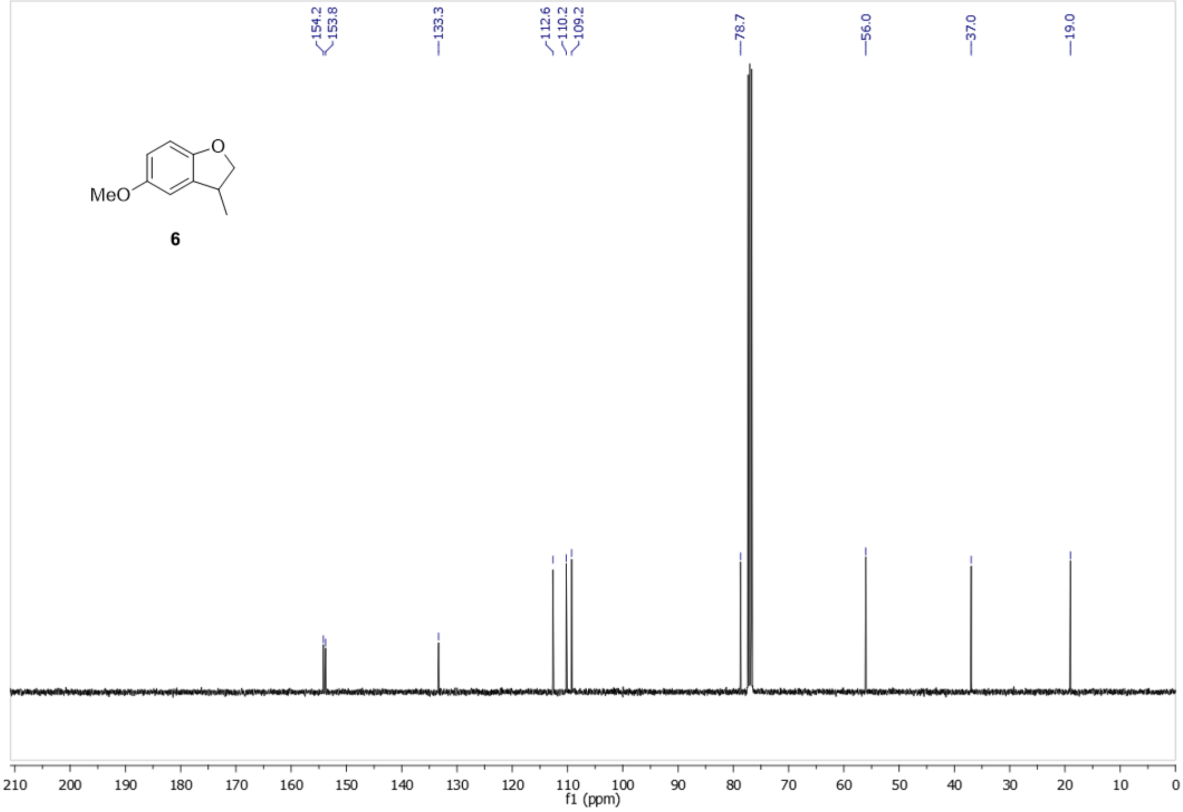

13.11 4-(Allyloxy)-3-bromobenzonitrile

^{1}H NMR (400 MHz, $CDCl_3$):

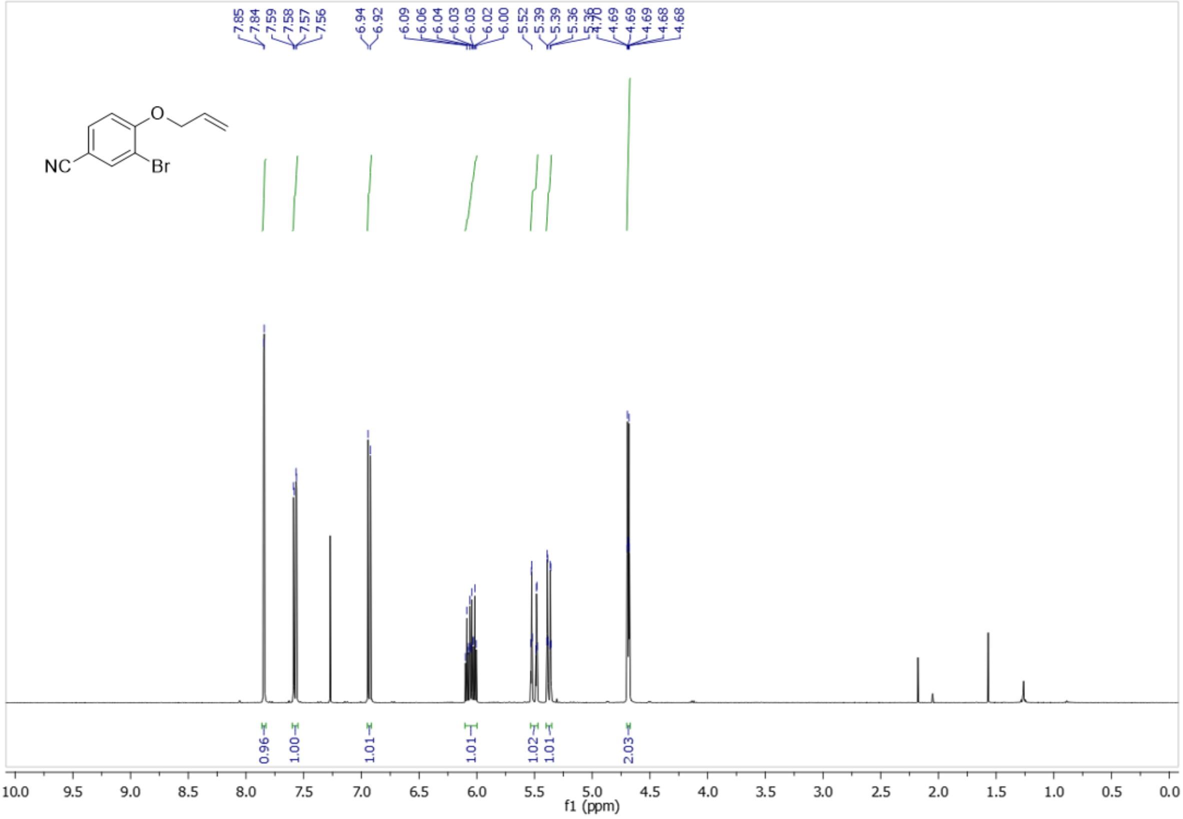

^{13}C NMR (101 MHz, $CDCl_3$):

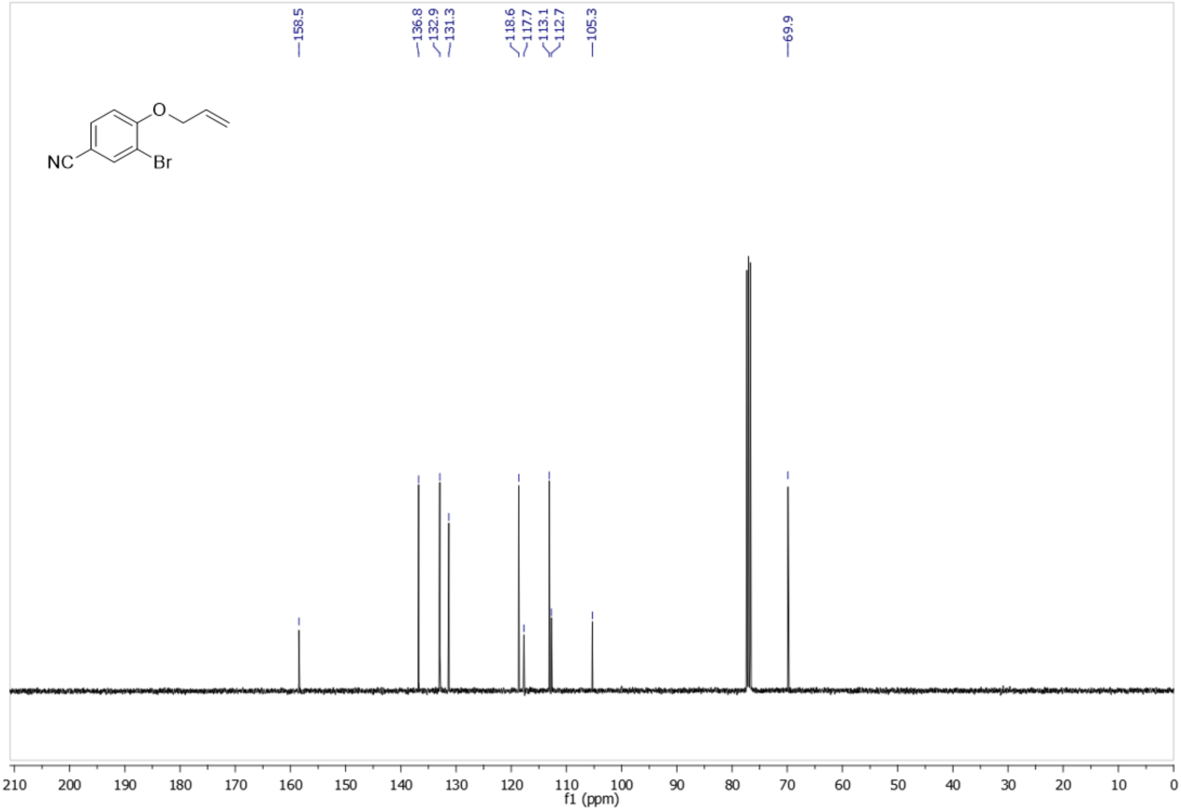

13.12 3-Methyl-2,3-dihydrobenzofuran-5-carbonitrile (7)

^{1}H NMR (400 MHz, $CDCl_3$):

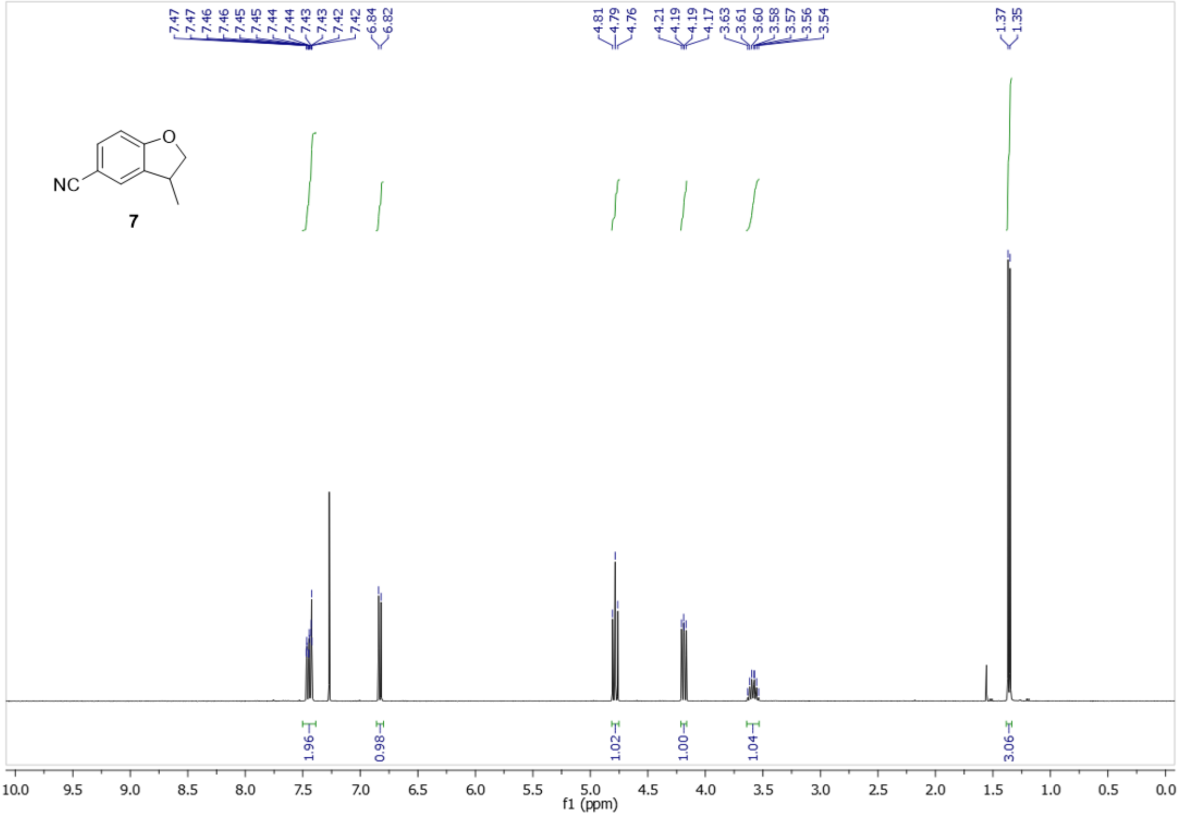

^{13}C NMR (101 MHz, $CDCl_3$):

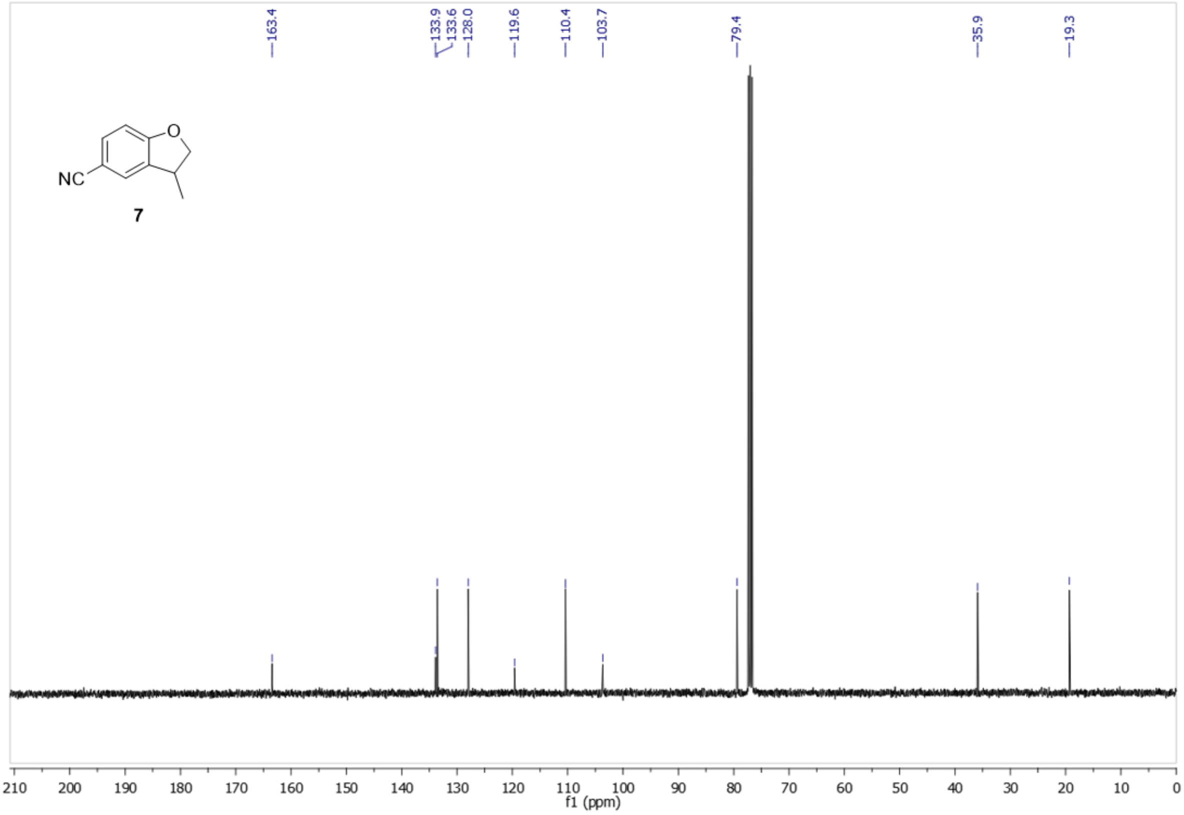

13.13 1-Iodo-2-((3-methylbut-2-en-1-yl)oxy)benzene

^{1}H NMR (400 MHz, $CDCl_3$):

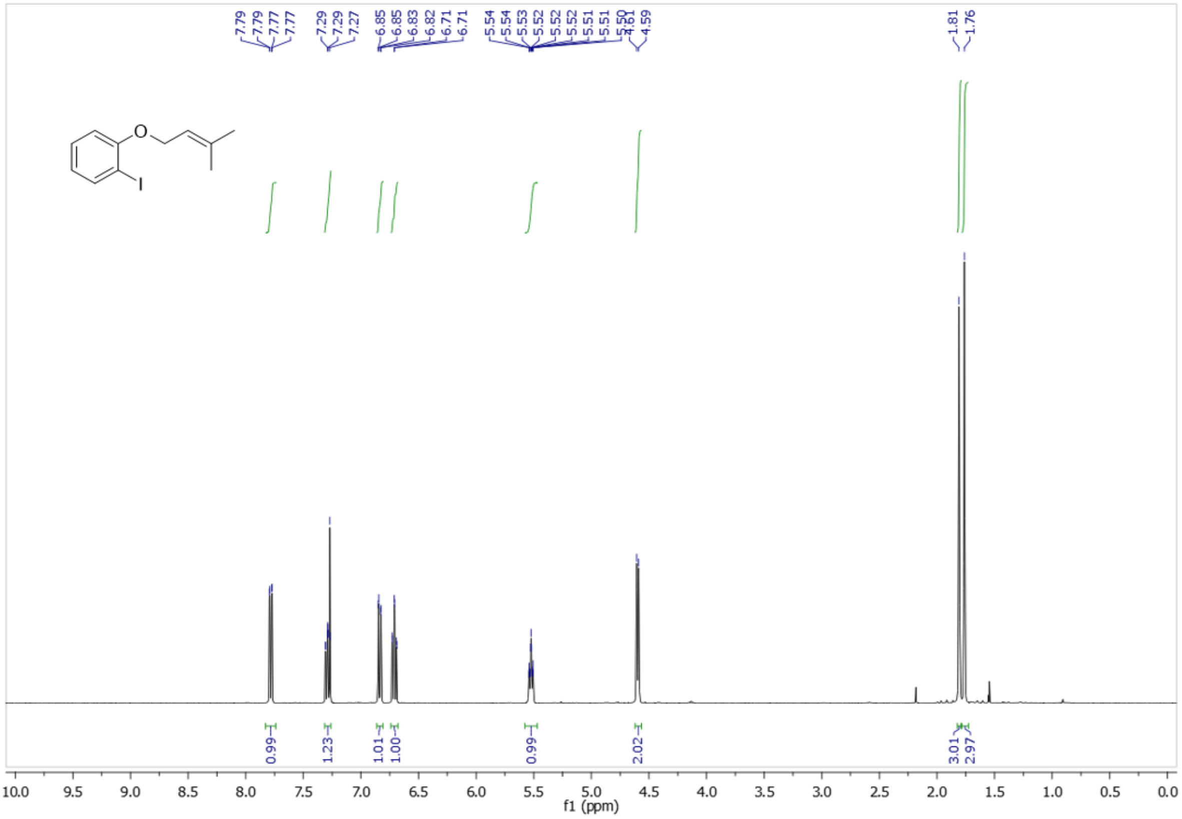

^{13}C NMR (101 MHz, $CDCl_3$):

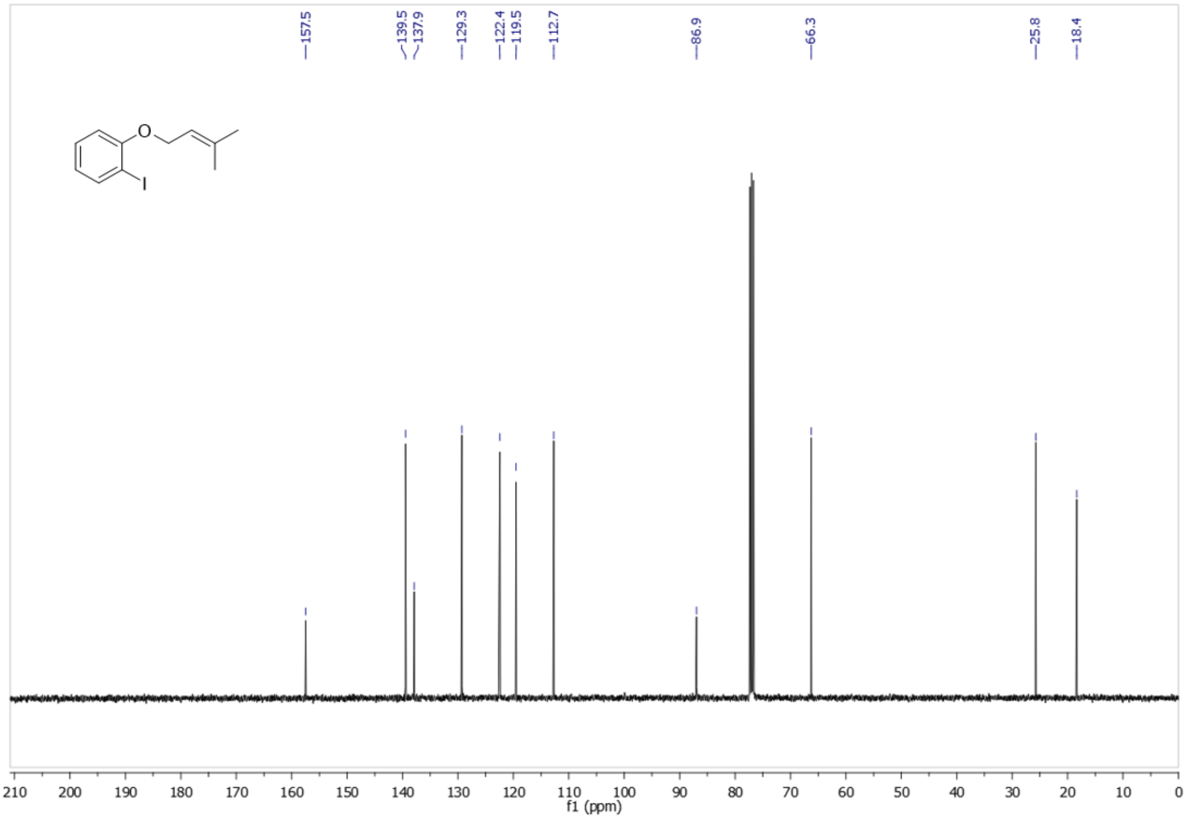

13.14 3-Isopropyl-2,3-dihydrobenzofuran (8)

^{1}H NMR (400 MHz, $CDCl_3$):

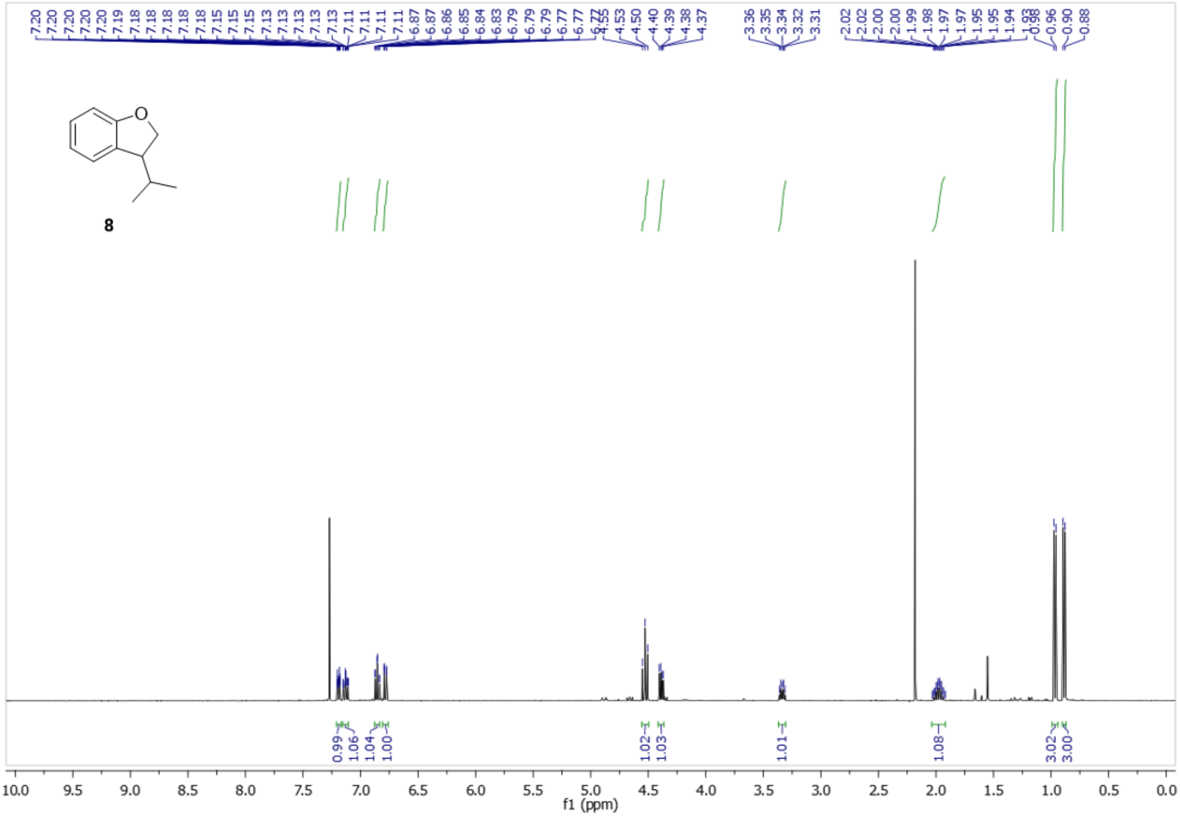

^{13}C NMR (101 MHz, $CDCl_3$):

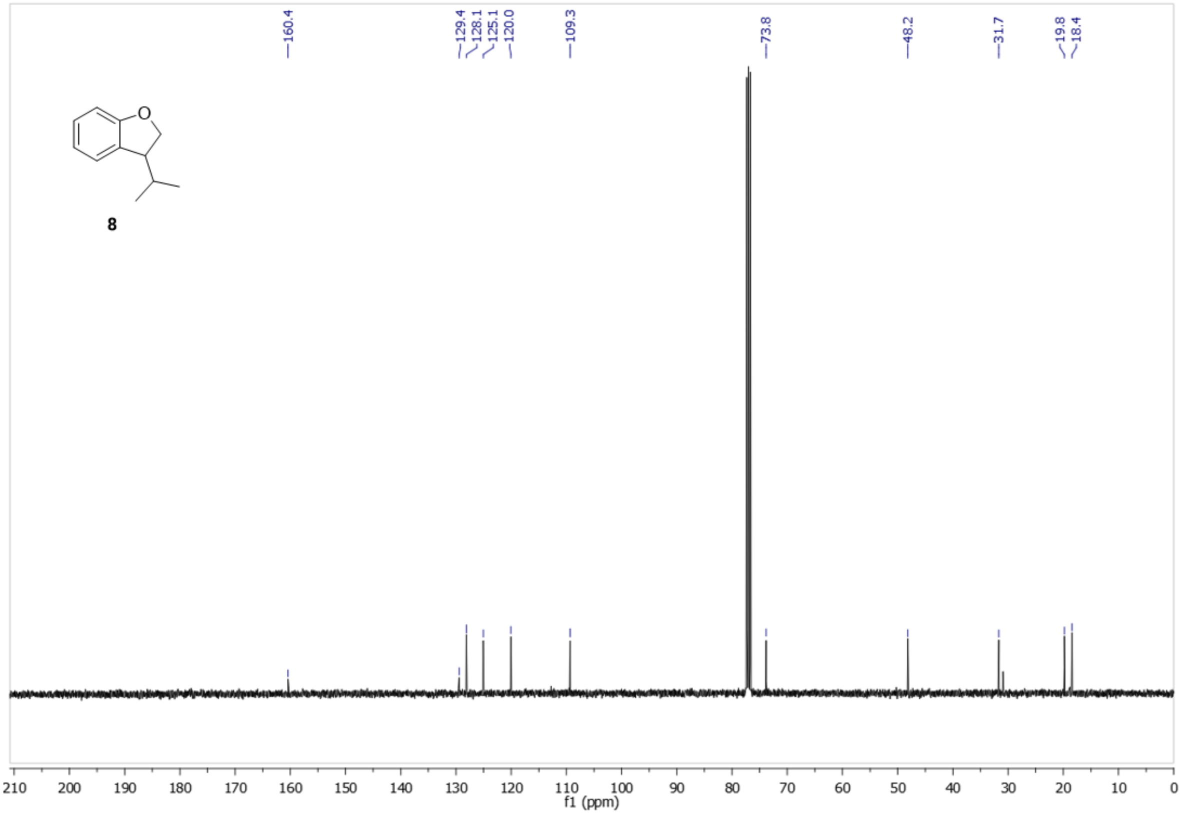

13.15 2-Iodo-3-((3-methylbut-2-en-1-yl)oxy)pyridine

^{1}H NMR (400 MHz, $CDCl_3$):

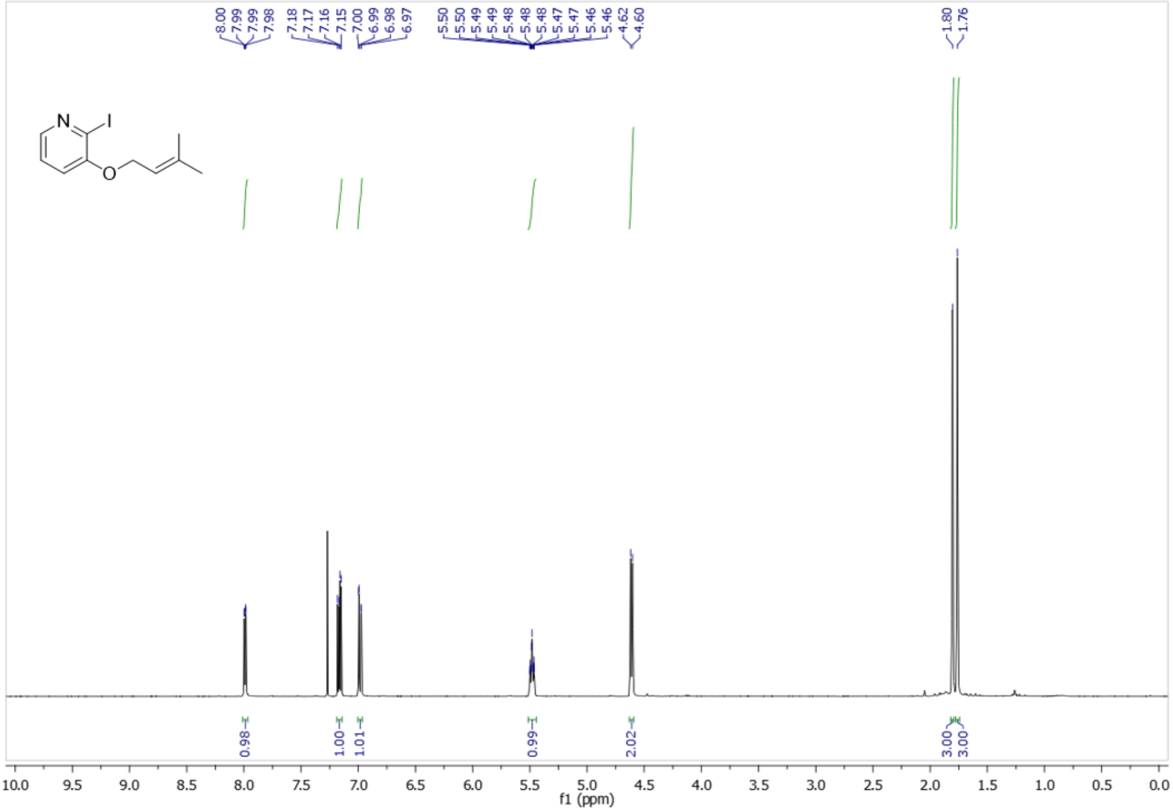

^{13}C NMR (101 MHz, $CDCl_3$):

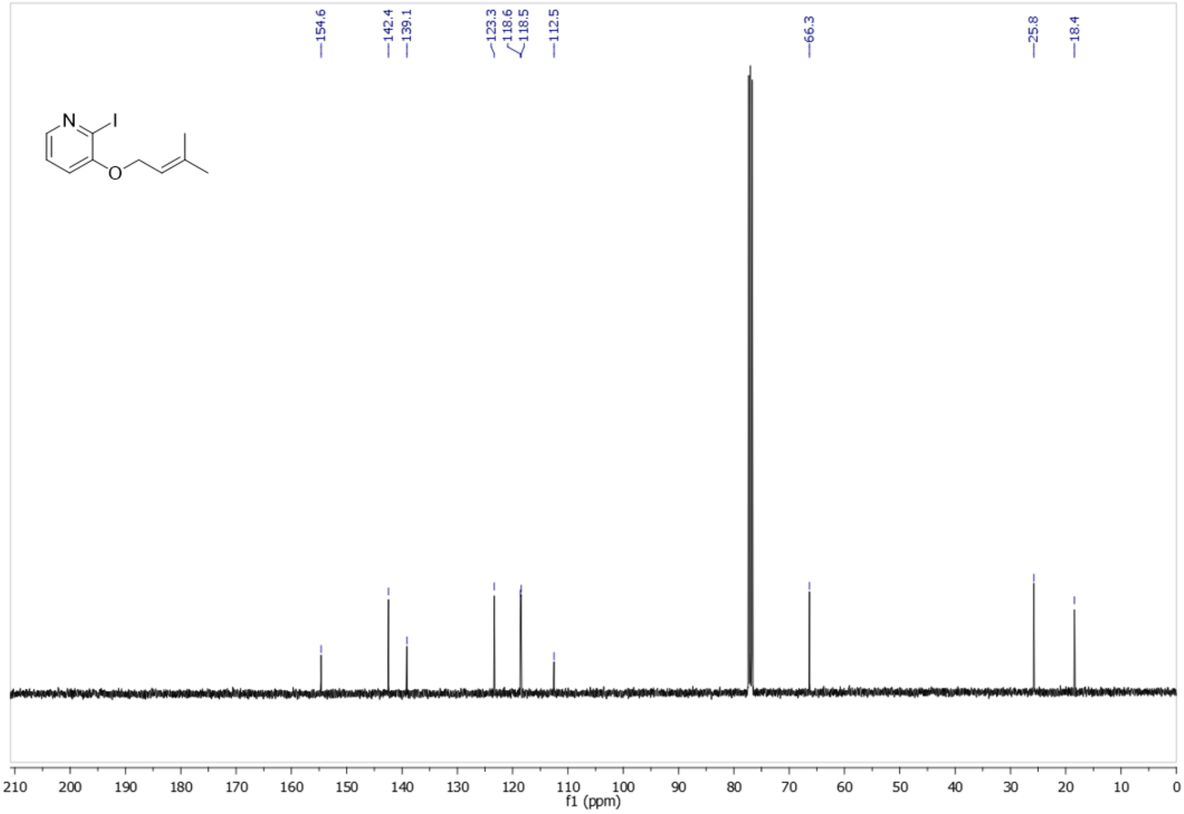

13.16 3-Isopropyl-2,3-dihydrofuro[3,2-b]pyridine (9)

^{1}H NMR (400 MHz, $CDCl_3$):

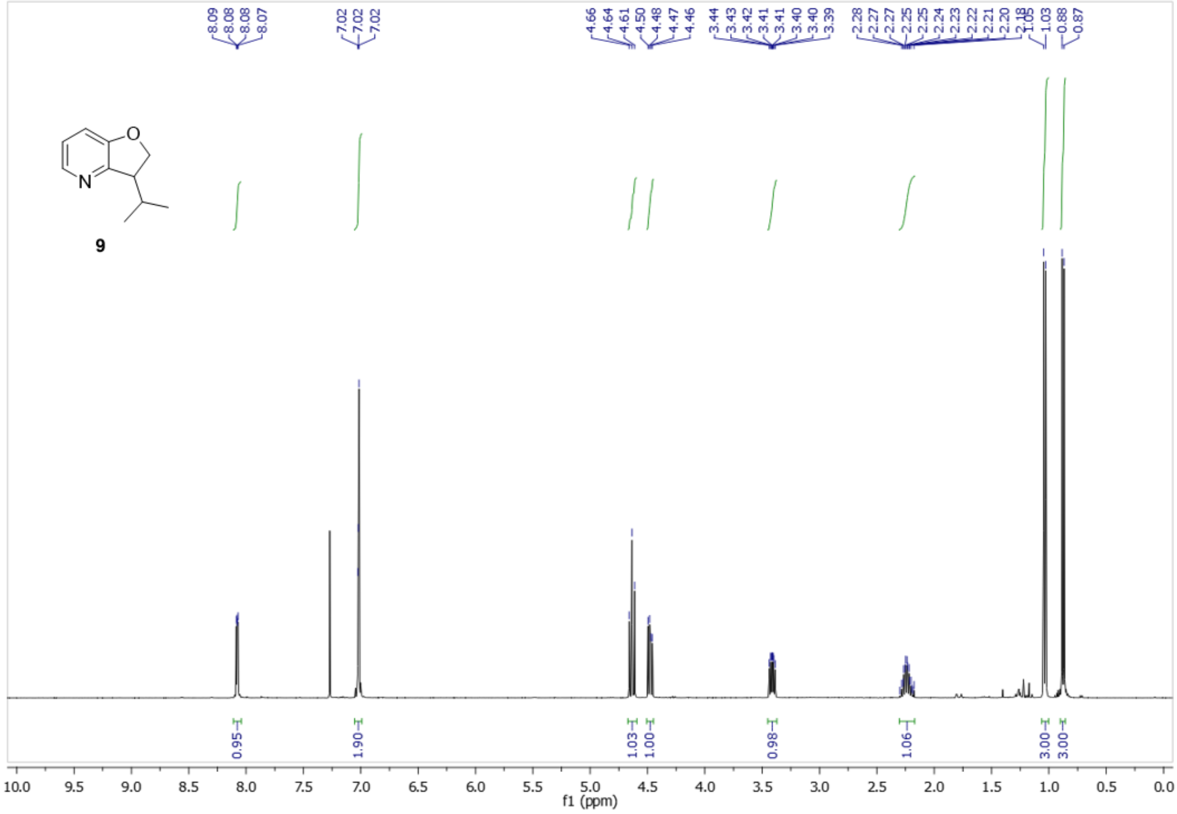

^{13}C NMR (101 MHz, $CDCl_3$):

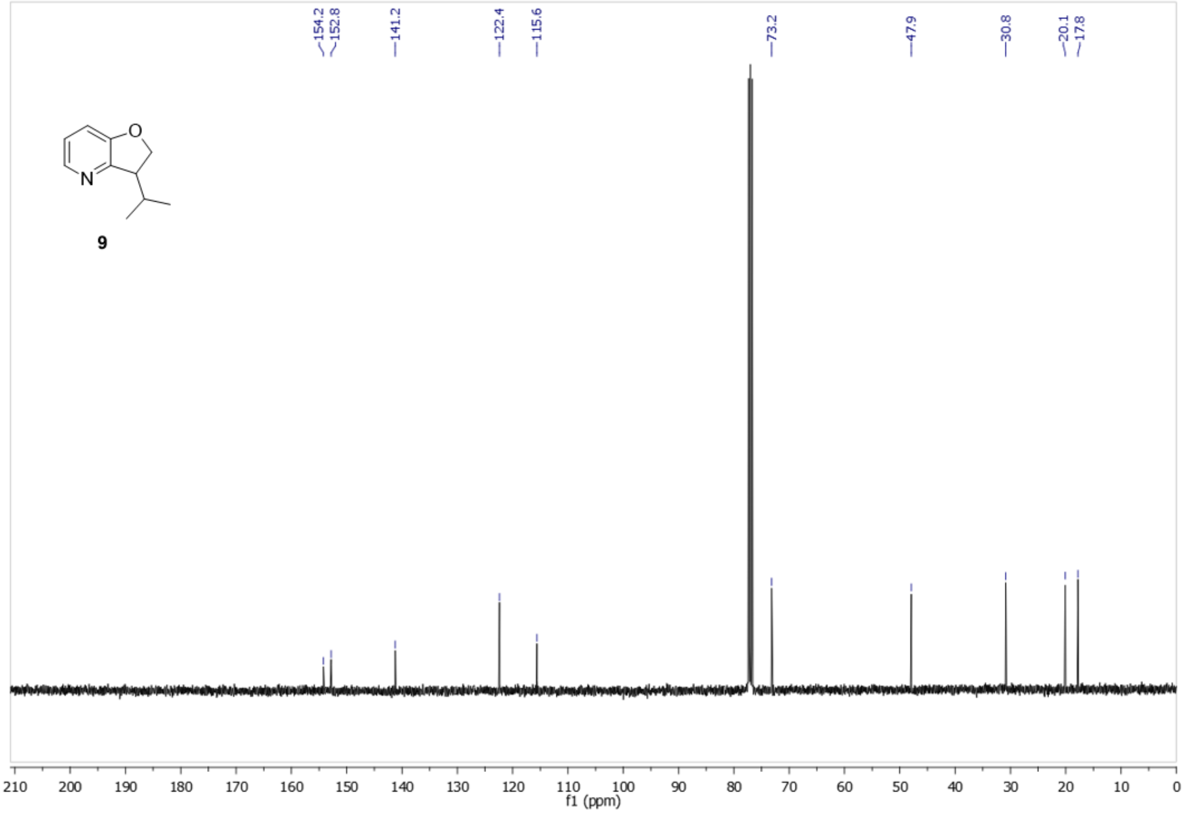

13.17 2-Iodo-3-((2-methylallyl)oxy)pyridine

^{1}H NMR (400 MHz, $CDCl_3$):

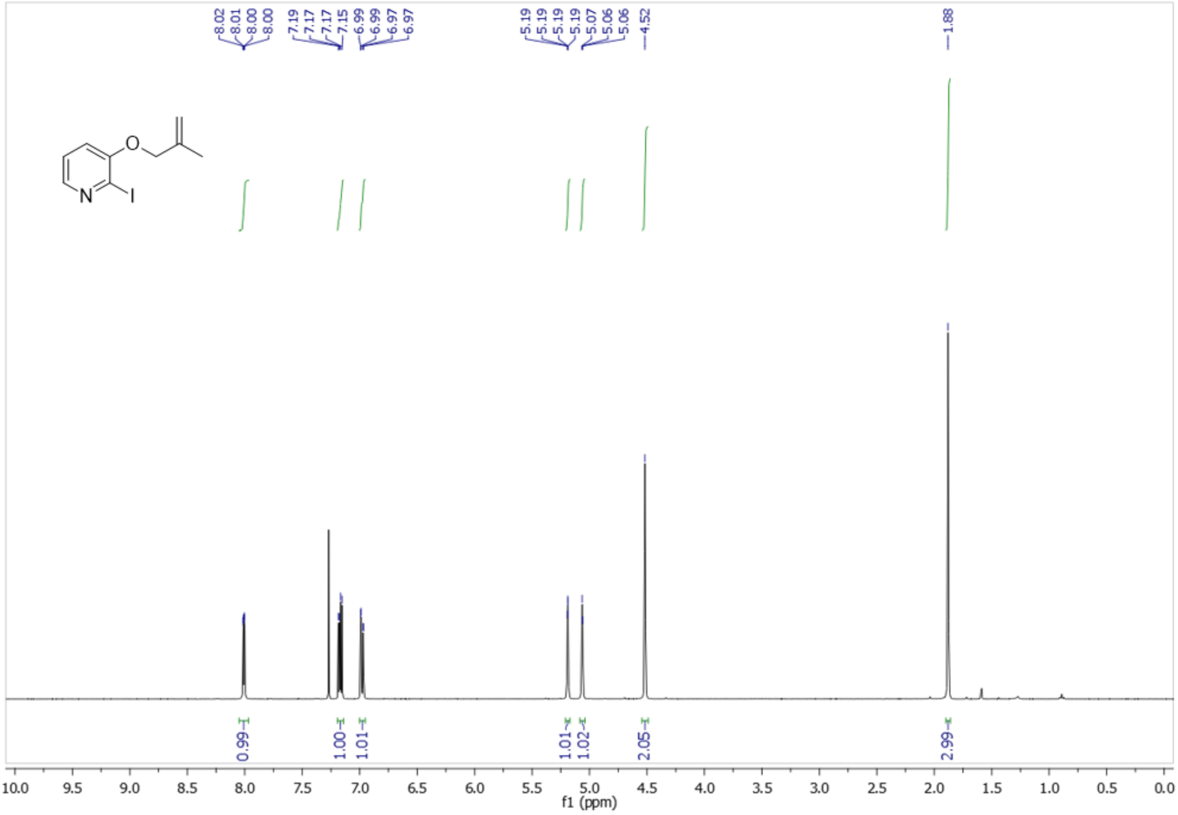

^{13}C NMR (101 MHz, $CDCl_3$):

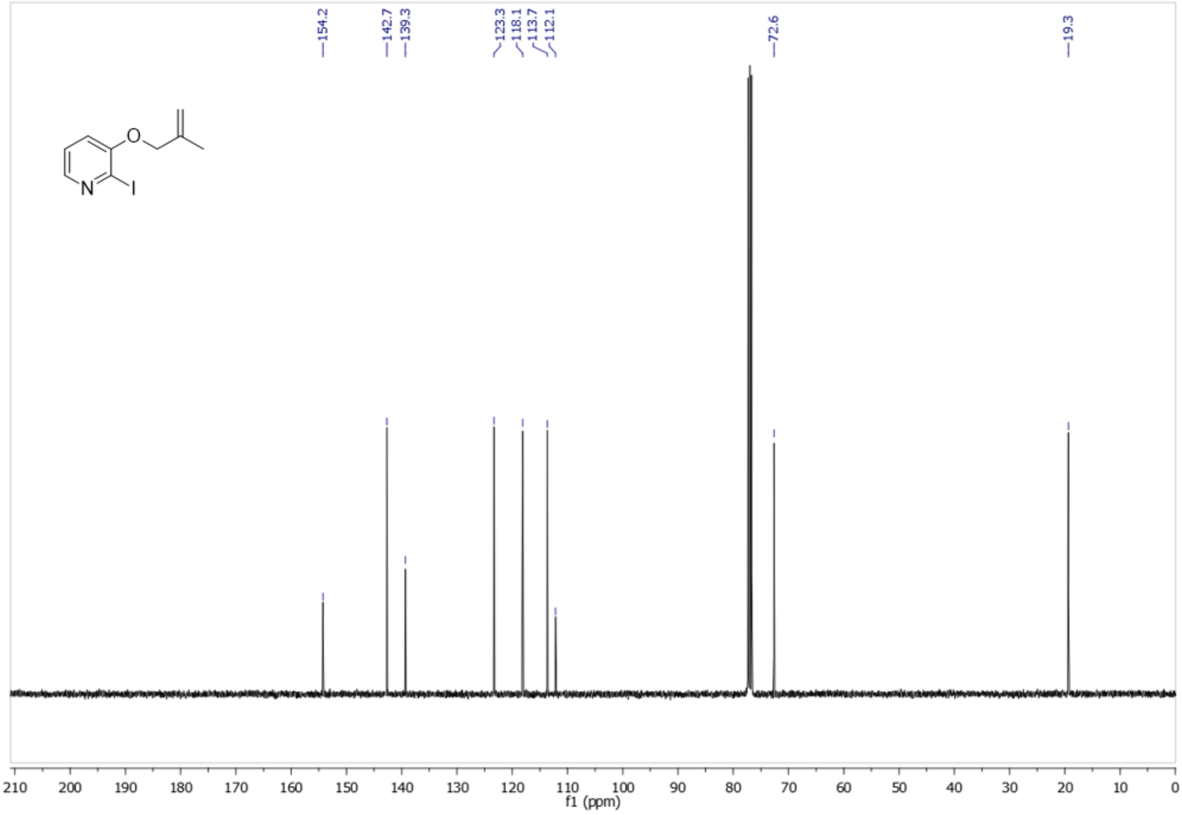

13.18 3,3-Dimethyl-2,3-dihydrofuro[3,2-*b*]pyridine (10)

^{1}H NMR (400 MHz, $CDCl_3$):

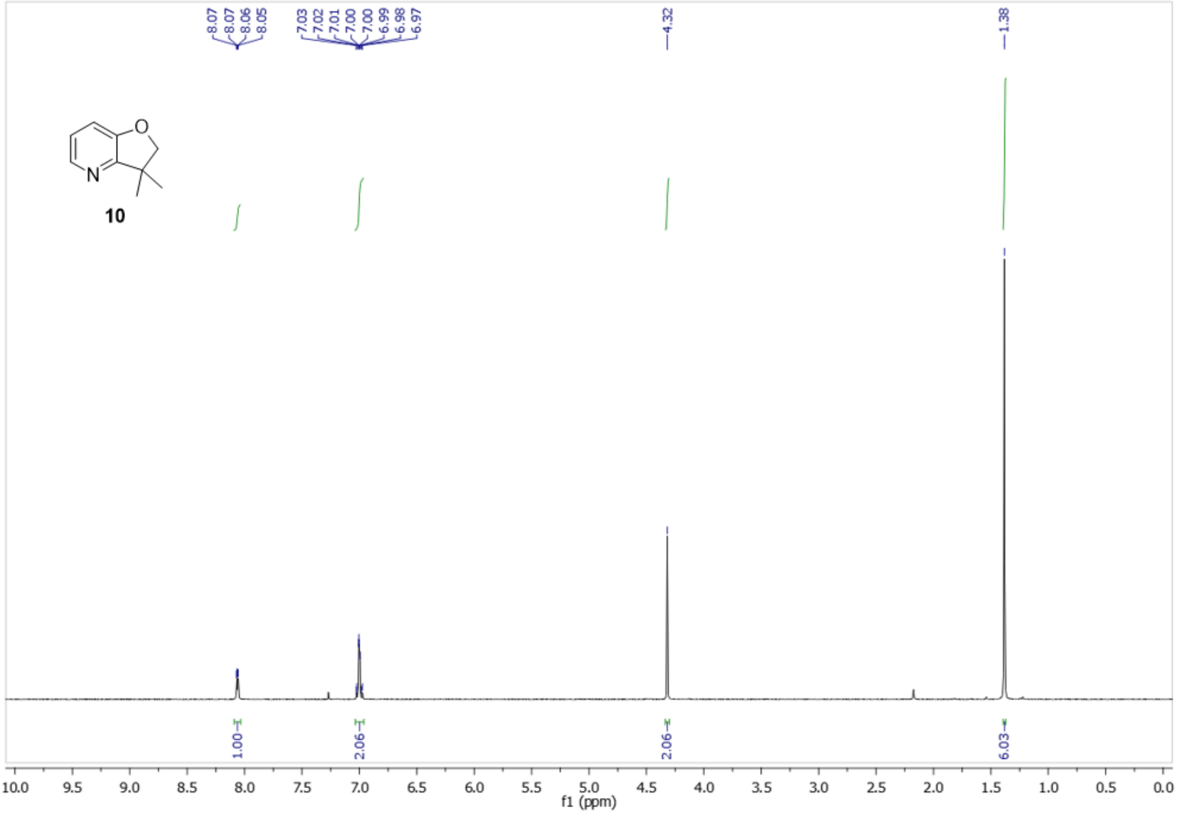

^{13}C NMR (101 MHz, $CDCl_3$):

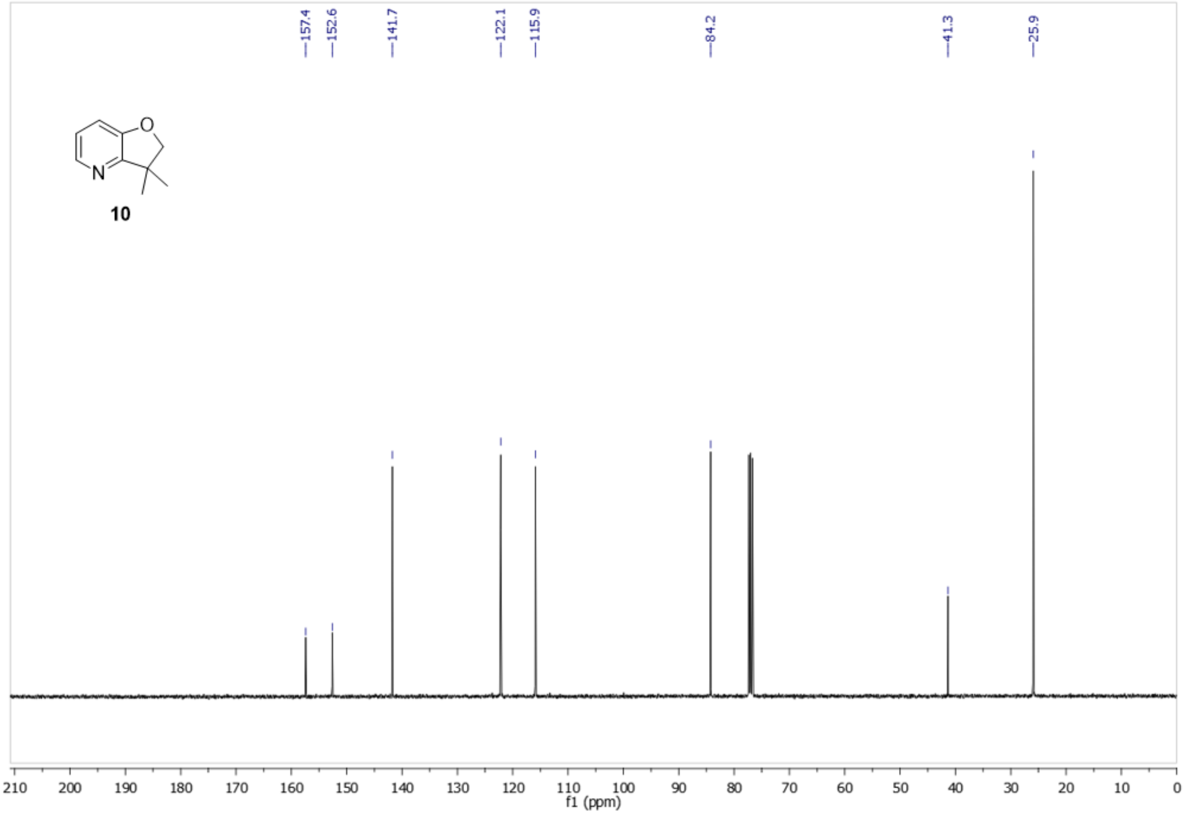

13.19 1-(Cinnamyloxy)-2-iodobenzene (*E:Z* ~ 14:1)

^{1}H NMR (400 MHz, $CDCl_3$):

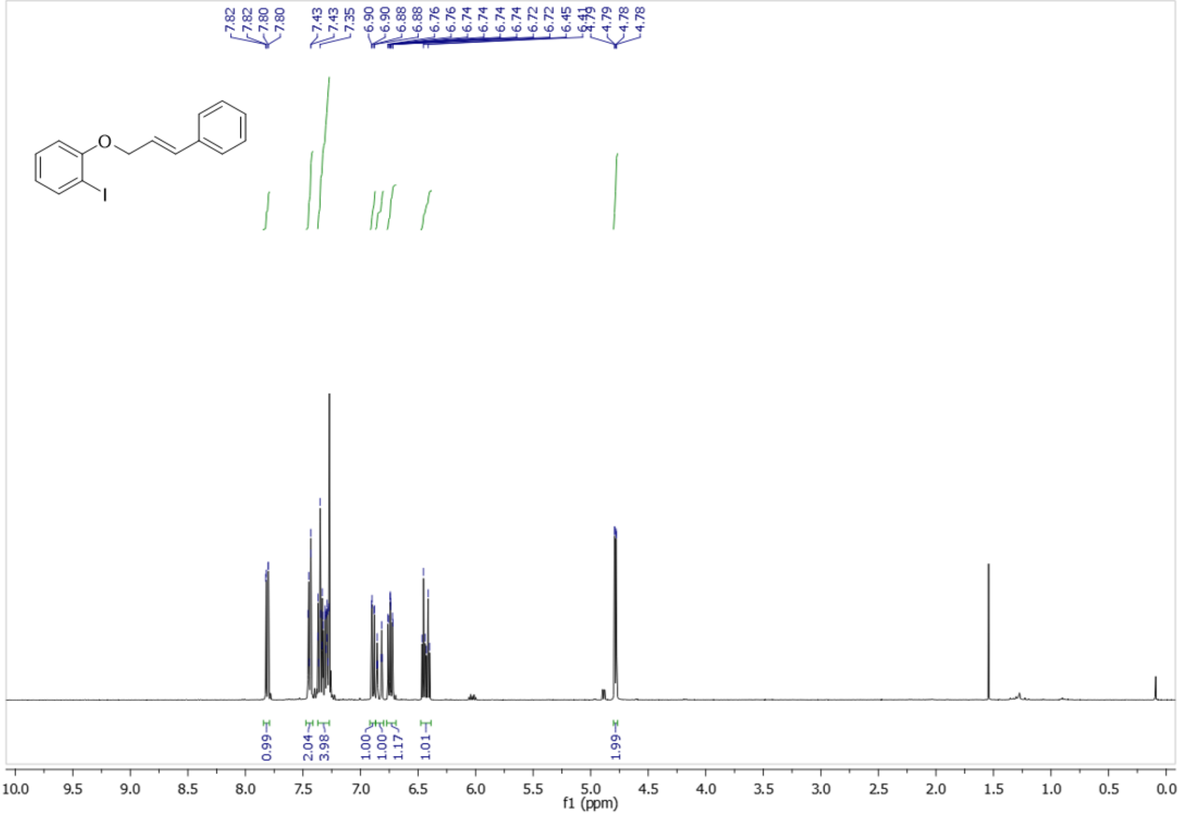

^{13}C NMR (101 MHz, $CDCl_3$):

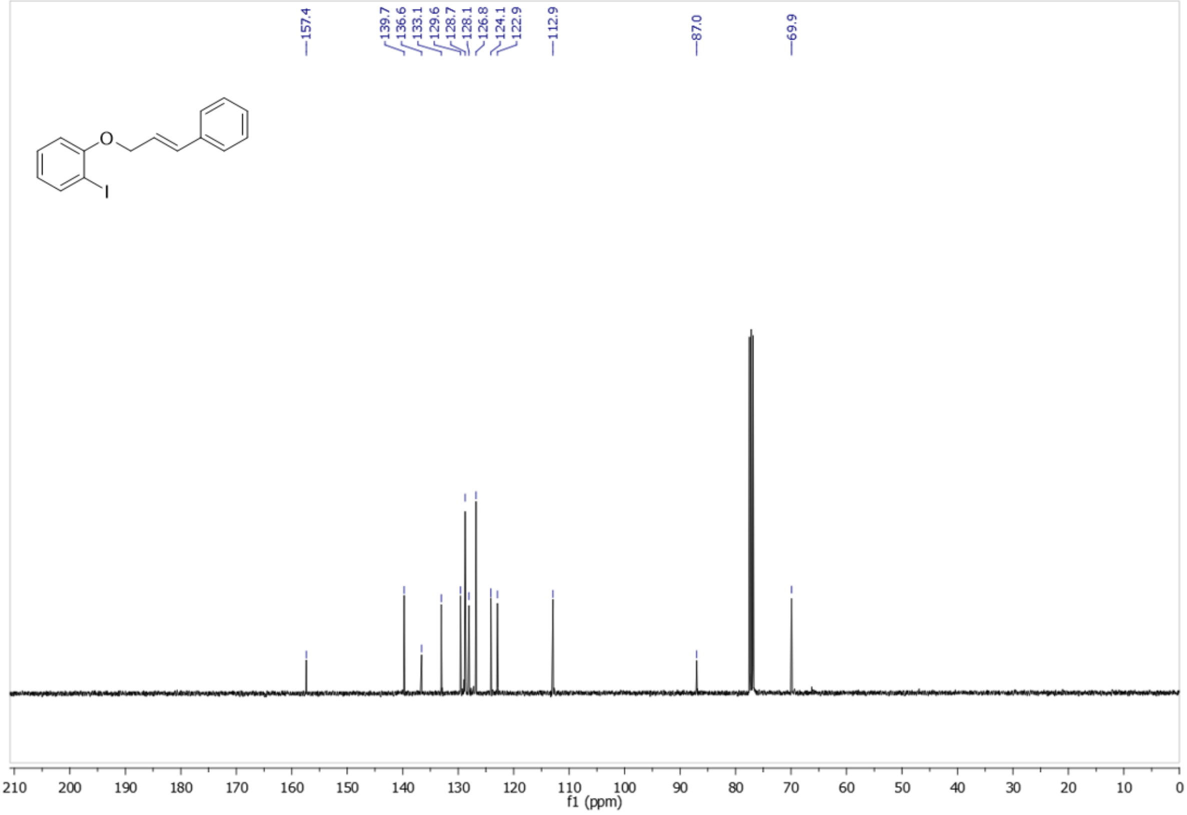

13.20 1-Chloro-2-(cinnamyloxy)benzene

^{1}H NMR (400 MHz, $CDCl_3$):

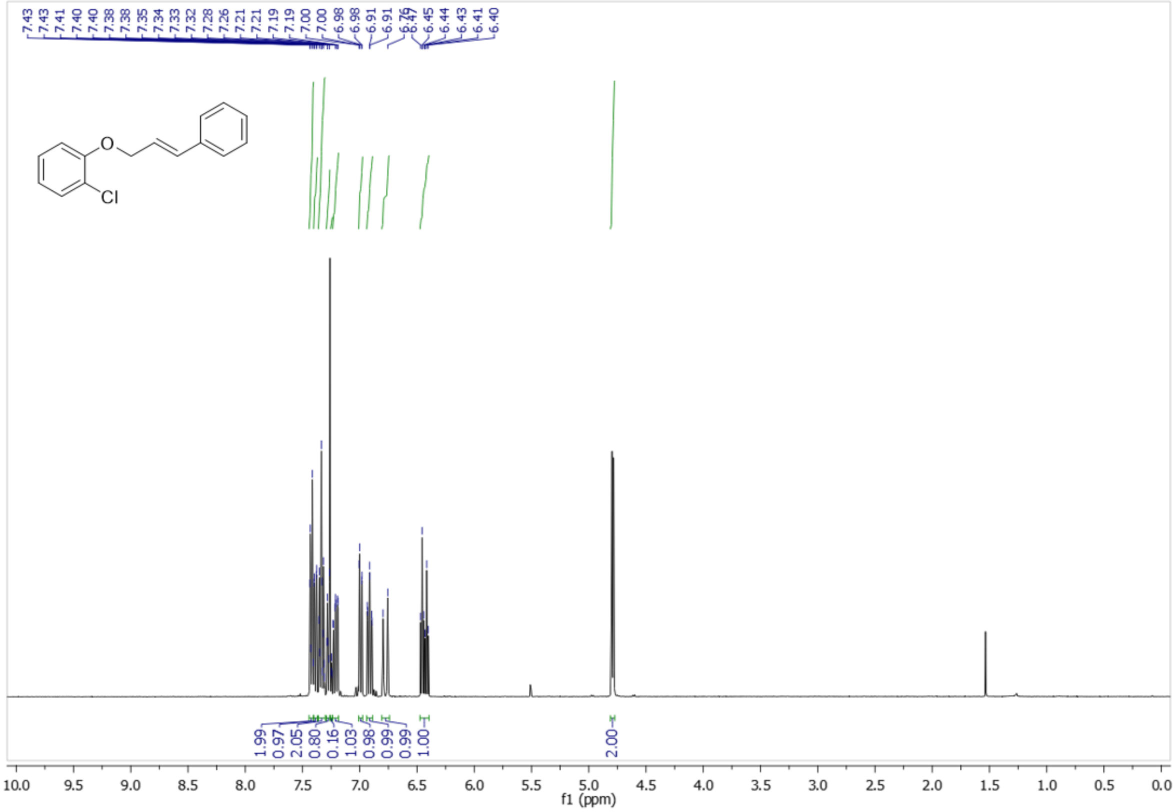

^{13}C NMR (101 MHz, $CDCl_3$):

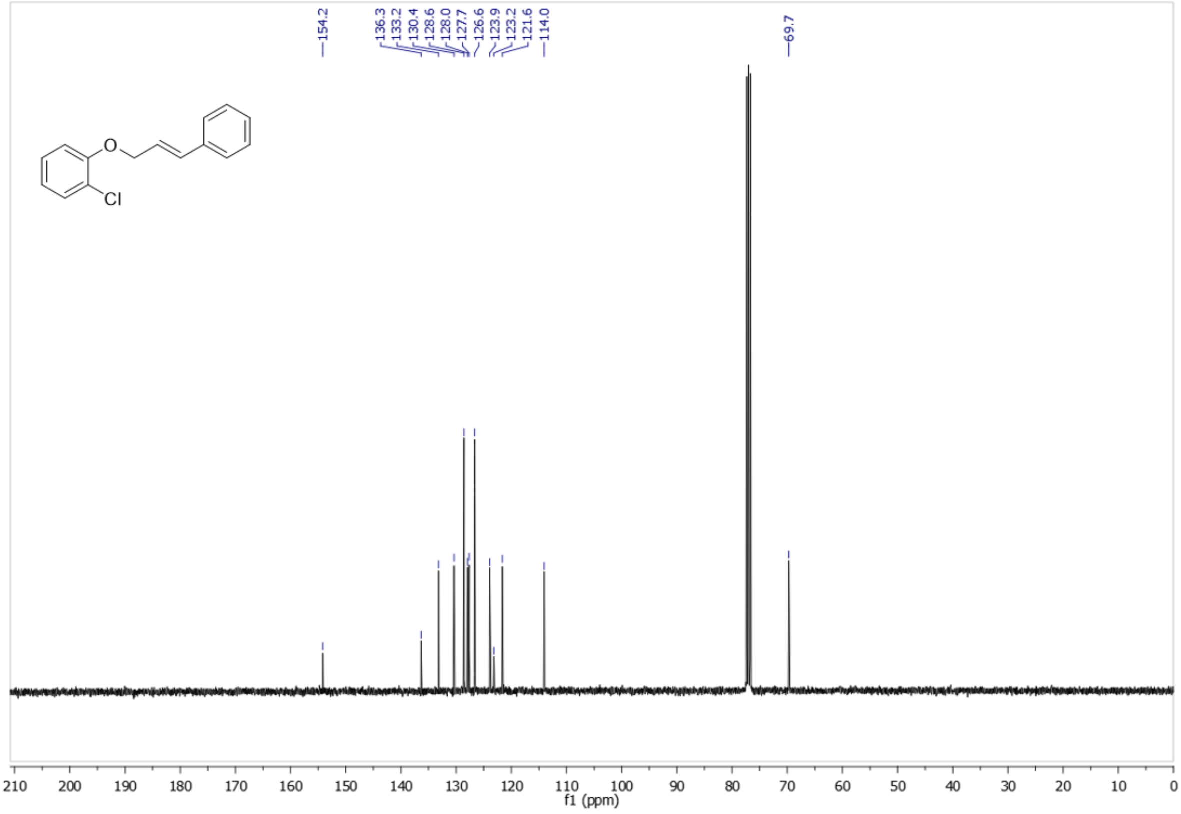

13.21 3-Benzyl-2,3-dihydrobenzofuran (11)

^{1}H NMR (400 MHz, $CDCl_3$):

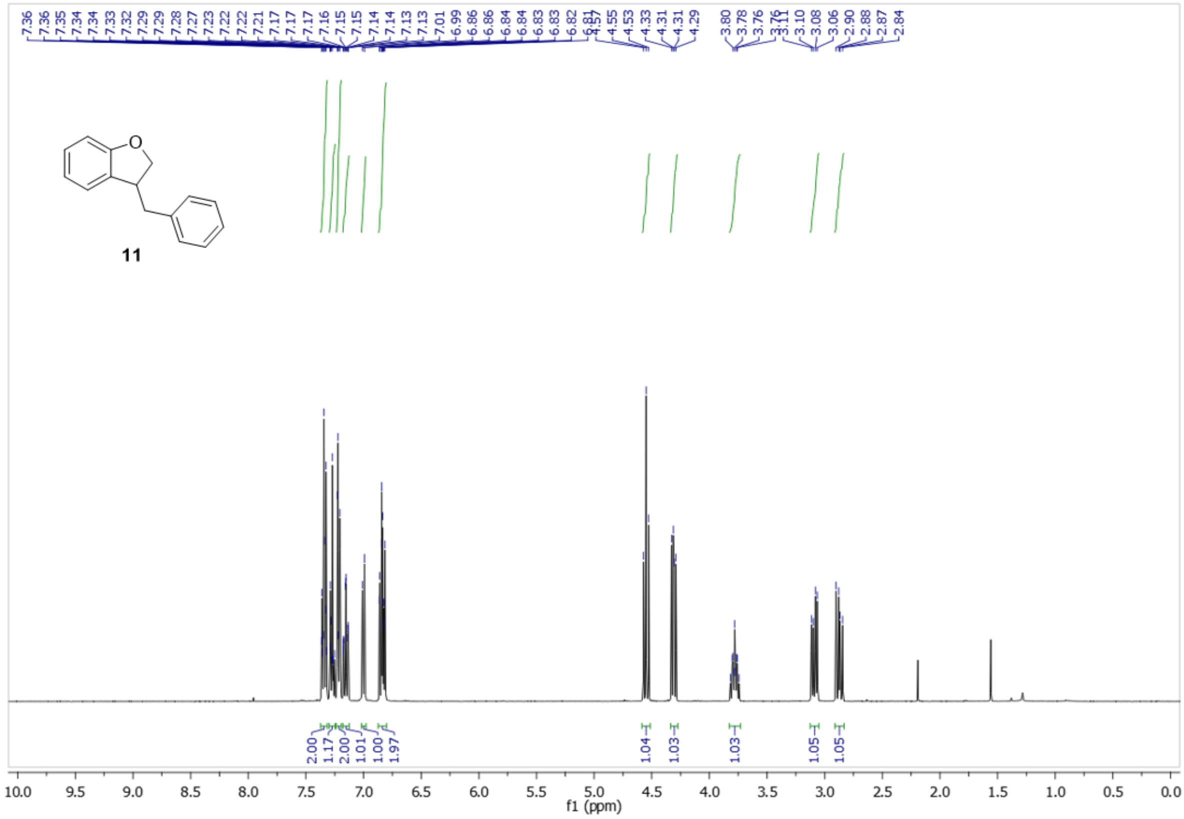

^{13}C NMR (101 MHz, $CDCl_3$):

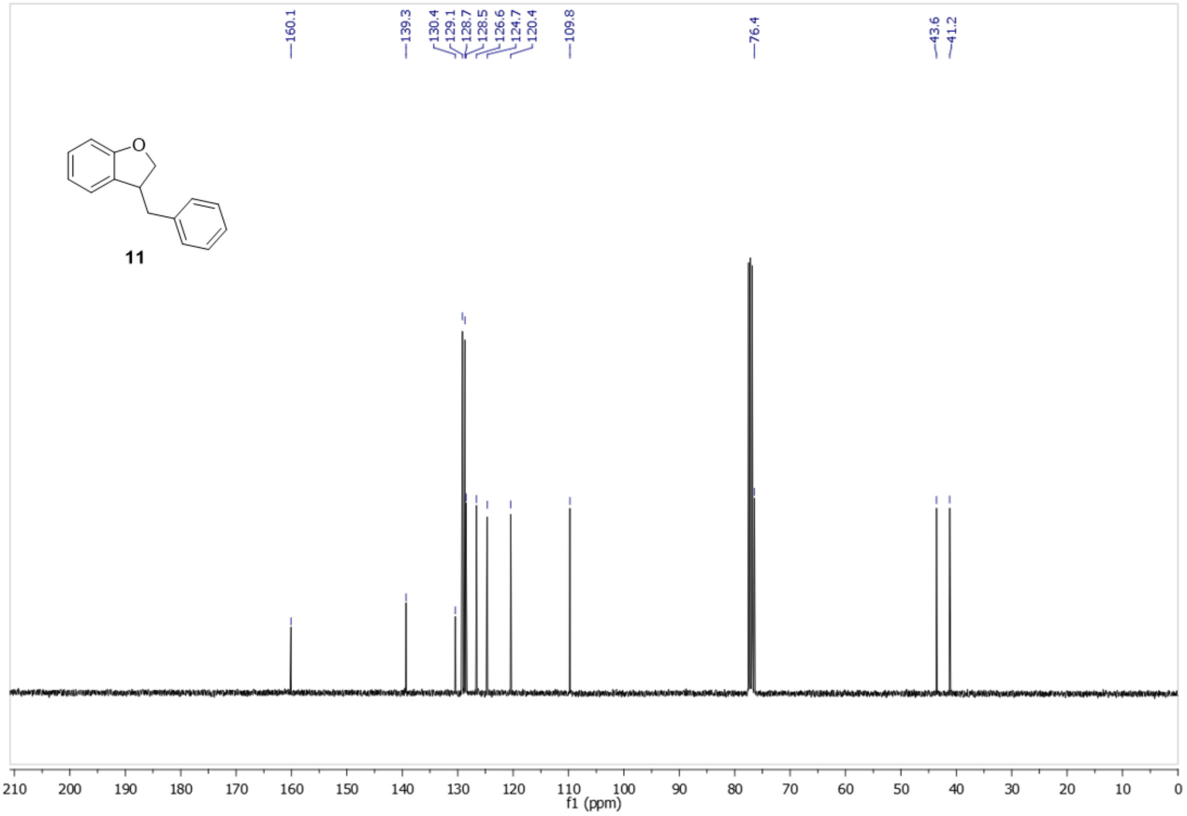

13.22 3-Bromo-4-(cyclohex-2-en-1-yloxy)benzonitrile

^{1}H NMR (400 MHz, $CDCl_3$):

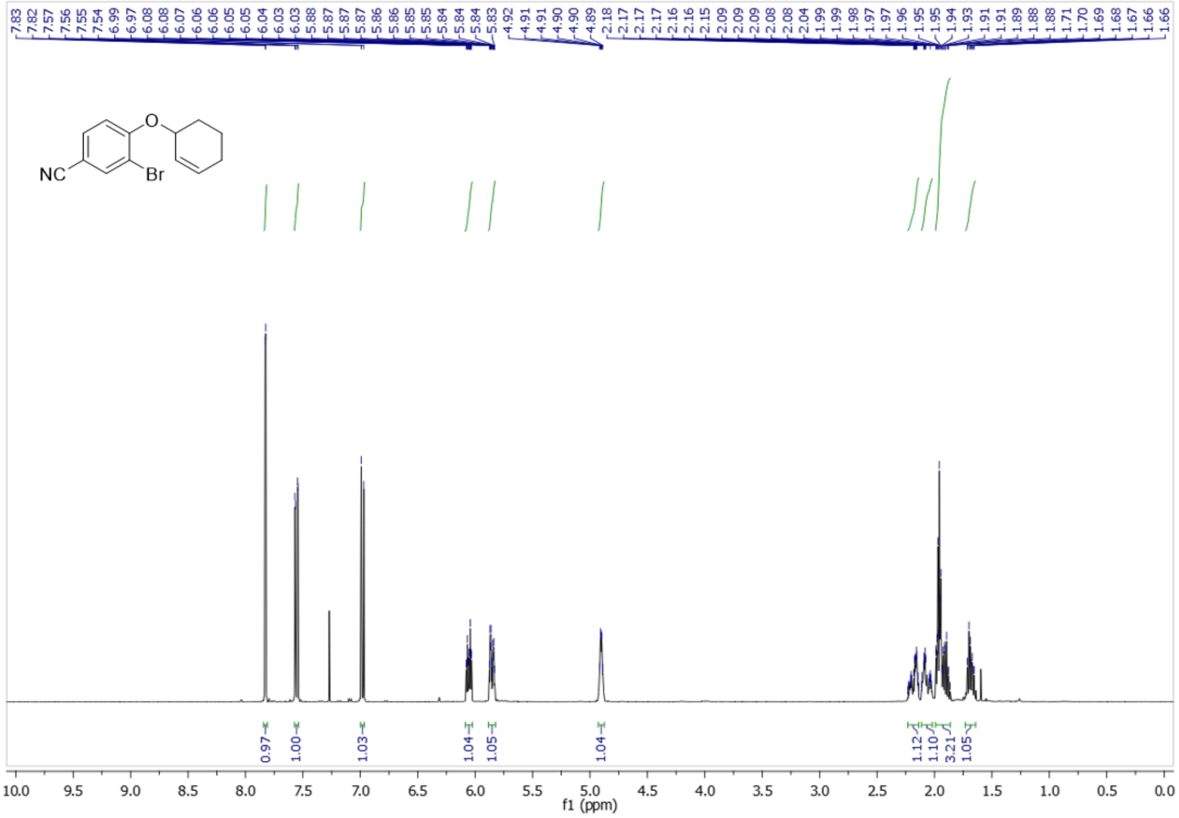

^{13}C NMR (101 MHz, $CDCl_3$):

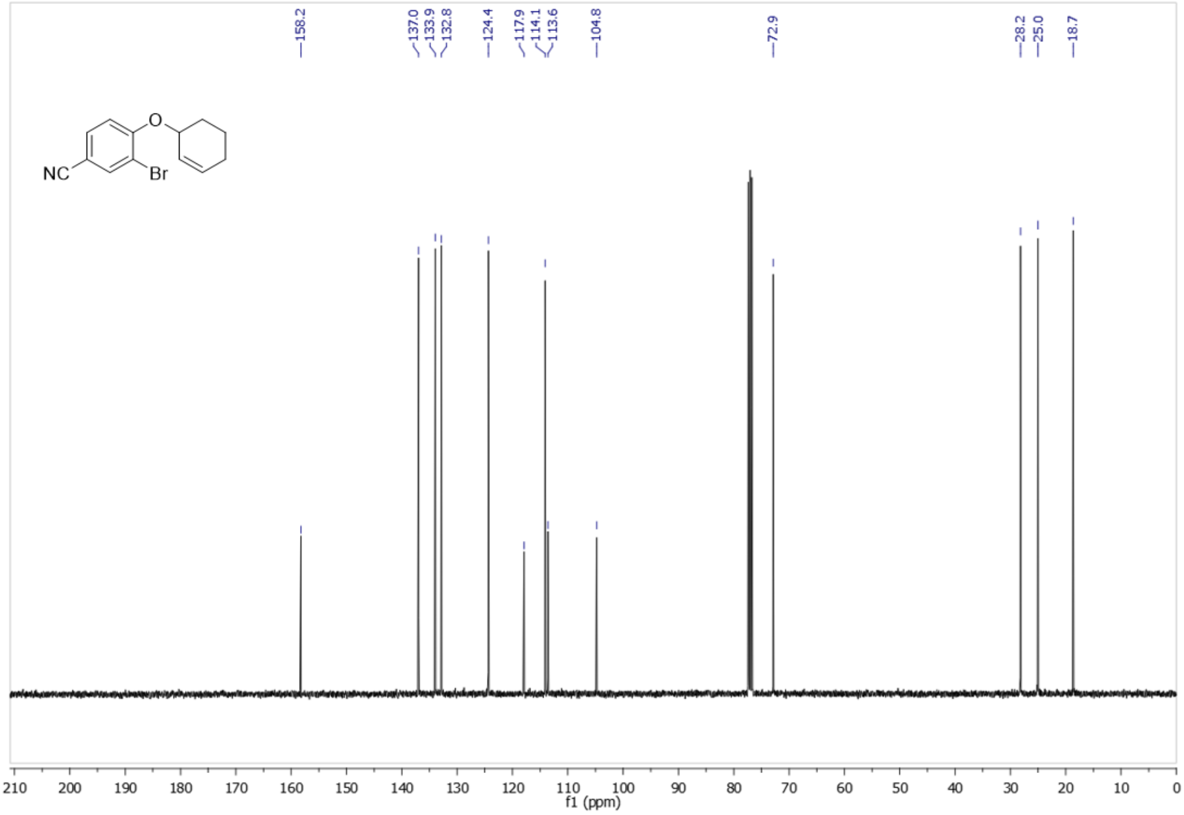

13.23 5a,6,7,8,9,9a-Hexahydrodibenzo[*b*,*d*]furan-2-carbonitrile (12)

^{1}H NMR (400 MHz, $CDCl_3$):

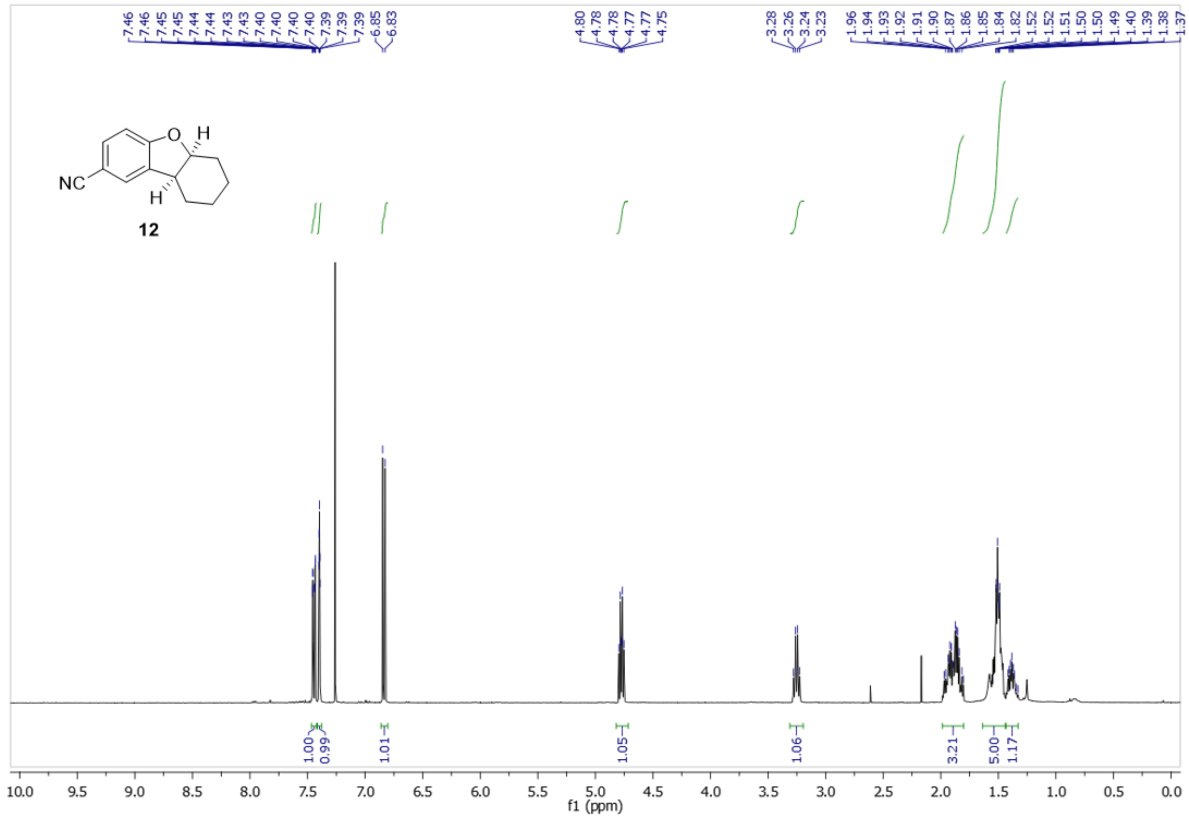

^{13}C NMR (101 MHz, $CDCl_3$):

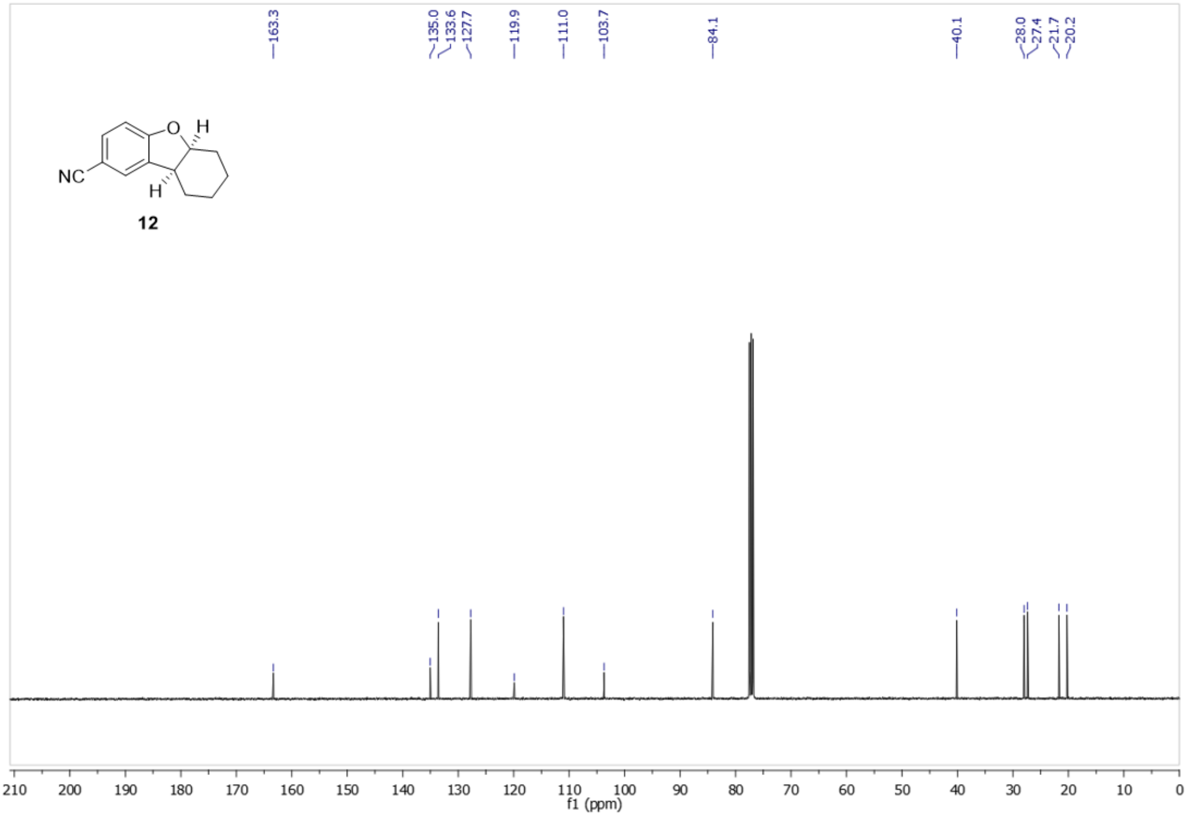

13.24 3-(Cyclohex-2-en-1-yloxy)-2-iodopyridine

^{1}H NMR (400 MHz, $CDCl_3$):

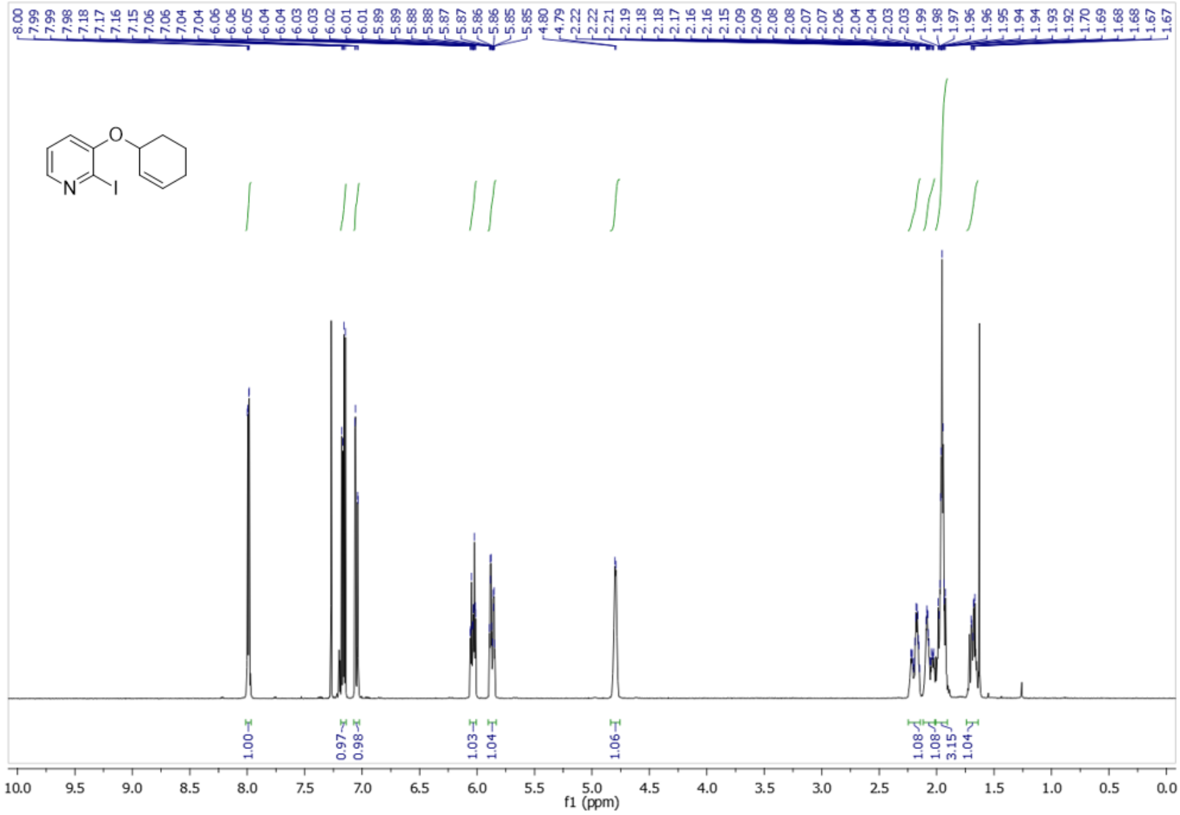

^{13}C NMR (101 MHz, $CDCl_3$):

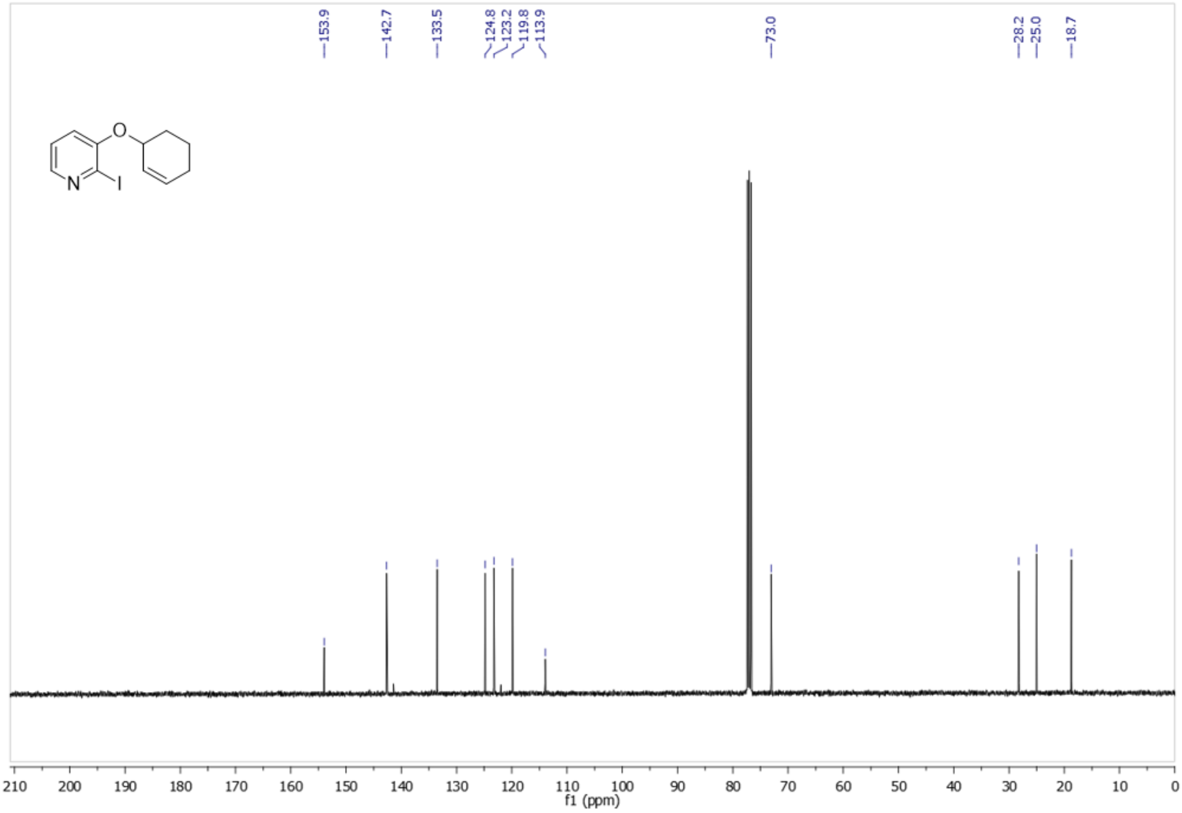

13.25 (5a*S*,9a*S*)-5a,6,7,8,9,9a-Hexahydrobenzofuro[3,2-*b*]pyridine (13)

^{1}H NMR (400 MHz, $CDCl_3$):

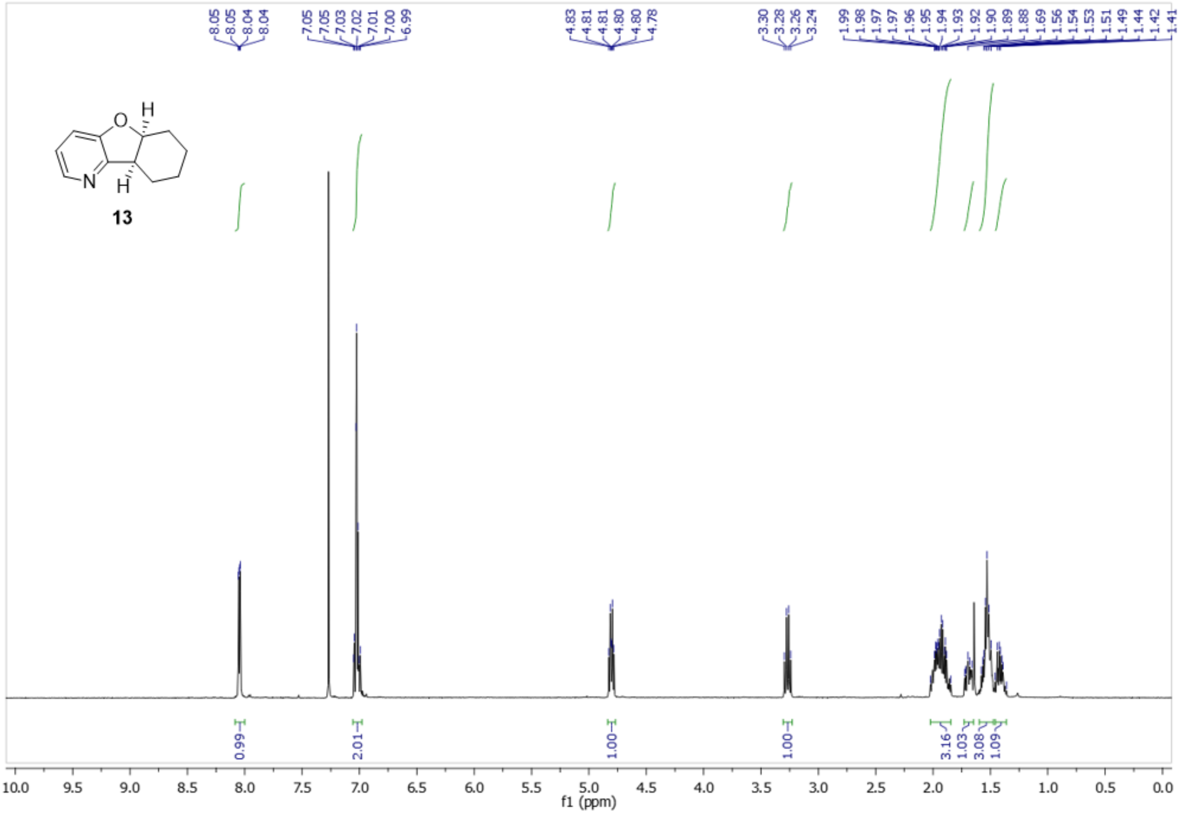

^{13}C NMR (101 MHz, $CDCl_3$):

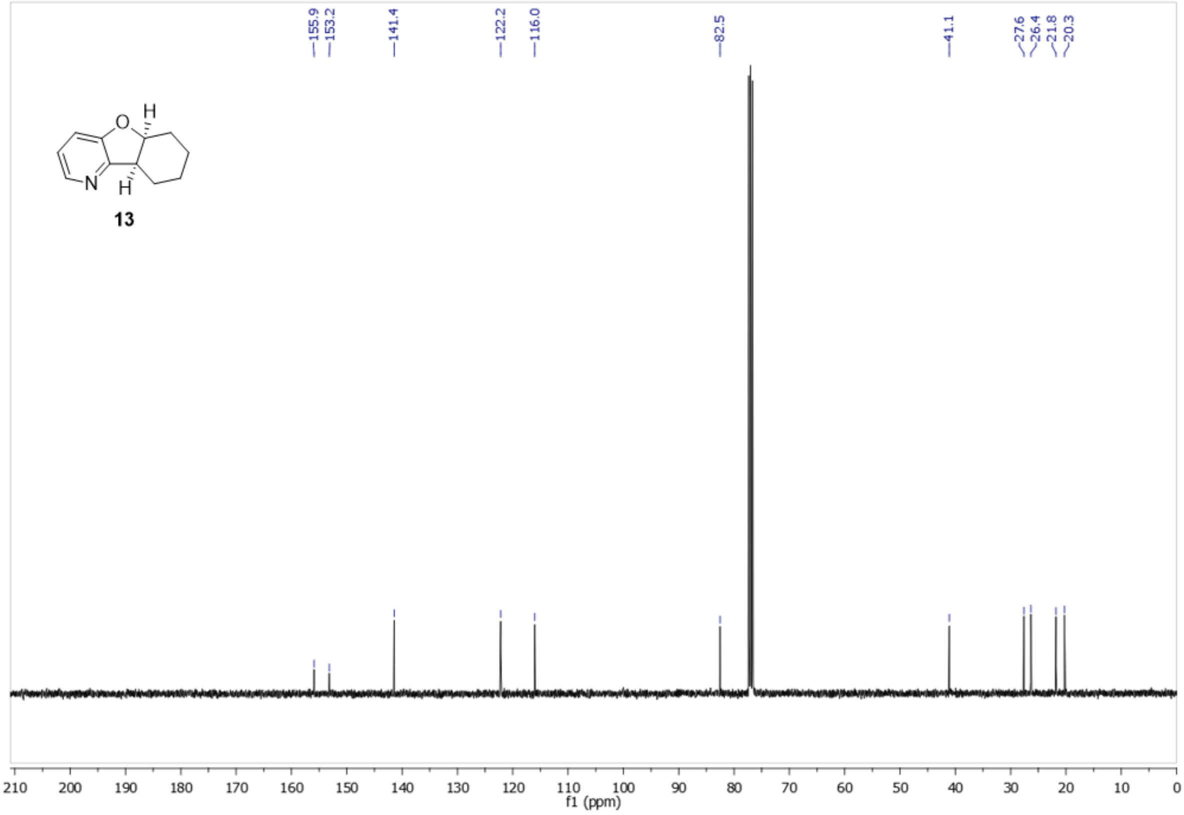

13.26 1-(Cyclohex-2-en-1-yloxy)-2-iodobenzene

^{1}H NMR (400 MHz, $CDCl_3$):

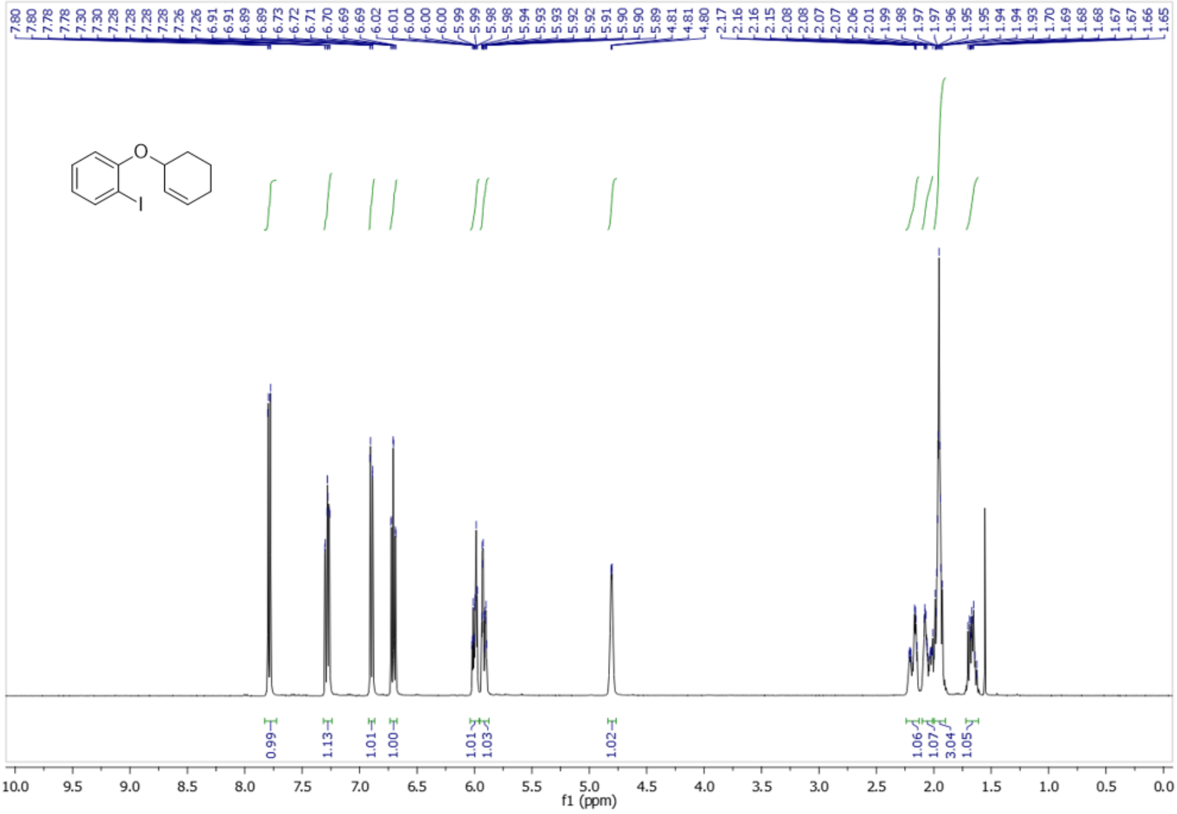

^{13}C NMR (101 MHz, $CDCl_3$):

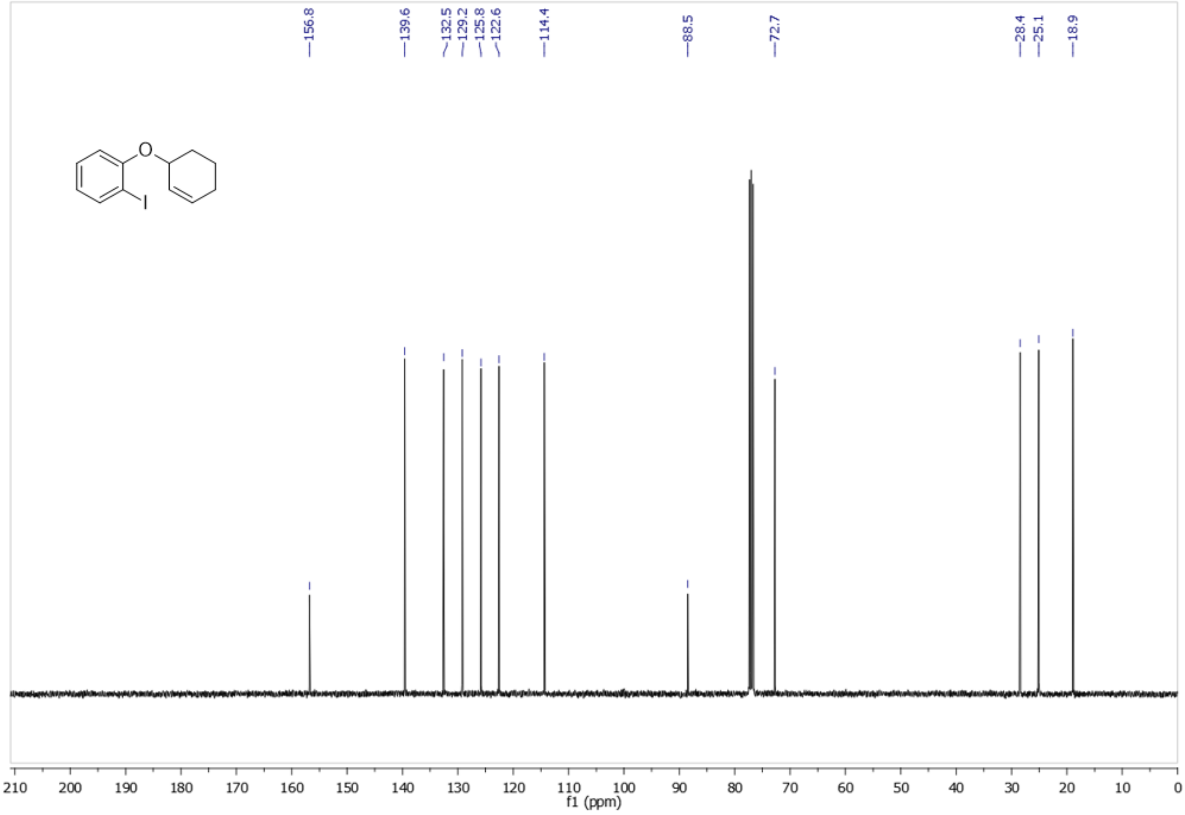

13.27 1-Chloro-2-(cyclohex-2-en-1-yloxy)benzene

^{1}H NMR (400 MHz, $CDCl_3$):

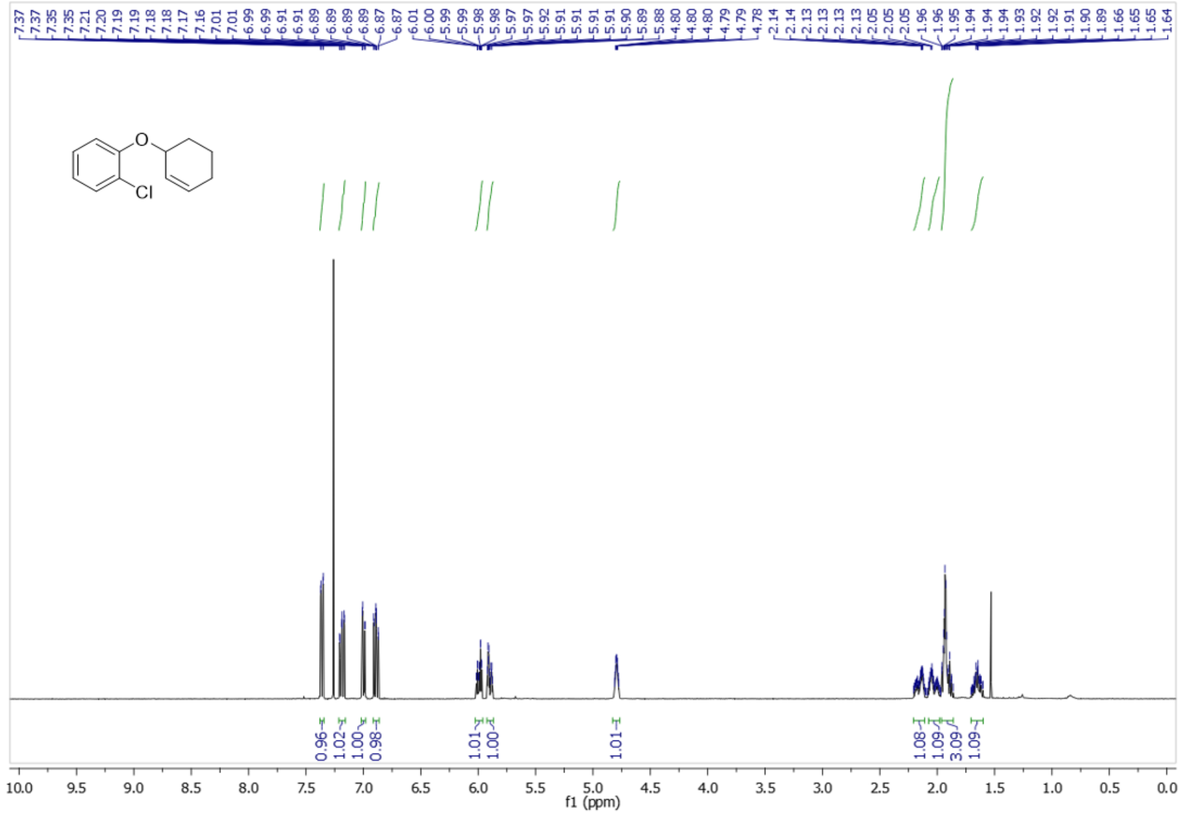

^{13}C NMR (101 MHz, $CDCl_3$):

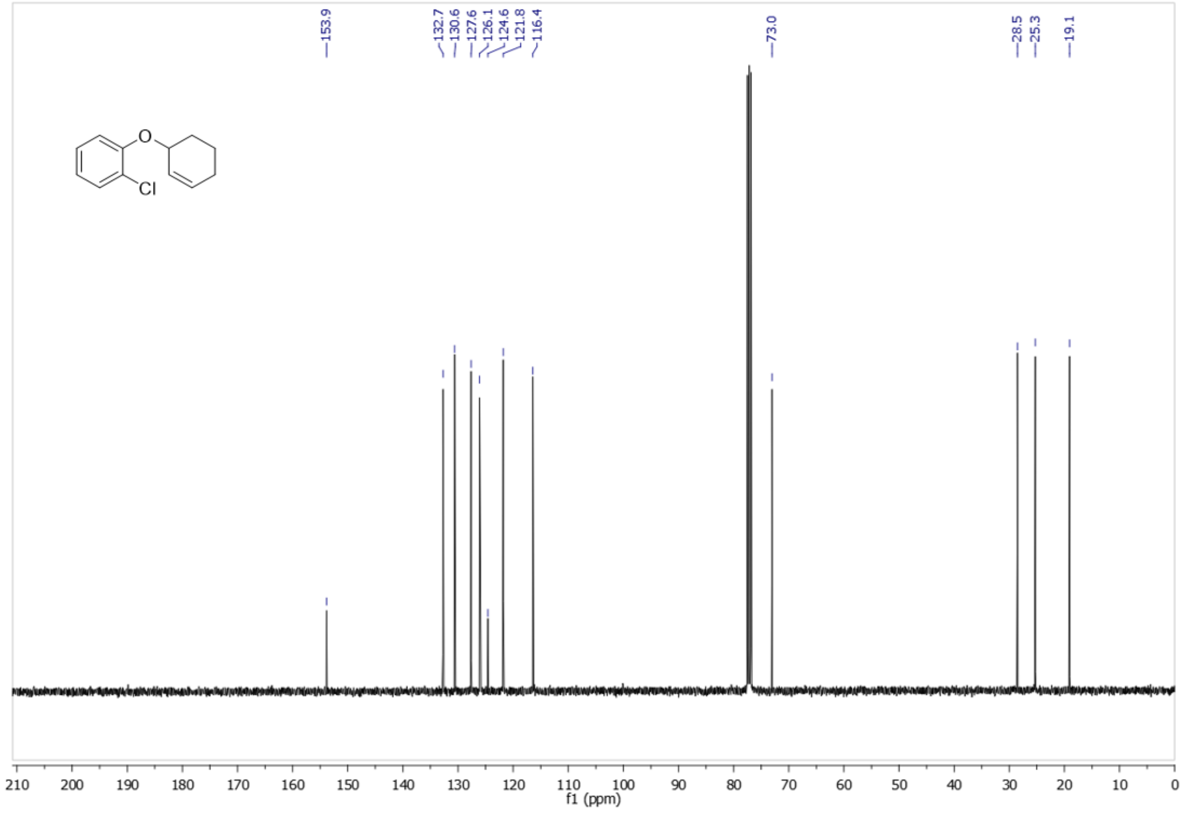

13.28 (4aS,9bS)-1,2,3,4,4a,9b-Hexahydrodibenzo[*b*,*d*]furan (14)

^{1}H NMR (400 MHz, $CDCl_3$):

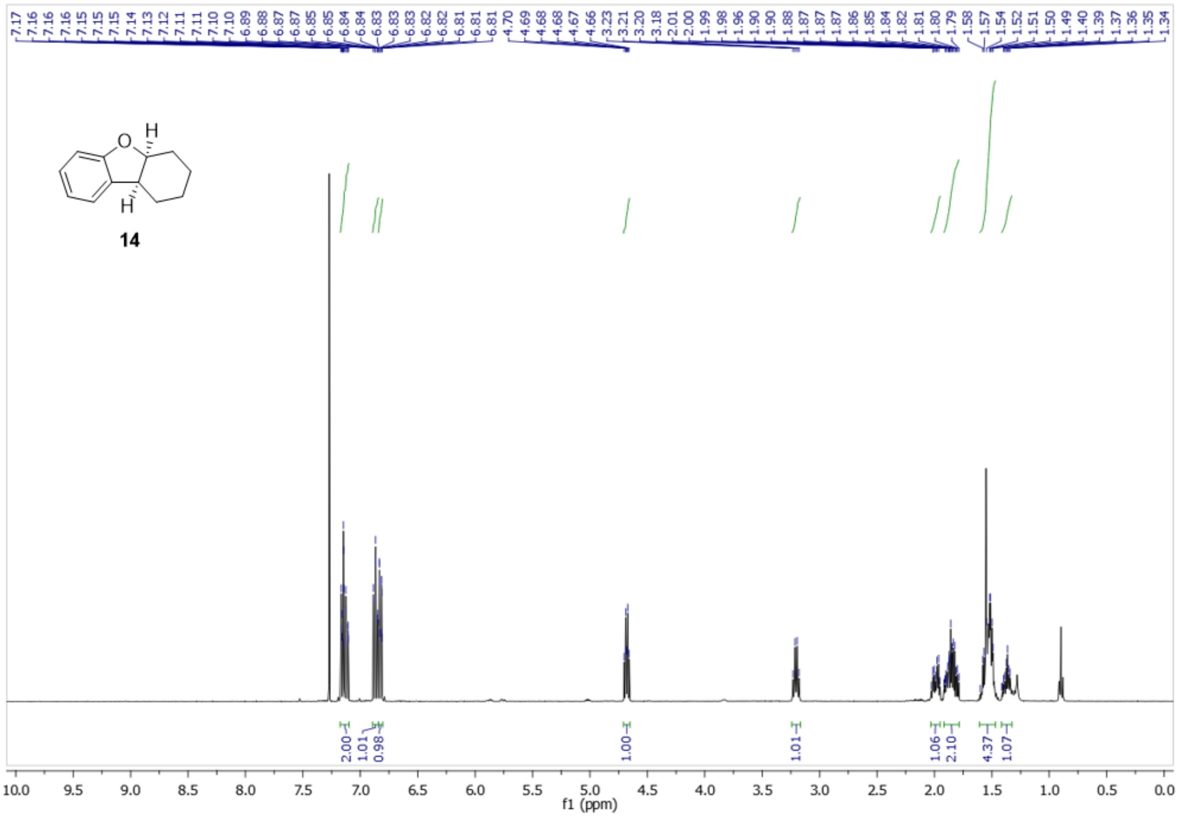

^{13}C NMR (101 MHz, $CDCl_3$):

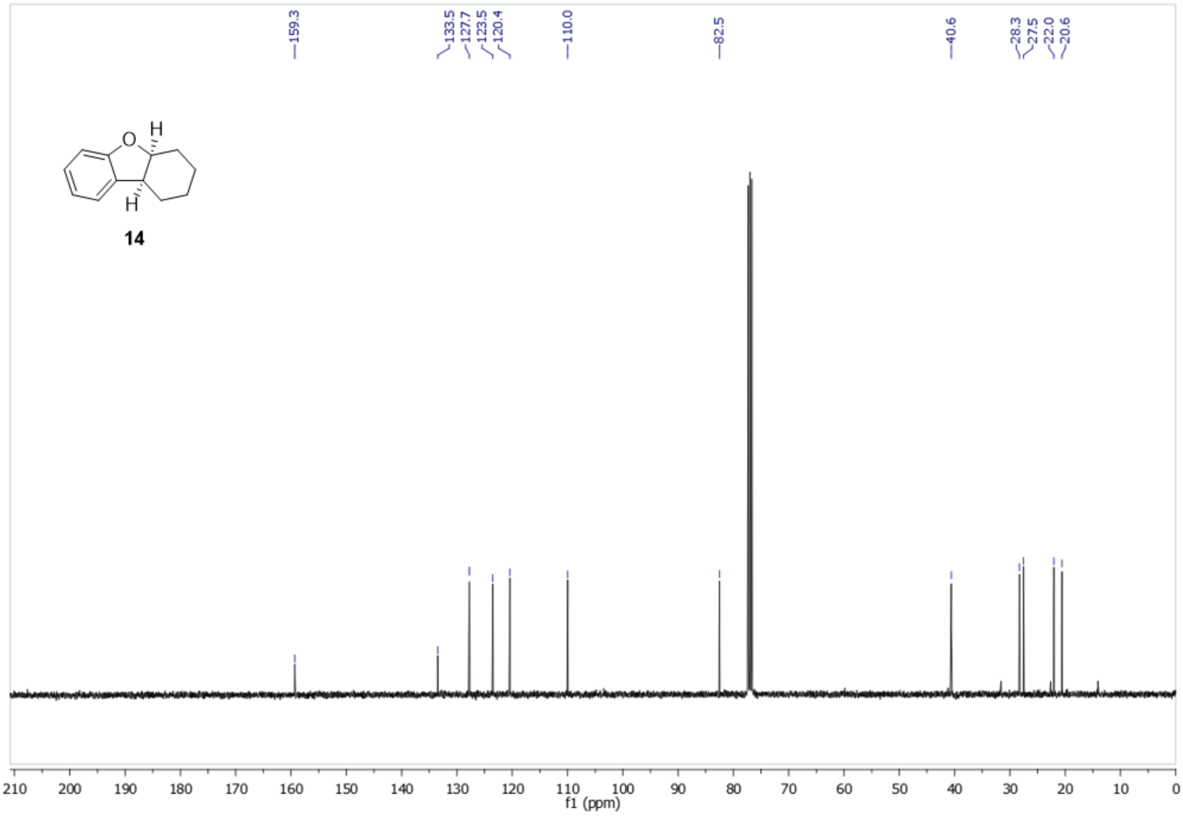

13.29 *tert*-Butyl 4-hydroxy-4-((phenylsulfinyl)methyl)piperidine-1-carboxylate

^{1}H NMR (400 MHz, $CDCl_3$):

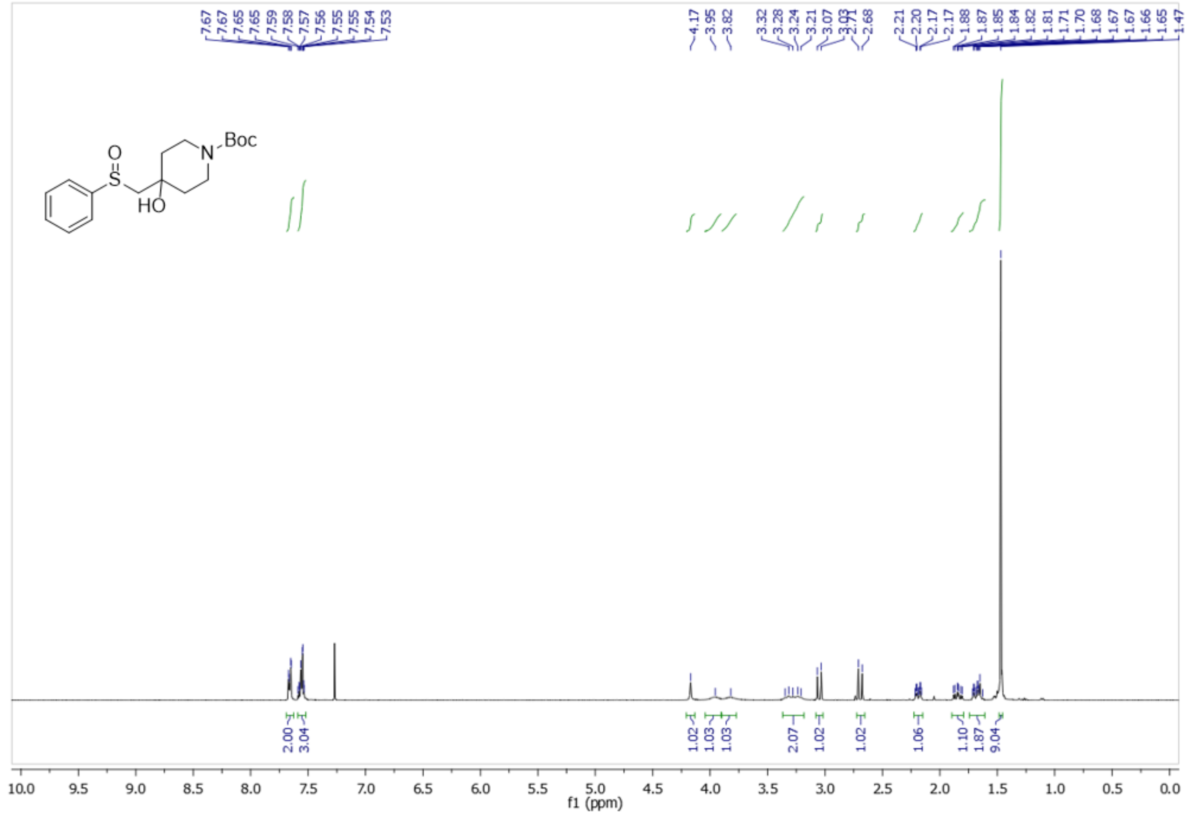

^{13}C NMR (101 MHz, $CDCl_3$):

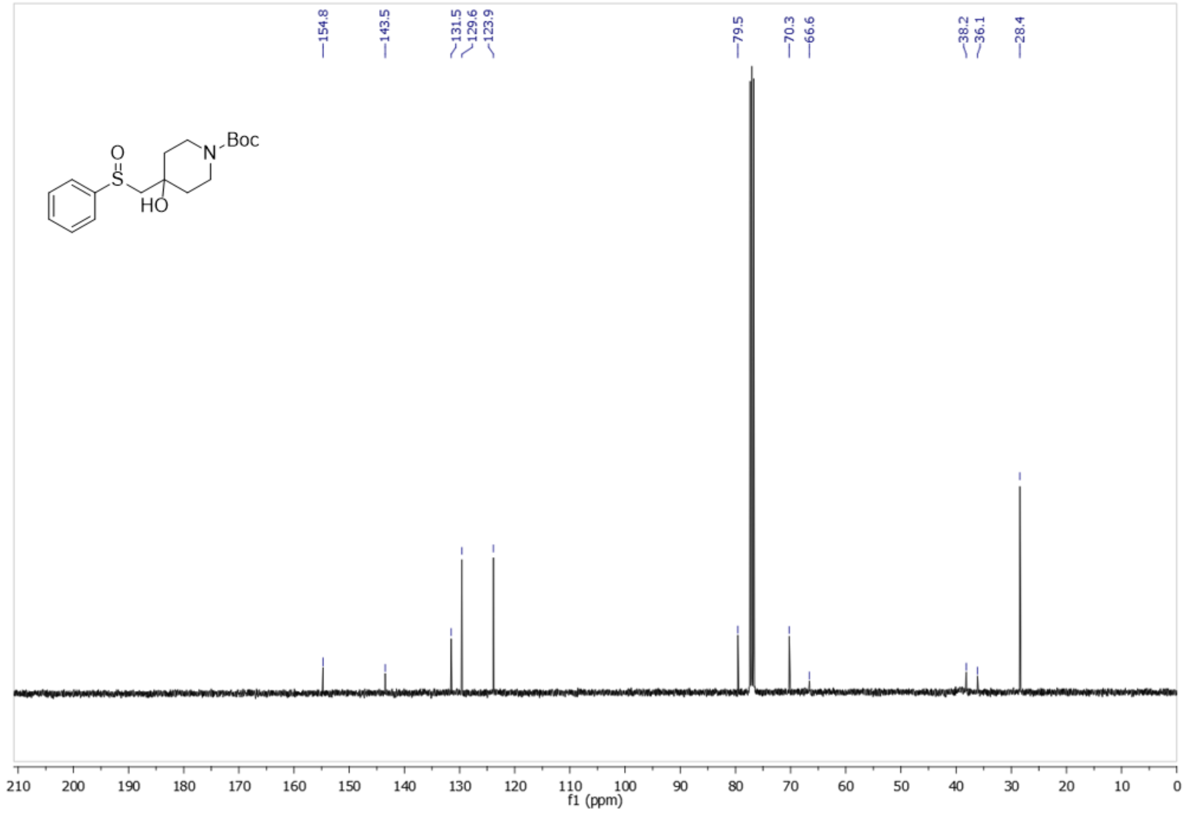

13.30 *tert*-Butyl 3-hydroxy-4-methylenepiperidine-1-carboxylate

^{1}H NMR (400 MHz, $CDCl_3$):

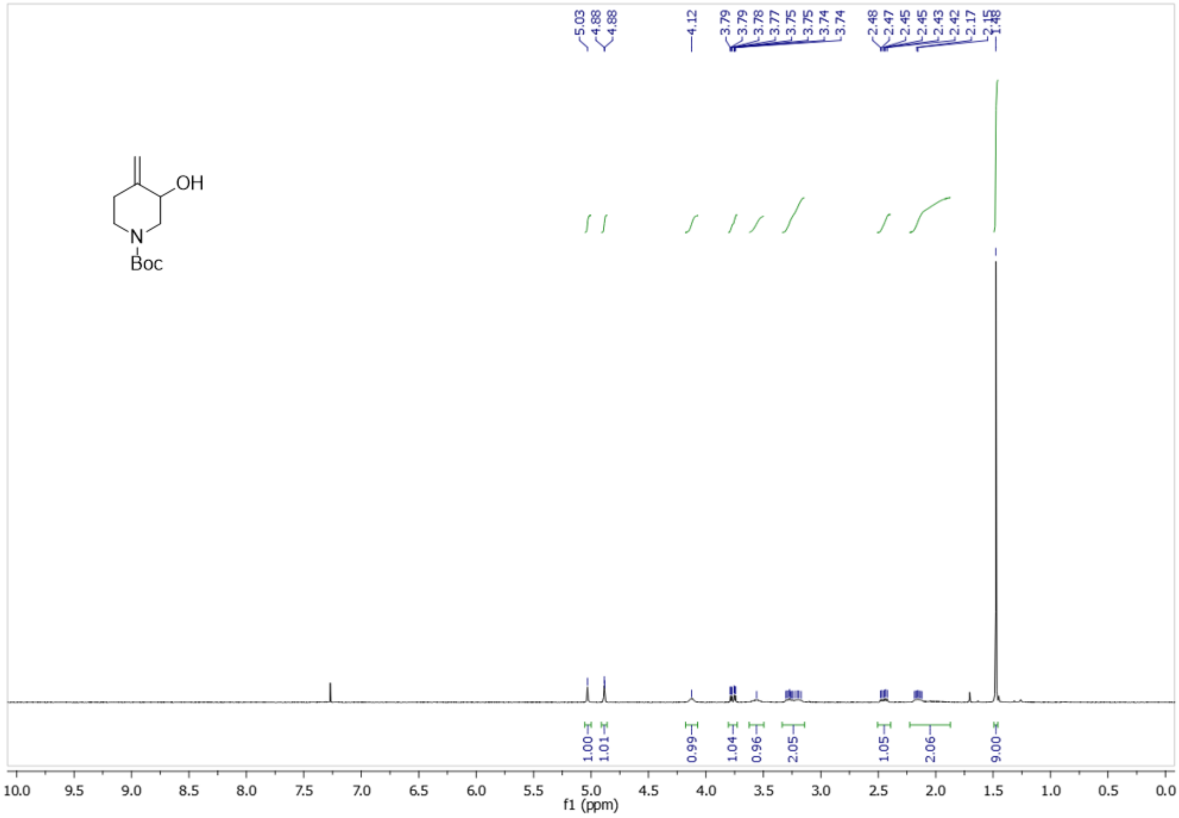

^{13}C NMR (101 MHz, $CDCl_3$):

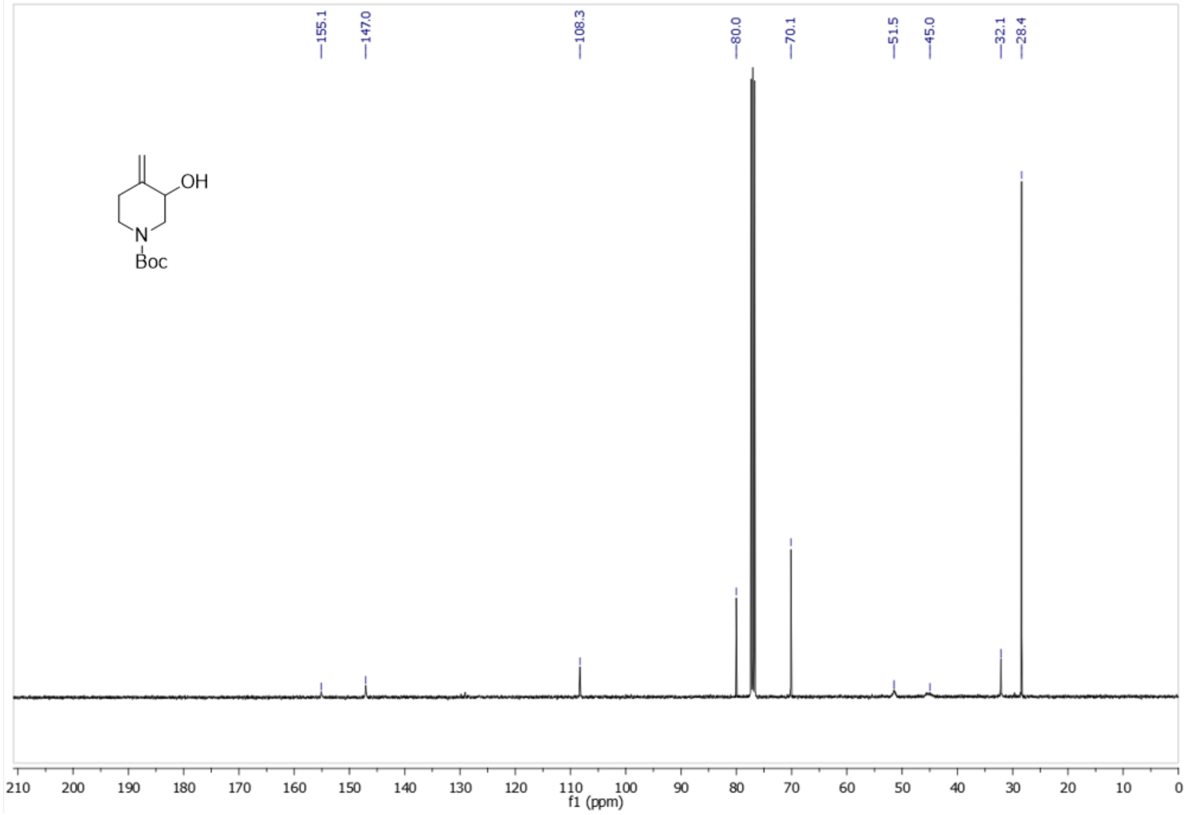

13.31 *tert*-Butyl 4-(chloromethyl)-3,6-dihydropyridine-1(2*H*)-carboxylate

^{1}H NMR (400 MHz, $CDCl_3$):

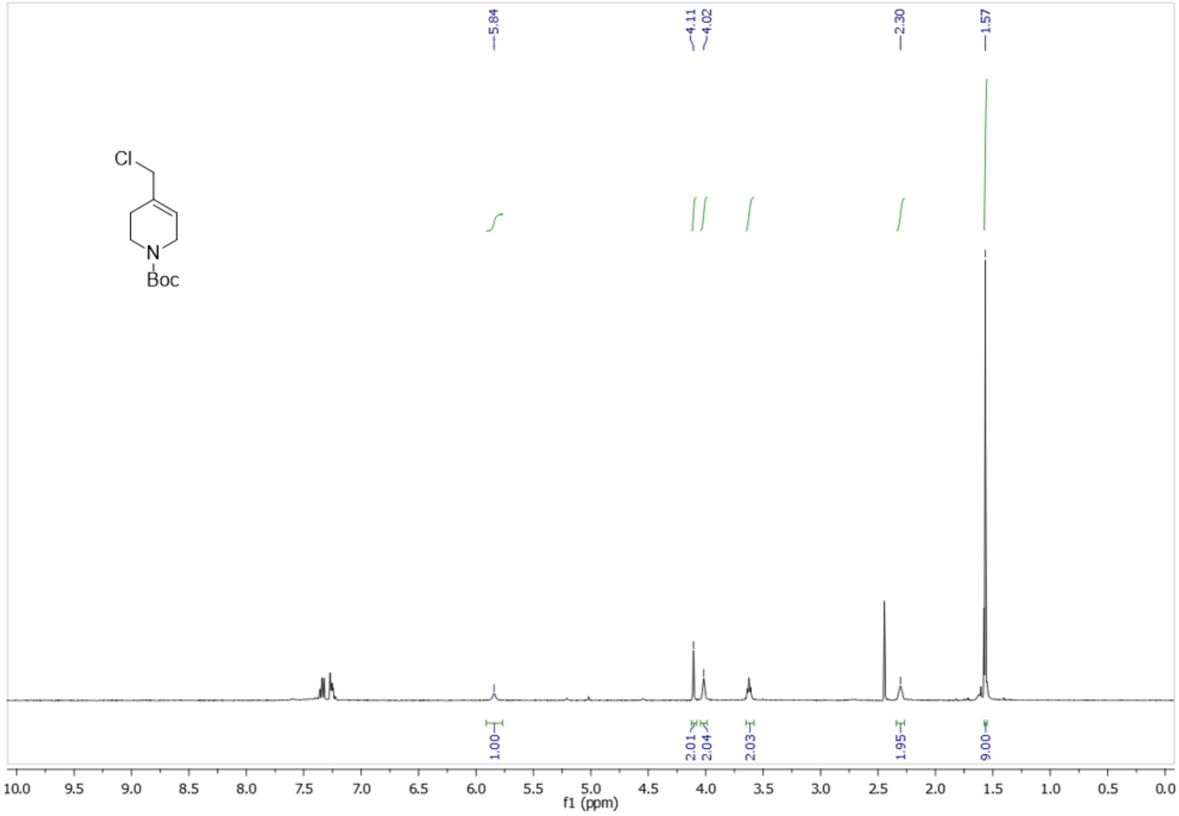

^{13}C NMR (101 MHz, $CDCl_3$):

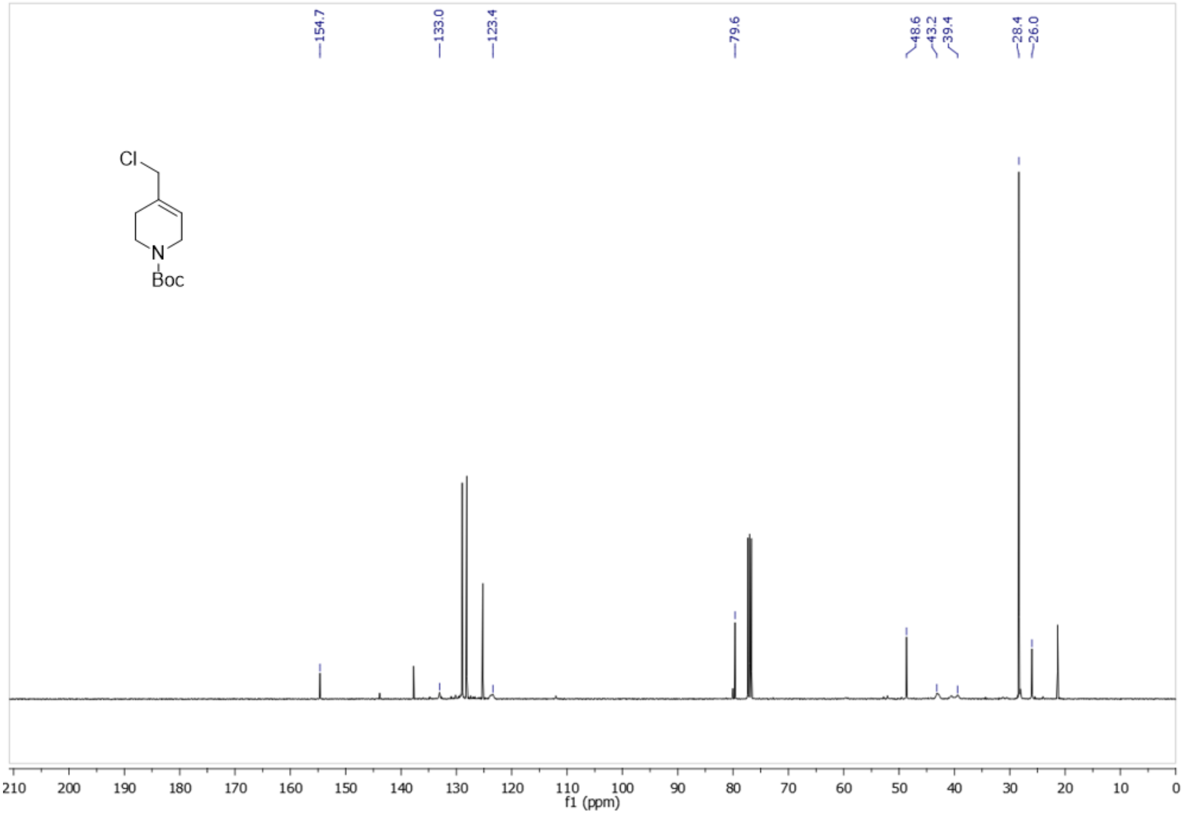

13.32 *tert*-Butyl 4-((2-bromo-4-cyanophenoxy)methyl)-3,6-dihydropyridine-1(2*H*)-carboxylate

^{1}H NMR (400 MHz, 338 K, $CDCl_3$):

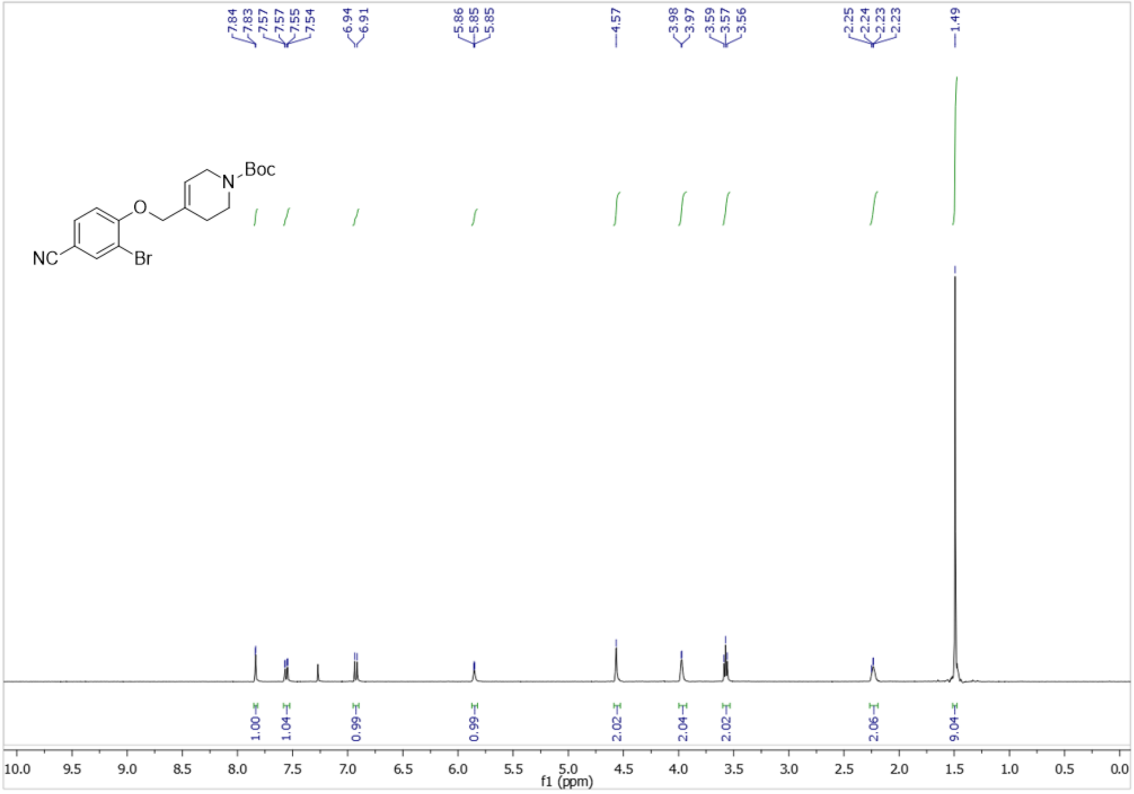

^{13}C NMR (101 MHz, 338 K, $CDCl_3$):

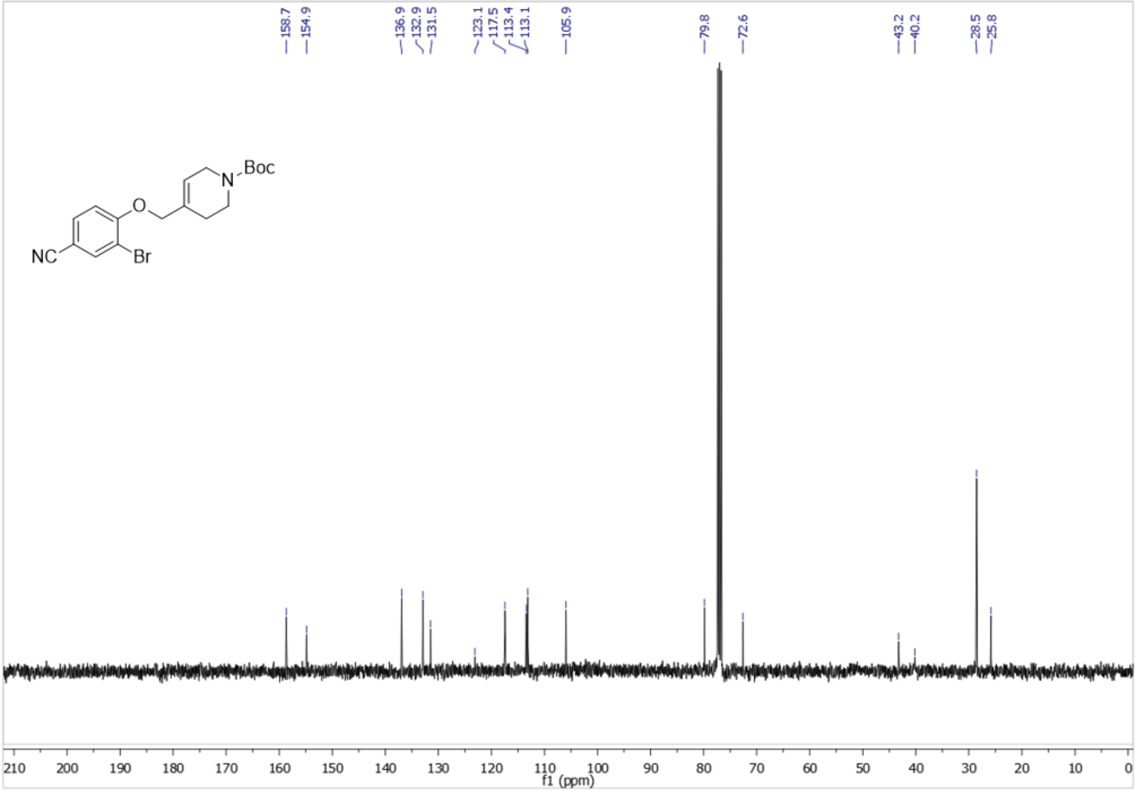

13.33 *tert*-Butyl 5-cyano-2*H*-spiro[benzofuran-3,4'-piperidine]-1'-carboxylate (15)

^{1}H NMR (400 MHz, $CDCl_3$):

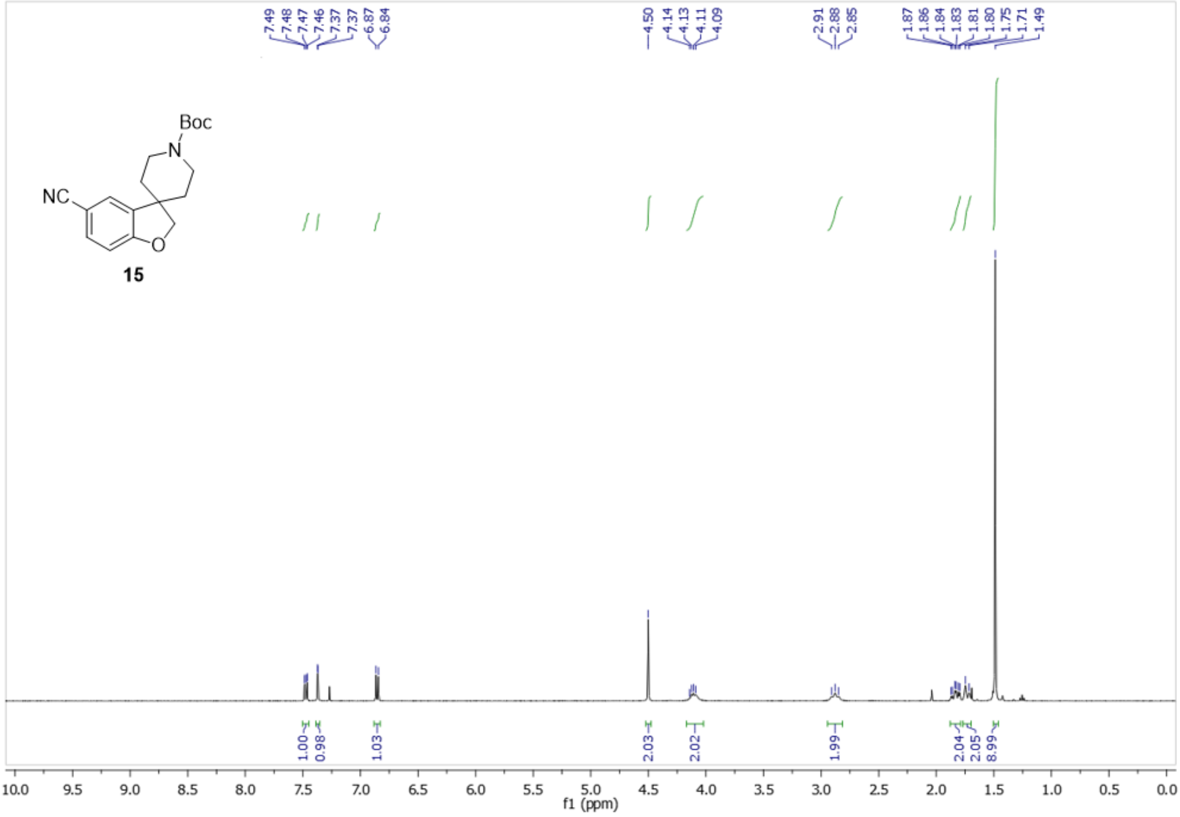

^{13}C NMR (101 MHz, $CDCl_3$):

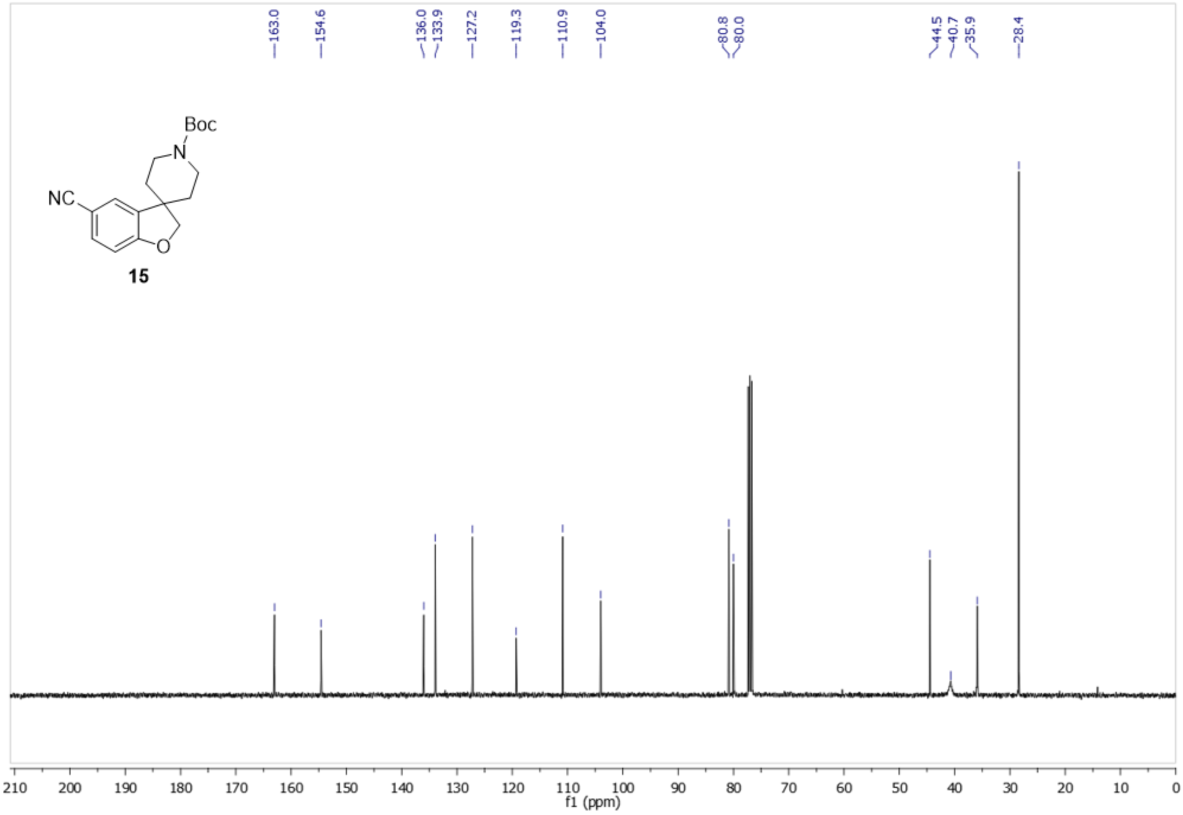

13.34 Cyclohex-1-en-1-ylmethanol

^{1}H NMR (400 MHz, $CDCl_3$):

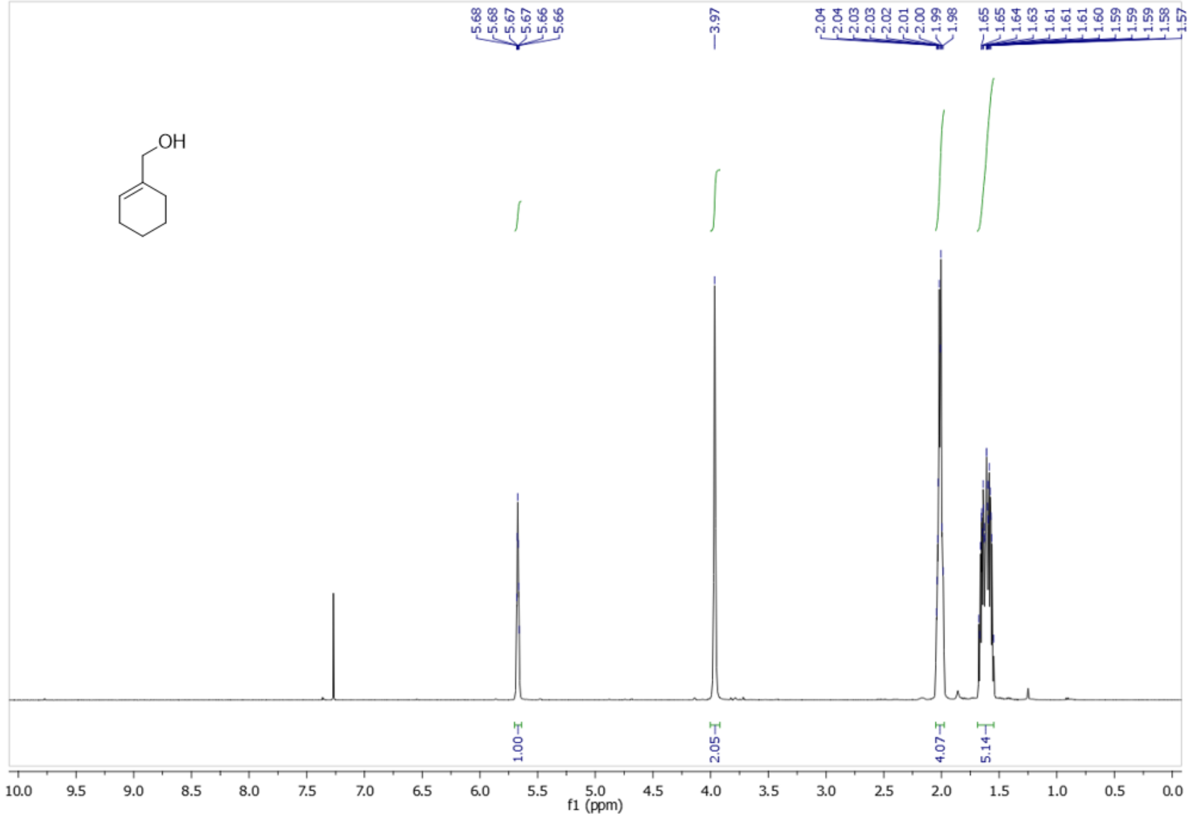

^{13}C NMR (101 MHz, $CDCl_3$):

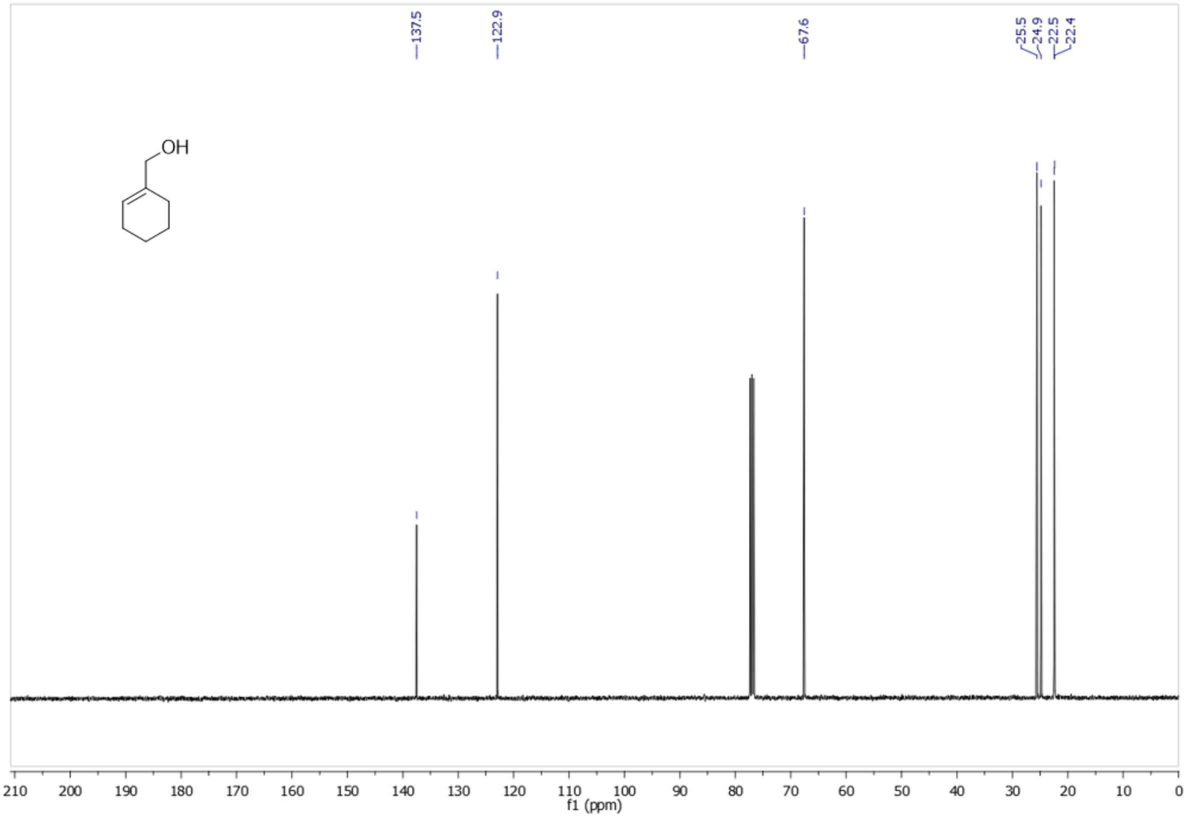

13.35 1-(Bromomethyl)cyclohex-1-ene

^{1}H NMR (400 MHz, $CDCl_3$):

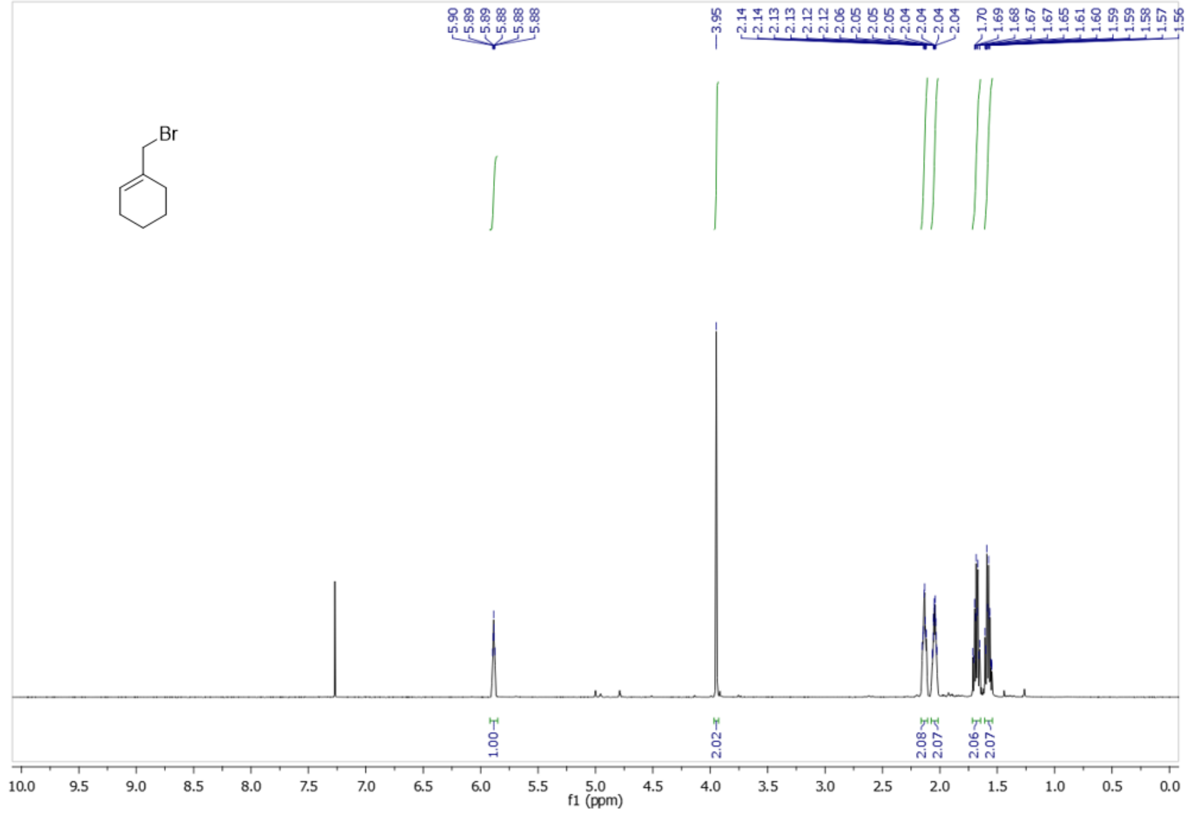

^{13}C NMR (101 MHz, $CDCl_3$):

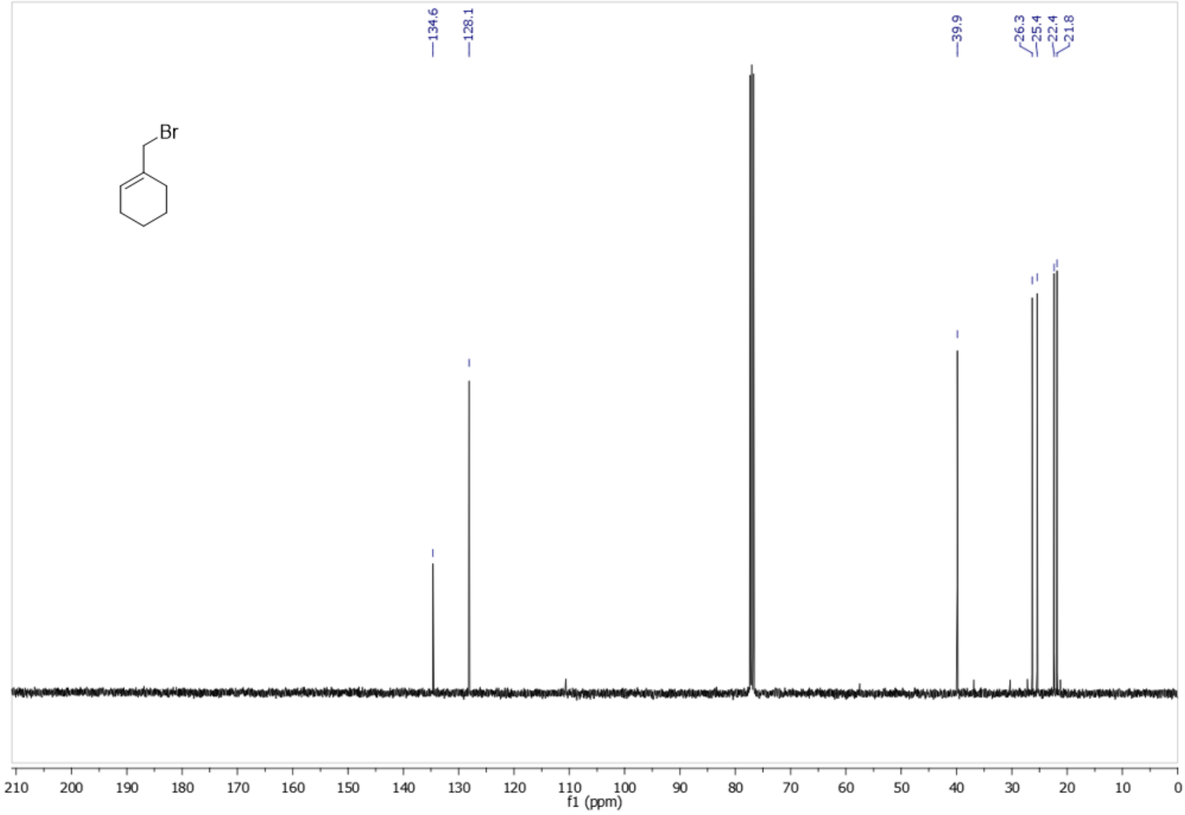

13.36 2-Bromo-1-(cyclohex-1-en-1-ylmethoxy)-4-methoxybenzene

^{1}H NMR (400 MHz, $CDCl_3$):

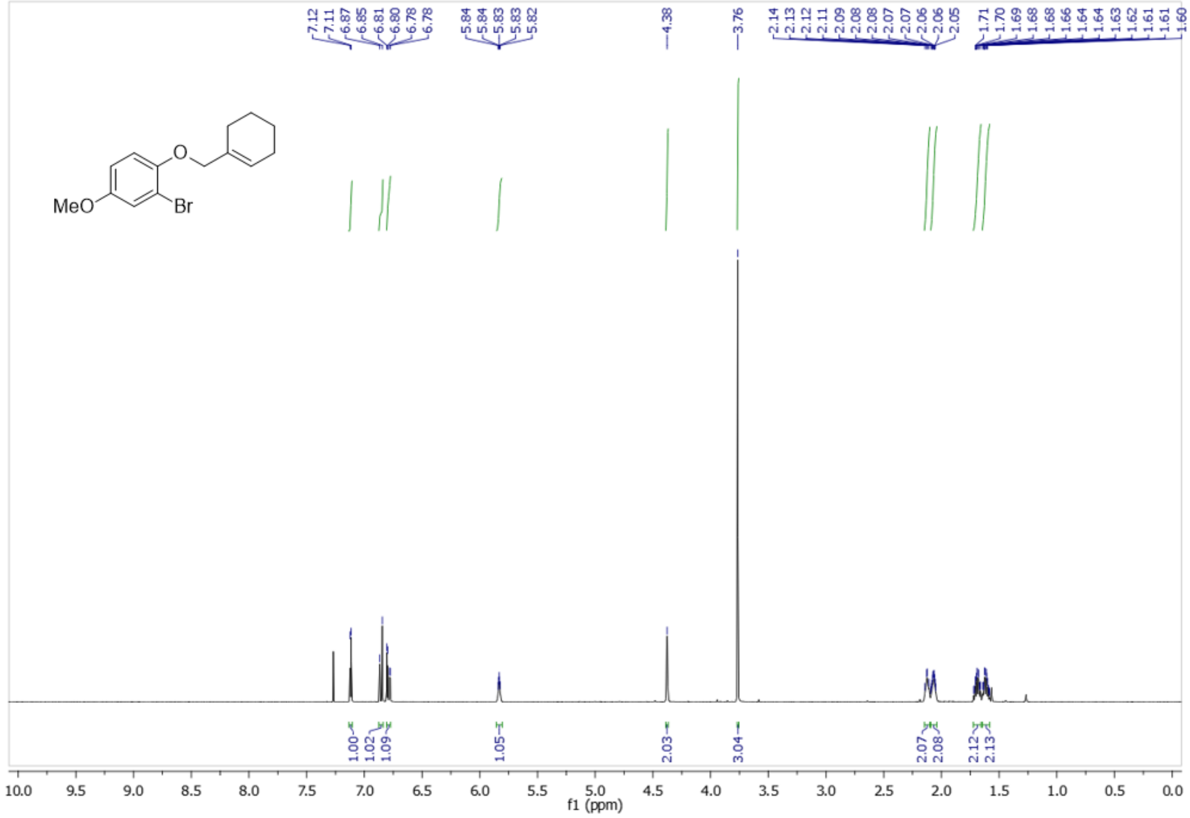

^{13}C NMR (101 MHz, $CDCl_3$):

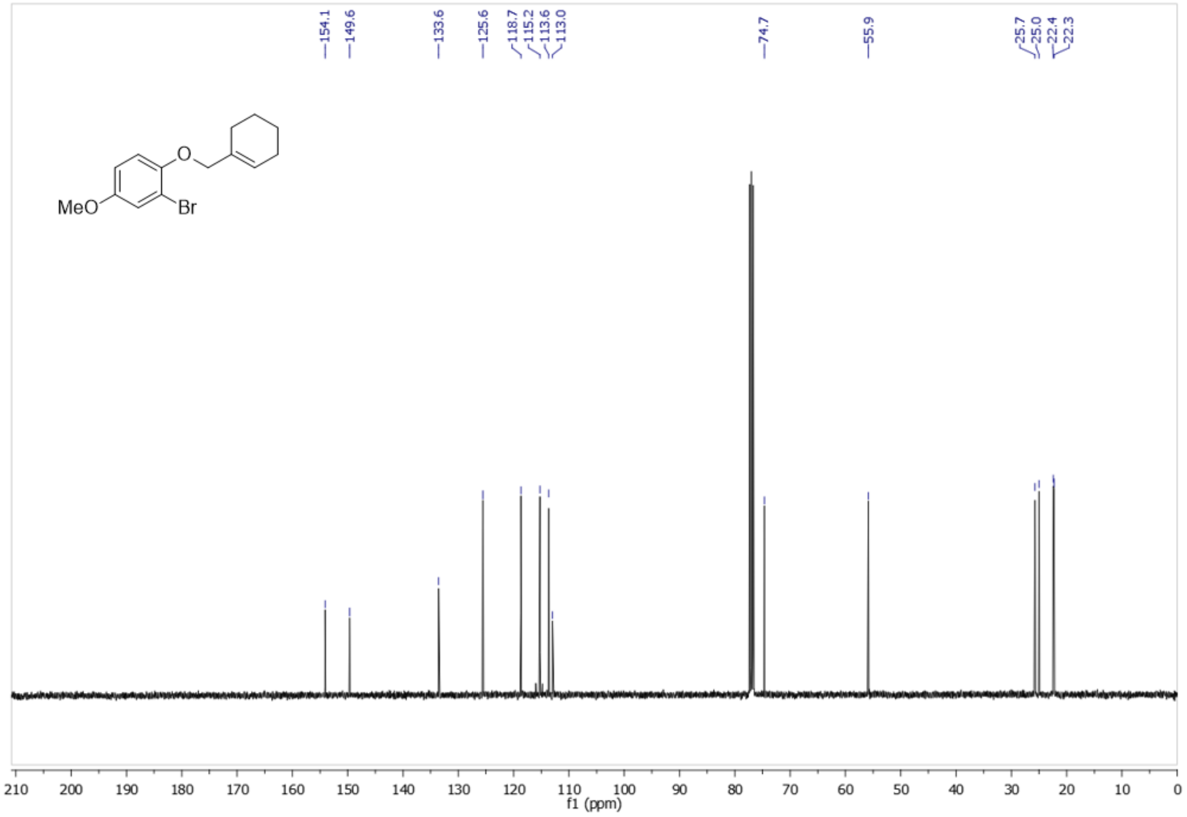

13.37 5-Methoxy-2*H*-spiro[benzofuran-3,1'-cyclohexane] (16)

^{1}H NMR (400 MHz, $CDCl_3$):

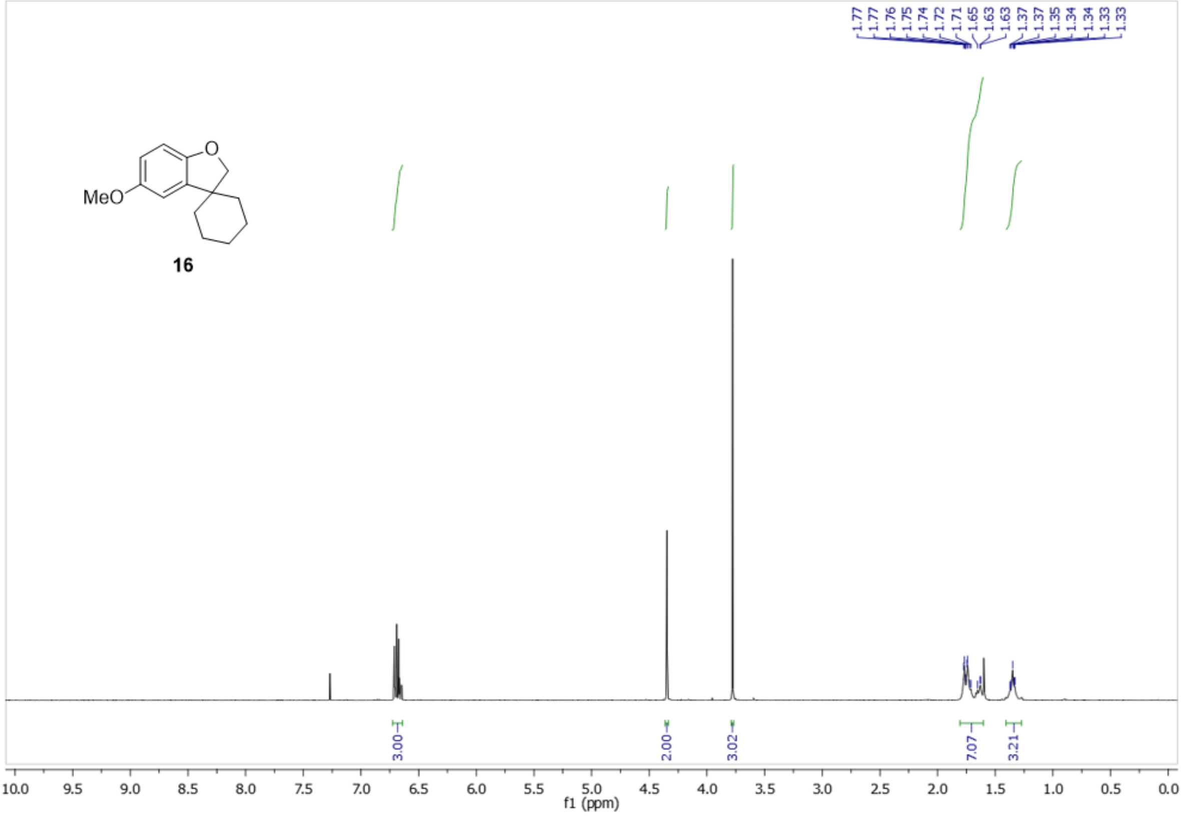

^{13}C NMR (101 MHz, $CDCl_3$):

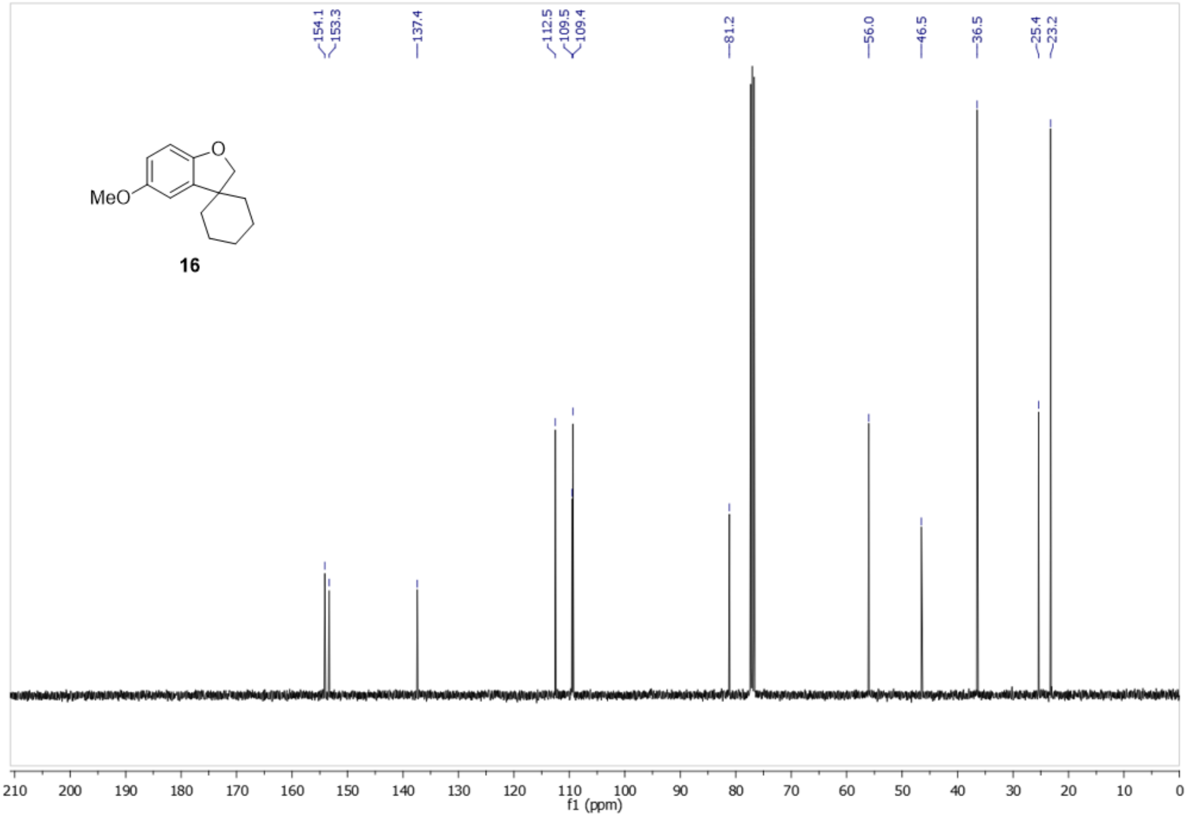

13.38 3-Bromo-4-(cyclohex-1-en-1-ylmethoxy)benzonitrile

^1H NMR (400 MHz, $CDCl_3$):

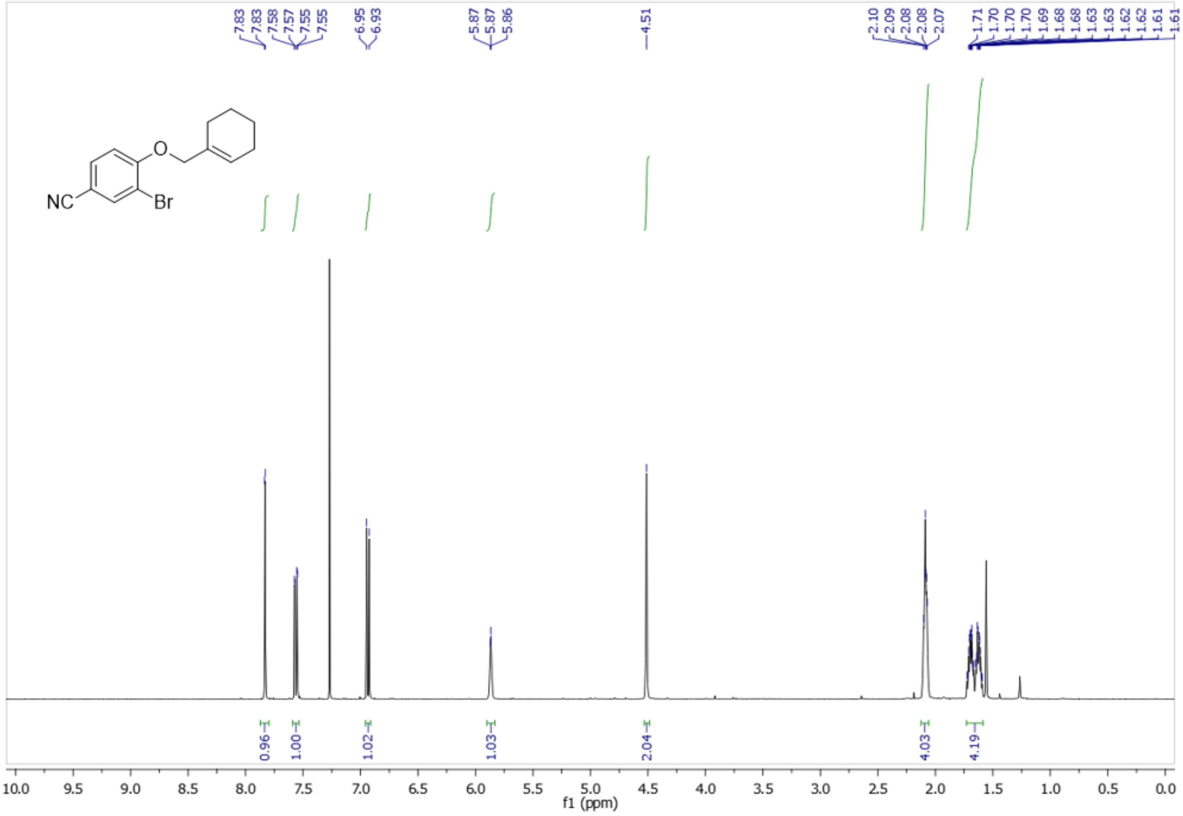

^{13}C NMR (101 MHz, $CDCl_3$):

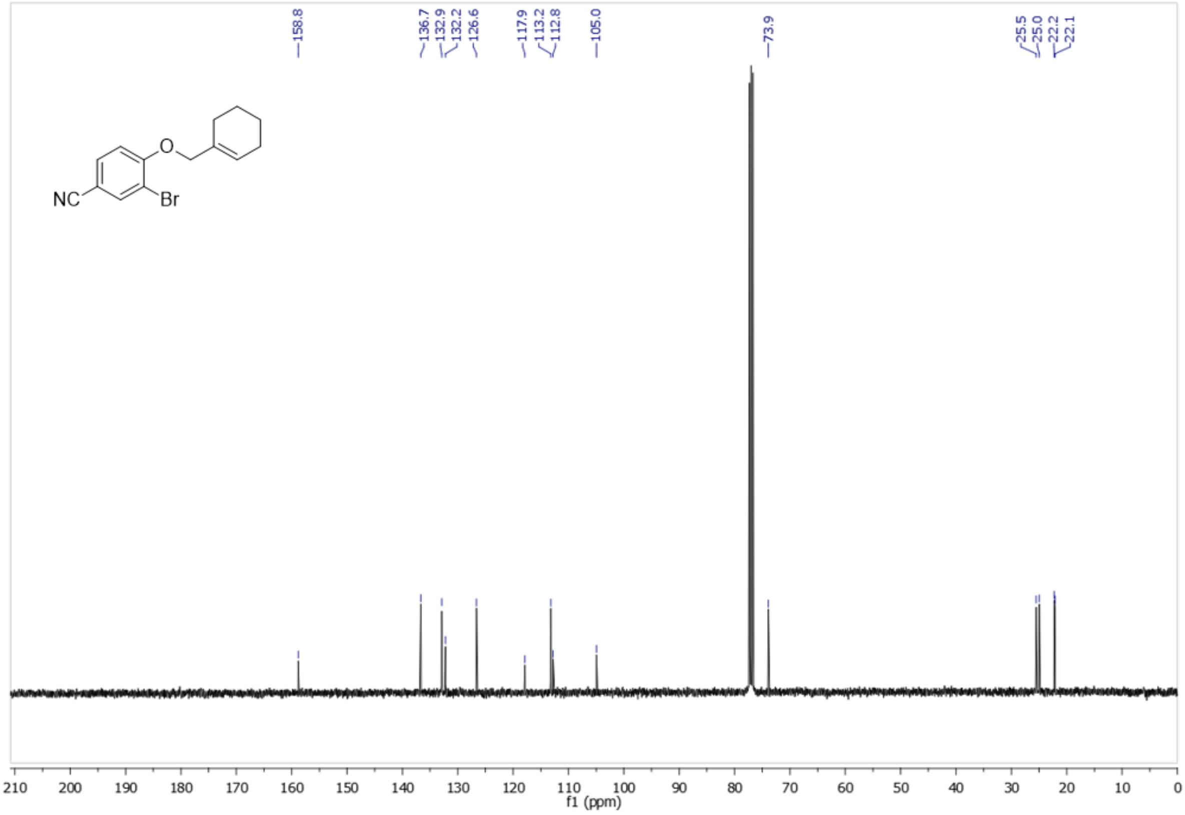

13.39 2*H*-Spiro[benzofuran-3,1'-cyclohexane]-5-carbonitrile (17)

^1H NMR (400 MHz, $CDCl_3$):

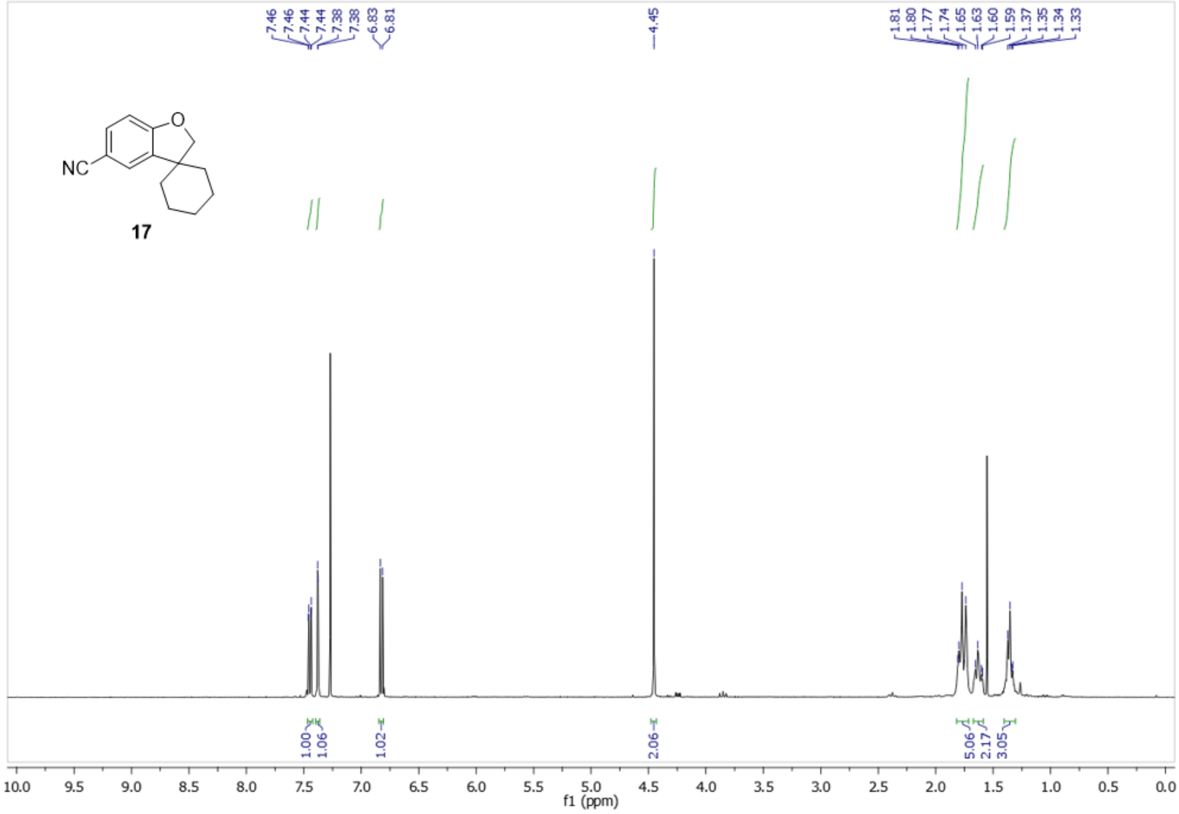

^{13}C NMR (101 MHz, $CDCl_3$):

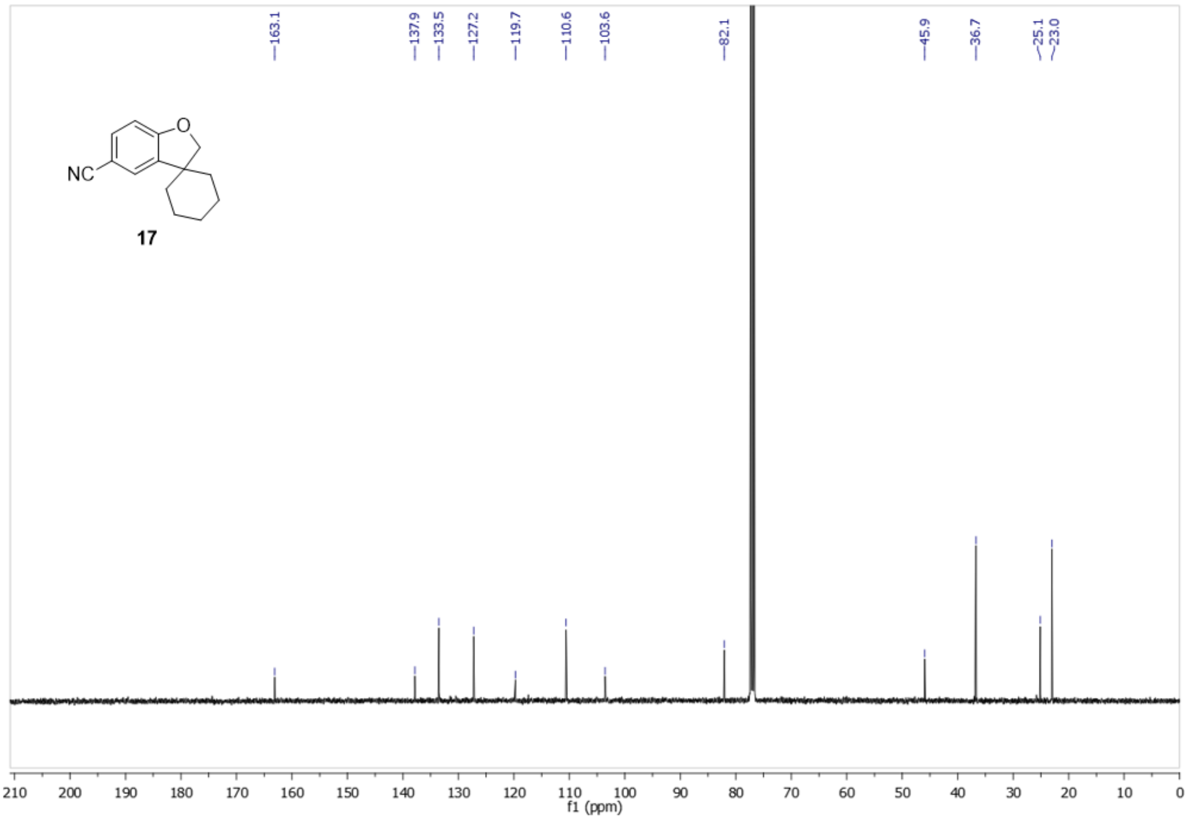

13.40 1-(But-3-en-1-yloxy)-2-iodobenzene

^{1}H NMR (400 MHz, $CDCl_3$):

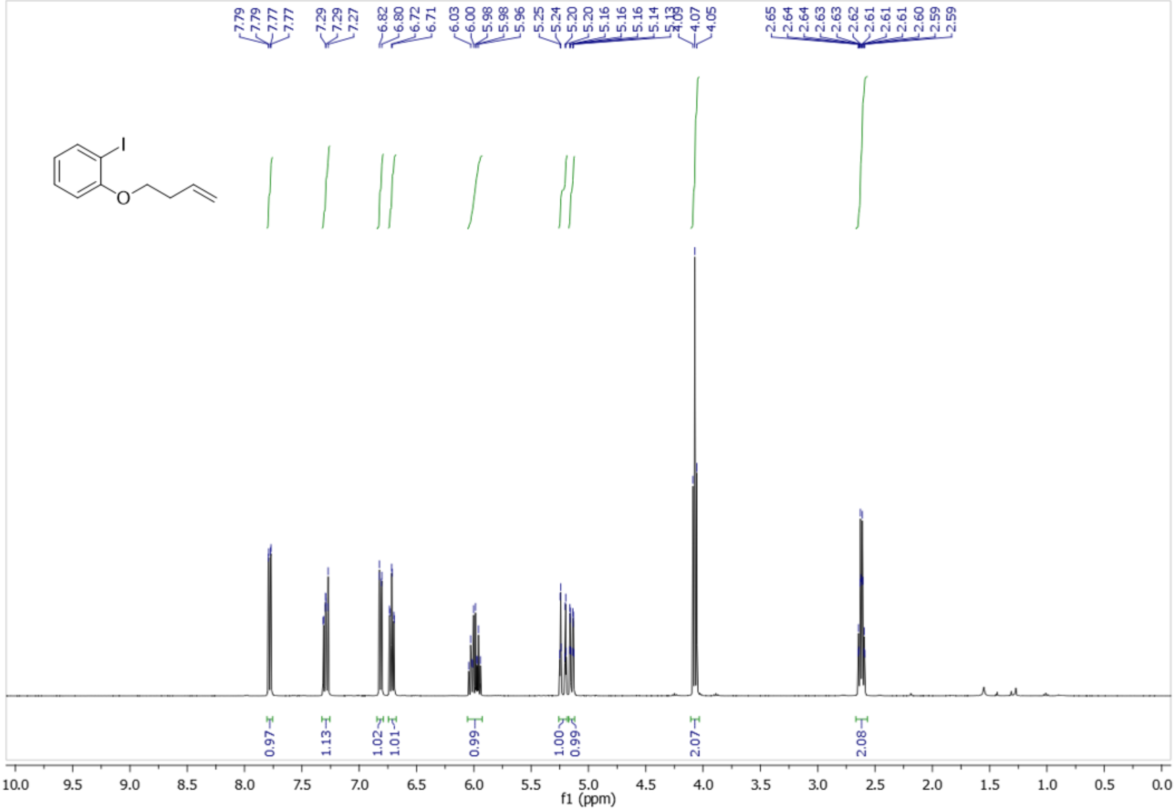

^{13}C NMR (101 MHz, $CDCl_3$):

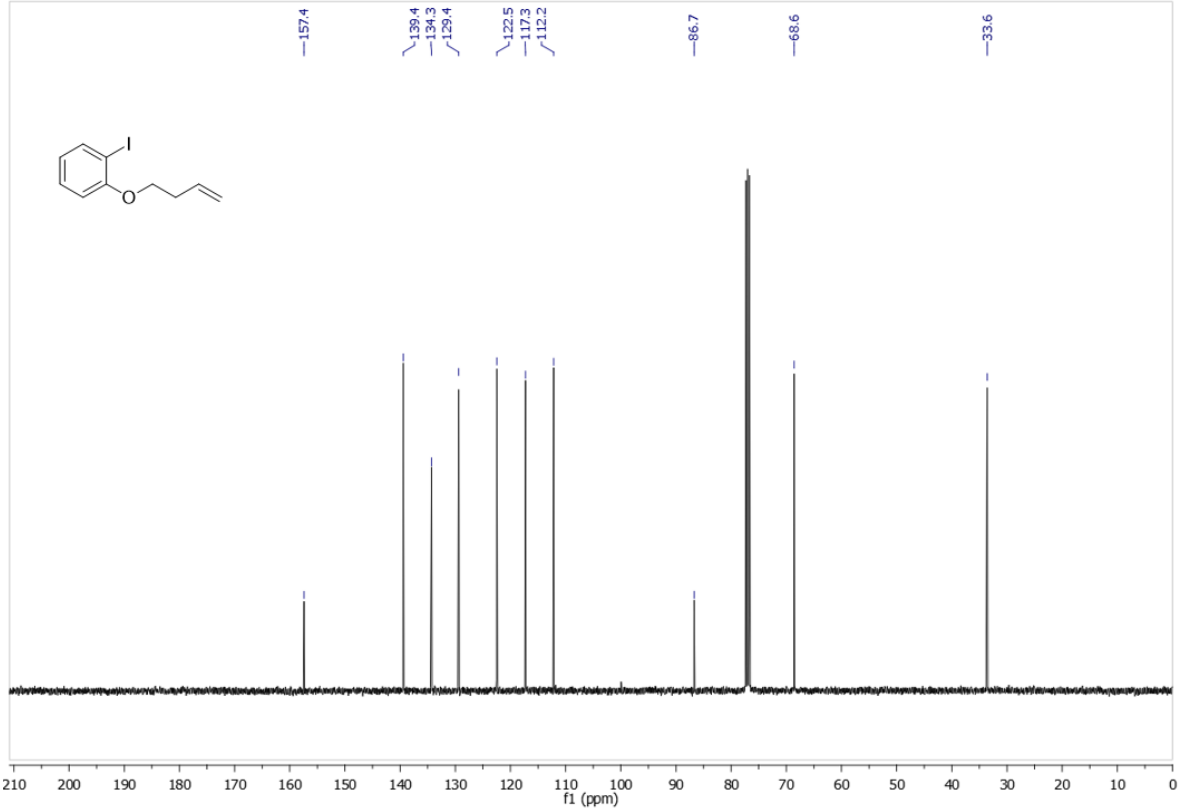

13.41 1-Iodo-2-(oct-1-en-3-yloxy)benzene

^{1}H NMR (400 MHz, $CDCl_3$):

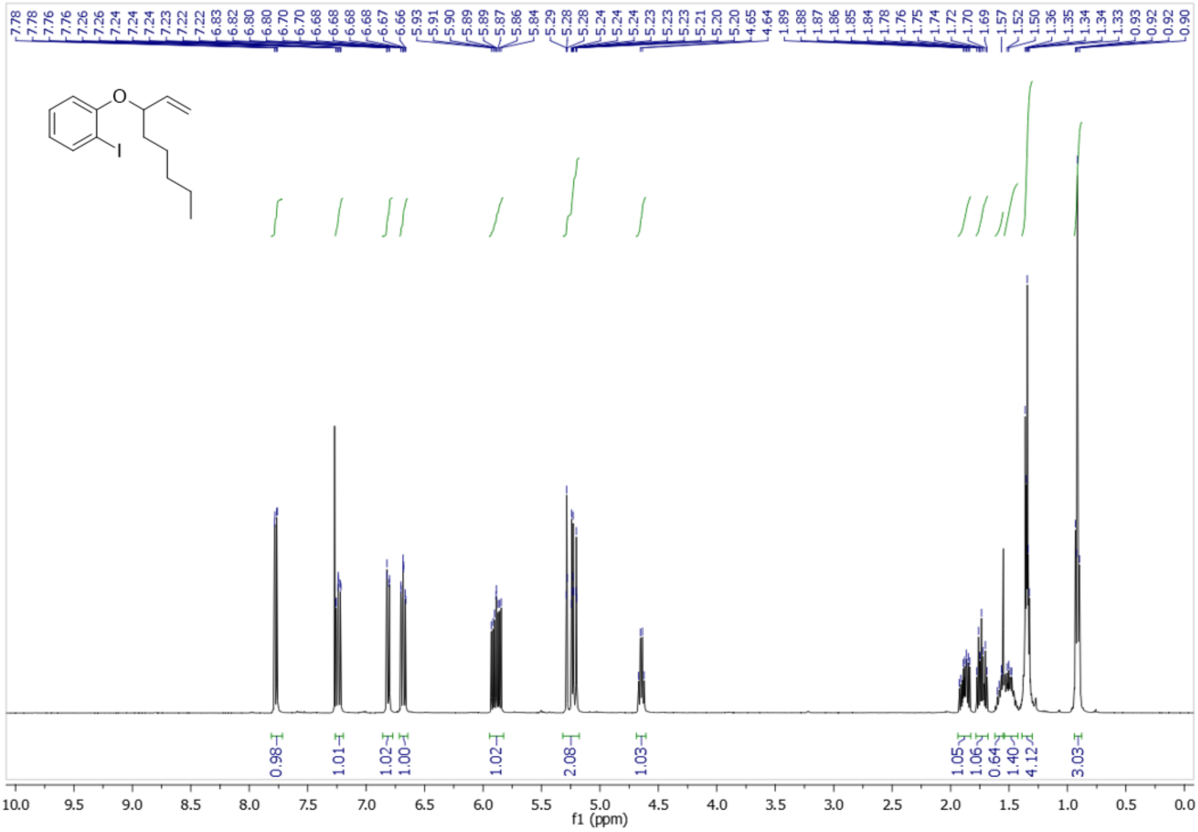

^{13}C NMR (101 MHz, $CDCl_3$):

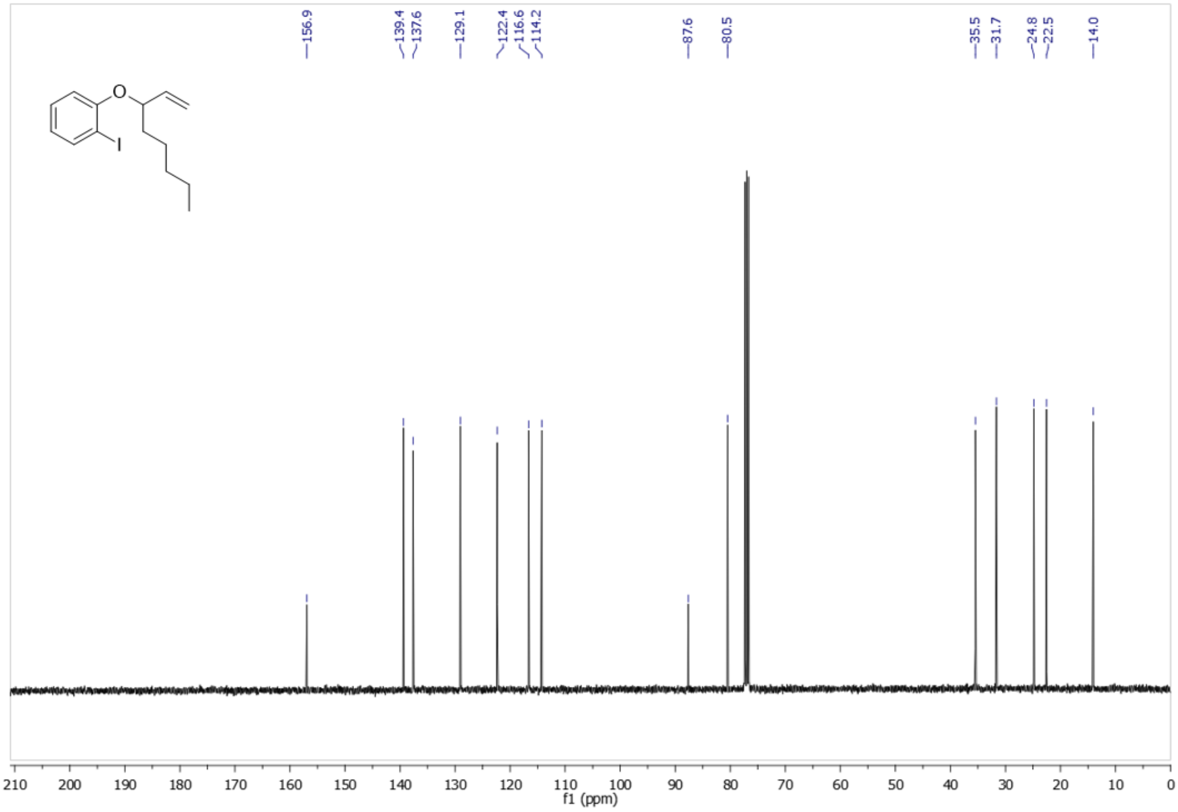

13.42 3-Methyl-2-pentyl-2,3-dihydrobenzofuran (18, dr ~ 82:12)

^{1}H NMR (400 MHz, $CDCl_3$):

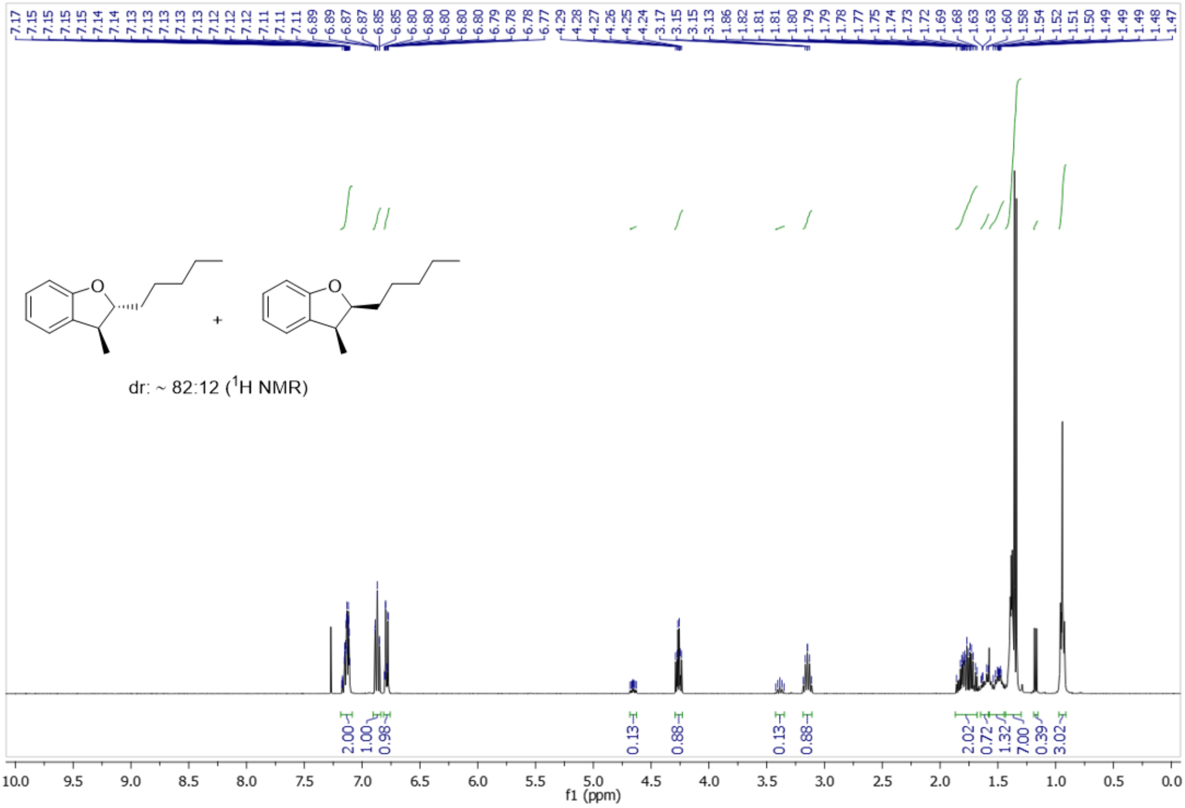

^{13}C NMR (101 MHz, $CDCl_3$):

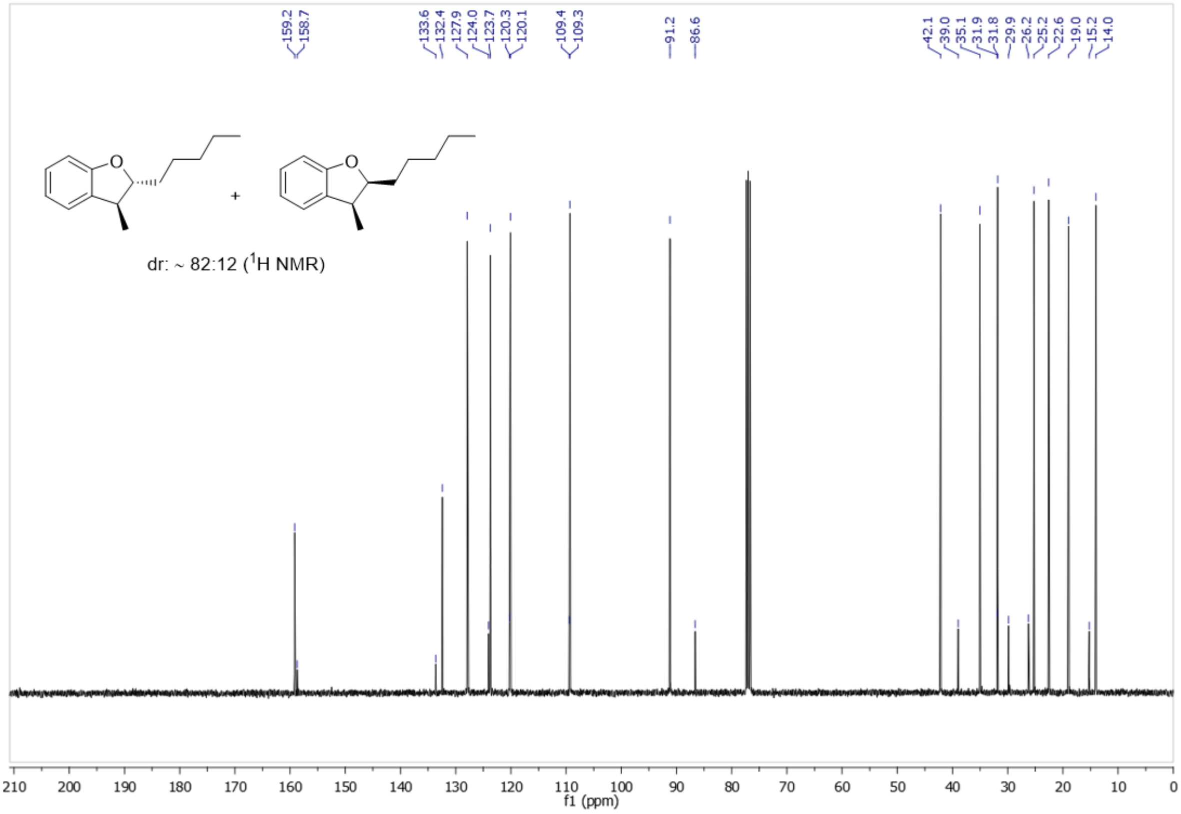

13.43 (*E*)-1-(Dec-2-en-1-yloxy)-2-iodobenzene

^{1}H NMR (400 MHz, CDCl$_3$):

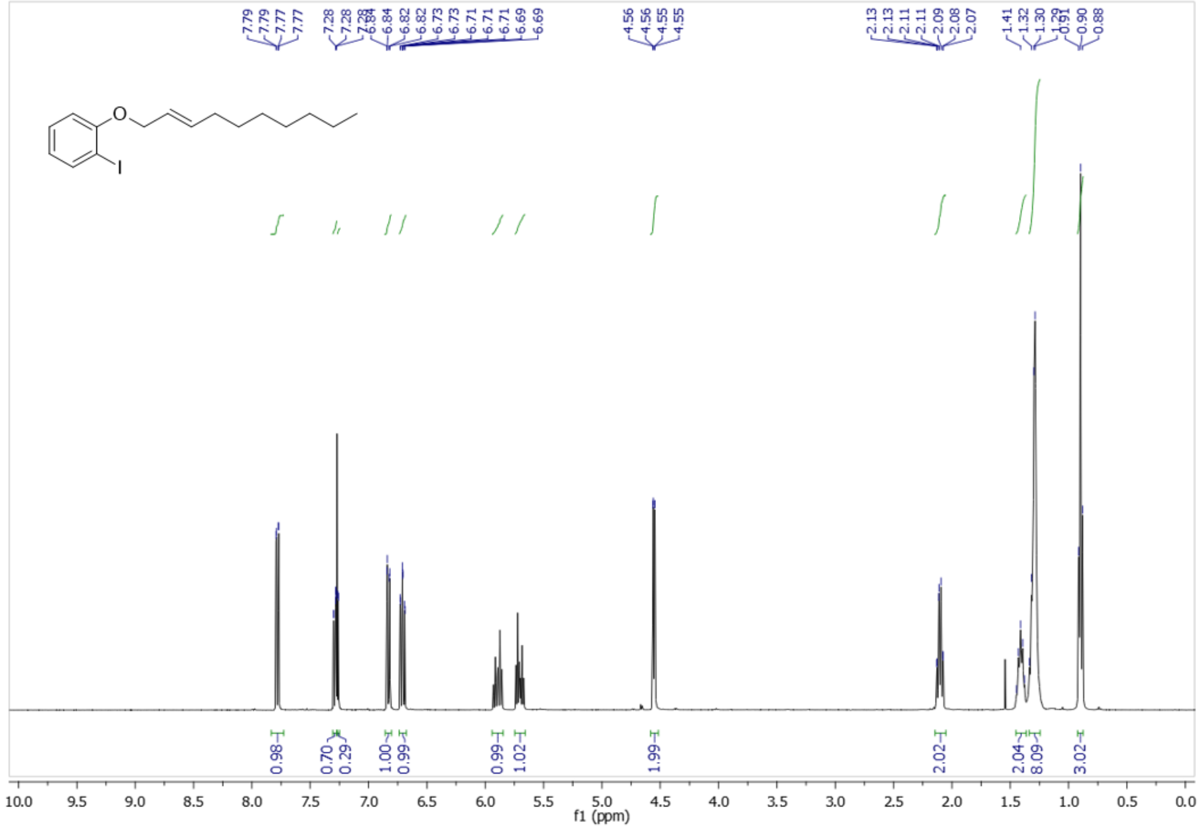

^{13}C NMR (101 MHz, CDCl$_3$):

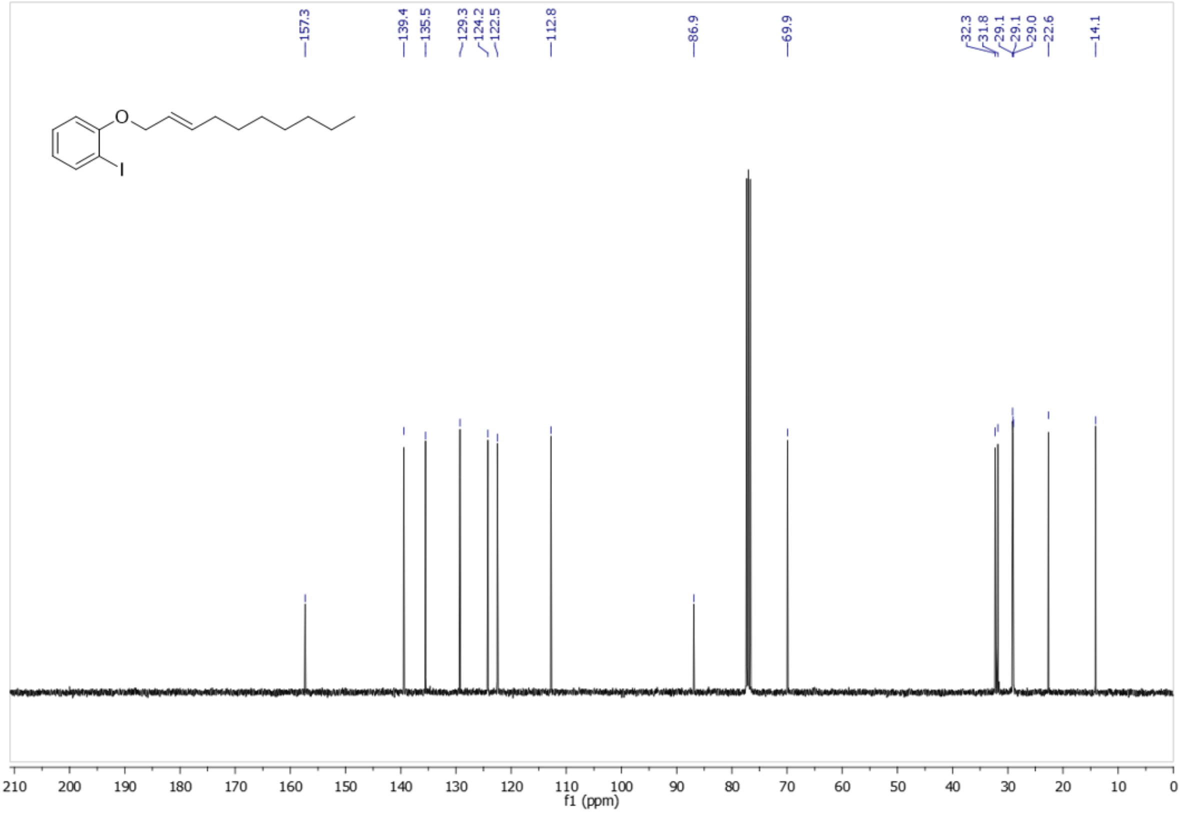

13.44 (*E*)-1-Chloro-2-(dec-2-en-1-yloxy)benzene

^{1}H NMR (400 MHz, $CDCl_3$):

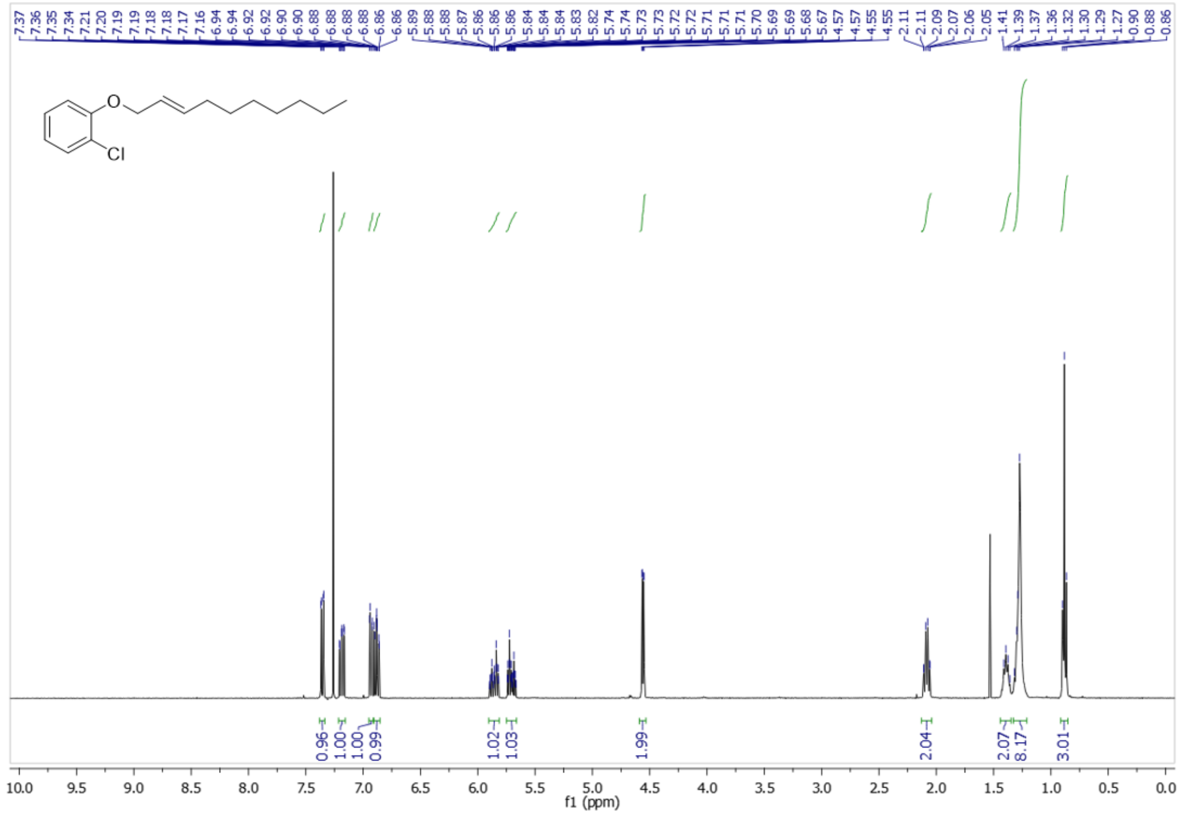

^{13}C NMR (101 MHz, $CDCl_3$):

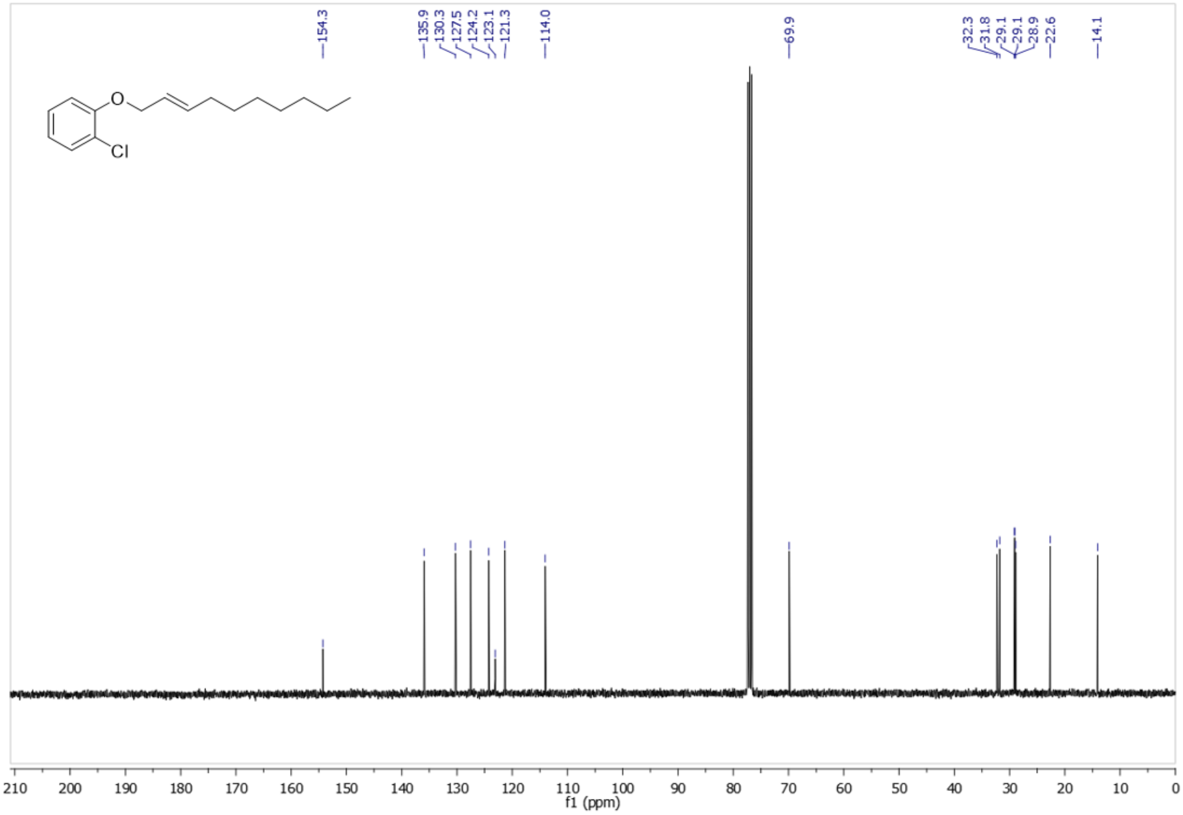

13.45 3-Octyl-2,3-dihydrobenzofuran (19)

^{1}H NMR (400 MHz, $CDCl_3$):

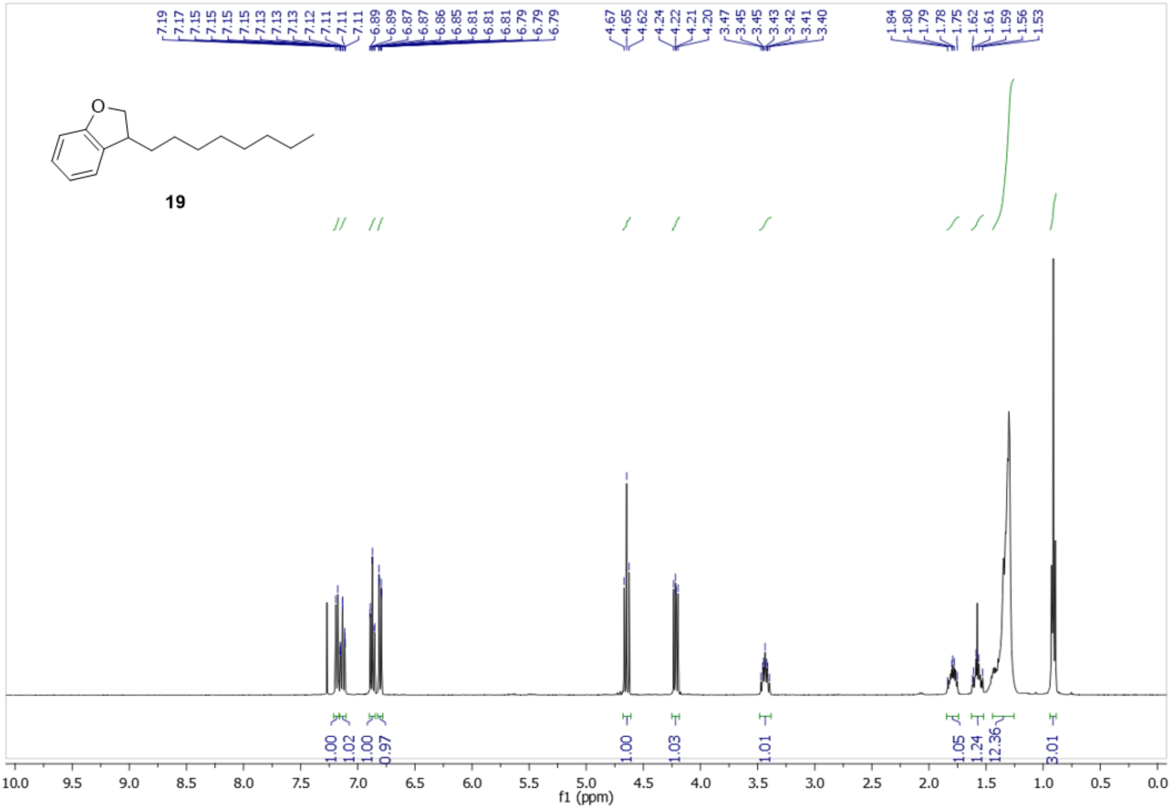

^{13}C NMR (101 MHz, $CDCl_3$):

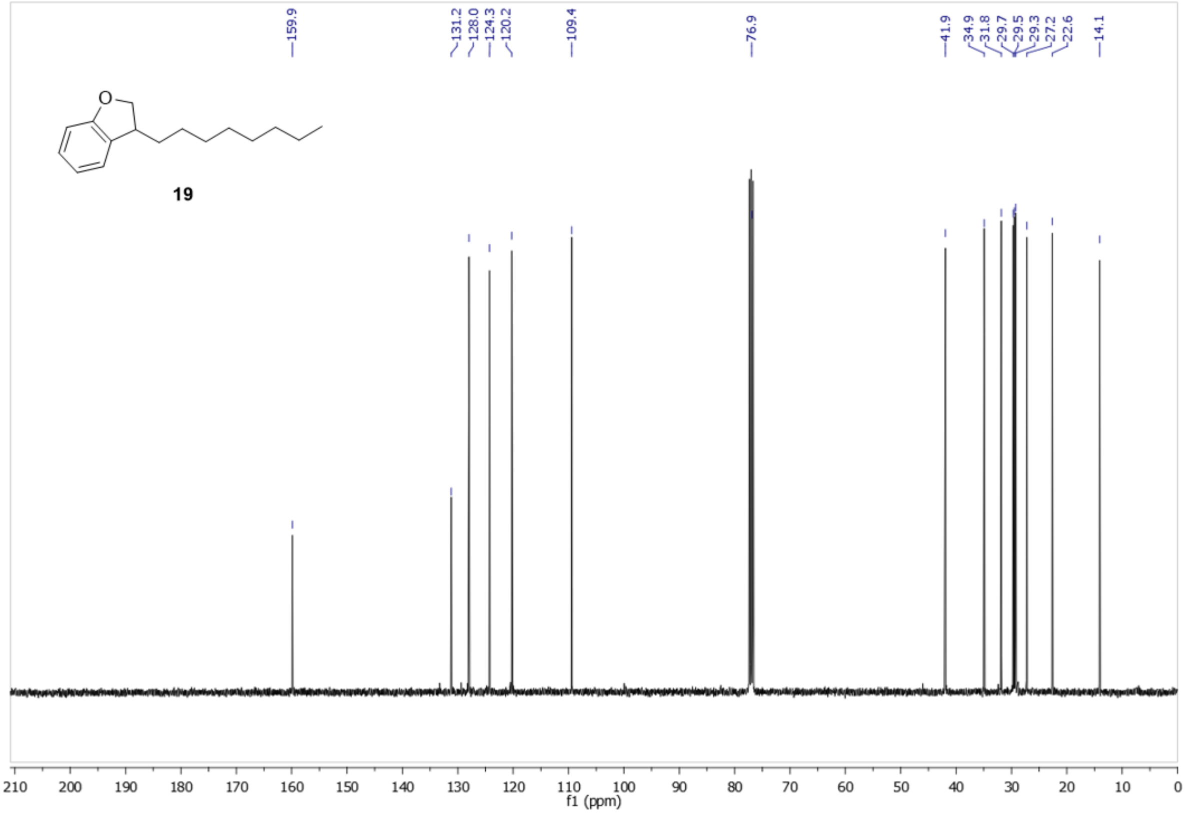

13.46 4-Methylchromane (20)

^{1}H NMR (400 MHz, $CDCl_3$):

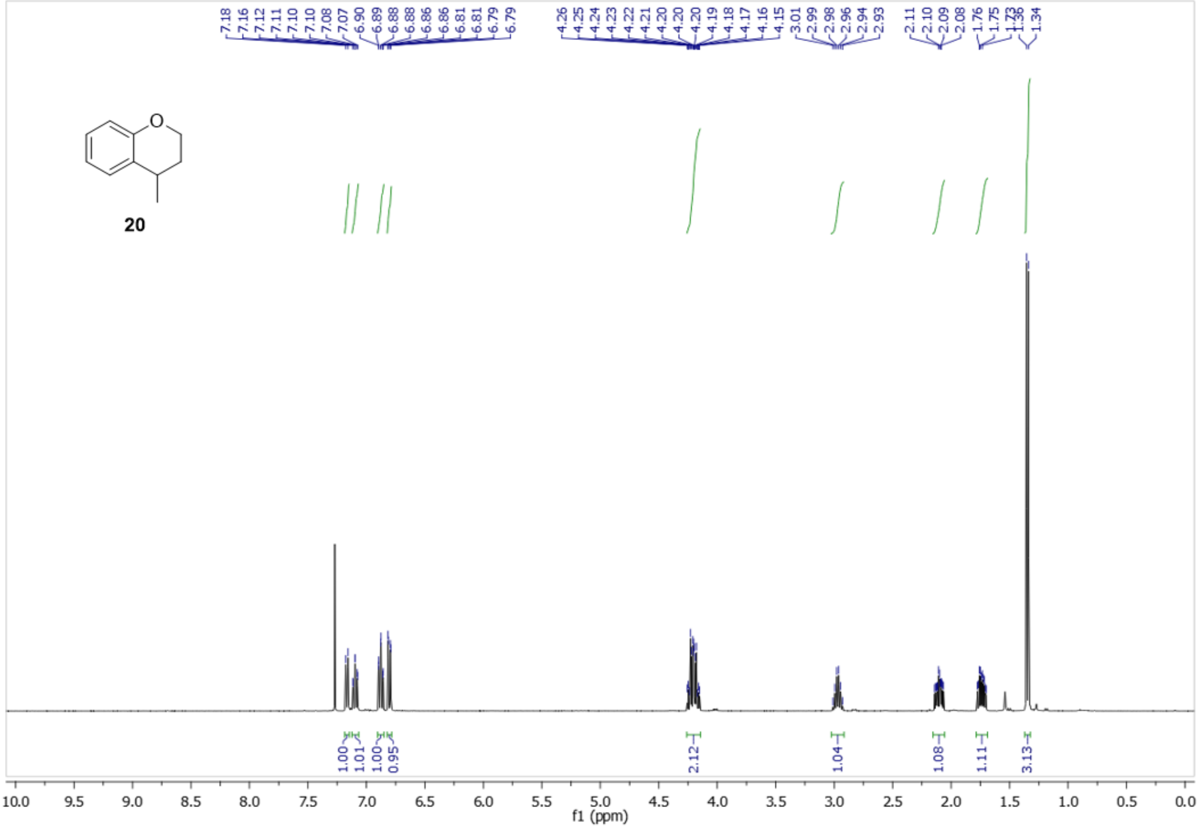

^{13}C NMR (101 MHz, $CDCl_3$):

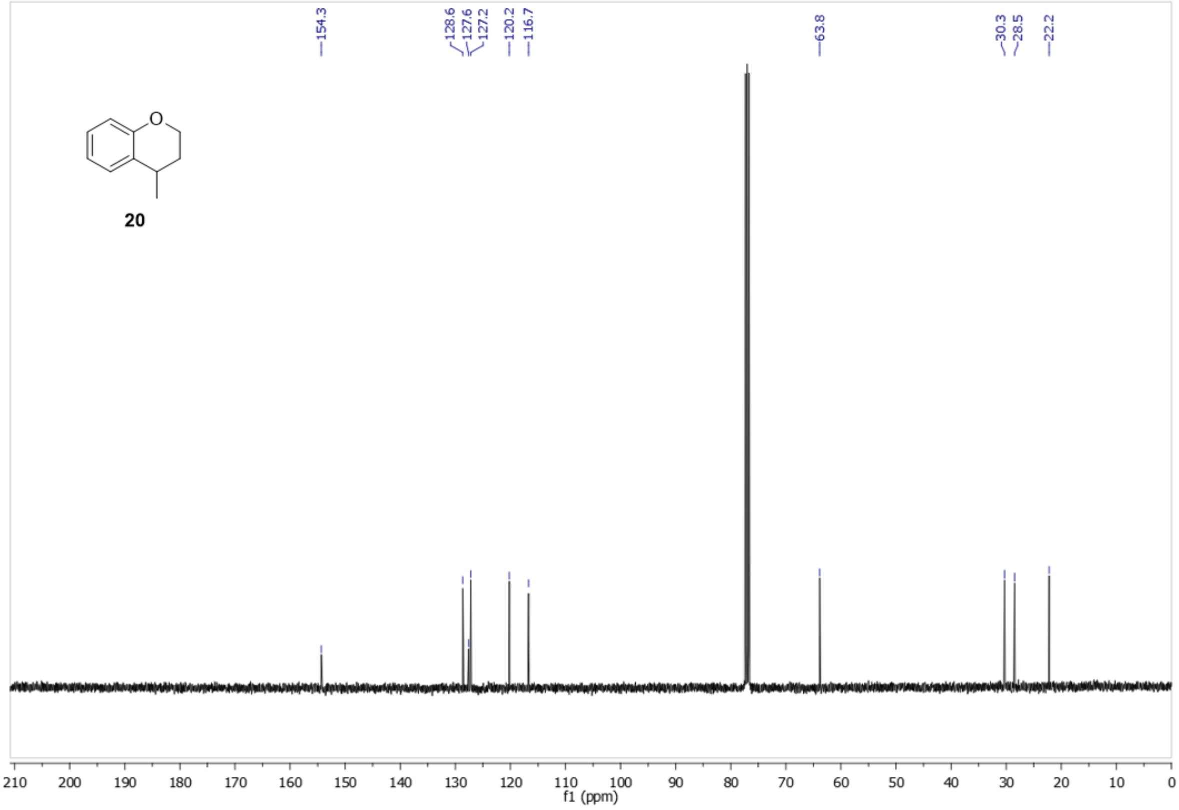

13.47 1,2-Di(chroman-4-yl)ethane

^{1}H NMR (400 MHz, $CDCl_3$):

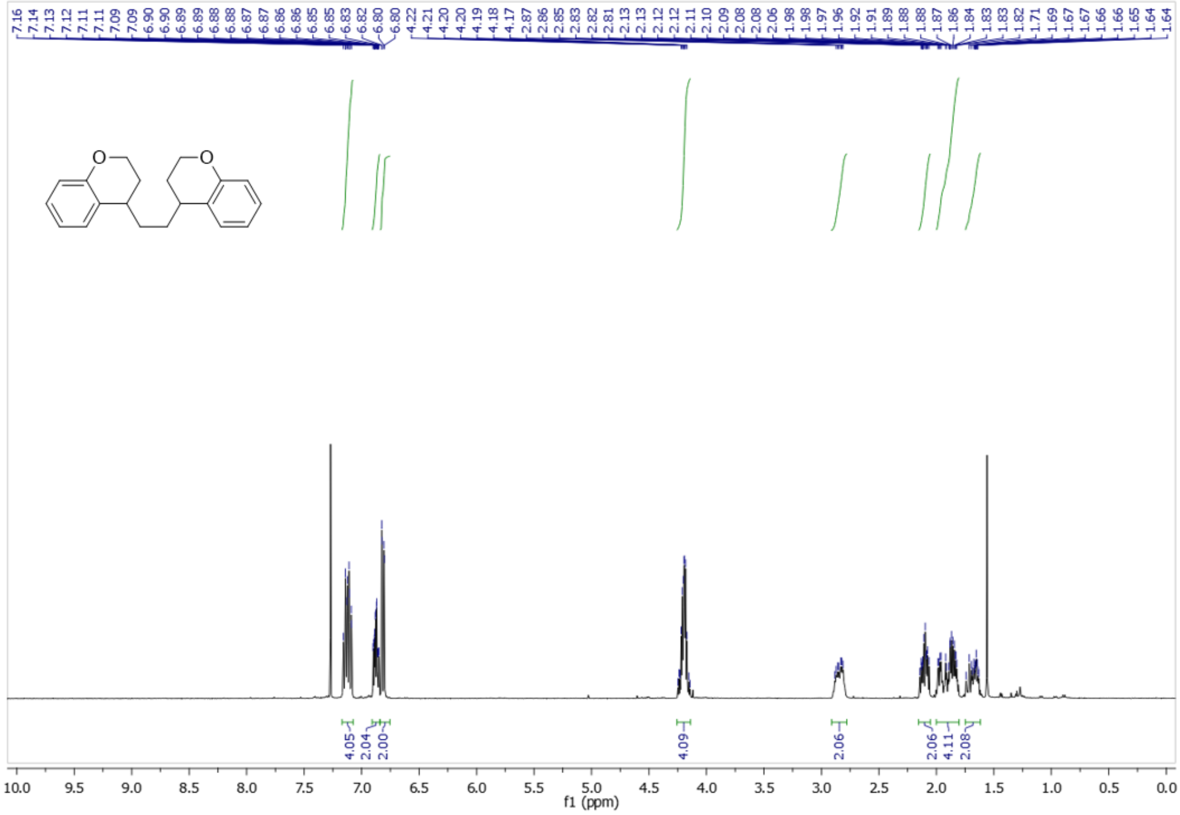

^{13}C NMR (101 MHz, $CDCl_3$):

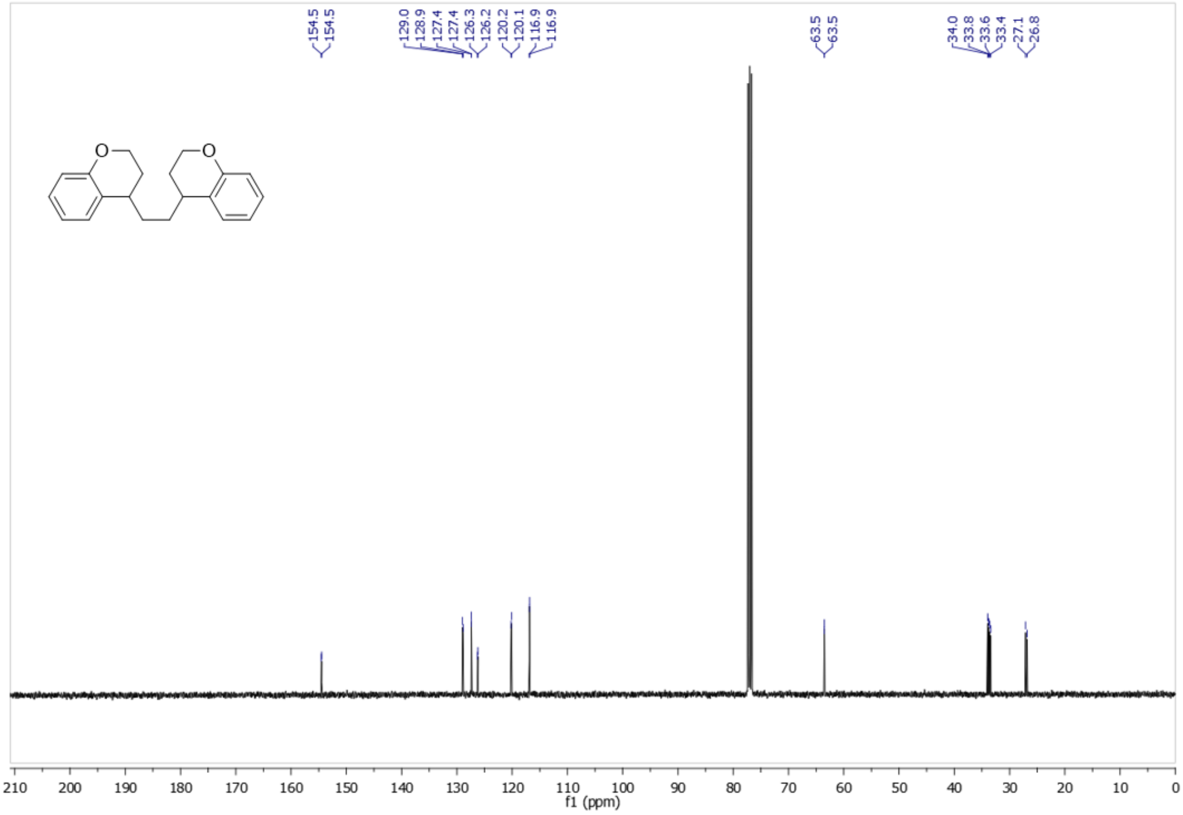

13.48 3-(But-3-en-1-yloxy)-2-iodopyridine

^{1}H NMR (400 MHz, $CDCl_3$):

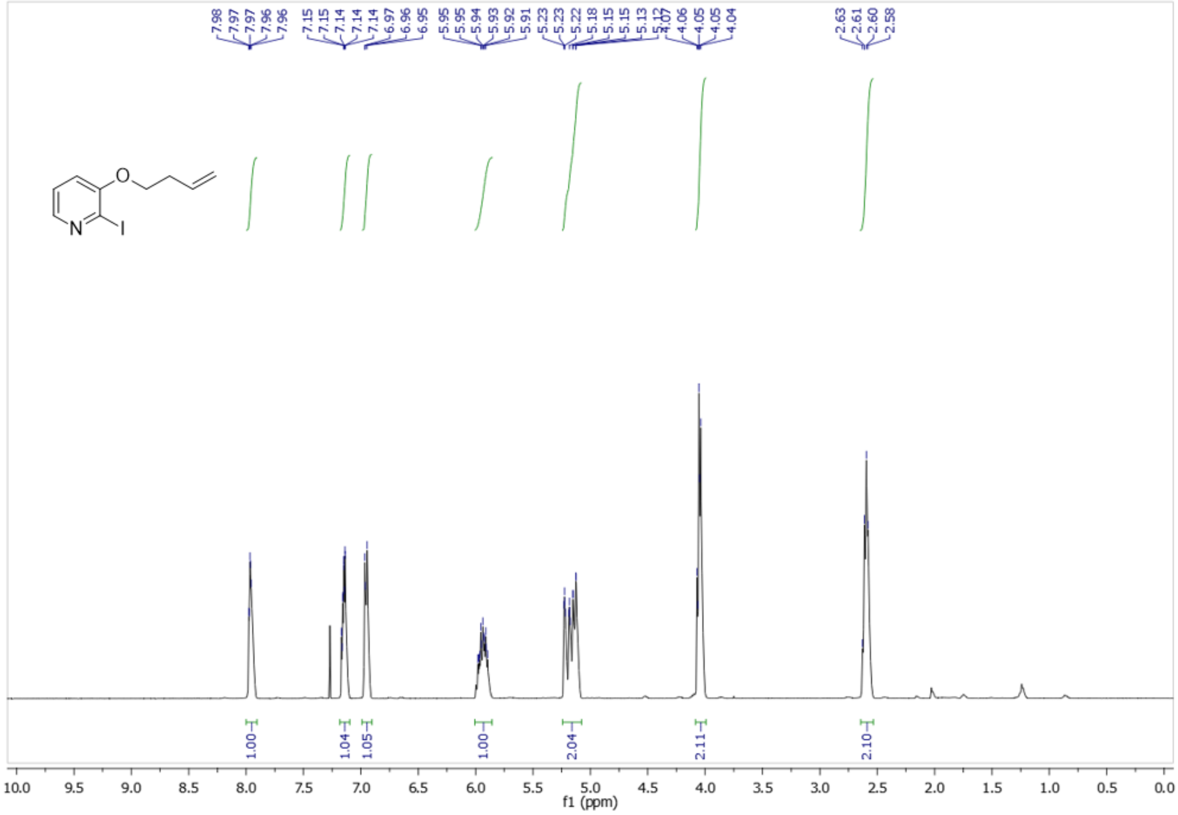

^{13}C NMR (101 MHz, $CDCl_3$):

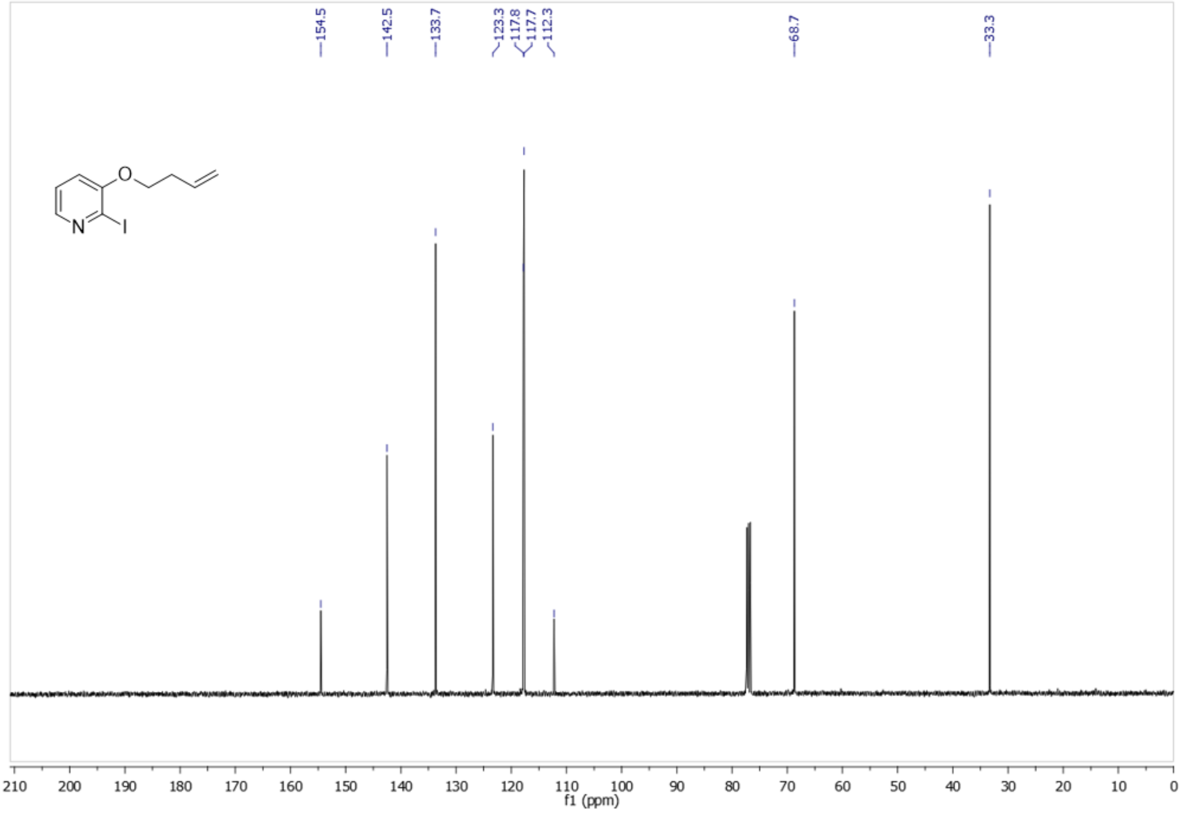

13.49 4-Methyl-3,4-dihydro-2*H*-pyrano[3,2-*b*]pyridine (21)

^1H NMR (400 MHz, $CDCl_3$):

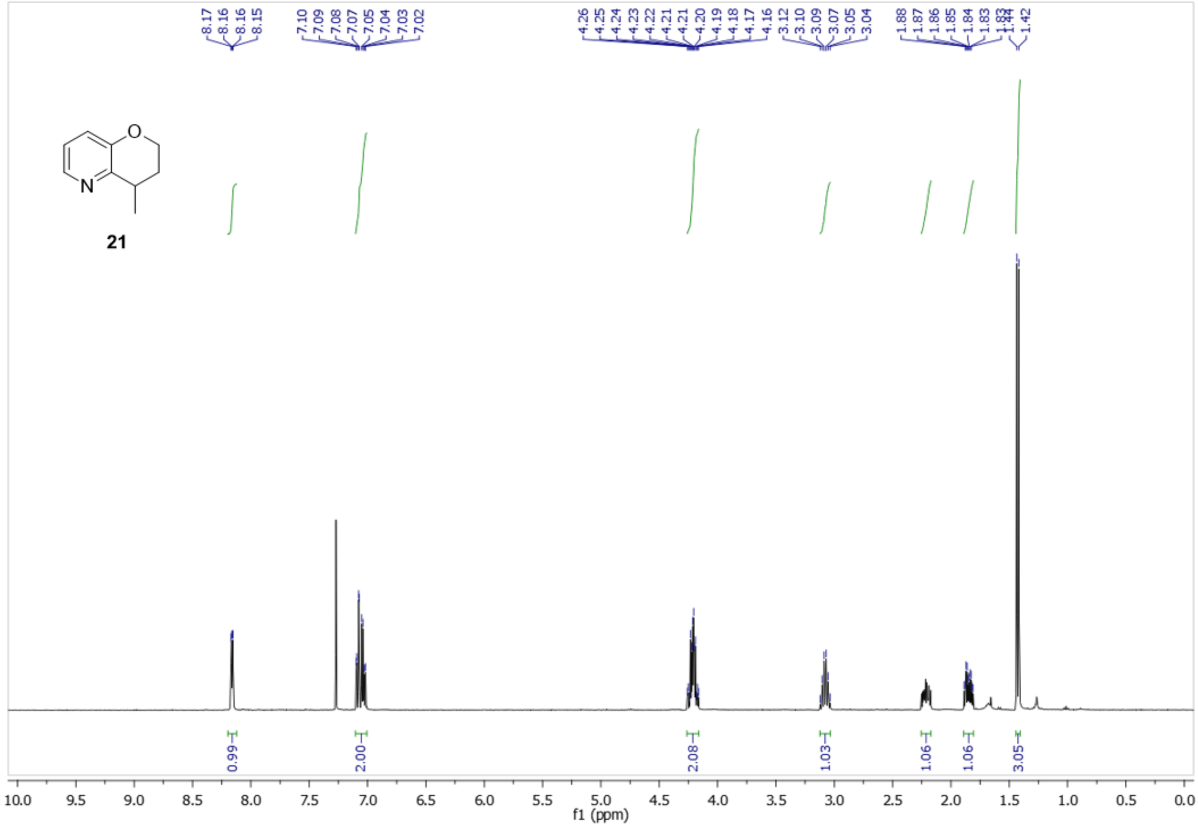

^{13}C NMR (101 MHz, $CDCl_3$):

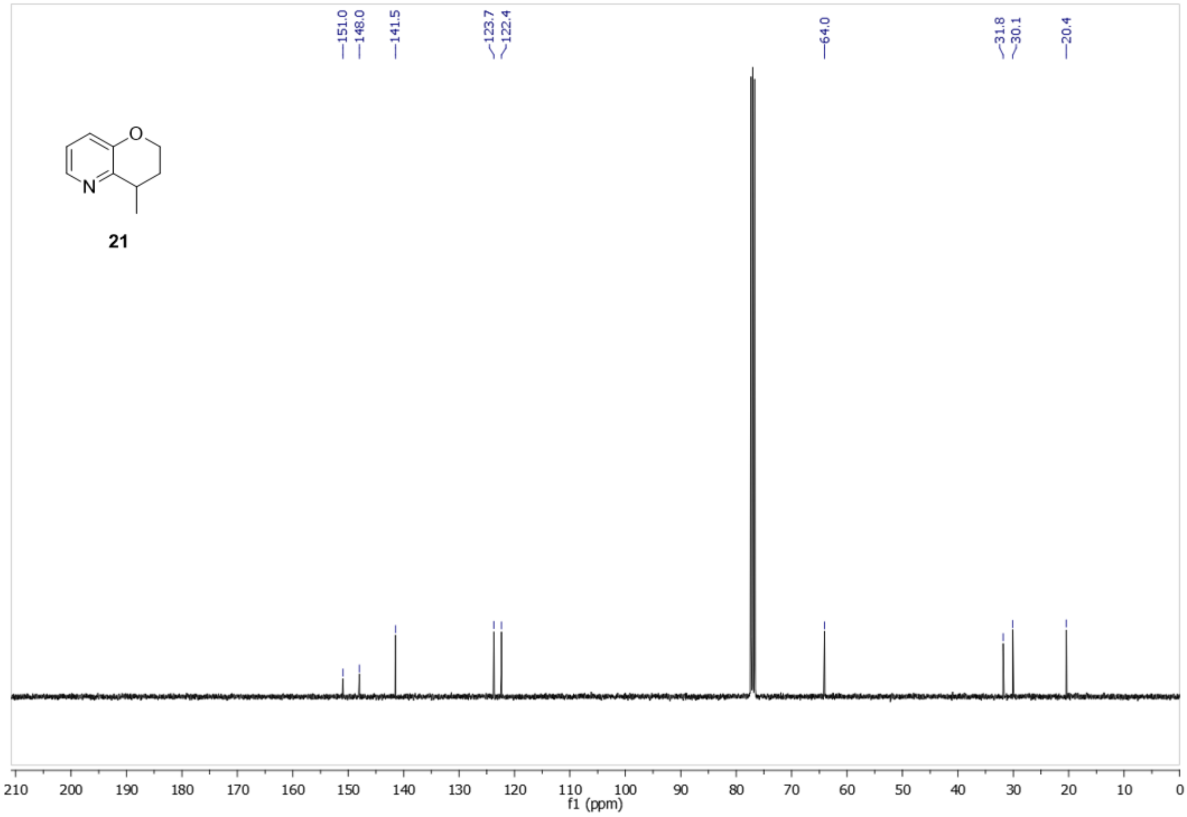

13.50 Ethyl (*E*)-5-(2-bromophenyl)pent-2-enoate

^{1}H NMR (400 MHz, $CDCl_3$):

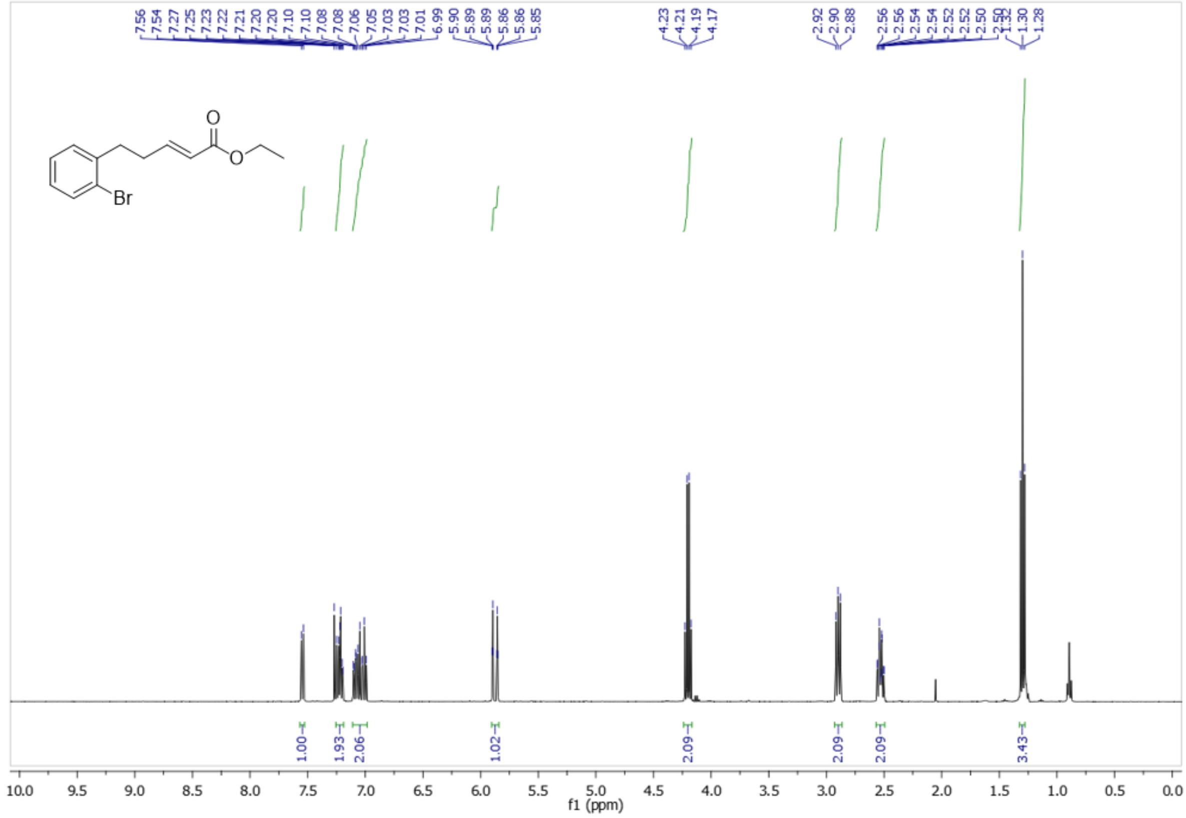

^{13}C NMR (101 MHz, $CDCl_3$):

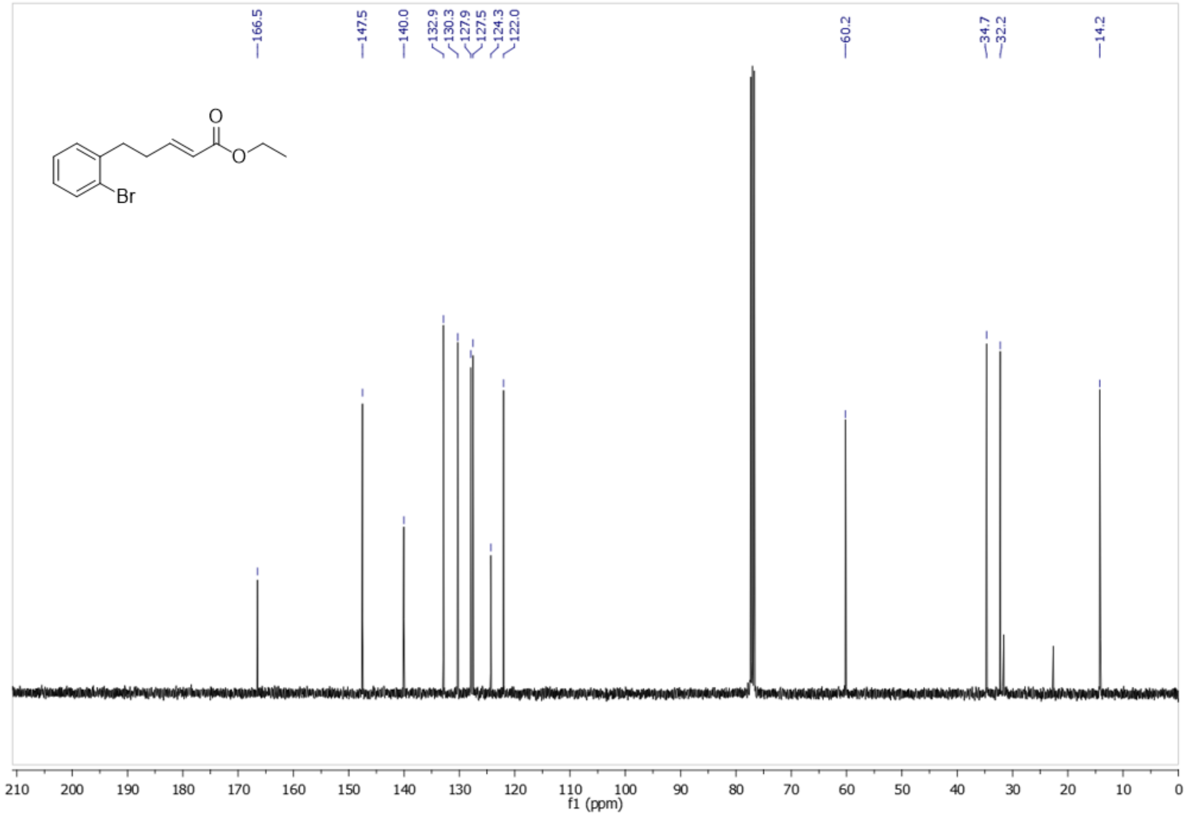

13.51 Ethyl 2-(2,3-dihydro-1*H*-inden-1-yl)acetate (22)

^{1}H NMR (400 MHz, $CDCl_3$):

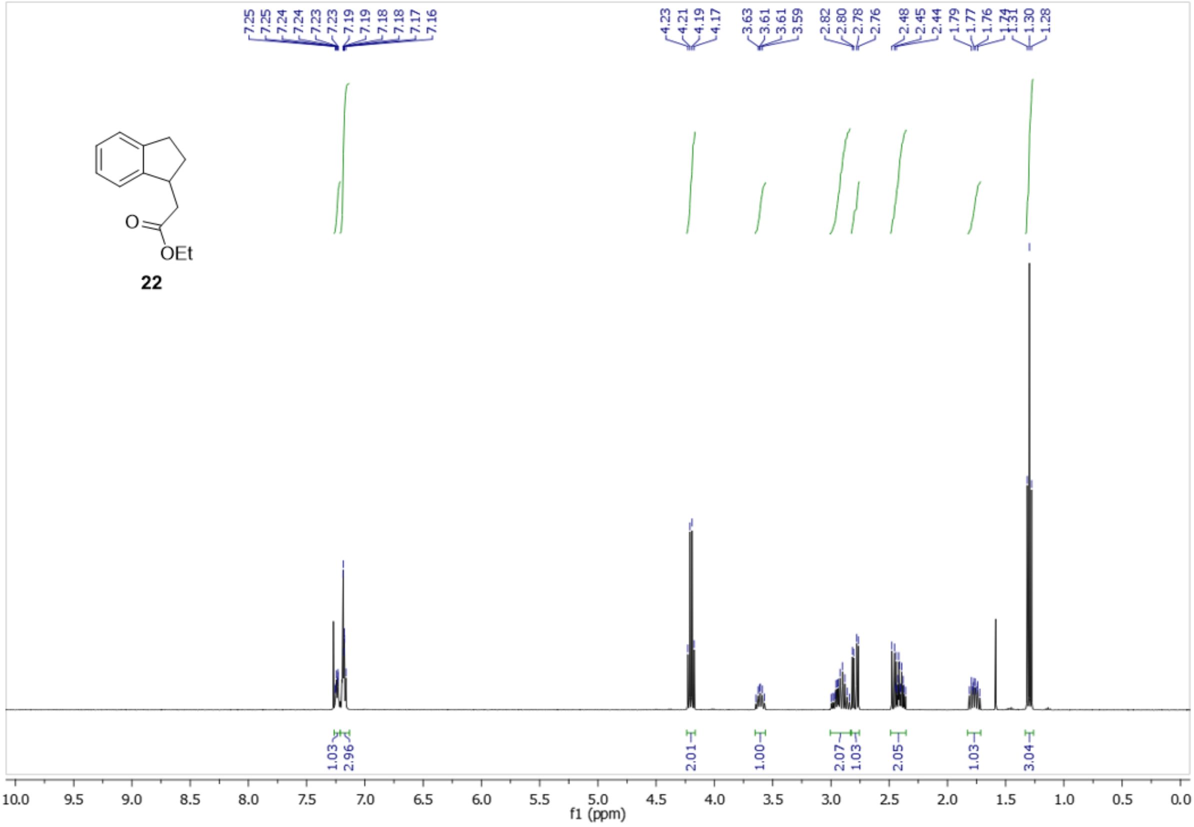

^{13}C NMR (101 MHz, $CDCl_3$):

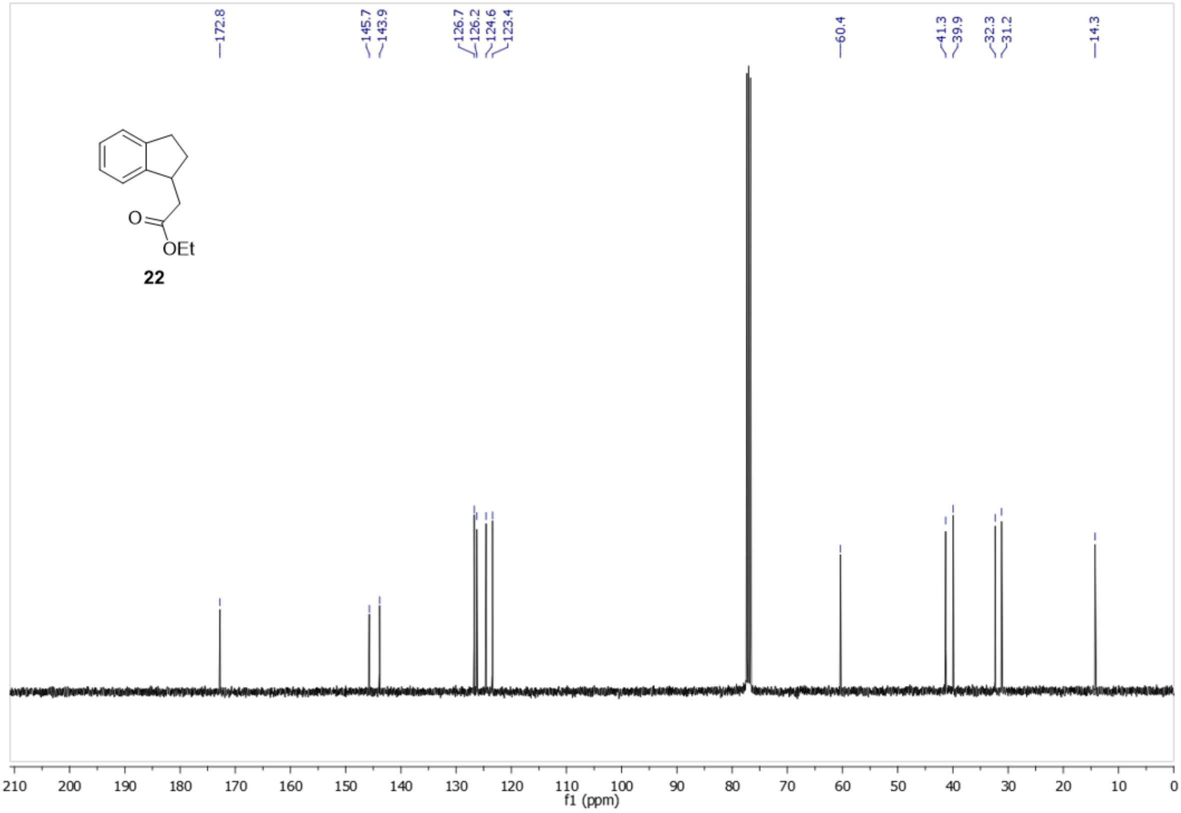

13.52 1-(6-Bromobenzo[*d*][1,3]dioxol-5-yl)-2,2-dimethylbut-3-en-1-ol

^{1}H NMR (400 MHz, $CDCl_3$):

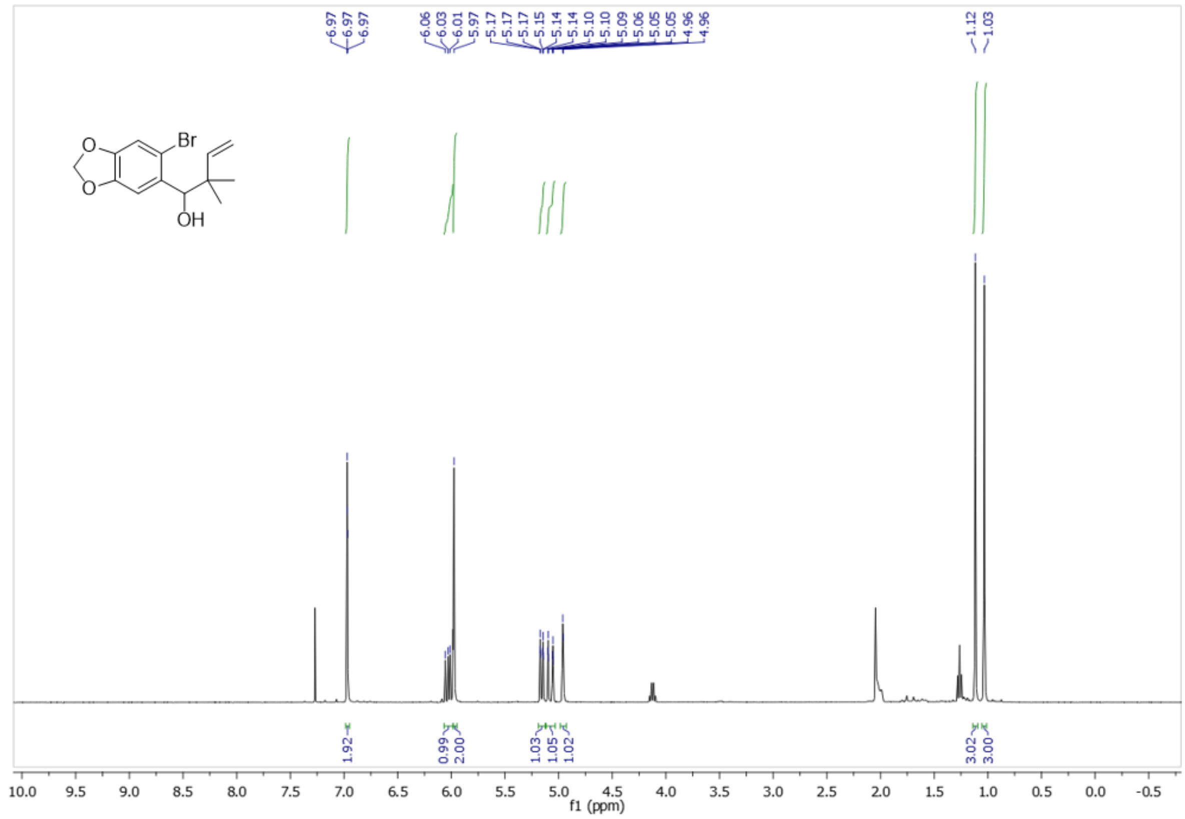

^{13}C NMR (101 MHz, $CDCl_3$):

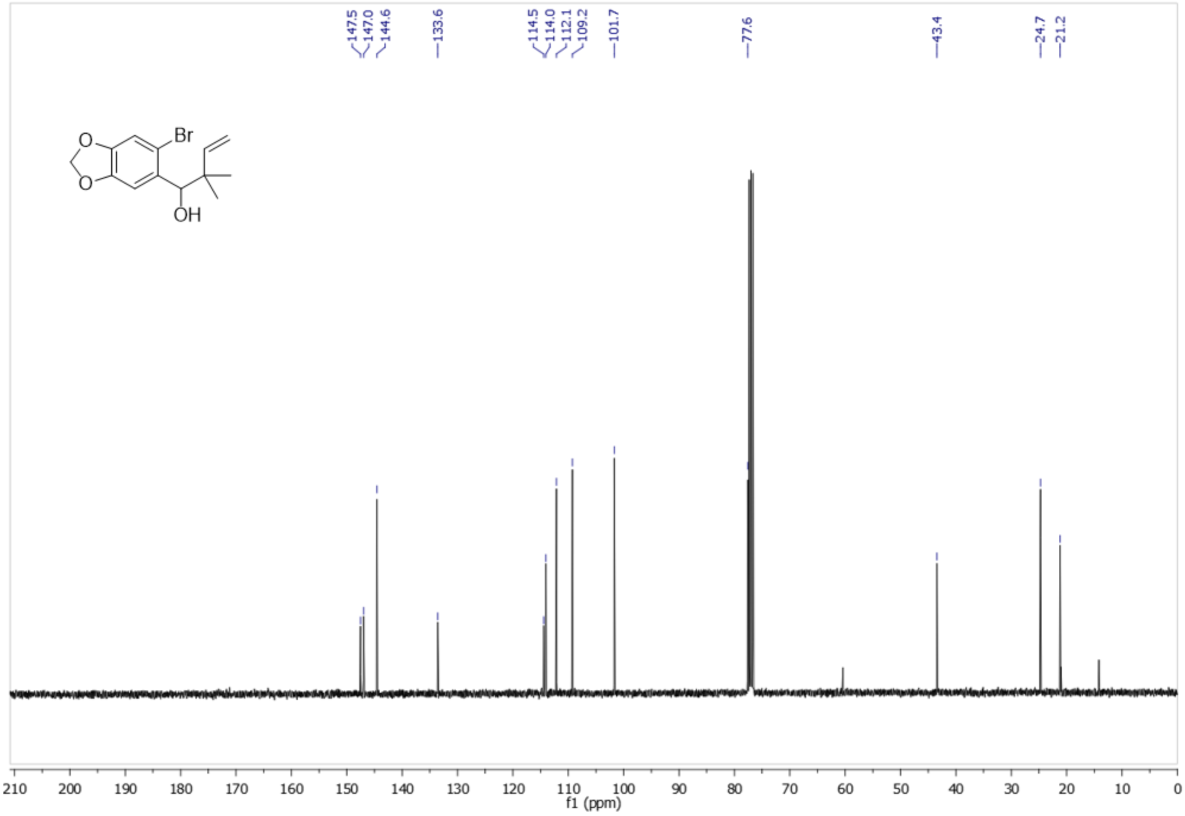

13.53 6,6,7-Trimethyl-6,7-dihydro-5*H*-indeno[5,6-*d*][1,3]dioxol-5-ol (23, dr ~ 3:2)

^{1}H NMR (400 MHz, $CDCl_3$):

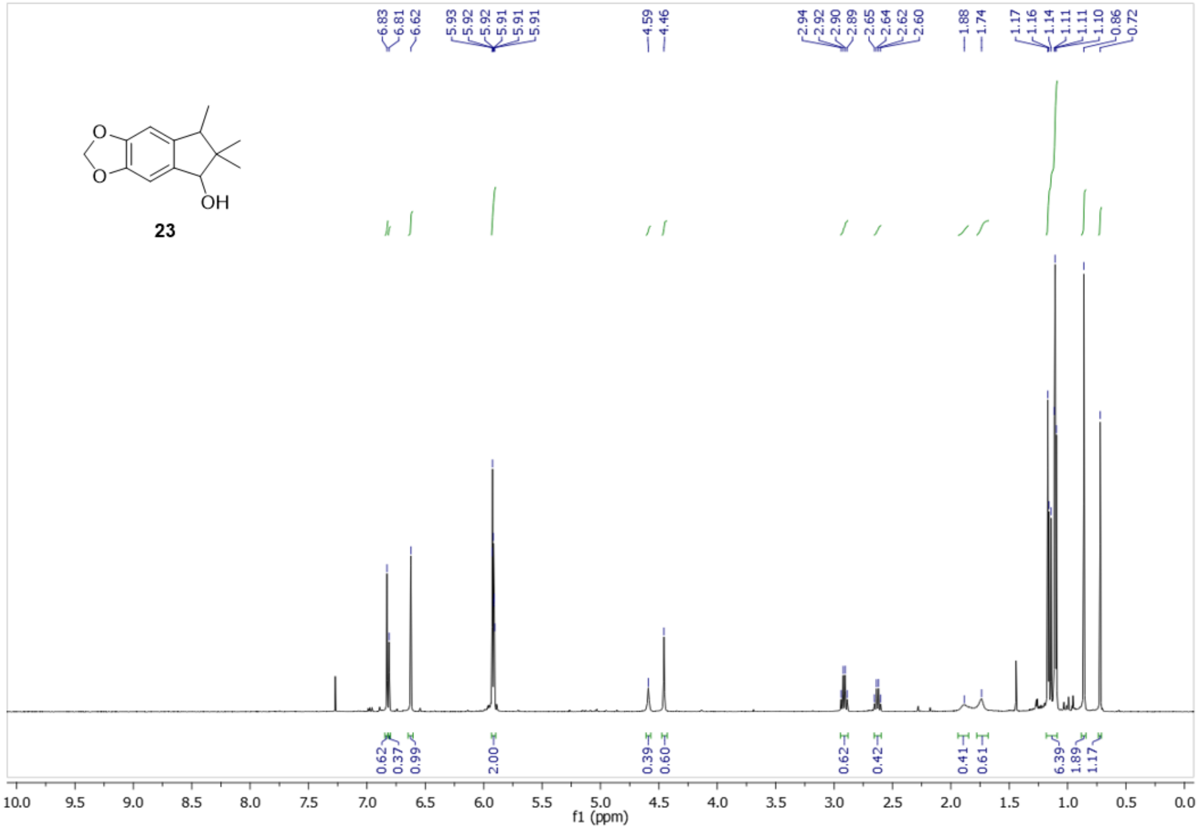

^{13}C NMR (101 MHz, $CDCl_3$):

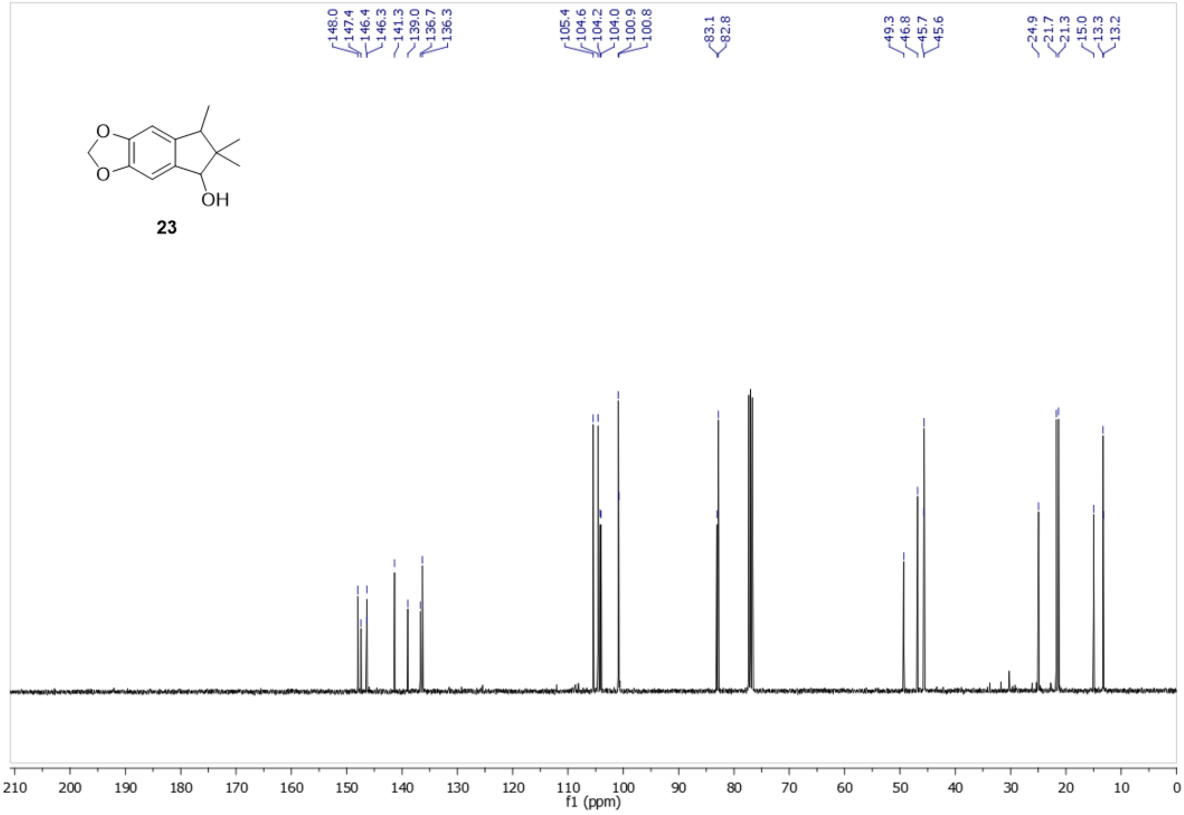

13.54 1-(6-Bromobenzo[*d*][1,3]dioxol-5-yl)-2,2-dimethylbut-3-en-1-one

^{1}H NMR (400 MHz, $CDCl_3$):

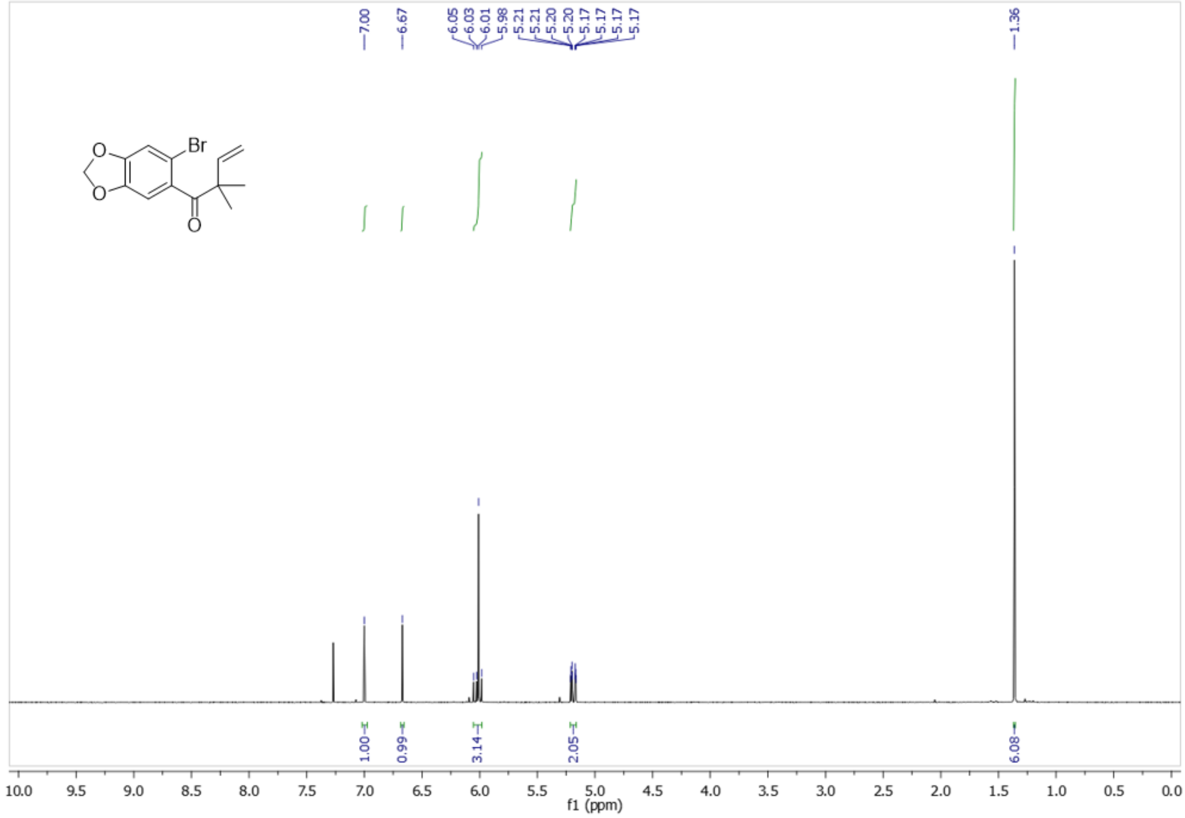

^{13}C NMR (101 MHz, $CDCl_3$):

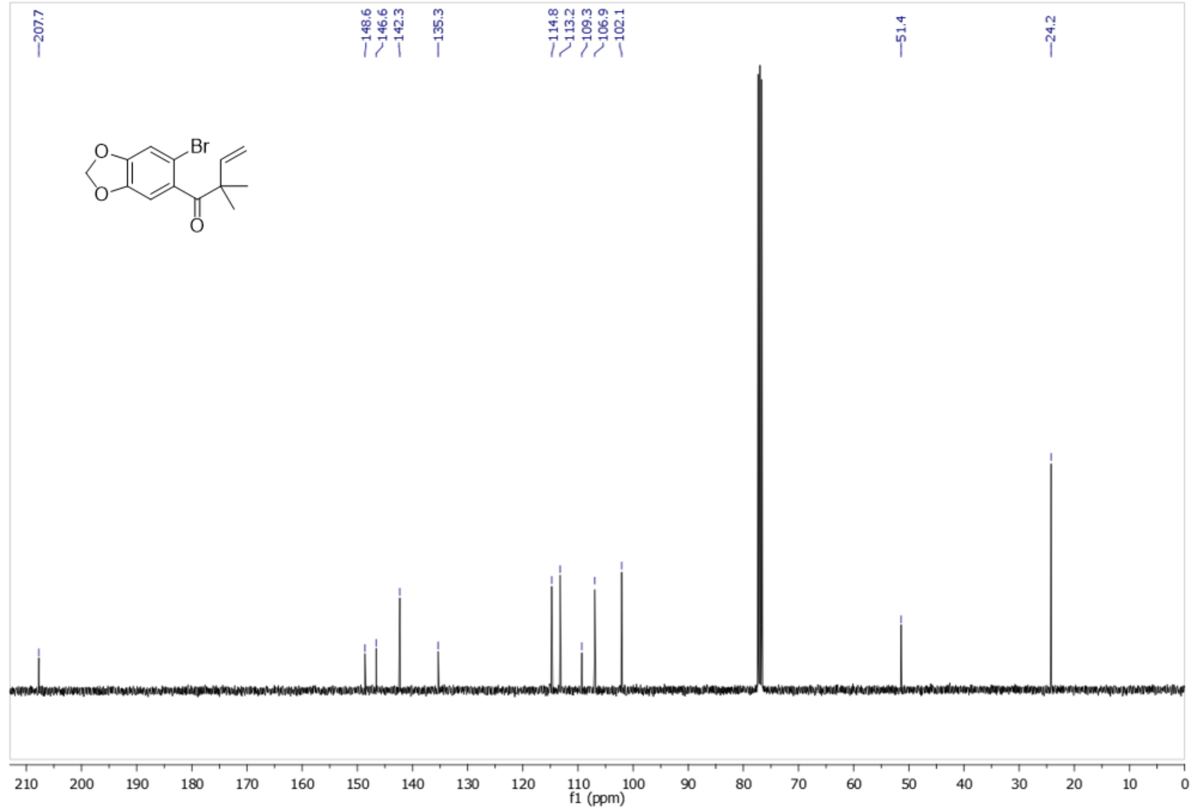

13.55 6,6,7-Trimethyl-6,7-dihydro-5*H*-indeno[5,6-*d*][1,3]dioxol-5-one (24)

^{1}H NMR (400 MHz, $CDCl_3$):

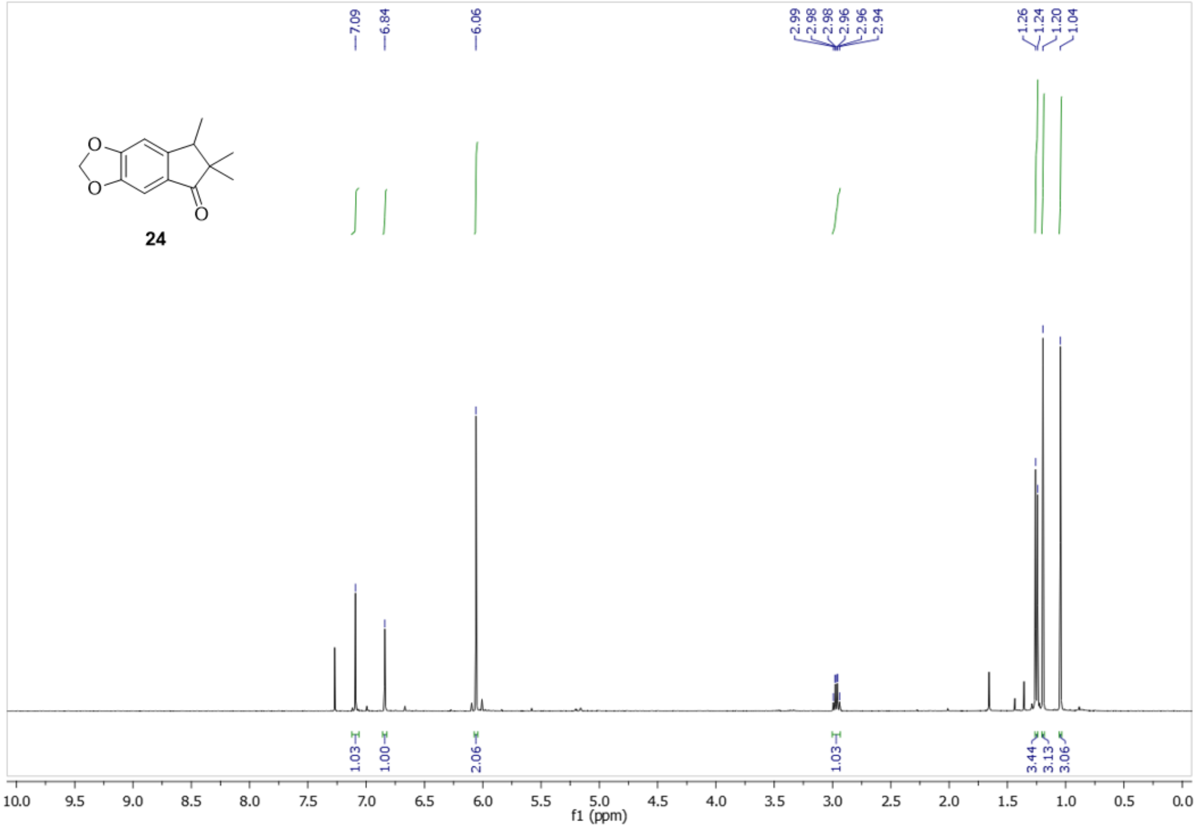

^{13}C NMR (101 MHz, $CDCl_3$):

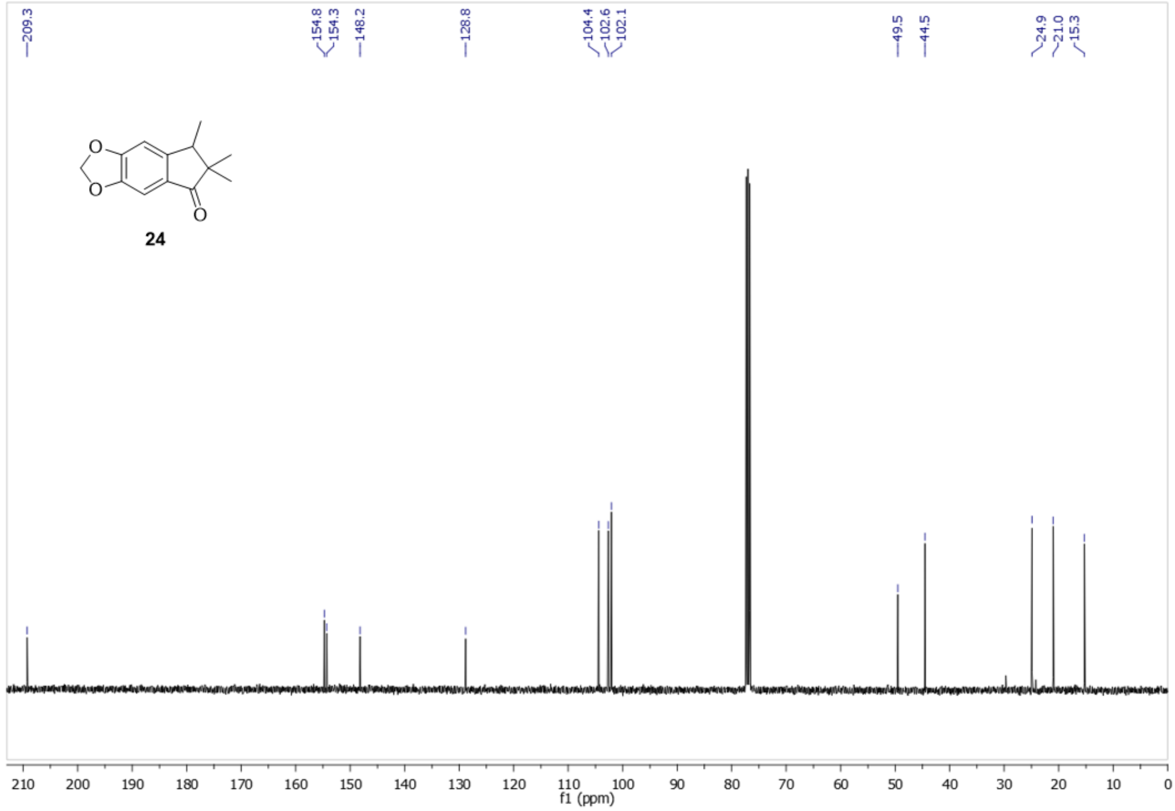

13.56 *tert*-Butyl (2-iodophenyl)carbamate

^{1}H NMR (400 MHz, $CDCl_3$):

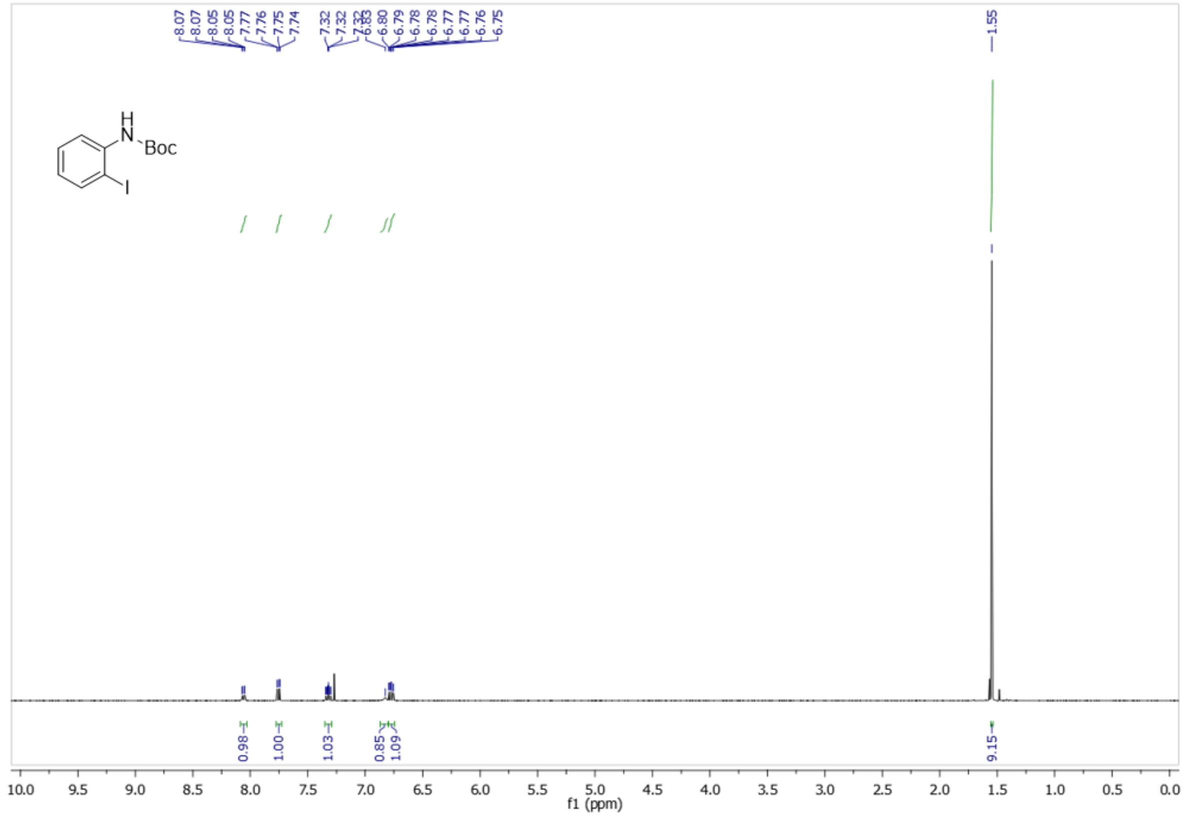

^{13}C NMR (101 MHz, $CDCl_3$):

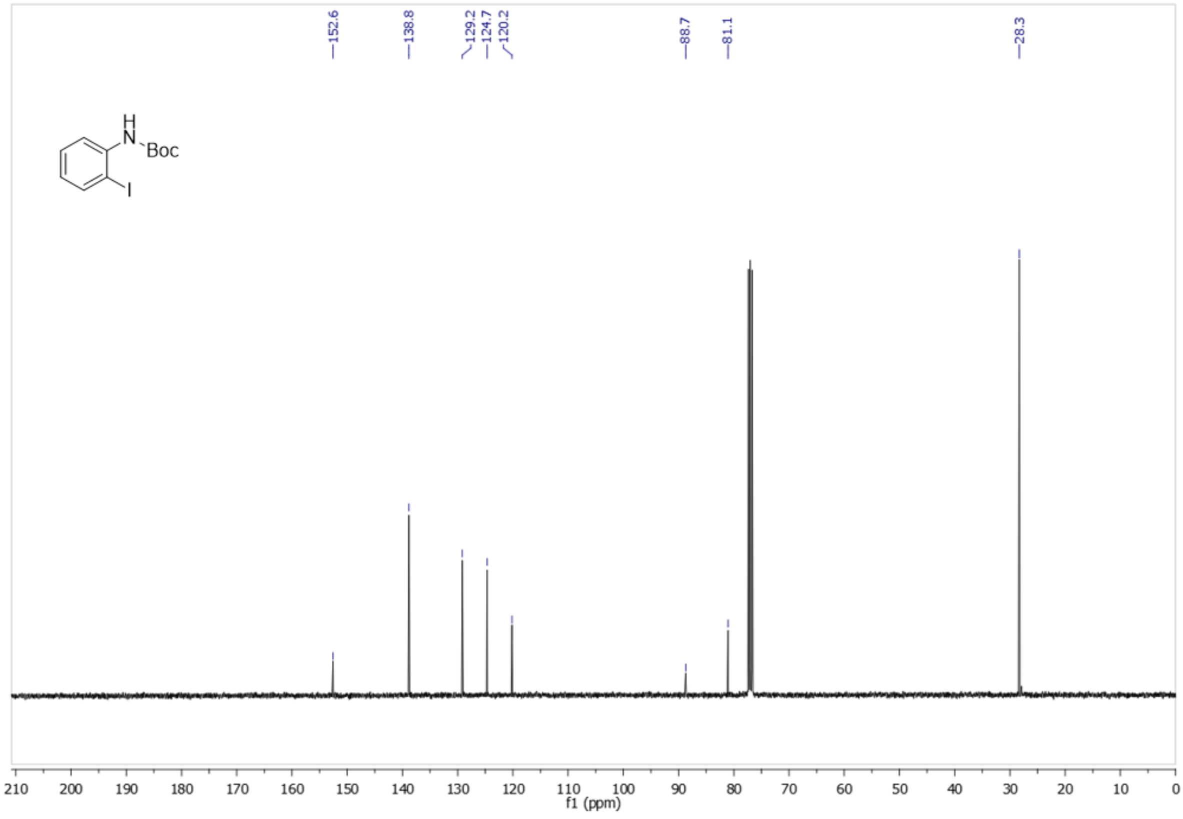

13.57 *tert*-Butyl cyclohex-2-en-1-yl(2-iodophenyl)carbamate

^{1}H NMR (500 MHz, 363 K, DMSO-d_6):

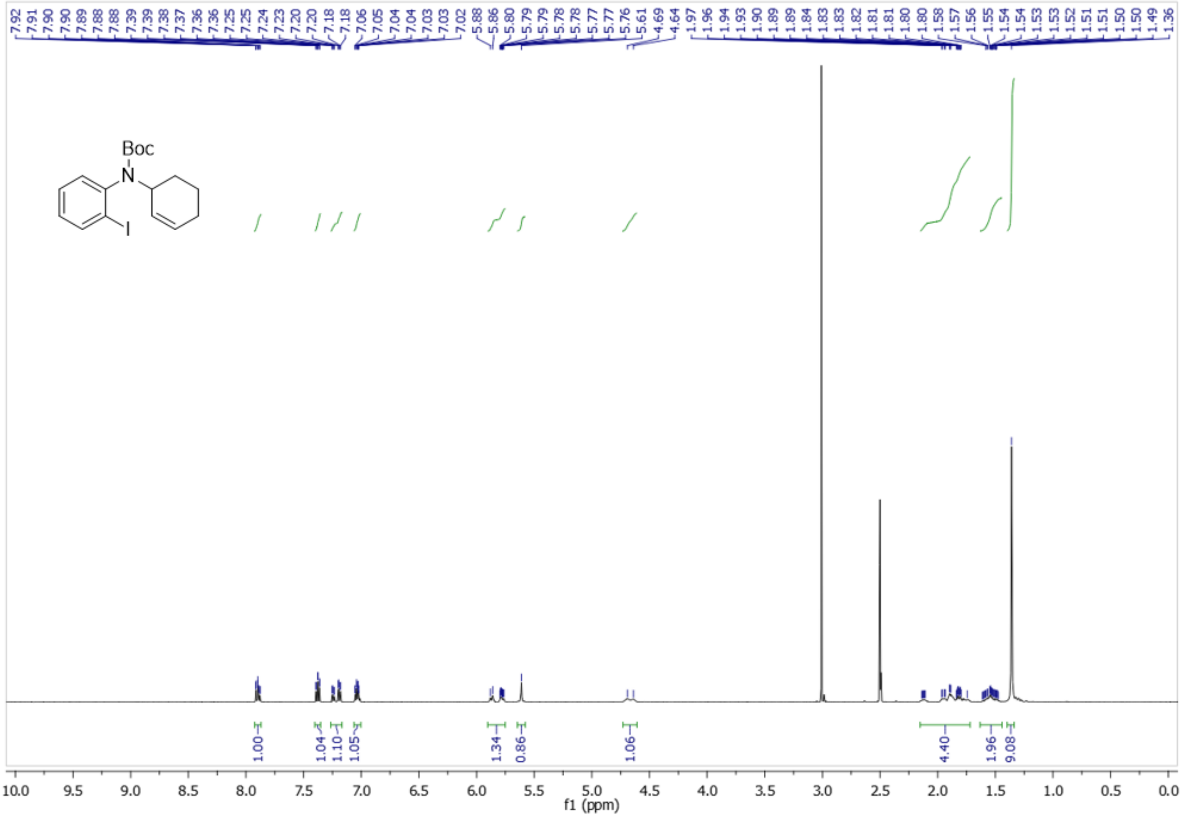

^{13}C NMR (126 MHz, 363 K, DMSO-d_6):

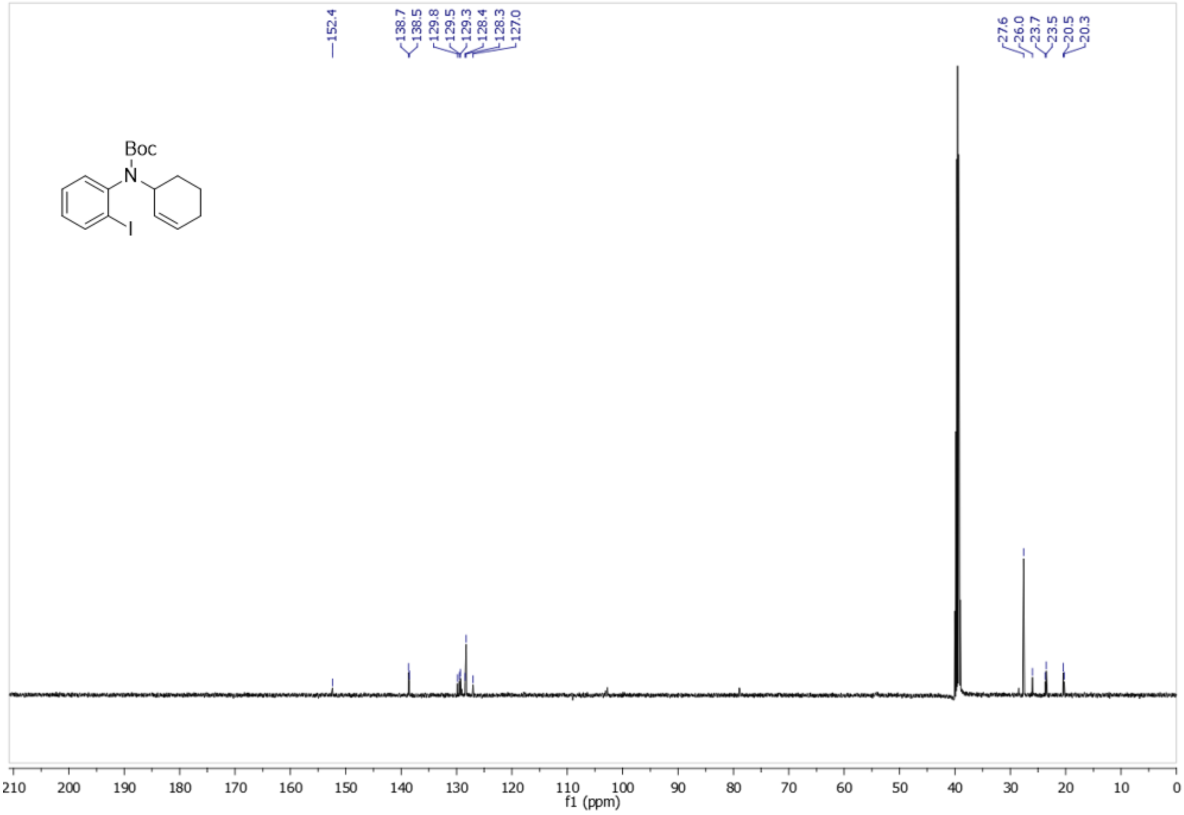

13.58 *tert*-Butyl 1,2,3,4,4a,9a-hexahydro-9*H*-carbazole-9-carboxylate (25)

^{1}H NMR (400 MHz, $CDCl_3$):

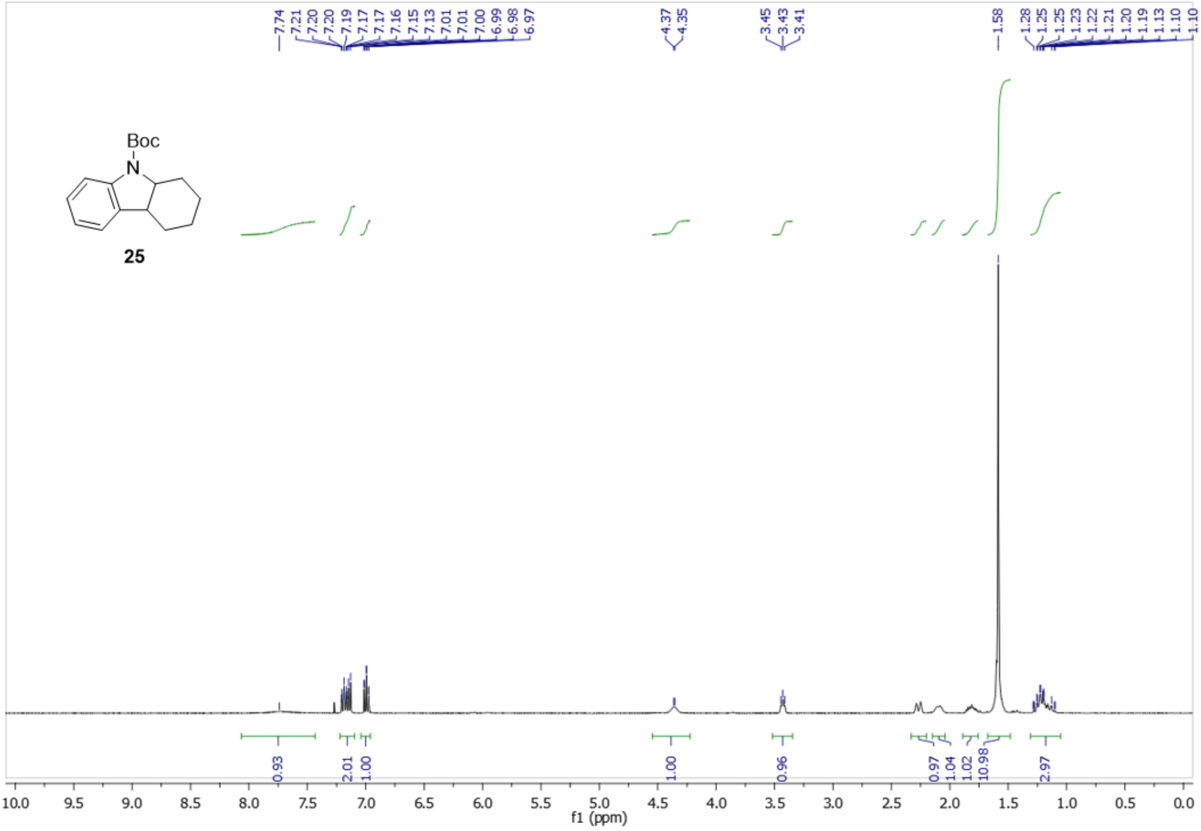

^{13}C NMR (101 MHz, $CDCl_3$):

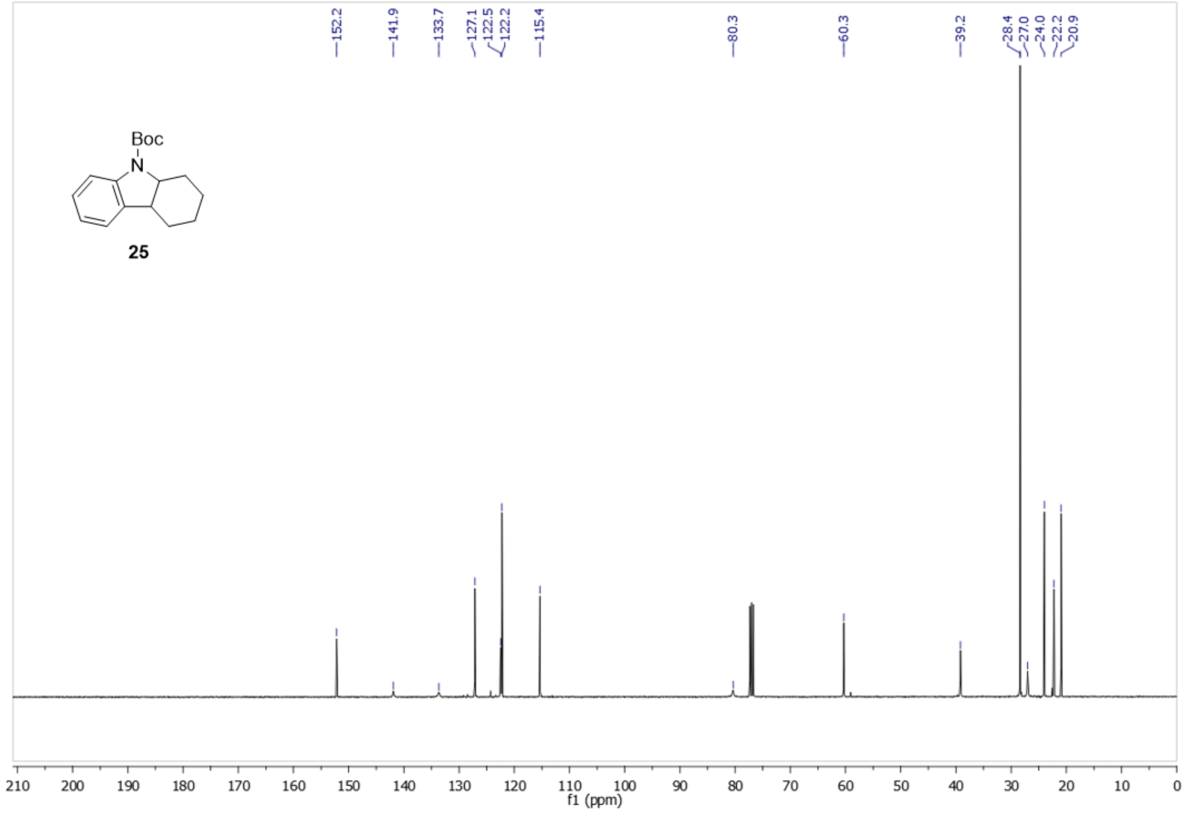

^{1}H NMR (500 MHz, 323 K, DMSO-d_6):

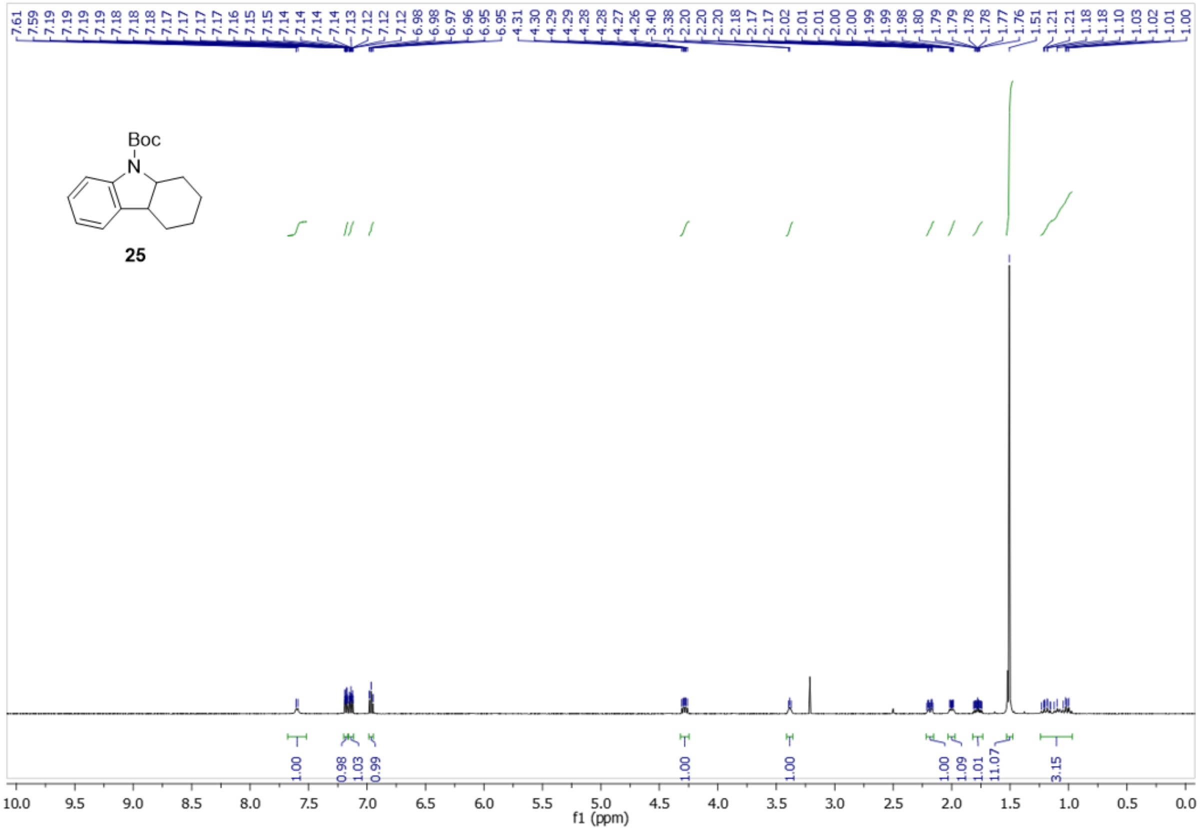

^{13}C NMR (126 MHz, 323 K, DMSO-d_6):

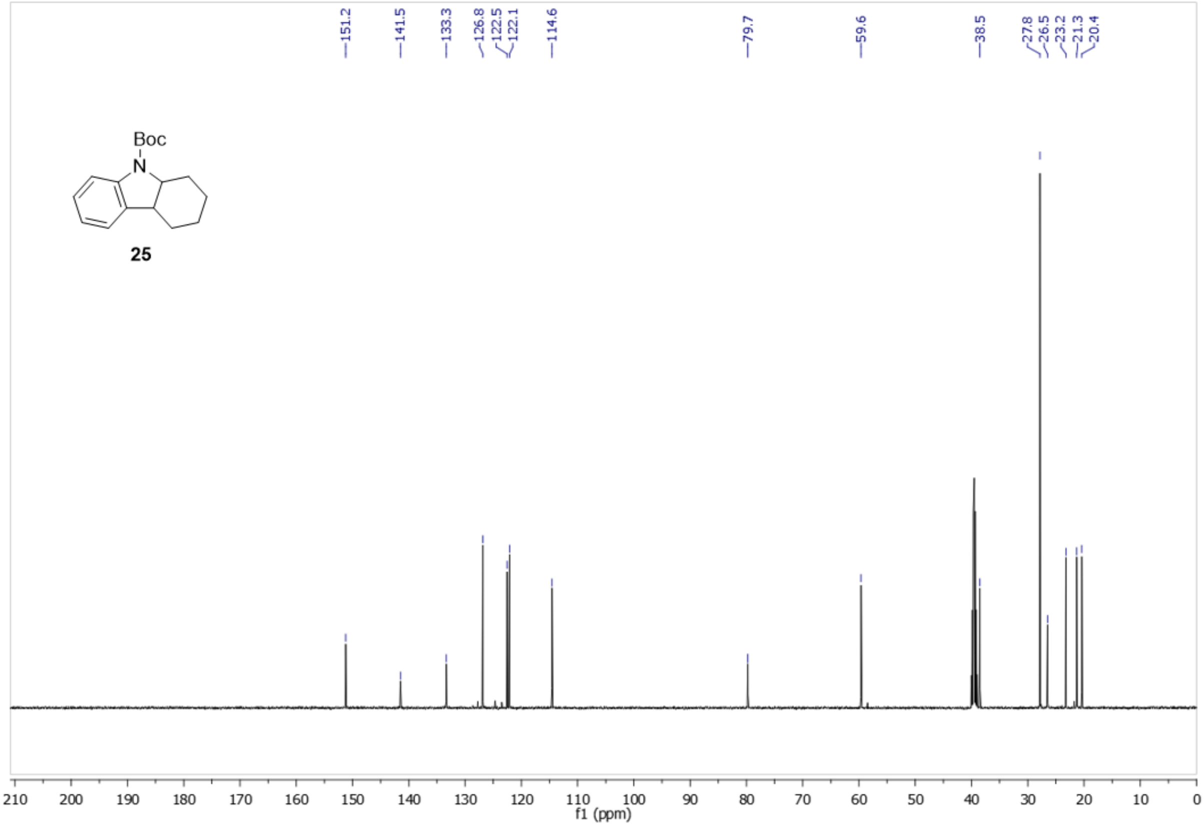

13.59 *tert*-Butyl but-3-en-1-yl(2-iodophenyl)carbamate

^{1}H NMR (500 MHz, 353 K, DMSO-d_6):

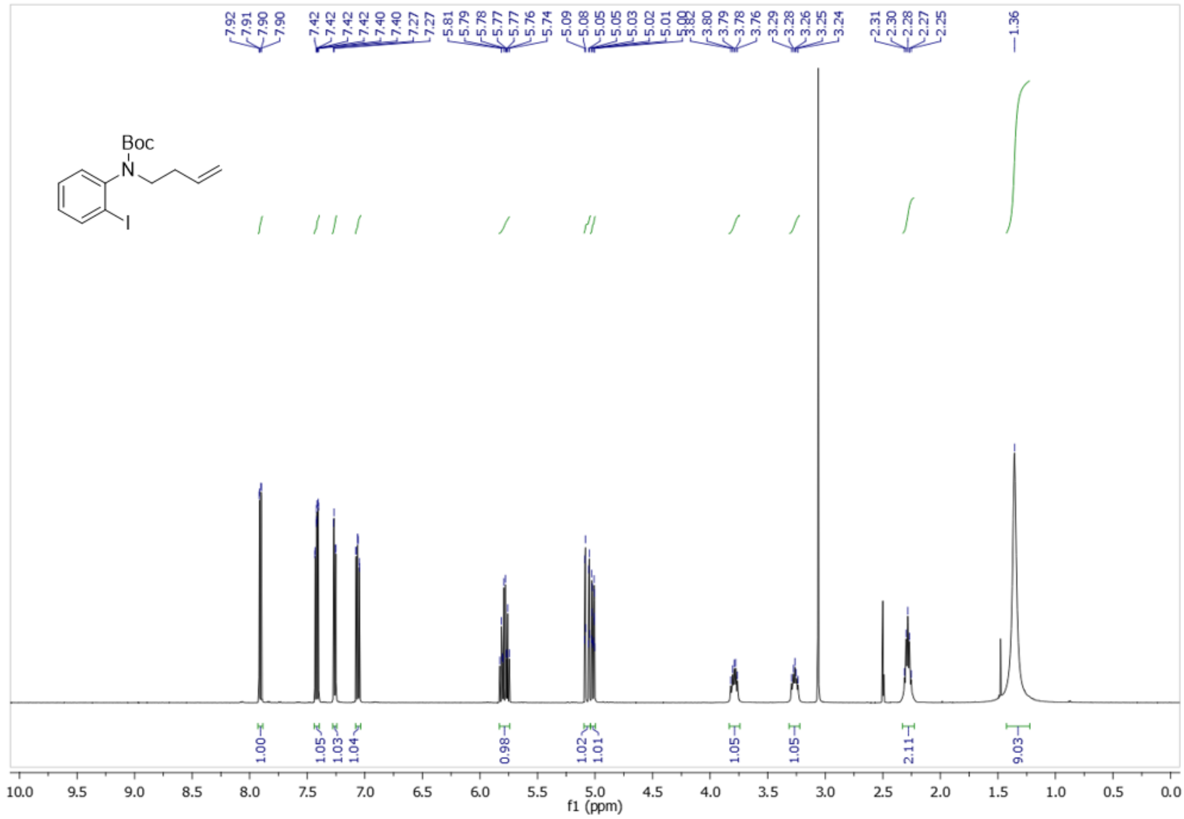

^{13}C NMR (126 MHz, 353 K, DMSO-d_6):

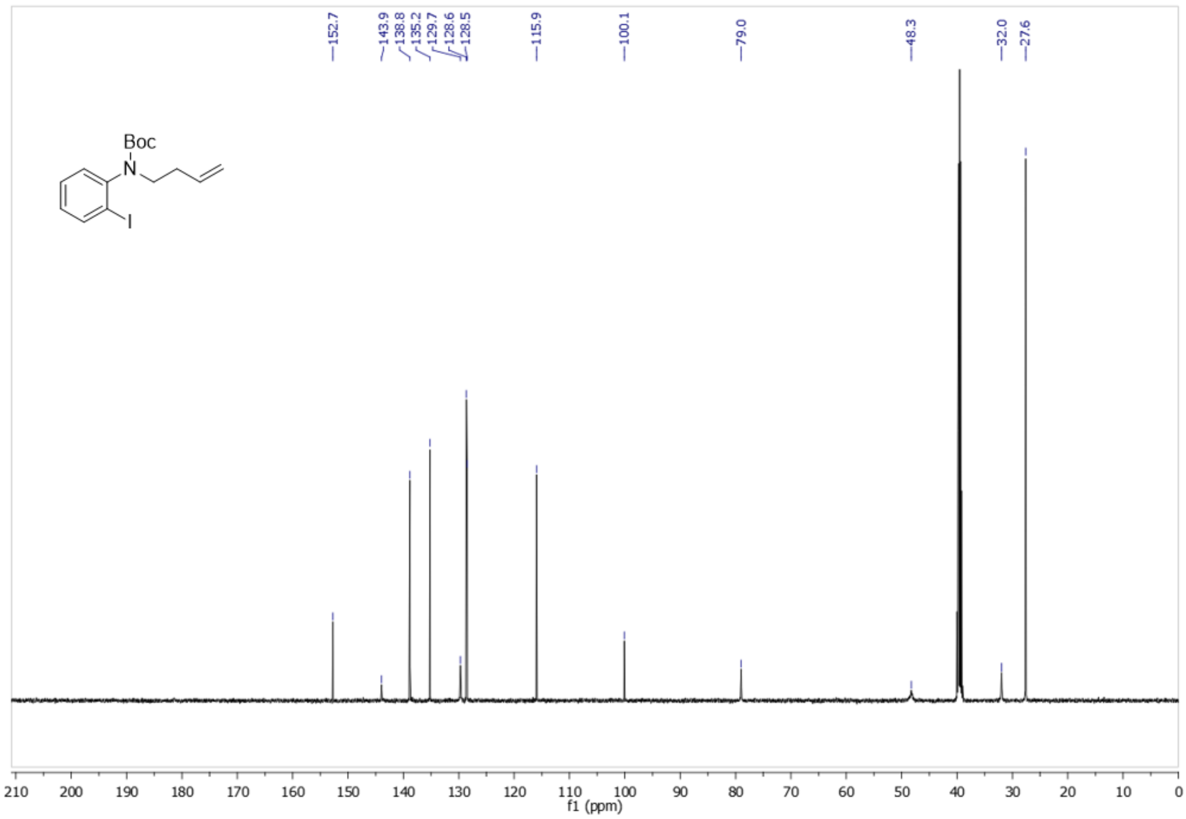

13.60 *tert*-Butyl 4-methyl-3,4-dihydroquinoline-1(2*H*)-carboxylate (26)

^{1}H NMR (500 MHz, 303 K, DMSO-d_6):

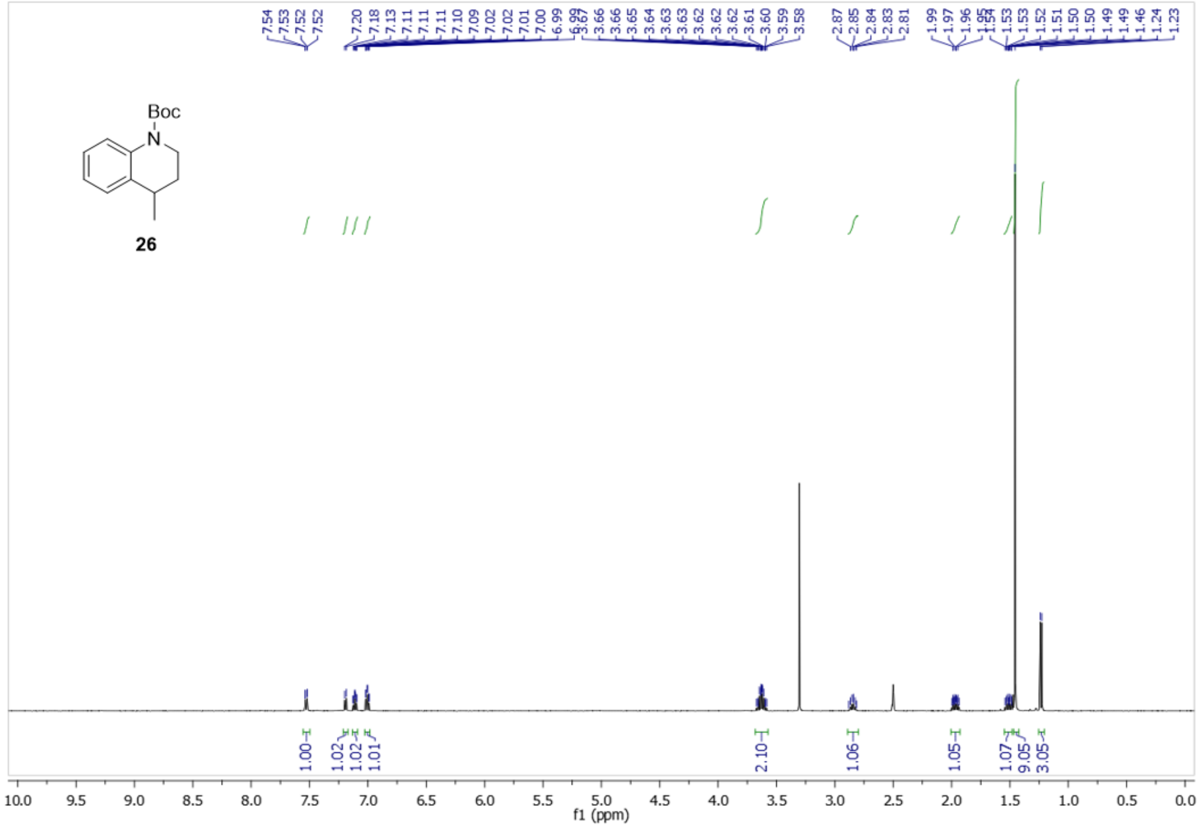

^{13}C NMR (126 MHz, 303 K, DMSO-d_6):

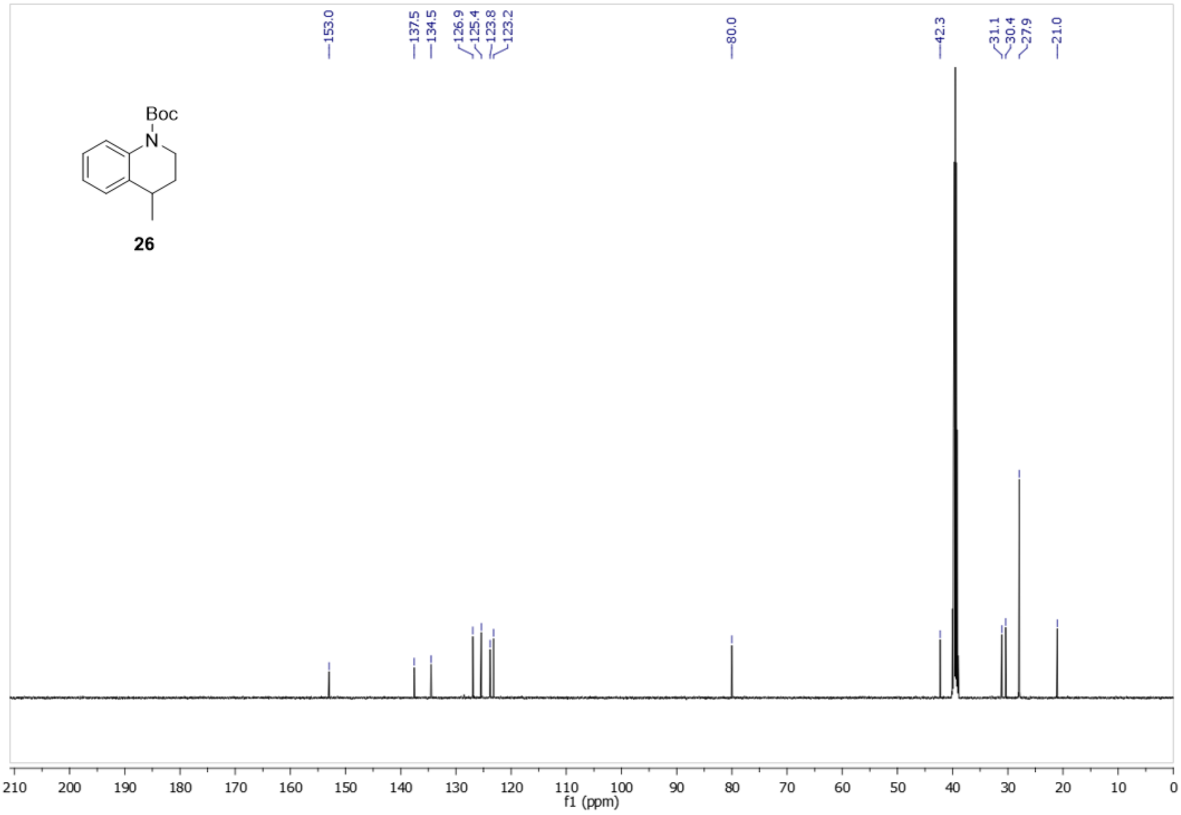

13.61 *tert*-Butyl allyl(2-iodophenyl)carbamate

^{1}H NMR (500 MHz, 353 K, DMSO-d_6):

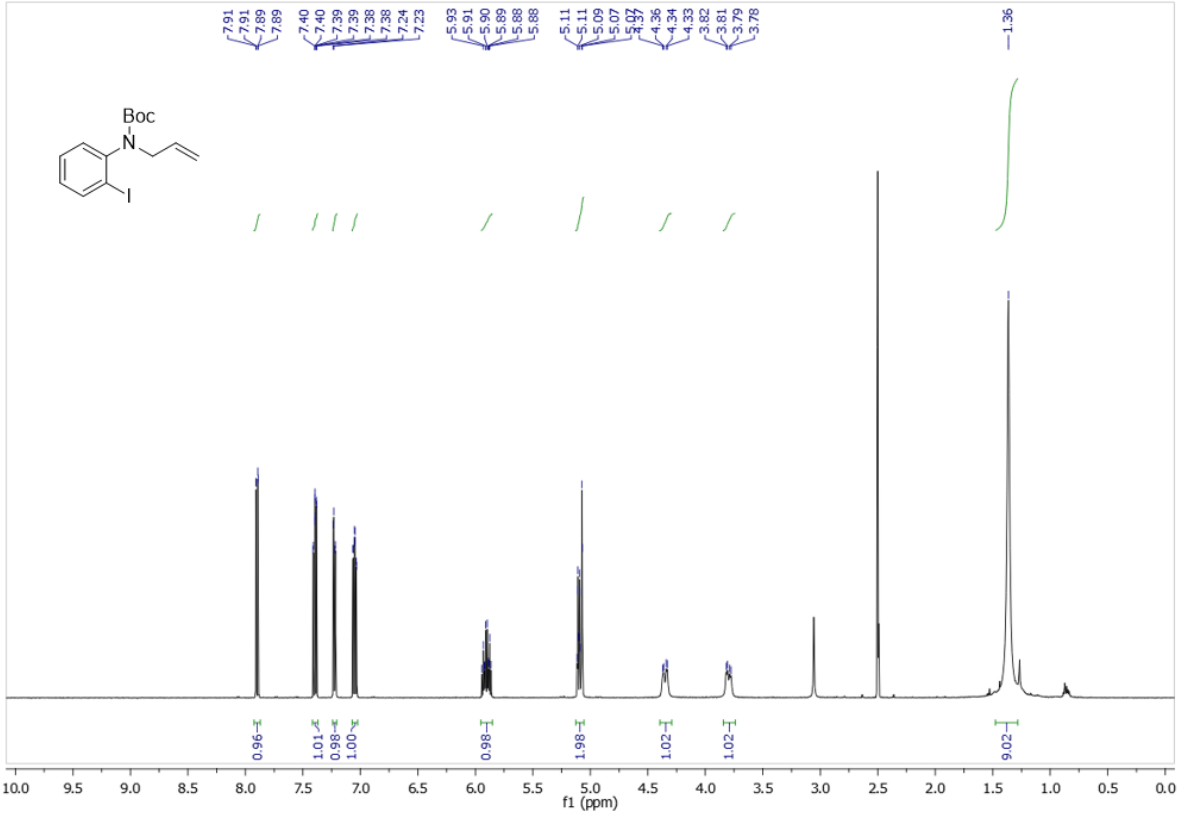

^{13}C NMR (126 MHz, 353 K, DMSO-d_6):

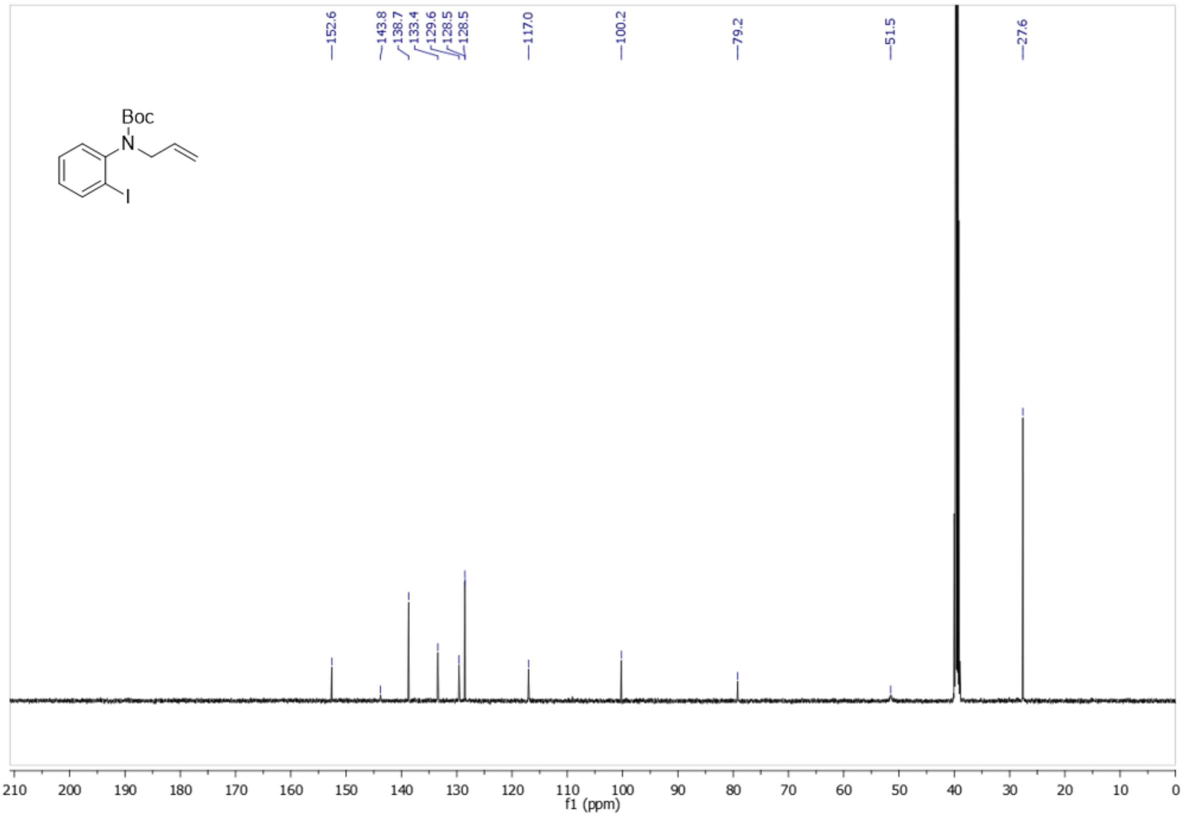

13.62 *tert*-Butyl 3-methylindoline-1-carboxylate (27)

^{1}H NMR (400 MHz, DMSO-d_6):

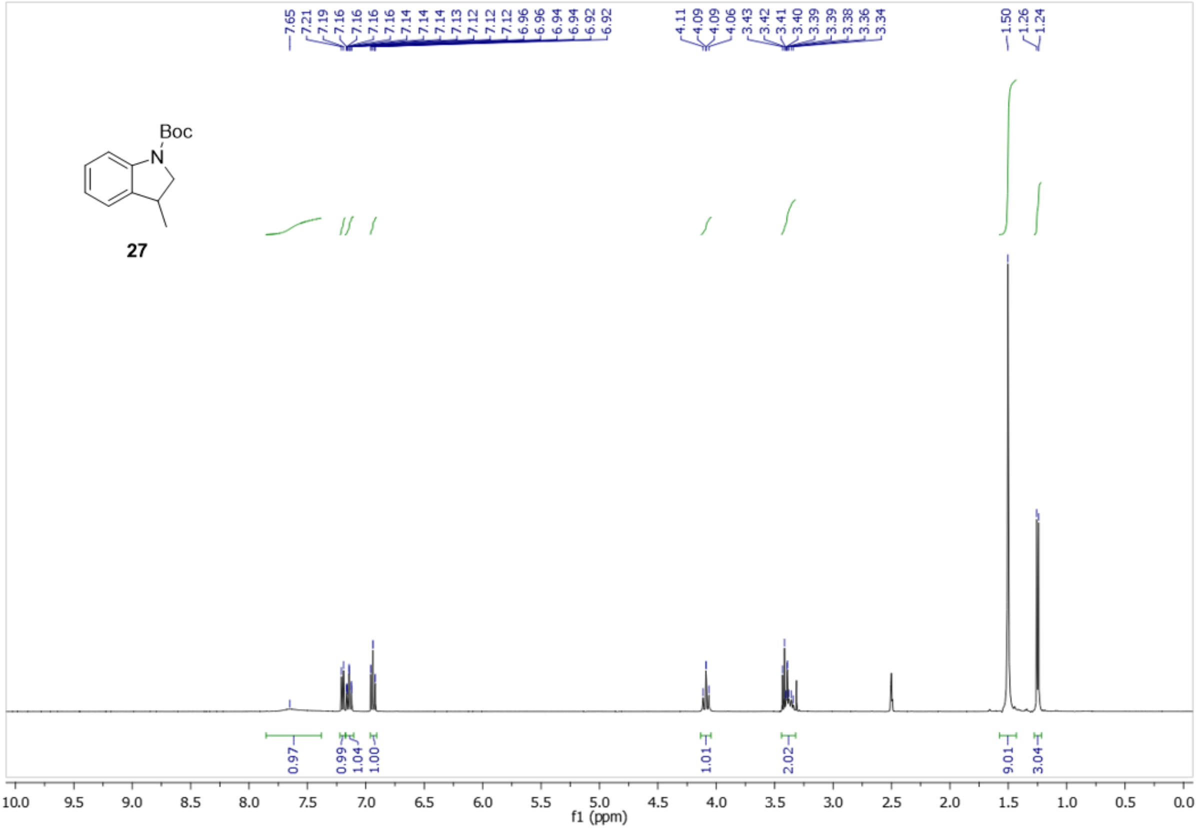

^{13}C NMR (101 MHz, DMSO-d_6):

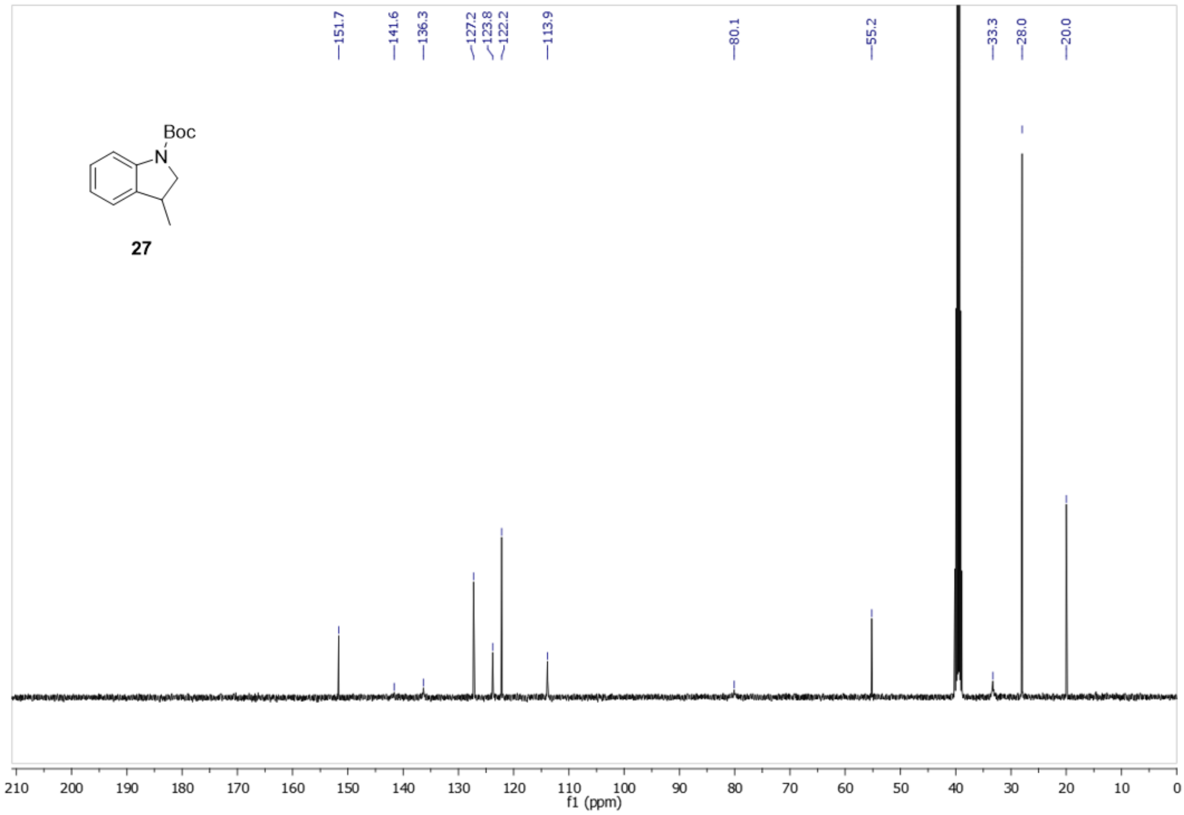

^{1}H NMR (500 MHz, 353 K, DMSO-d_6):

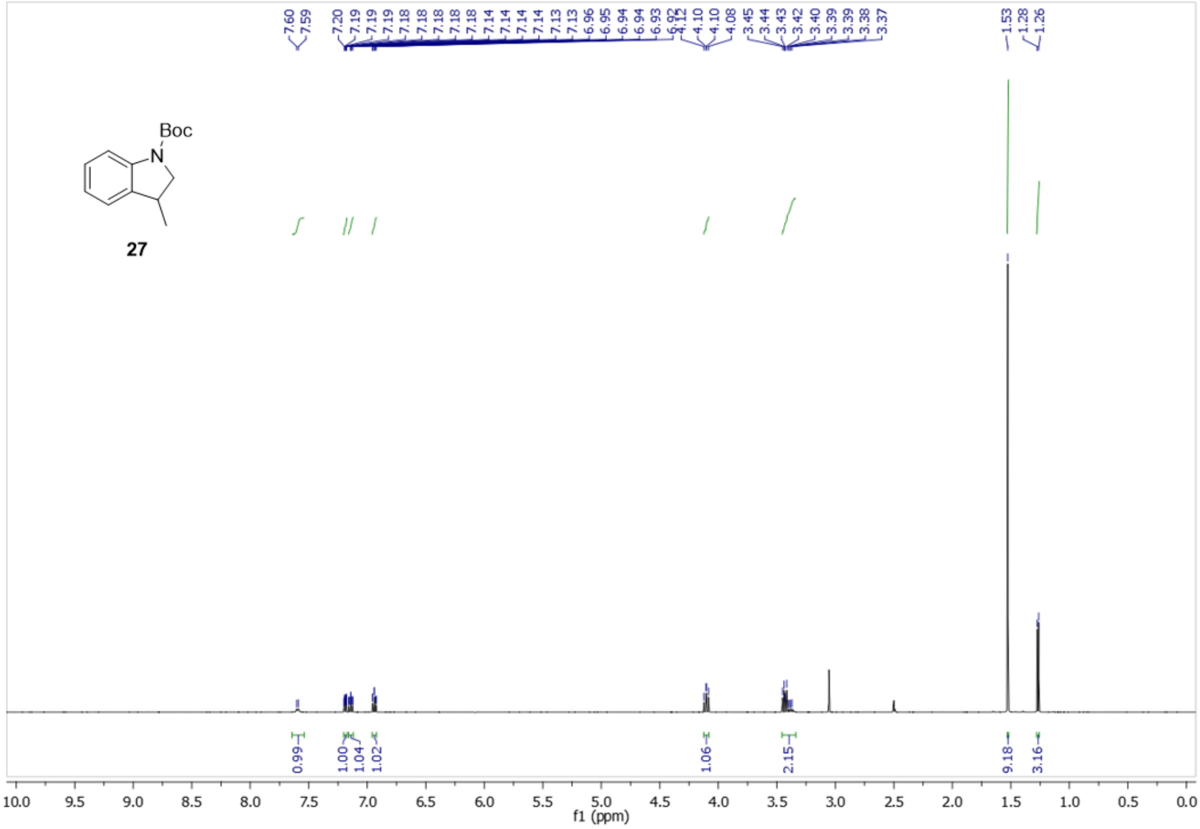

^{13}C NMR (126 MHz, 353 K, DMSO-d_6):

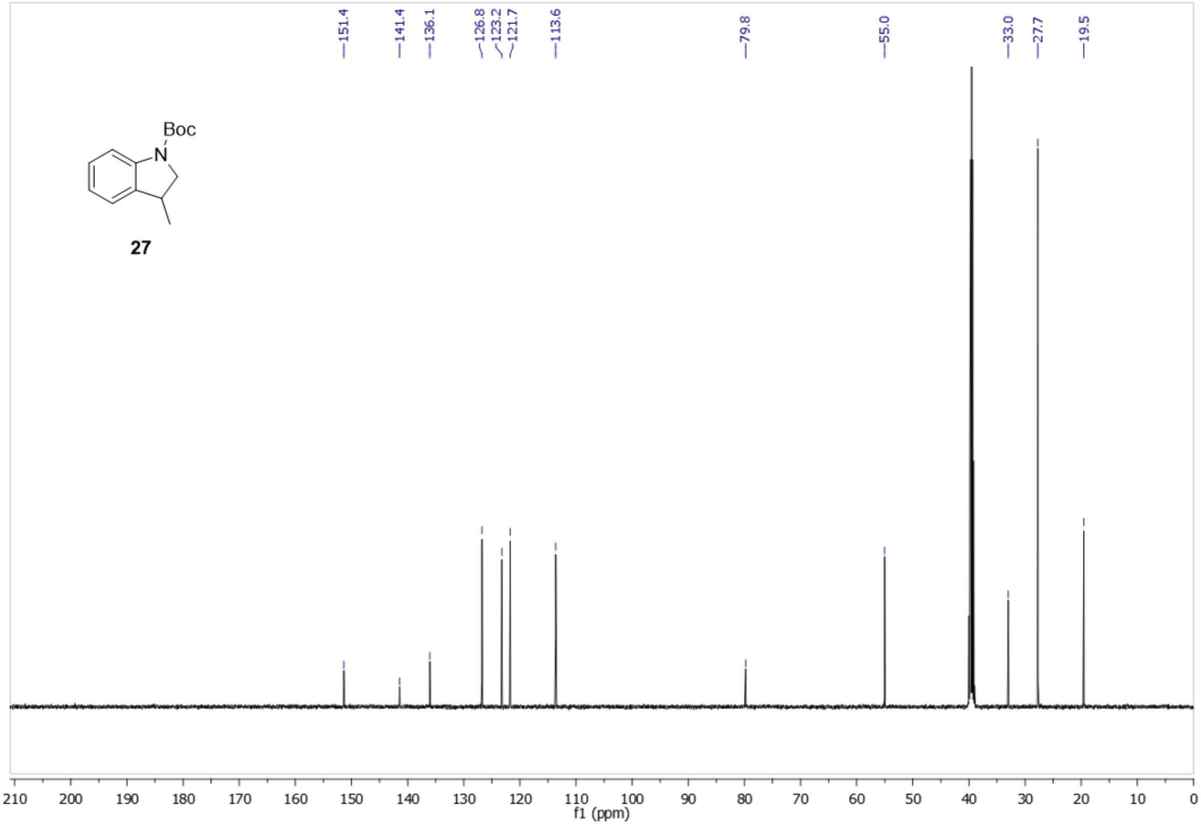

13.63 2-Iodo-*N*-methylaniline

^{1}H NMR (400 MHz, $CDCl_3$):

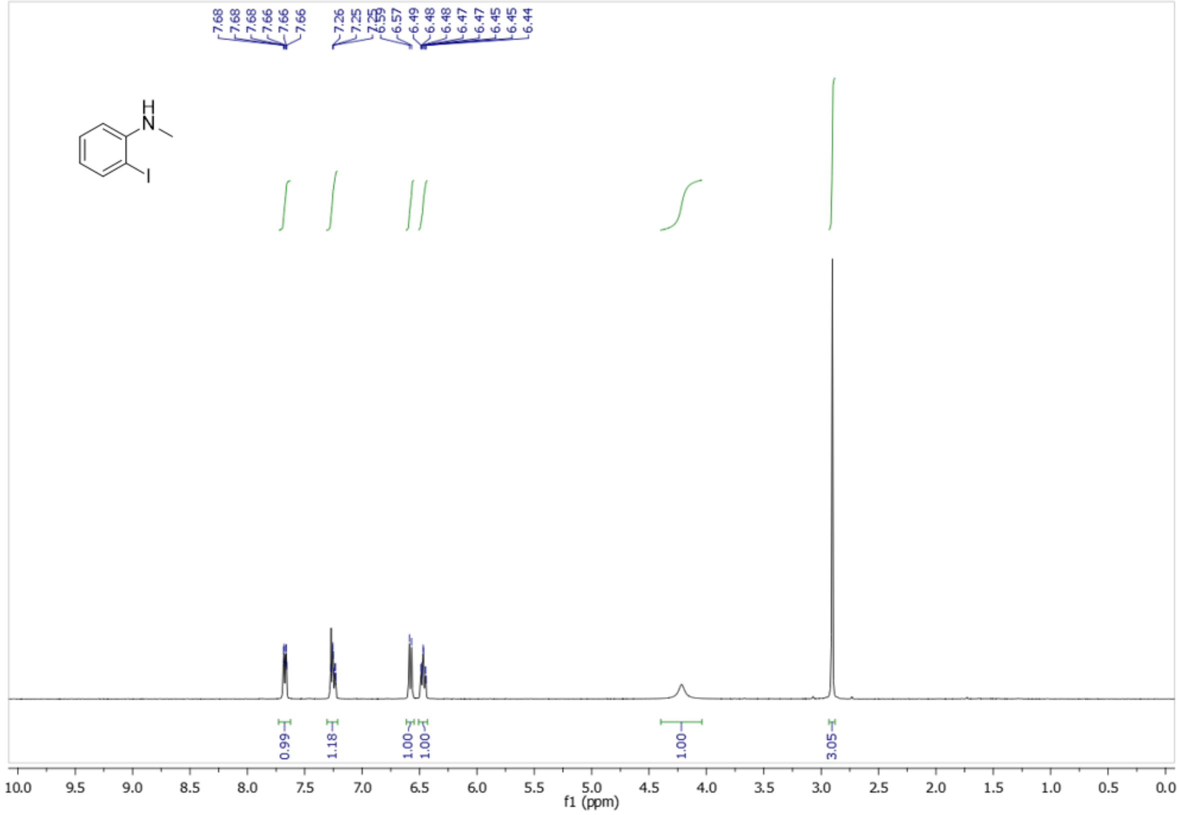

^{13}C NMR (101 MHz, $CDCl_3$):

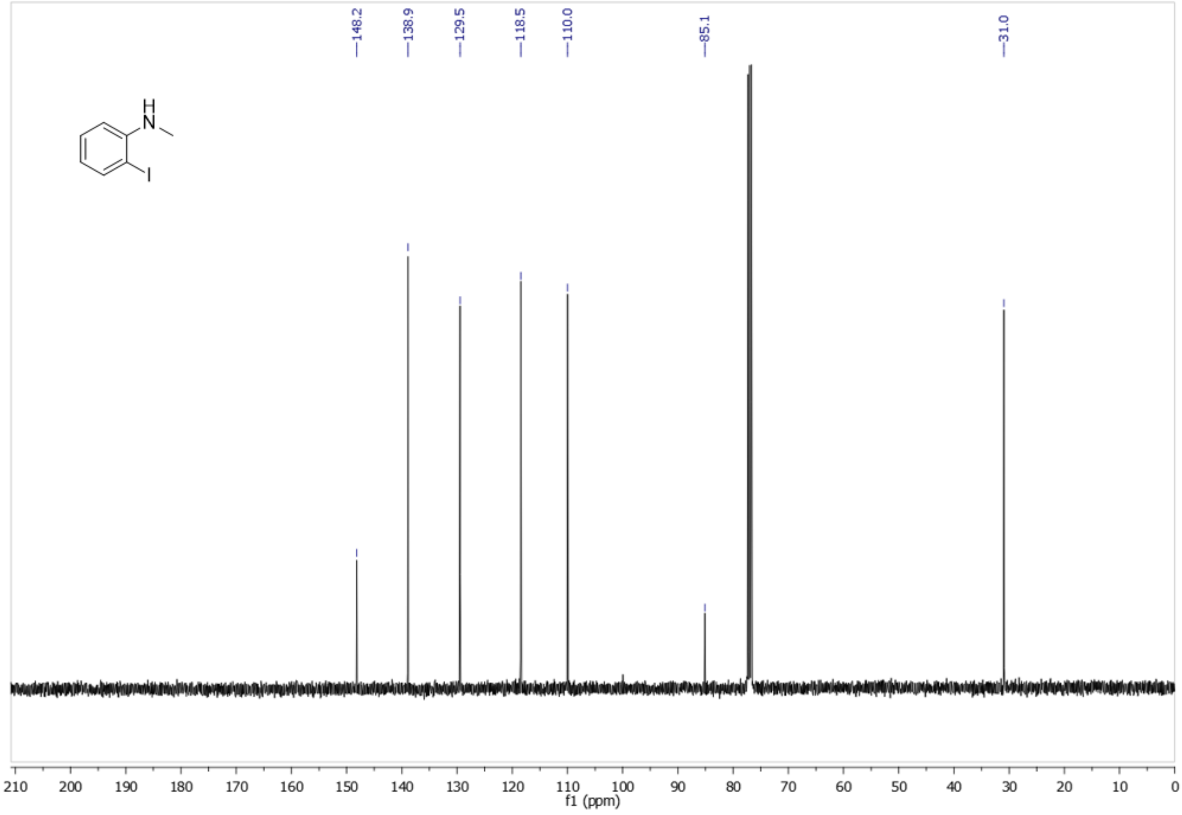

13.64 *N*-Allyl-2-iodo-*N*-methylaniline

^{1}H NMR (400 MHz, $CDCl_3$):

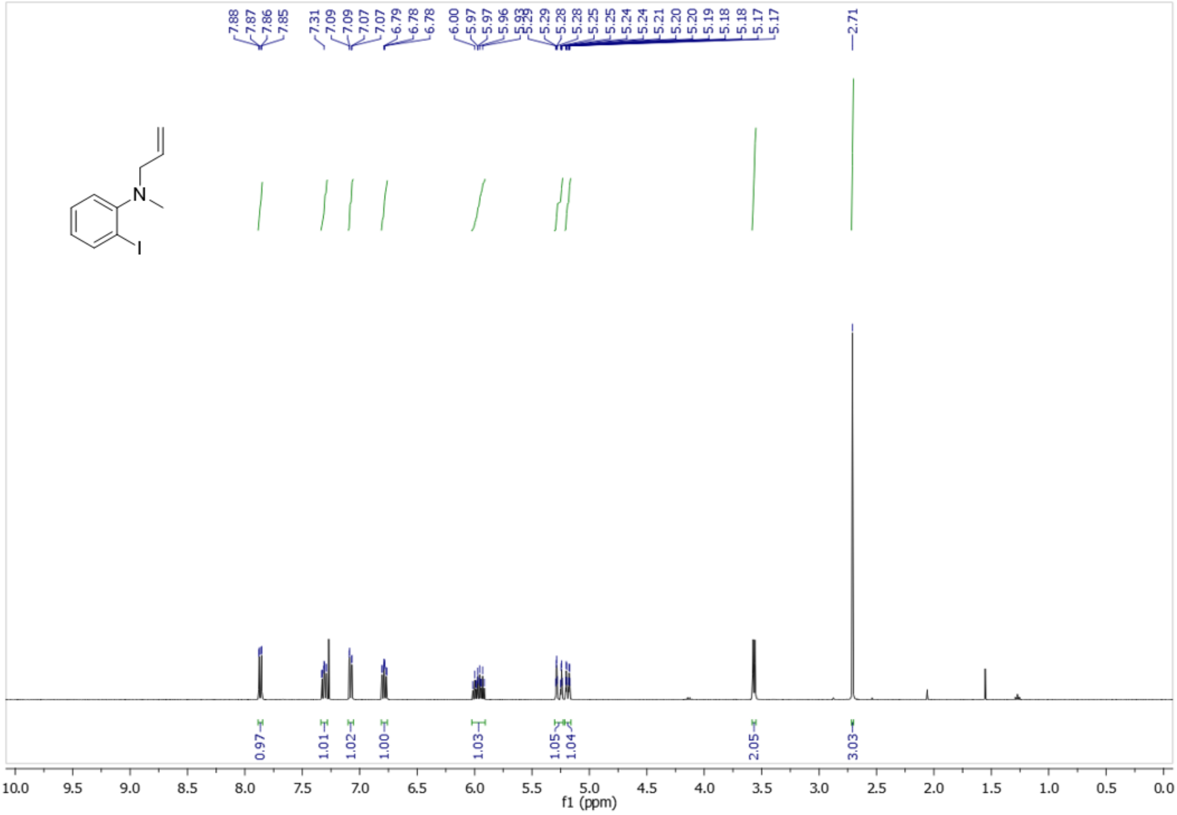

^{13}C NMR (101 MHz, $CDCl_3$):

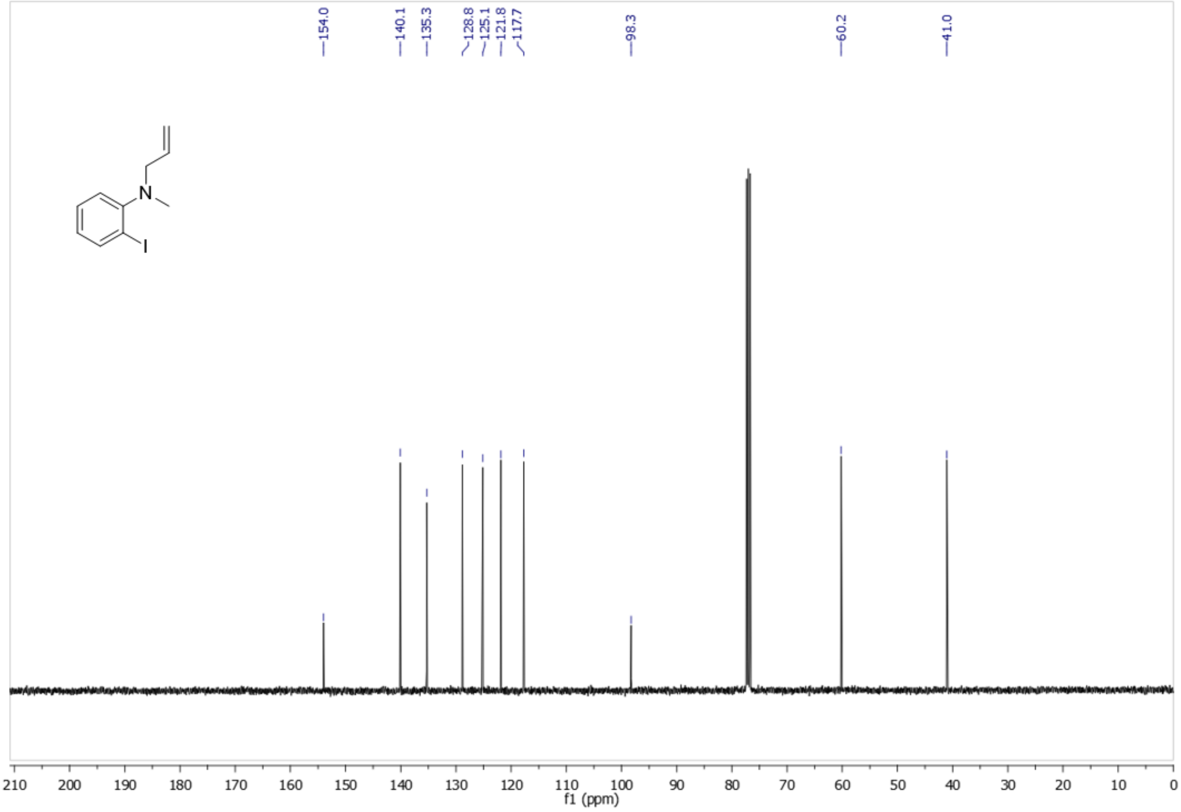

13.65 1,3-Dimethylindoline (28)

^{1}H NMR (400 MHz, $CDCl_3$):

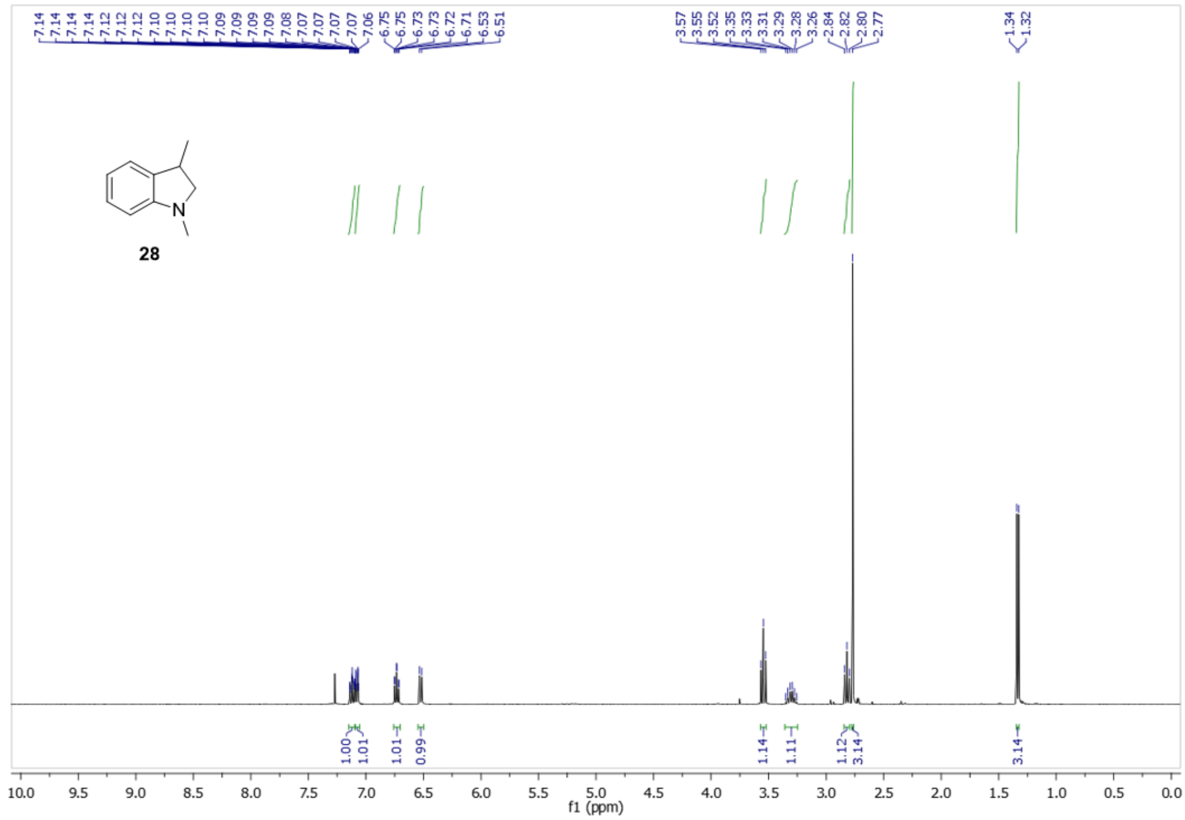

^{13}C NMR (101 MHz, $CDCl_3$):

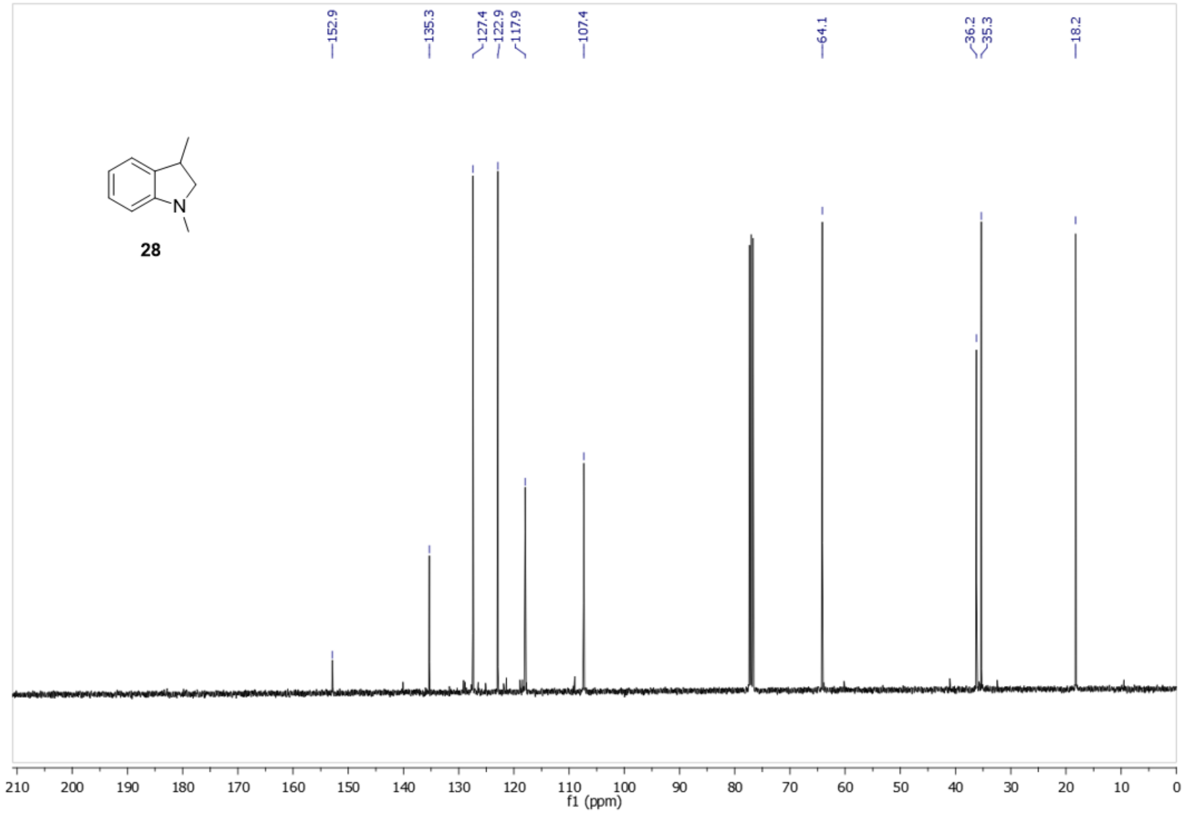

13.66 Ethyl (*E*)-3-cyclopropylacrylate

^{1}H NMR (400 MHz, $CDCl_3$):

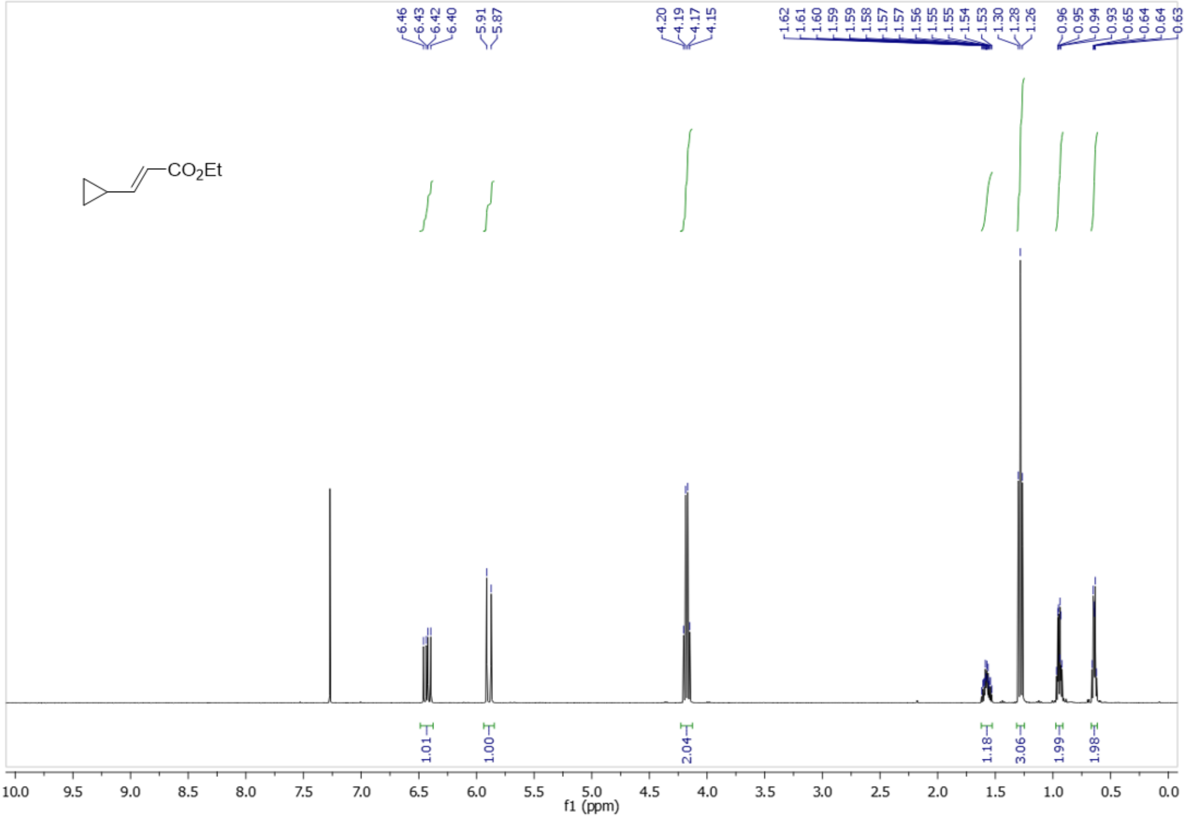

^{13}C NMR (101 MHz, $CDCl_3$):

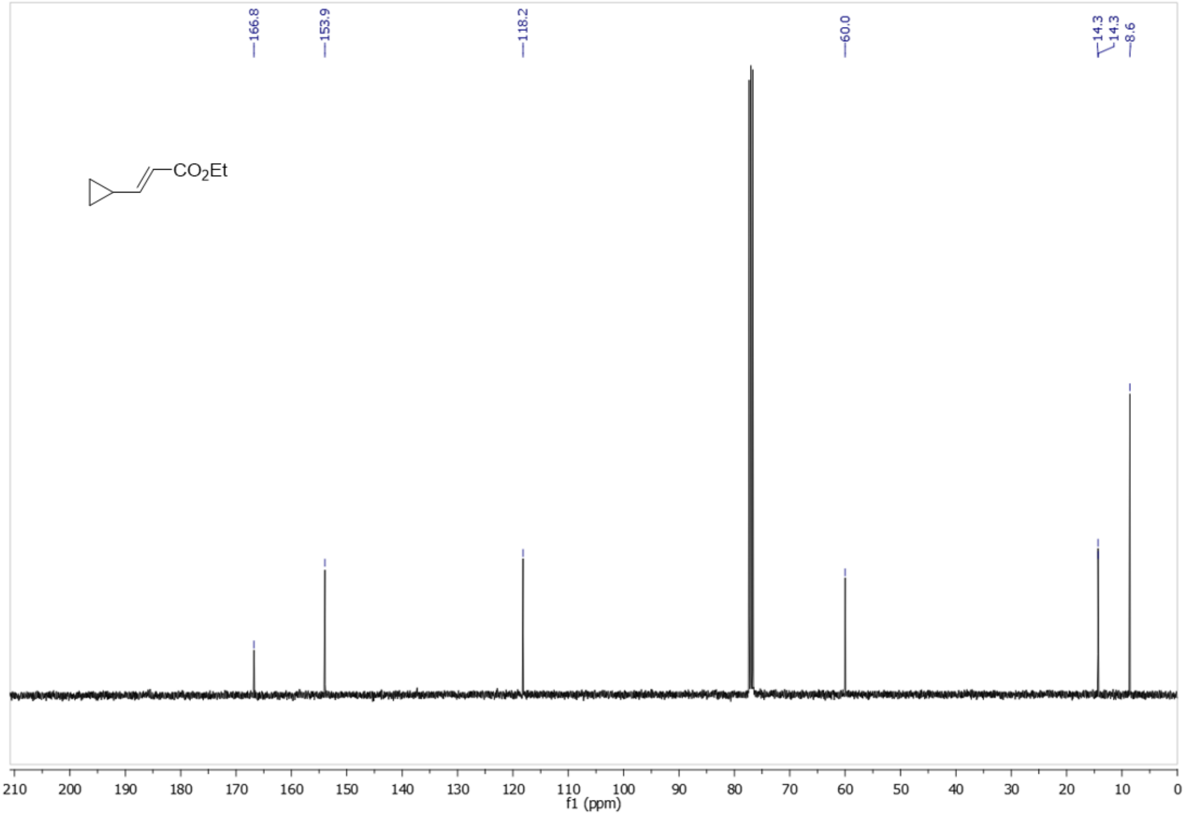

13.67 (*E*)-3-Cyclopropylprop-2-en-1-ol

^{1}H NMR (400 MHz, $CDCl_3$):

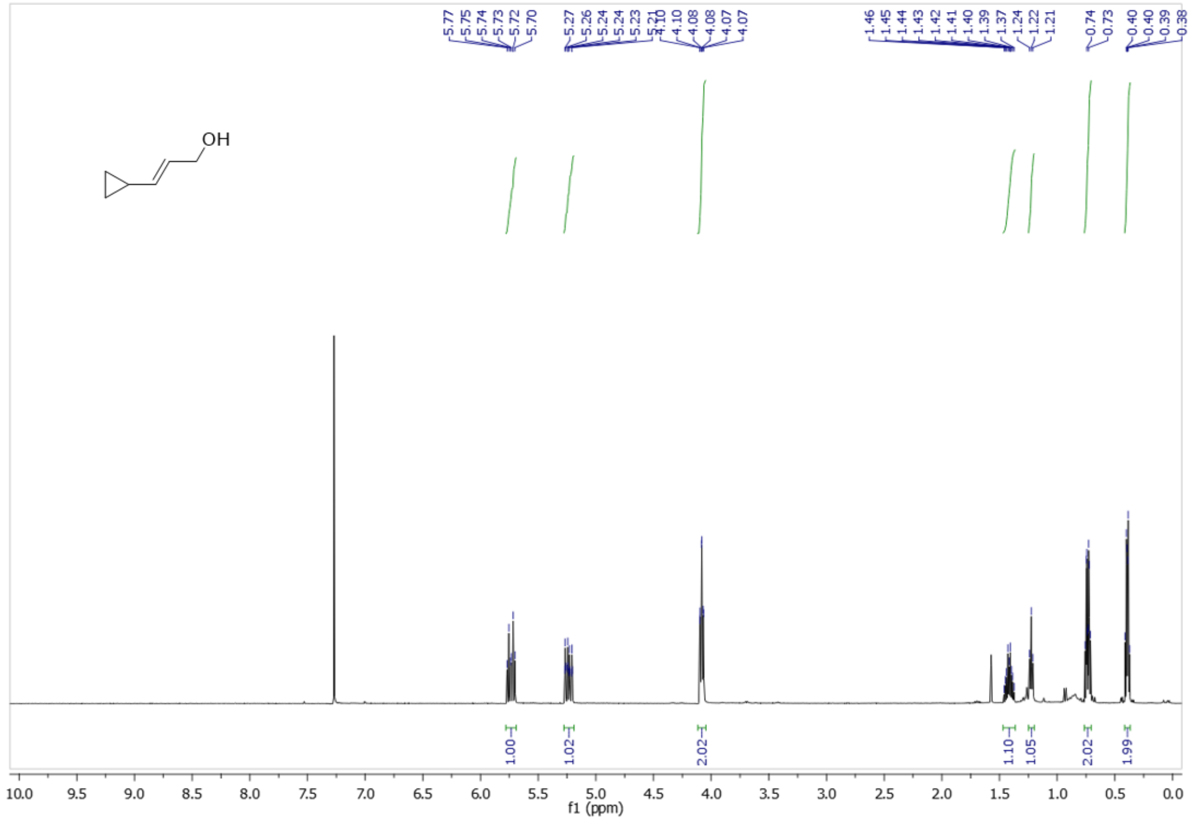

^{13}C NMR (101 MHz, $CDCl_3$):

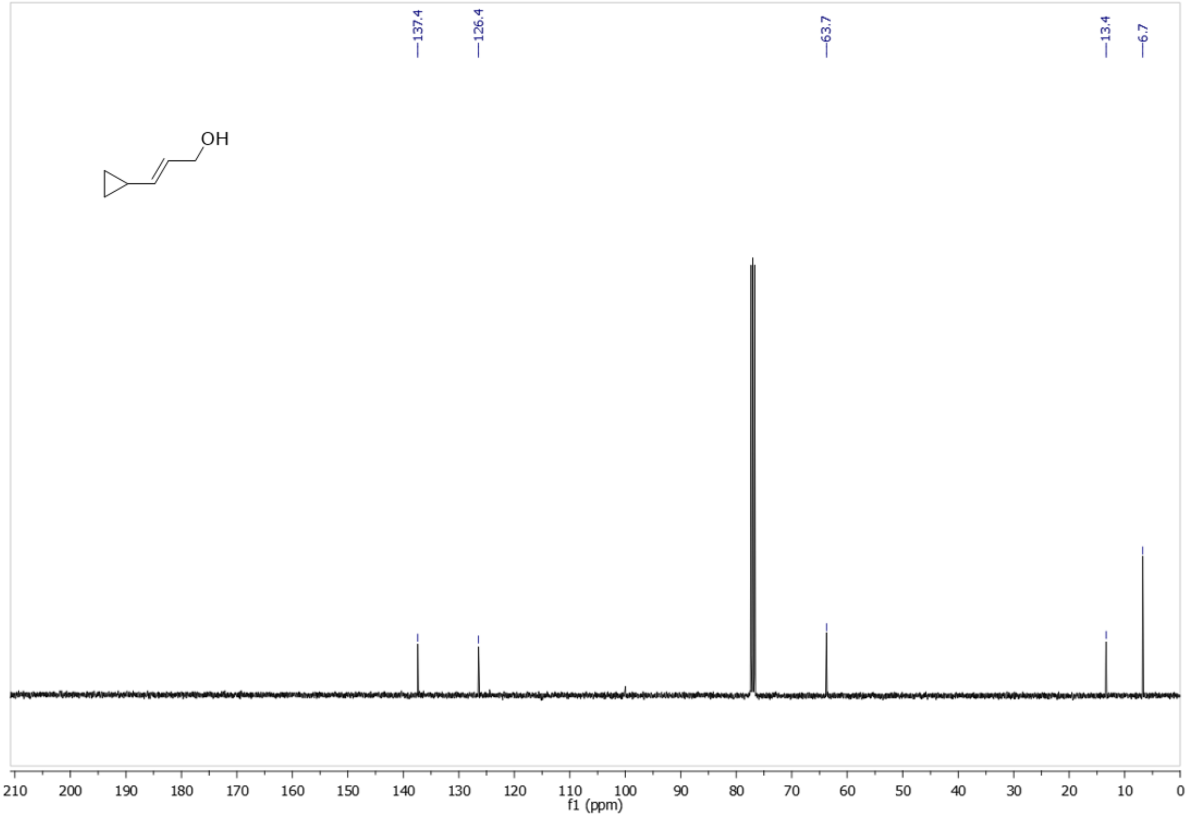

13.68 1-((3-Cyclopropylallyl)oxy)-2-iodobenzene (SI1, *E*:*Z* ~ 7:1)

^{1}H NMR (400 MHz, $CDCl_3$):

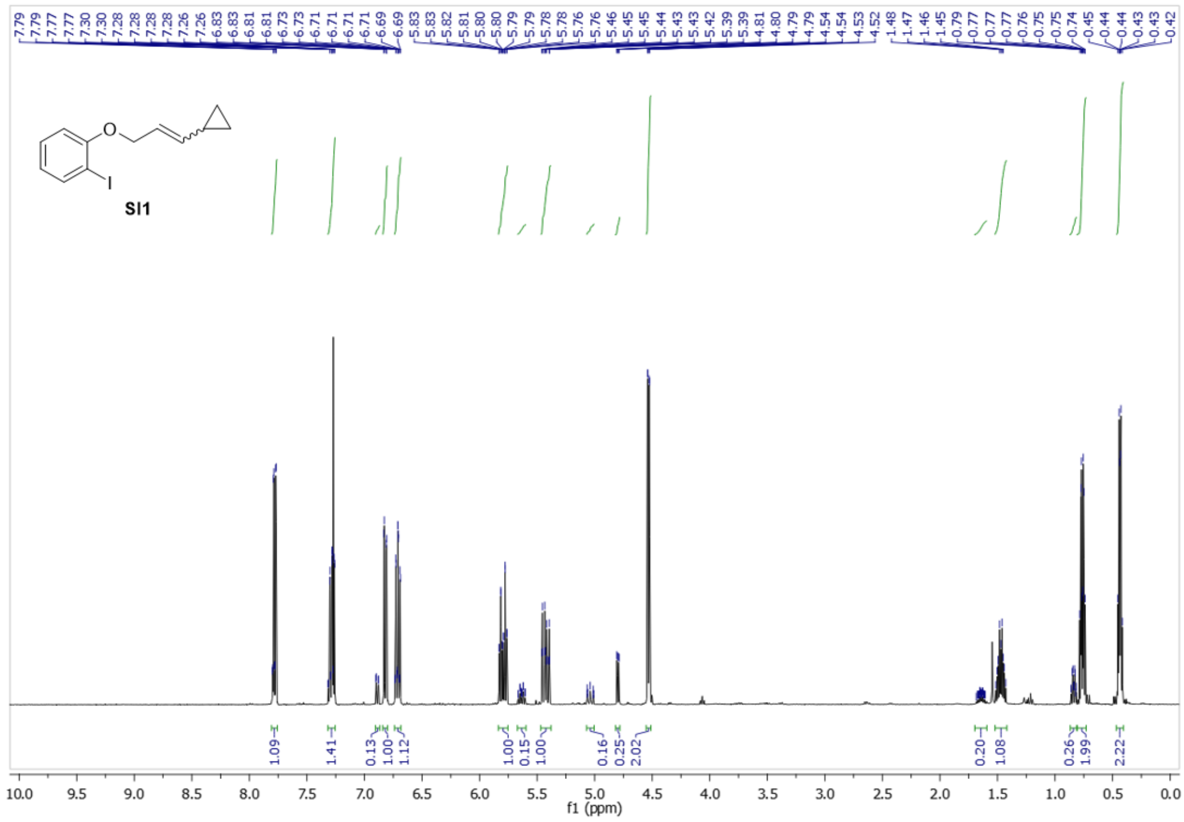

^{13}C NMR (101 MHz, $CDCl_3$):

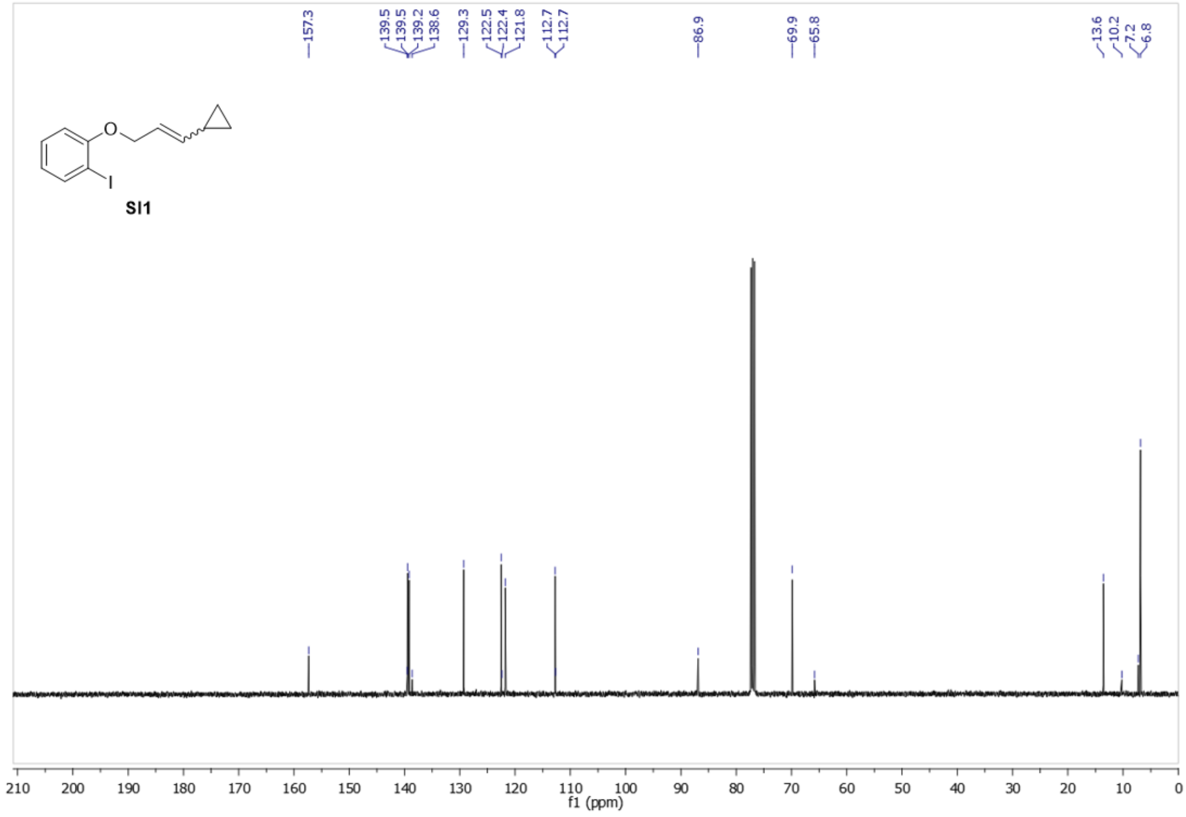

13.69 3-(But-1-en-1-yl)-2,3-dihydrobenzofuran (SI2, *E:Z* ~ 3:1)

^{1}H NMR (400 MHz, $CDCl_3$):

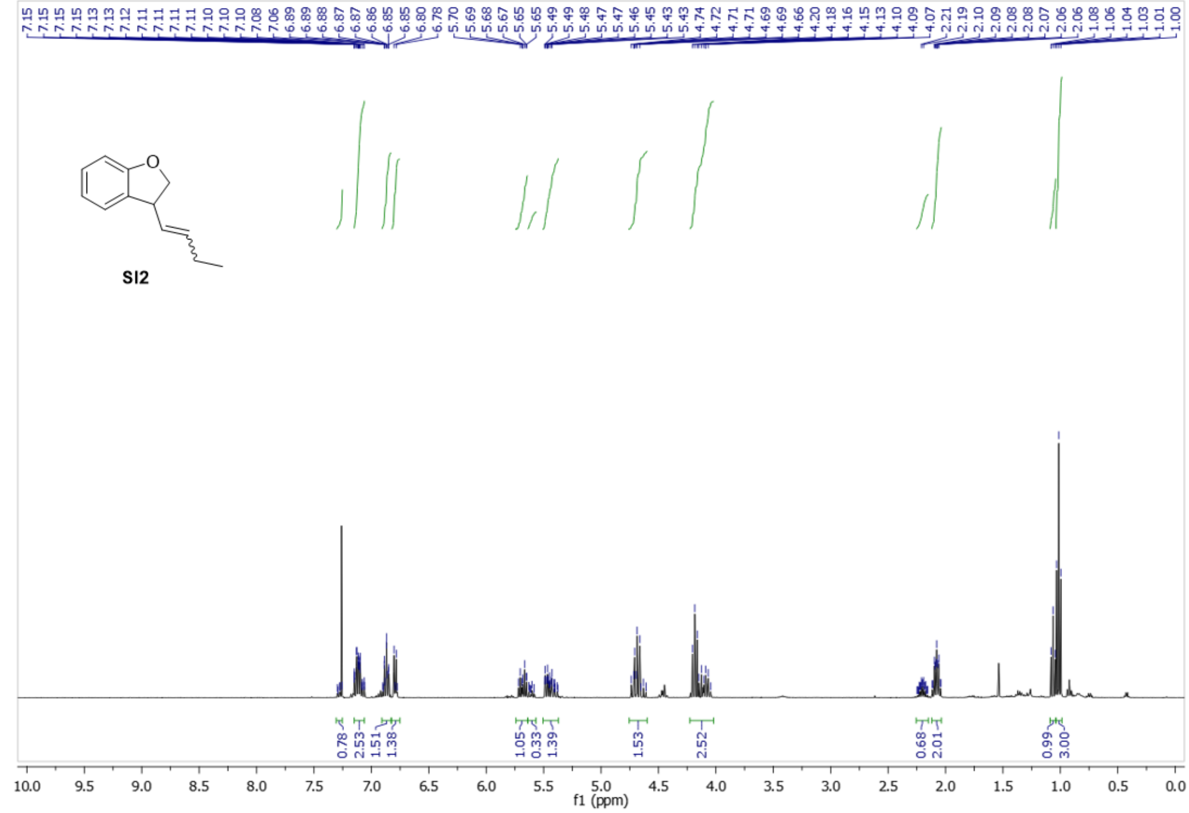

^{13}C NMR (101 MHz, $CDCl_3$):

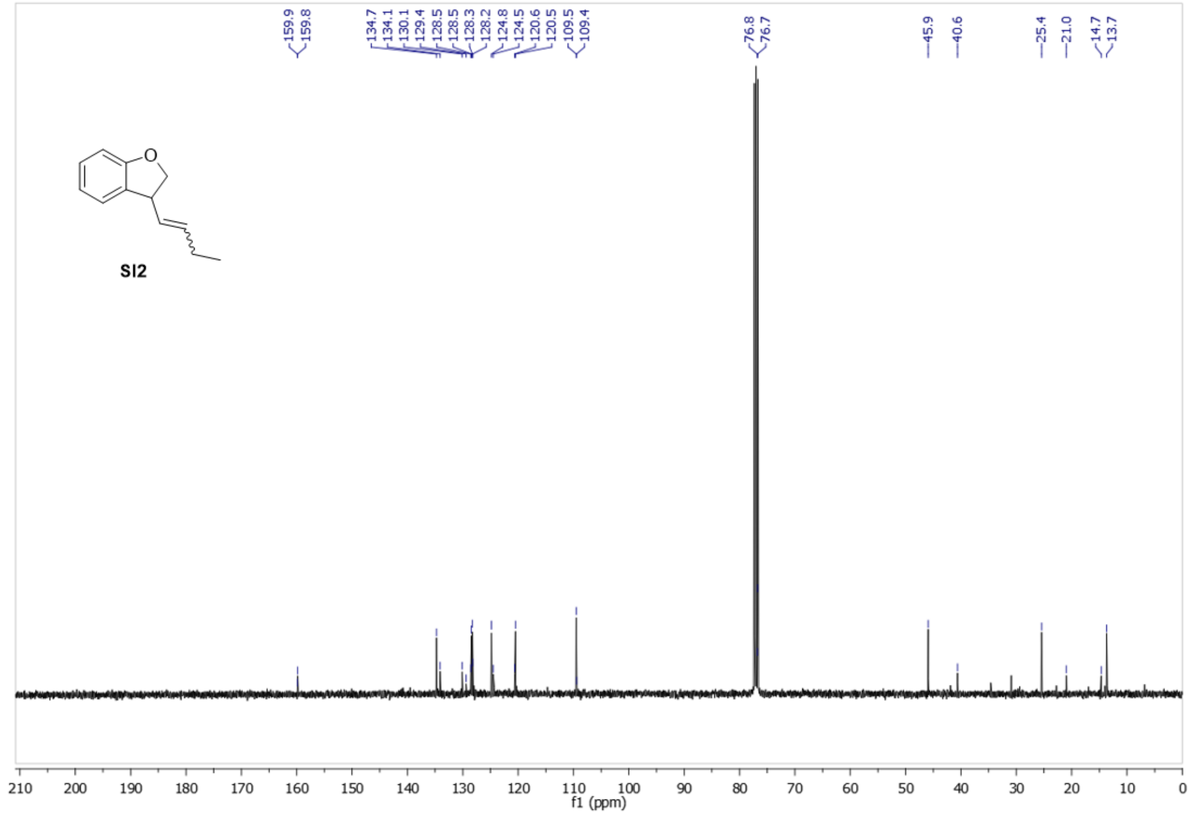

13.70 Tetrabutylammonium triiodide

^{1}H NMR (400 MHz, $CDCl_3$):

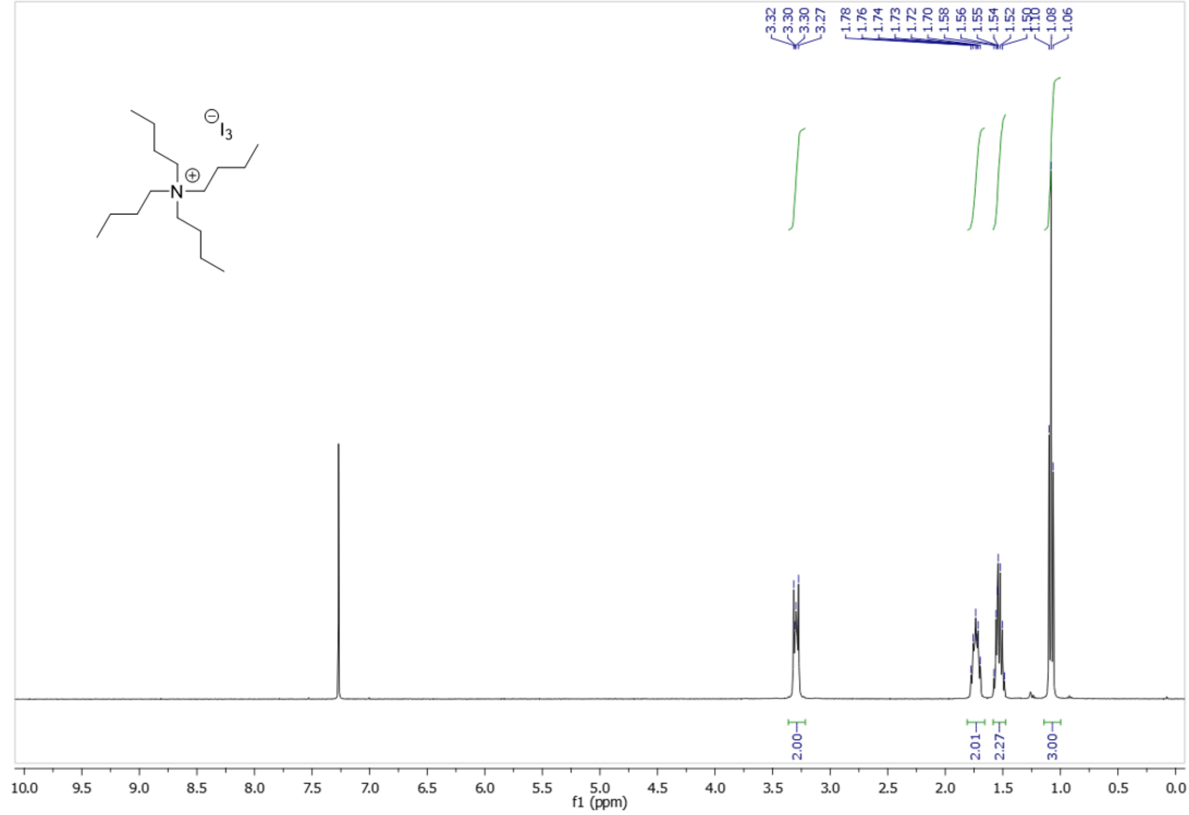

^{13}C NMR (101 MHz, $CDCl_3$):

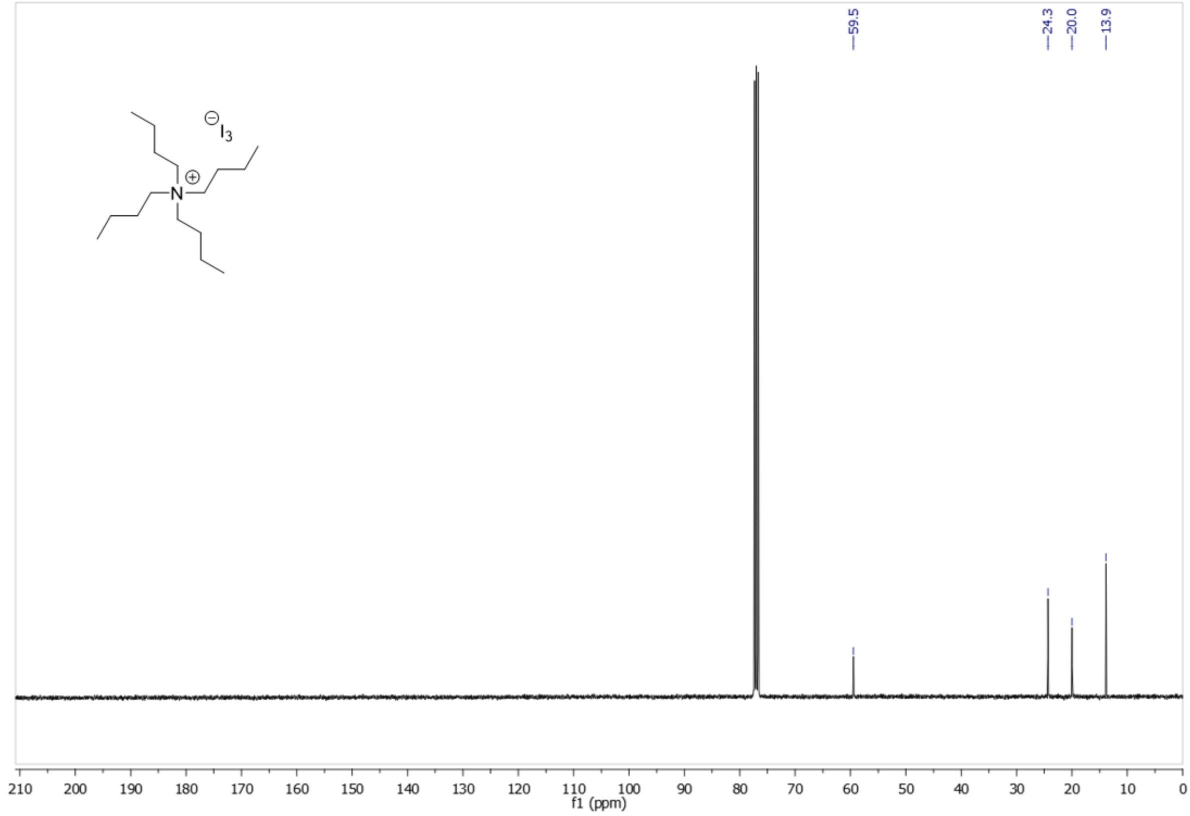

Supplement: Supplementary file 2 — Supporting Information [file ANIE-61-0-s002.pdf]
